# Identification of candidate chemosensory genes of *Ophraella communa* LeSage (Coleoptera: Chrysomelidae) based on antennal transcriptome analysis

**DOI:** 10.1038/s41598-019-52149-x

**Published:** 2019-10-29

**Authors:** Chao Ma, Chenchen Zhao, Shaowei Cui, Yan Zhang, Guangmei Chen, Hongsong Chen, Fanghao Wan, Zhongshi Zhou

**Affiliations:** 1 State Key Laboratory for Biology of Plant Diseases and Insect Pests, Institute of Plant Protection, Chinese Academy of Agricultural Sciences, Beijing, 100193 China; 2 School of Plant Protection, Jilin Agricultural University, Changchun, 130118 China; 3 Guangxi Key Laboratory for Biology of Crop Diseases and Insect Pests, Institute of Plant Protection, Guangxi Academy of Agricultural Sciences, Nanning, 530007 China

**Keywords:** Transcriptomics, Chemical ecology

## Abstract

Antennal olfaction plays a key role in insect survival, which mediates important behaviors like host search, mate choice, and oviposition site selection. As an oligophagous insect, olfaction is extremely important for *Ophraella communa* to locate host plants. However, information on the olfactory genes has been lacking in *O*. *communa*. Using next generation sequencing, we assembled the antennal transcriptome of *O*. *communa* and first reported the major chemosensory genes necessary for olfaction in this species. In this study, a total 105 candidate chemosensory genes were identified in *O*. *communa* antennae, including 25 odorant-binding proteins (OBPs), 11 chemosensory proteins (CSPs), four sensory neuron membrane proteins (SNMPs), 30 odorant receptors (ORs), 18 ionotropic receptors (IRs), and 17 gustatory receptors (GRs). We also identified full-length sequences of the highly conserved ORco and IR8a/25a family in *O*. *communa*. In addition, the expression profile of 15 ORs and four OBPs were validated by quantitative real-time polymerase chain reaction (qPCR). We found that *OcomOR2*/4/*19* and *OcomOBP19*/*20* had a biased expression in male antennae, and *OcomOR8* had a biased expression in the female antennae. This large number of chemosensory genes handled by homology analysis and qPCR results will provide the first insights into molecular basis for the olfactory systems of *O*. *communa* as well as advance our understanding of olfactory mechanisms in Coleoptera.

## Introduction

The olfactory system is very important to insects because it is involved in various insect behaviors, such as locating suitable hosts, avoiding predators, identifying oviposition sites, and finding sexual partners^1^. The antenna is the major organ for insect olfactory sensing, especially for olfaction. Mounting evidence suggests that diverse olfactory genes are involved in the signal recognition process, including odorant receptors (ORs), ionotropic receptors (IRs), odorant binding proteins (OBPs), gustatory receptors (GRs), chemosensory proteins (CSPs), and sensory neuron membrane proteins (SNMPs)^2–4^. Olfactory signal transduction can be summarized as follows: Firstly, the hydrophobic chemical compounds penetrate the sensillar lymph through pores, wherein they are recognized and bound by OBPs^5,6^ or CSPs^7^. Secondly, it was speculated that the OBPs or CSPs were the transporters that transferred odorants through the sensillar lymph to ORs, a family of integral membrane proteins, located on the dendrites of olfactory receptor neurons (ORNs)^8–10^. Additionally, SNMPs^11^, IRs^12,13^ and GRs^14^ have also been proposed to play a role in insect olfaction.

Although insect ORs are seven-transmembrane domain (TMD) proteins with a reversed membrane topology (intracellular N-terminus)^15,16^, they do not belong to the G protein-coupled receptors. In the transduction process, ORs appear to be the primary mechanism by which insects detect volatile chemicals, facilitating the conversion of the chemical message to an electrical signal, such as a biological transducer^17,18^. It is generally thought that each ORN expresses a highly conserved OR co-receptor (Orco protein) and a divergent, conventional ORx, such that the heterodimer of the Orco-OR forms an ion channel and mediates odorant-binding specificity^19–21^. ORs are broadly tuned to a variety of volatile chemicals, including pheromones, plant volatiles, and odor molecules present in the environment^17,22,23^.

Coleoptera species account for approximately 25% of all known species of animal life-forms^24^. Almost 40% of all previously described insect species are beetles^25^. However, compared with Lepidoptera, olfaction genes identified in Coleoptera are poorly known. To date, there are only about 20 species of Coleoptera for which olfactory genes have been identified, such as *Tribolium castaneum*^25,26^, *Megacyllene caryae*^27^, *Leptinotarsa decemlineata*^28^, *Phyllotreta striolata*^29^, *Colaphellus bowringi*^30^, *Pyrrhalta maculicollis*^31^, *P*. *aenescens*^31^, *Ambrostoma quadriimpressum*^32^ and *Galeruca daurica*^33^. Thus, much work is needed to investigate and better understand olfaction and its associated molecular biology in other species of Coleoptera. *Ophraella communa* LeSage (Coleoptera: Chrysomelidae) originated in North America, and it is considered a potential biological control agent of common ragweed, *Ambrosia artemisiifolia* L. (Asteraceae)^34^. Both adults and larvae feed on the leaves of common ragweed, resulting in severe defoliation^35^. Since the beetle was first discovered in Nanjing, Jiangsu province (China) in 2001^36^, it has been reported widely in eastern and central China, where it has significantly suppressed the population of common ragweed^37^. Zhou *et al*.^38^ reported that when the olfaction of male *O*. *communa* was hindered by covering their antennae with paint, the males spent significantly more time seeking mates in the arena, indicating that olfaction is important to the mating process. However, the molecular mechanism of olfaction recognition in this insect is still unknown. In this study, we performed a transcriptome analysis of the adult antennae of *O*. *communa* and identified 105 candidate chemosensory genes, including 30 ORs, 25 OBPs, 11 CSPs, 18 IRs, 17 GRs, and four SNMPs. Furthermore, we conducted a comprehensive and comparative phylogenetic analysis and examined 19 genes expression profiles using quantitative real-time polymerase chain reaction (qPCR). These results could help us better understand the olfactory signal transduction mechanisms in this insect.

## Results

### Transcriptome overview

**Figure 1:**
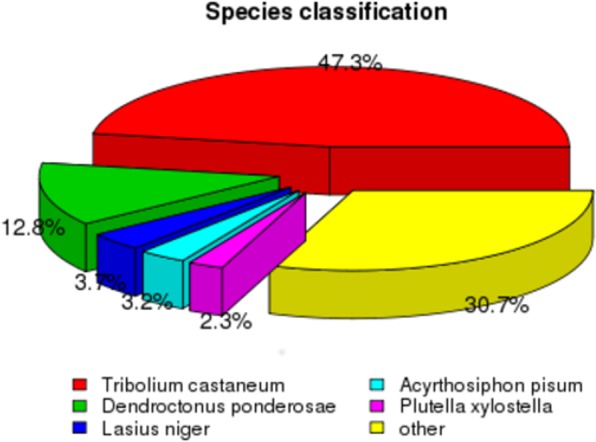
All unigenes sequences (92,259) that had blast annotations against the nr database with a cut-off E-value 10^−5^ were analyzed for species distribution.

Using an Illumina HiSeq 2000TM platform, a total of 31.1 million and 34.8 million raw reads were yielded, respectively, from the libraries of male and female antennae. After removing low-quality and adaptor reads, 29.4 million and 32.9 million clean-reads were generated. The total bases of sequence data were approximately 4.4 and 4.9 gigabases from male and female samples, respectively. Overall, 153,276 transcripts were generated, and we identified 92,259 unigenes by clustering and redundancy filtering. The mean length of unigenes was 1,229 nt and the N50 length reached 2,068 nt. In total, 35,508 unigenes were larger than 1,000 nt in length, which comprised 38.5% of all unigenes (Table 1). Homology searches of all unigenes with respect to other insect species showed that the highest percentage of unigenes matched *T*. *castaneum* (47.5%), followed by *Dendroctonus ponderosae* (12.8%), *Lasius niger* (3.7%), *Acyrthosiphon pisum* (3.2%), and *Plutella xylostella* (2.3%). The remaining 30.7% of the sequences showed similarity with the sequences of other insects (Fig. 1).

**Table 1:** Assembly summary of *O*. *communa* antennal transcriptome. nt = nucleotides, Gb = gigabases.

Transcripts	Total Number
Female antenna-Raw Reads (nt)	34,825,000
Male antenna-Raw Reads (nt)	31,106,518
Female antenna-Clean reads (nt)	32,944,204
Male antenna-Clean reads (nt)	29,369,754
Female antenna-Clean bases (Gb)	4.9
Male antenna-Clean bases (Gb)	4.4
Total transcripts	153,276
Total Unigene	92,259
N50 of unigenes (nt)	2,068
Mean length (nt)	1,229
Unigenes larger than 1,000 nt	35,508 (38.5%)
Unigenes with homolog in nr	43,779 (47.5%)
Unigenes annotated to GO term	33,258 (36.0%)

### OBPs

**Figure 2:**
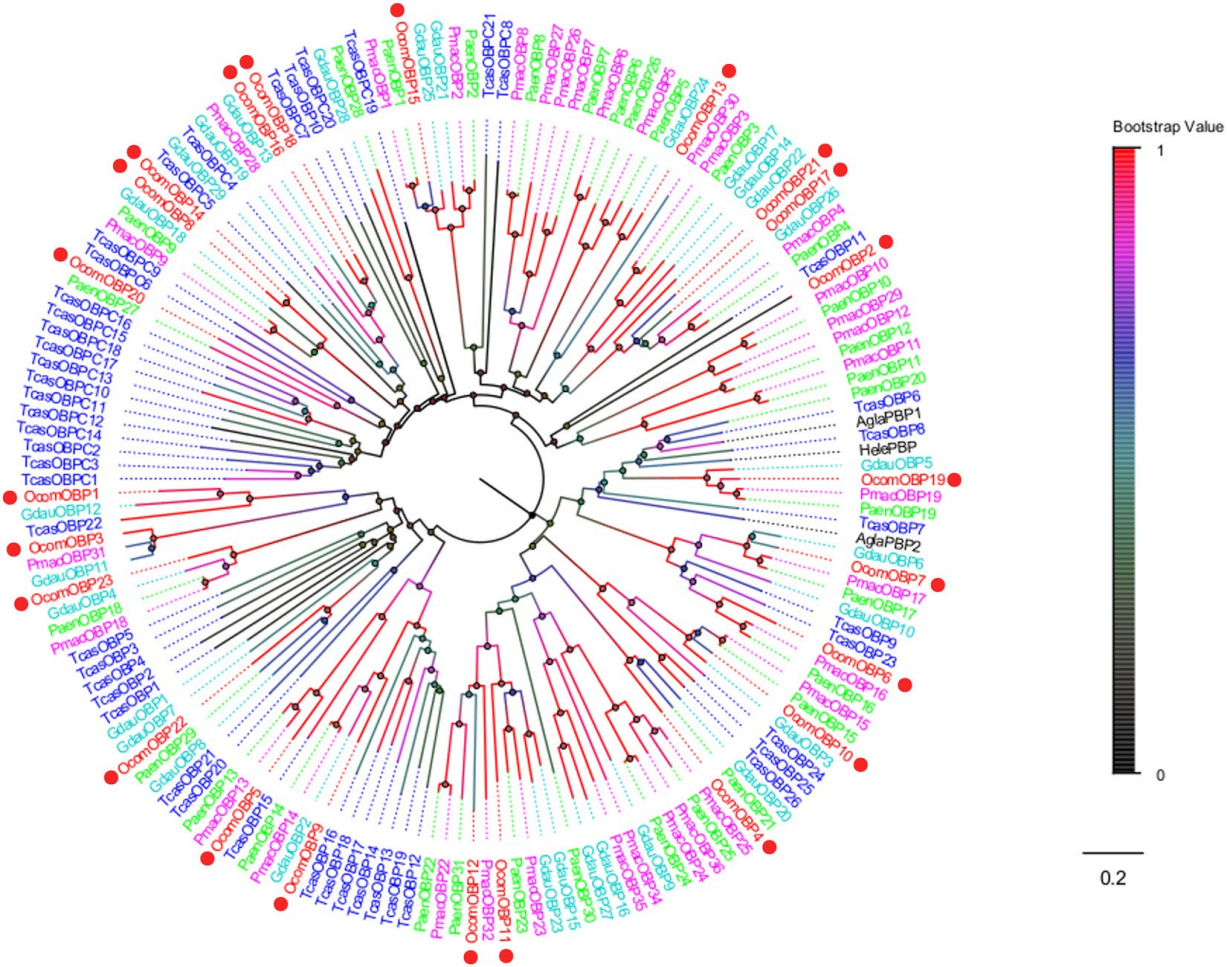
Neighbor joining phylogenetic tree of candidate *OcomOBPs* with known Coleopteran OBP sequences. Tcas, *Tribolium castaneum* (N = 47); Pmac, *Pyrrhalta maculicollis* (N = 33); Paen, *Pyrrhalta aenescens* (N = 31); Gdua, *Galeruca daurica* (N = 29); Agla, *Anoplophora glabripennis* (N = 2); Hele, *Hylamorpha elegans* (N = 1). Candidate *OcomOBPs* were indicated by red circles.

We identified 25 different sequences encoding odorant binding proteins in *O*. *communa* antennal transcriptomes. Sequence analysis results showed that 20 unigenes had a putative full-length open reading frame (ORF) and 19 unigenes had predicted signal peptide sequences. All of the candidate OBPs sequences Blastx best hits were similar to known Coleoptera OBPs. The length of all putative full-length *OcomOBPs* ranged from 119 to 198 amino acids. Compared to ORs, insect OBPs were highly conserved. Twenty-one of 25 putative OBPs had more than 50% similarity with OBPs from *G*. *daurica*, *P*. *maculicollis*, and *P*. *aenescens*. Based on phylogenetic analysis, *OcomOBPs* were split in various branches and they formed small subgroups together with OBPs from other beetles. These groups were strongly supported by high bootstrap values. Remarkably, we found *OcomOBP19*, a pheromone binding protein (PBP), which clustered with other Coleoptera PBPs in a clade (Fig. 2). Information, including unigene reference, length, and best Blastx hit for all 25 OBPs are listed in Table 2.

**Table 2:** The Blastx match of *O*. *communa* candidate candidate odorant binding proteins.

Unigene ID	NAME	Length	ORF (aa)	Status	Signal peptide	Blastx best-hit	Species	Score	E-value	Ident	Accession
Cluster-9750.32846	OcomOBP1	1109	198	Complete ORF	Y	odorant-binding protein	*Galeruca daurica*	188	5.00E-55	54%	AQY18976.1
Cluster-9750.2277	OcomOBP2	604	192	3′ missing	Y	odorant binding protein 25	*Colaphellus bowringi*	154	3.00E-43	45%	ALR72513.1
Cluster-9750.23752	OcomOBP3	1228	182	Complete ORF	Y	odorant-binding protein 31	*Pyrrhalta maculicollis*	312	3.00E-103	82%	APC94203.1
Cluster-9750.32204	OcomOBP4	593	169	Complete ORF	N	odorant-binding protein 21	*Pyrrhalta maculicollis*	267	2.00E-61	61%	APC94176.1
Cluster-3339.0	OcomOBP5	551	153	Complete ORF	Y	odorant-binding protein 13, partial	*Pyrrhalta aenescens*	232	1.00E-75	71%	APC94286.1
Cluster-9750.21729	OcomOBP6	738	153	Complete ORF	N	odorant-binding protein 16	*Pyrrhalta aenescens*	219	5.00E-69	71%	APC94289.1
Cluster-9750.32217	OcomOBP7	644	152	Complete ORF	Y	odorant-binding protein 17	*Pyrrhalta aenescens*	240	2.00E-78	79%	APC94290.1
Cluster-17495.0	OcomOBP8	551	151	Complete ORF	N	odorant-binding protein 9	*Pyrrhalta aenescens*	177	5.00E-54	64%	APC94272.1
Cluster-9750.33001	OcomOBP9	657	148	Complete ORF	Y	odorant-binding protein 14	*Pyrrhalta aenescens*	193	9.00E-60	60%	APC94287.1
Cluster-9750.32206	OcomOBP10	738	146	Complete ORF	N	odorant-binding protein 15	*Pyrrhalta aenescens*	254	4.00E-83	88%	APC94288.1
Cluster-9853.0	OcomOBP11	628	144	Complete ORF	Y	odorant-binding protein 23	*Pyrrhalta maculicollis*	139	1.00E-38	48%	APC94180.1
Cluster-1804.1	OcomOBP12	693	144	Complete ORF	Y	odorant-binding protein 22	*Pyrrhalta maculicollis*	122	2.00E-31	44%	APC94177.1
Cluster-8649.0	OcomOBP13	748	141	Complete ORF	Y	odorant-binding protein	*Galeruca daurica*	238	6.00E-77	79%	AQY18988.1
Cluster-15202.0	OcomOBP14	493	141	Complete ORF	Y	odorant-binding protein 9	*Pyrrhalta maculicollis*	197	3.00E-62	62%	APC94188.1
Cluster-9750.28158	OcomOBP15	603	137	Complete ORF	Y	odorant-binding protein	*Galeruca daurica*	222	1.00E-71	82%	AQY18989.1
Cluster-9750.32896	OcomOBP16	778	135	Complete ORF	Y	odorant-binding protein 28	*Pyrrhalta maculicollis*	177	5.00E-53	69%	APC94187.1
Cluster-16599.0	OcomOBP17	418	135	3′ missing	Y	odorant-binding protein	*Galeruca daurica*	160	6.00E-48	62%	AQY18990.1
Cluster-9750.26603	OcomOBP18	412	131	3′ missing	Y	odorant-binding protein 28	*Pyrrhalta maculicollis*	177	8.00E-55	62%	APC94187.1
Cluster-9750.32590	OcomOBP19	819	131	Complete ORF	Y	odorant-binding protein 19	*Pyrrhalta aenescens*	216	5.00E-68	87%	APC94292.1
Cluster-9750.34868	OcomOBP20	574	130	Complete ORF	Y	odorant-binding protein 7	*Monochamus alternatus*	142	2.00E-19	37%	AIX97022.1
Cluster-11640.0	OcomOBP21	401	127	Complete ORF	Y	odorant-binding protein 4	*Pyrrhalta aenescens*	138	4.00E-39	61%	APC94279.1
Cluster-9750.24950	OcomOBP22	540	120	Complete ORF	Y	odorant-binding protein	*Galeruca daurica*	118	4.00E-31	50%	AQY18972.1
Cluster-9750.33322	OcomOBP23	618	119	Complete ORF	Y	odorant-binding protein 18	*Pyrrhalta aenescens*	166	2.00E-49	74%	APC94291.1
Cluster-9750.56133	OcomOBP24	513	99	5′ missing	N	odorant-binding protein 28	*Pyrrhalta maculicollis*	132	2.00E-36	55%	APC94187.1
Cluster-9750.32950	OcomOBP25	562	95	5′ missing	N	odorant-binding protein 21	*Pyrrhalta aenescens*	254	2.00E-57	66%	APC94263.1

### CSPs

**Figure 3:**
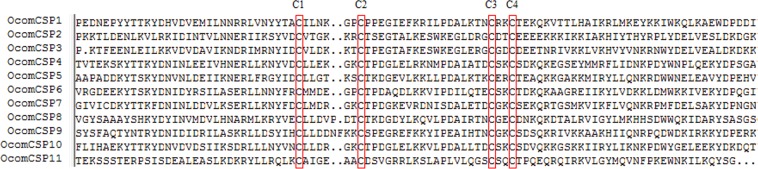
Sequences alignment of candidate *OcomCSPs* amino acid sequences. The conserved cysteine residues were marked with red box.

**Figure 4:**
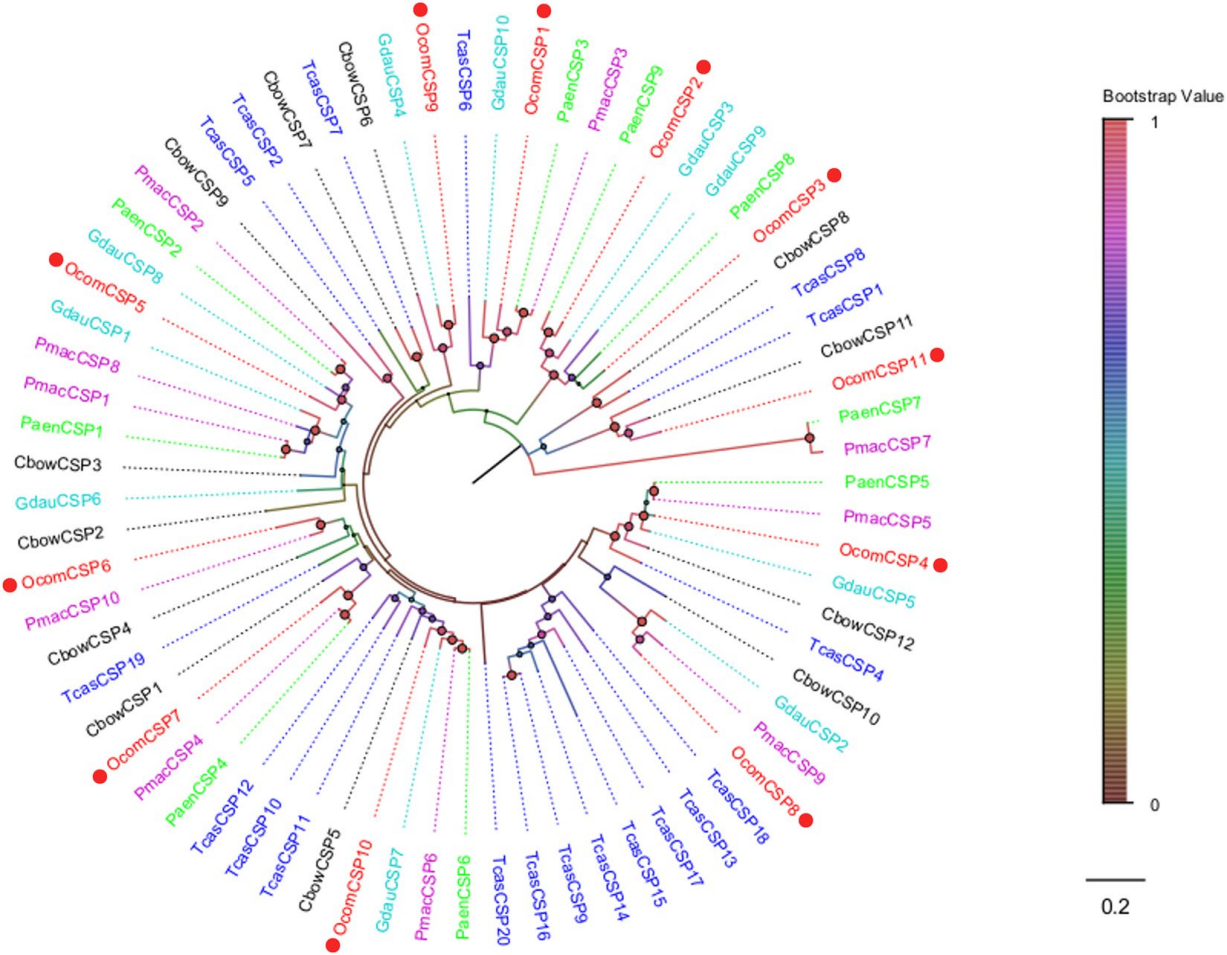
Neighbor joining phylogenetic tree of candidate *OcomCSPs* with known Coleopteran CSP sequences. Tcas, *Tribolium castaneum* (N = 19); Pmac, *Pyrrhalta maculicollis* (N = 10); Paen, *Pyrrhalta aenescens* (N = 9); Gdua, *Galeruca daurica* (N = 10); Cbow, *Colaphellus bowringi* (N = 12). Candidate *OcomCSPs* were indicated by red circles.

We identified 11 unigenes encoding candidate chemosensory proteins in *O*. *communa* antennal transcriptome. Notably, all putative chemosensory proteins were predicted with a putative full-length ORF and signal peptide through sequence analysis. The length of all putative full-length *OcomCSPs* ranged from 118 to 261 amino acids. In addition, all of the *OcomCSPs* followed the highly conserved pattern with four cysteines arranged with an exact spacing of C_1_X_6_C_2_X_18_C_3_X_2_C_4_ (Fig. 3). Insect CSPs are more conserved than ORs or OBPs, and all *OcomCSPs* amino acid sequences have more than 65% similarity with CSPs from *P*. *maculicollis*, *P*. *aenescens*, *G*. *daurica*, and *C*. *bowringi*. Homology analysis showed that the *OcomCSPs* were present on different branches throughout the dendrogram and supported by high bootstrap values (Fig. 4). Information, including unigene reference, length, and the best Blastx hit of all 11 CSPs are listed in Supplementary Material S3.

### SNMPs

**Figure 5:**
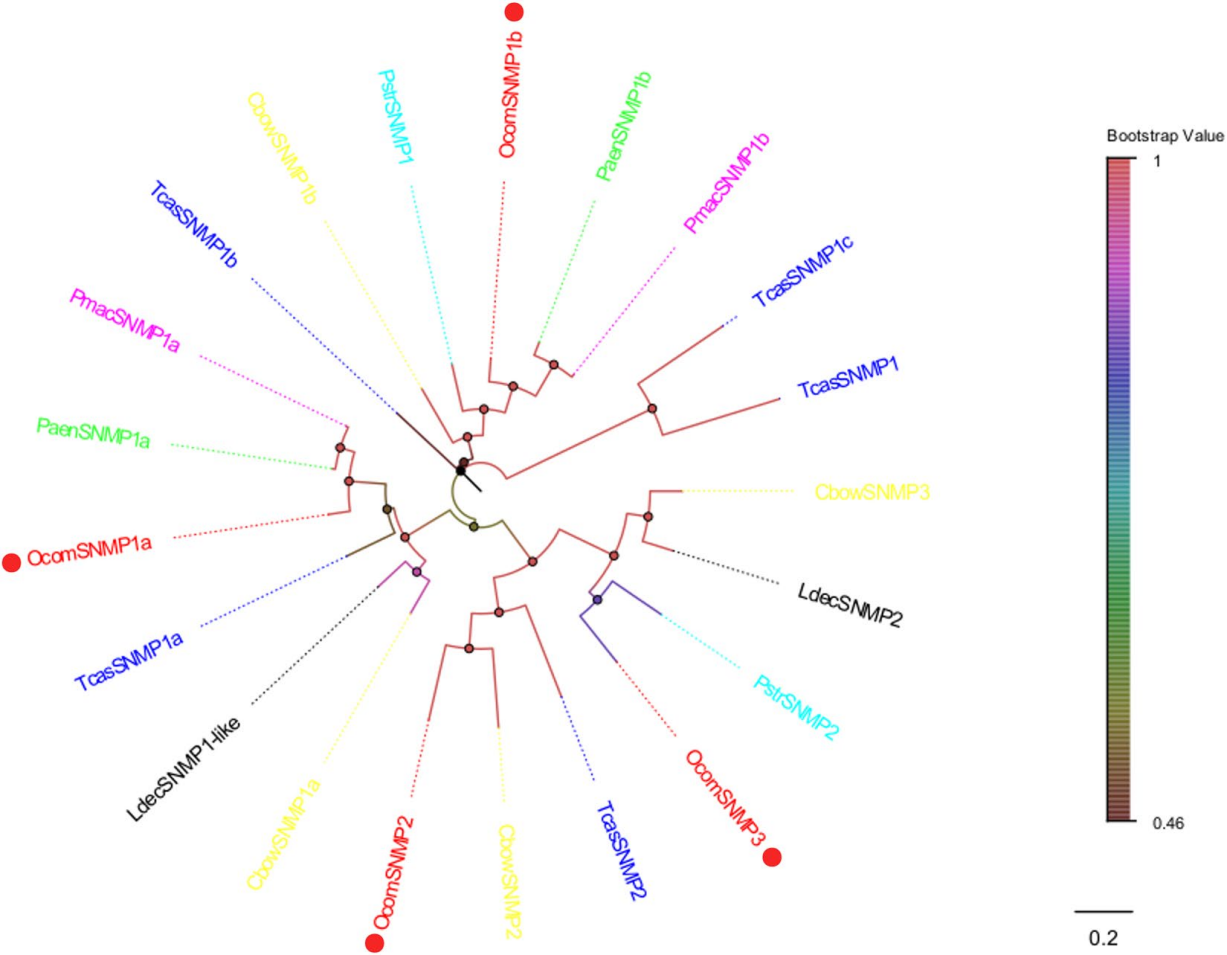
Neighbor joining phylogenetic tree of candidate *OcomSNMPs* with known Coleopteran SNMP sequences. Tcas, *Tribolium castaneum* (N = 5); Pmac, *Pyrrhalta maculicollis* (N = 2); Paen, *Pyrrhalta aenescens* (N = 2); Cbow, *Colaphellus bowringi* (N = 4); Ldec, *Leptinotarsa decemlineata* (N = 2); Pstr, *Phyllotreta striolata* (N = 2). Candidate *OcomSNMPs* were indicated by red circles.

We identified four SNMP genes in the antennal transcriptome (Fig. 5). Lengths of all candidate *OocmSNMPs* were over 500 amino acids and three of them were predicted to have a putative full-length ORF. Furthermore, all *OocmSNMPs* had more than 50% identity with SNMPs of *P*. *aenescens*, *P*. *striolata*, and *C*. *bowringi*. Information, including unigene reference, length, and the best Blastx hit of all four SNMPs are listed in Supplementary Material S3.

### ORs

**Figure 6:**
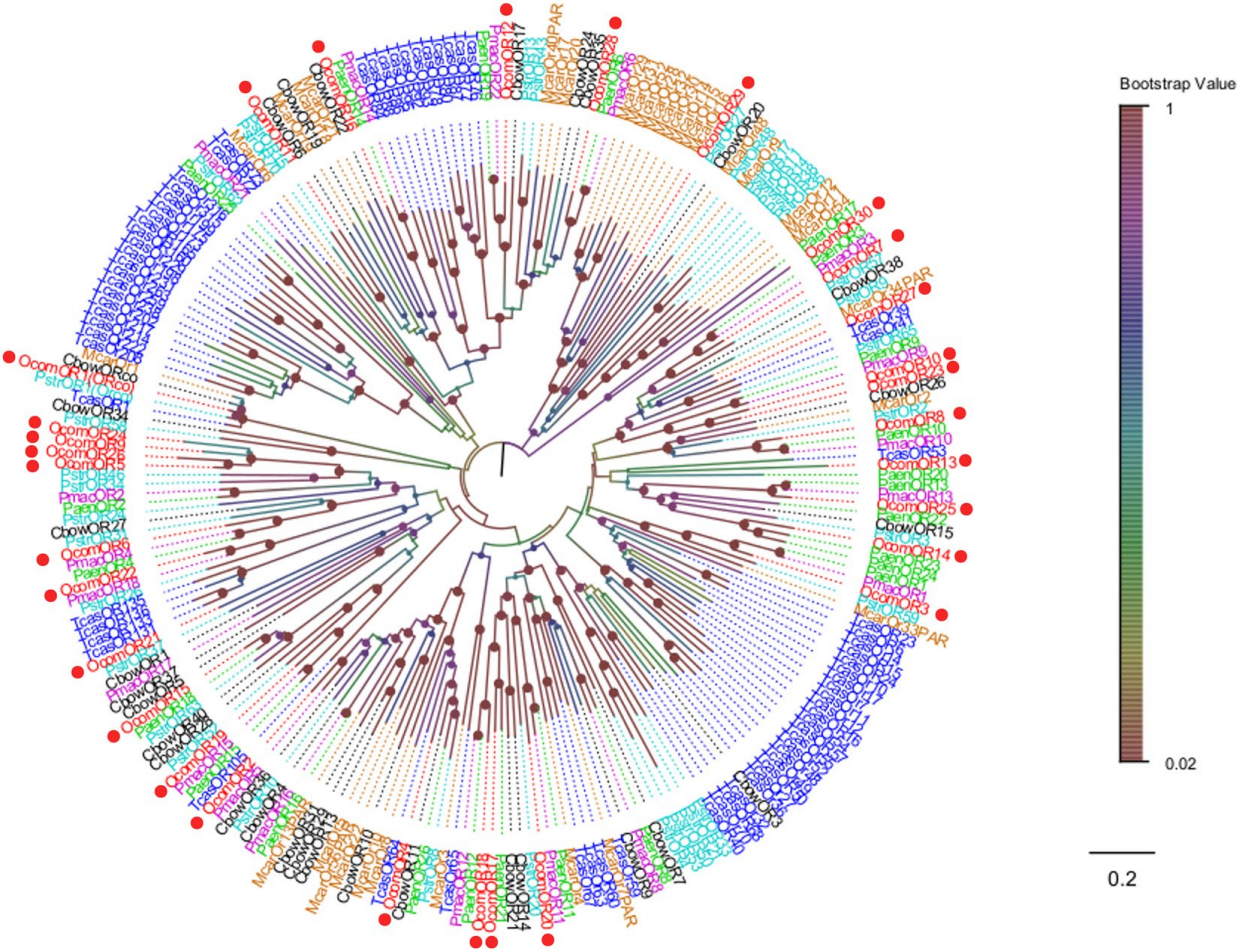
Neighbor joining phylogenetic tree of candidate *OcomORs* with known Coleopteran OR sequences. Tcas, *Tribolium castaneum* (N = 70); Pmac, *Pyrrhalta maculicollis* (N = 18); Paen, *Pyrrhalta aenescens* (N = 23); Cbow, *Colaphellus bowringi* (N = 30); Pstr, *Phyllotreta striolata* (N = 36); Mcar, *Megacyllene caryae* (N = 34). Candidate *OcomORs* were indicated by red circles.

Thirty putative OR transcripts were identified in the *O*. *communa* antennal transcriptome. *OcomOR1* (*OcomORco*) gene was easily identified because it had an intact ORF and seven transmembrane domains, which were characteristic of typical insects ORs. The amino acid sequences of *OcomOR1* shared 91% identity with the co-receptor of *C*. *bowringi*. Except for *OcomORco*, 13 putative ORs were predicted to have a full-length ORF, encoding proteins with more than 335 amino acids. The putative *OcomORs* transcripts encoded complete proteins that were predicted to have three to seven transmembrane domains. Ten *OcomORs* were highly divergent and they had low levels of identity (<50%) with other reported insect ORs. Following the phylogenetic analysis, the OR sequences were clustered into several subgroups (Fig. 6). The *OcomOR1* was clustered with other insects ORco containing *PstrORco*, *CbowORco*, and *TcasORco*. In addition, *OcomOR5*, *OcomOR9*, *OcomOR24*, and *OcomOR26* were grouped into the same clade. Interestingly, *OcomOR2* and *OcomOR4* clustered with *McarOR3* and *McarOR5* in the same clade, *OcomOR12* and *OcomOR28* grouped together with *McarOR20*, meaning *OcomOR2*, *OcomOR4*, *OcomOR12*, and *OcomOR28* may play a role in pheromone identity function, because *McarOR3*, *McarOR5*, and *McarOR20* have been demonstrated to be tuned to the male-produced pheromone chemicals of *M*. *caryae*. Information, including unigene reference, length, and the best Blastx hit of all 30 ORs are listed in Table 3.

**Table 3:** The Blastx match of *O*. *communa* candidate odorant receptors.

Unigene ID	NAME	Length	ORF (aa)	Status	TMD	Blastx best-hit	Species	Score	E-value	Ident	Accession
Cluster-9750.33622	OcomOR1(ORco)	4800	479	Complete ORF	7	odorant receptor ORco	*Colaphellus bowringi*	902	0.0E + 00	91%	ALR72547.1
Cluster-9750.66916	OcomOR2	1594	446	Complete ORF	7	odorant receptor 5	*Pyrrhalta maculicollis*	591	0.0E + 00	74%	APC94229.1
Cluster-9750.12389	OcomOR3	1353	423	Complete ORF	3	odorant receptor 1	*Pyrrhalta aenescens*	537	0.0E + 00	75%	APC94305.1
Cluster-8368.1	OcomOR4	1665	415	5′ missing	6	odorant receptor 26	*Pyrrhalta aenescens*	585	0.0E + 00	65%	APC94330.1
Cluster-9750.24474	OcomOR5	1478	410	Complete ORF	6	odorant receptor OR27	*Colaphellus bowringi*	185	6.00E-50	28%	ALR72570.1
Cluster-9750.589	OcomOR6	1330	407	5′ missing	5	odorant receptor 4	*Pyrrhalta aenescens*	505	7.00E-175	64%	APC94309.1
Cluster-9254.0	OcomOR7	1399	406	5′ missing	7	odorant receptor 3	*Pyrrhalta maculicollis*	622	0.0E + 00	79%	APC94226.1
Cluster-9750.2139	OcomOR8	1392	400	Complete ORF	6	odorant receptor 10	*Pyrrhalta aenescens*	669	0.0E + 00	79%	APC94317.1
Cluster-18330.0	OcomOR9	1212	399	5′ 3′ missing	6	odorant receptor 2	*Pyrrhalta aenescens*	171	1.00E-45	30%	APC94306.1
Cluster-13422.1	OcomOR10	1305	393	5′ missing	6	odorant receptor 9	*Pyrrhalta aenescens*	368	2.00E-121	49%	APC94316.1
Cluster-11030.0	OcomOR11	1331	387	Complete ORF	7	odorant receptor 4-like	*Leptinotarsa decemlineata*	272	4.00E-84	38%	XP_023017287.1
Cluster-9750.5088	OcomOR12	1336	387	Complete ORF	7	odorant receptor 19	*Pyrrhalta aenescens*	538	0.0E + 00	72%	APC94313.1
Cluster-9750.1861	OcomOR13	1352	385	Complete ORF	7	odorant receptor OR15	*Colaphellus bowringi*	178	7.00E-48	29%	ALR72560.1
Cluster-9750.9485	OcomOR14	1245	384	5′ missing	6	odorant receptor 23	*Pyrrhalta aenescens*	556	0.0E + 00	68%	APC94324.1
Cluster-9750.31554	OcomOR15	1677	383	Complete ORF	5	odorant receptor 18	*Pyrrhalta aenescens*	543	0.0E + 00	85%	APC94311.1
Cluster-18259.1	OcomOR16	1384	382	Complete ORF	7	odorant receptor 14	*Pyrrhalta aenescens*	463	1.00E-158	64%	APC94327.1
Cluster-17525.0	OcomOR17	1303	380	5′ missing	4	odorant receptor 21	*Pyrrhalta aenescens*	501	8.00E-174	67%	APC94321.1
Cluster-17306.1	OcomOR18	1357	376	Complete ORF	6	odorant receptor 12	*Pyrrhalta maculicollis*	583	0.0E + 00	74%	APC94239.1
Cluster-7195.0	OcomOR19	1264	375	Complete ORF	6	odorant receptor 15	*Pyrrhalta aenescens*	431	5.00E-147	61%	APC94328.1
Cluster-13662.1	OcomOR20	1250	372	5′ missing	7	odorant receptor 11	*Pyrrhalta maculicollis*	469	1.00E-161	67%	APC94238.1
Cluster-18700.0	OcomOR21	1200	357	Complete ORF	6	odorant receptor OR1	*Colaphellus bowringi*	134	3.00E-32	29%	ALR72546.1
Cluster-8036.1	OcomOR22	1088	349	3′ missing	5	odorant receptor 18	*Pyrrhalta maculicollis*	657	0.0E + 00	87%	APC94230.1
Cluster-5881.0	OcomOR23	1277	339	5′ missing	4	odorant receptor 9	*Pyrrhalta maculicollis*	275	1.00E-85	46%	APC94236.1
Cluster-9750.52233	OcomOR24	1076	337	3′ missing	5	odorant receptor 2	*Pyrrhalta aenescens*	94.4	1.00E-17	26%	APC94306.1
Cluster-6485.1	OcomOR25	1132	335	Complete ORF	5	odorant receptor 13	*Pyrrhalta maculicollis*	589	0.0E + 00	79%	APC94240.1
Cluster-9750.24472	OcomOR26	1219	326	5′ 3′ missing	5	odorant receptor 4	*Pyrrhalta aenescens*	104	5.00E-21	27%	APC94309.1
Cluster-11404.0	OcomOR27	1396	315	5′ missing	5	odorant receptor Or1-like	*Anoplophora glabripennis*	376	1.00E-122	55%	XP_023310030.1
Cluster-1406.0	OcomOR28	956	268	5′ missing	4	odorant receptor 6	*Pyrrhalta maculicollis*	378	2.00E-77	71%	APC94231.1
Cluster-17313.0	OcomOR29	1032	212	5′ missing	3	odorant receptor	*Anoplophora chinensis*	184	2.00E-51	35%	AUF73043.1
Cluster-9750.12020	OcomOR30	484	120	5′ missing	1	odorant receptor 24	*Pyrrhalta aenescens*	93.6	2.00E-19	79%	APC94325.1

### GRs

**Figure 7:**
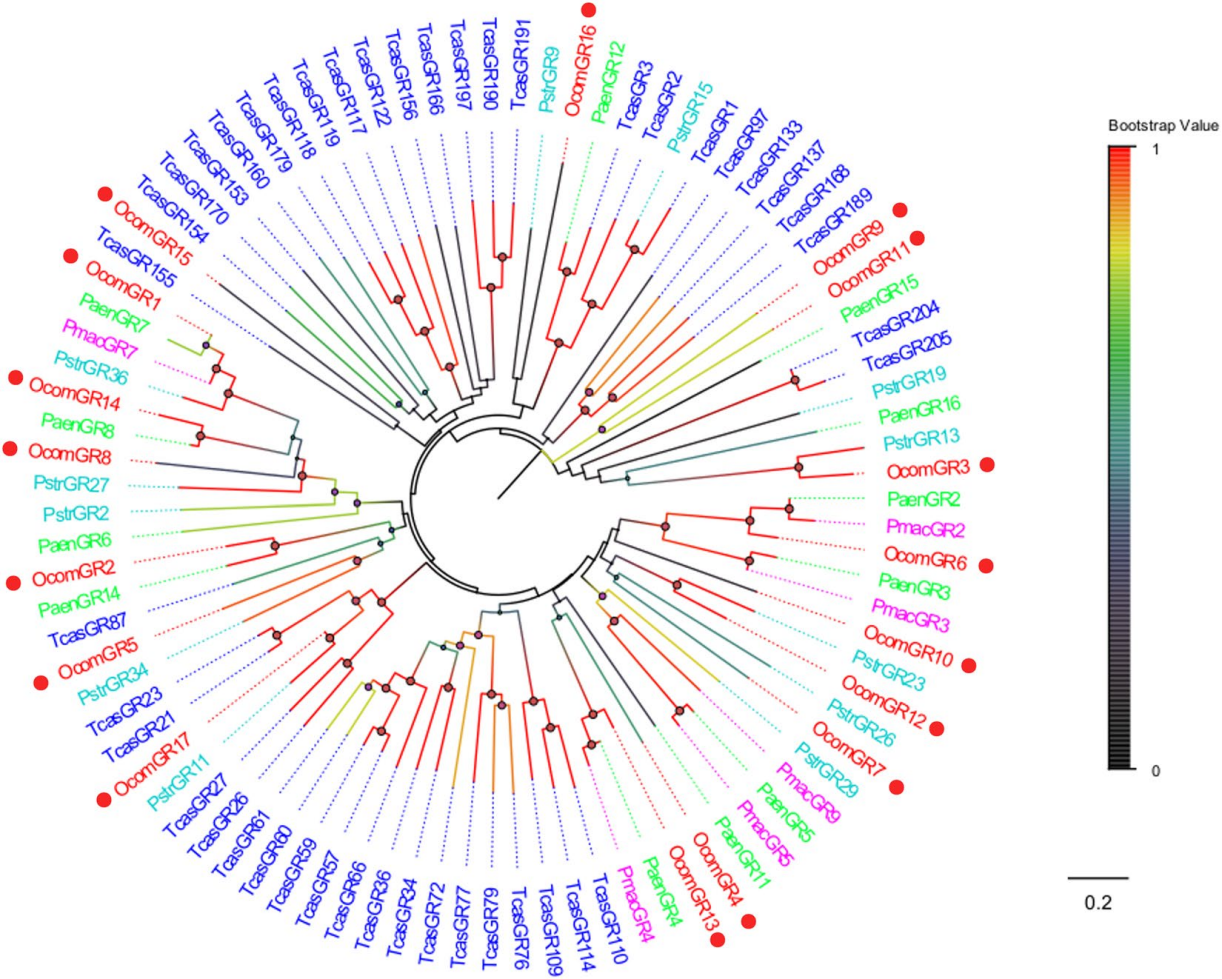
Neighbor joining phylogenetic tree of candidate *OcomGRs* with known Coleopteran GR sequences. Tcas, *Tribolium castaneum* (N = 44); Pmac, *Pyrrhalta maculicollis* (N = 6); Paen, *Pyrrhalta aenescens* (N = 12); Pstr, *Phyllotreta striolata* (N = 12). Candidate *OcomGRs* were indicated by red circles.

We found 17 candidate GRs transcripts in the *O*. *communa* antennal transcriptome (Fig. 7). The majority of candidate *OcomGRs* were partial fragments (only four were predicted to have a putative full-length ORF), encoding overlapping but distinct sequences. Eleven *OcomGRs* had more than 50% identity with GRs of *P*. *aenescens*, *P*. *striolata*, *C*. *bowringi*, *Monochamus alternatus*^39^, *Anomala corpulenta*^40^, and *Anoplophora glabripennis*^41^. Information, including unigene reference, length, and the best Blastx hit of all 17 GRs are listed in Supplementary Material S3.

### IRs

**Figure 8:**
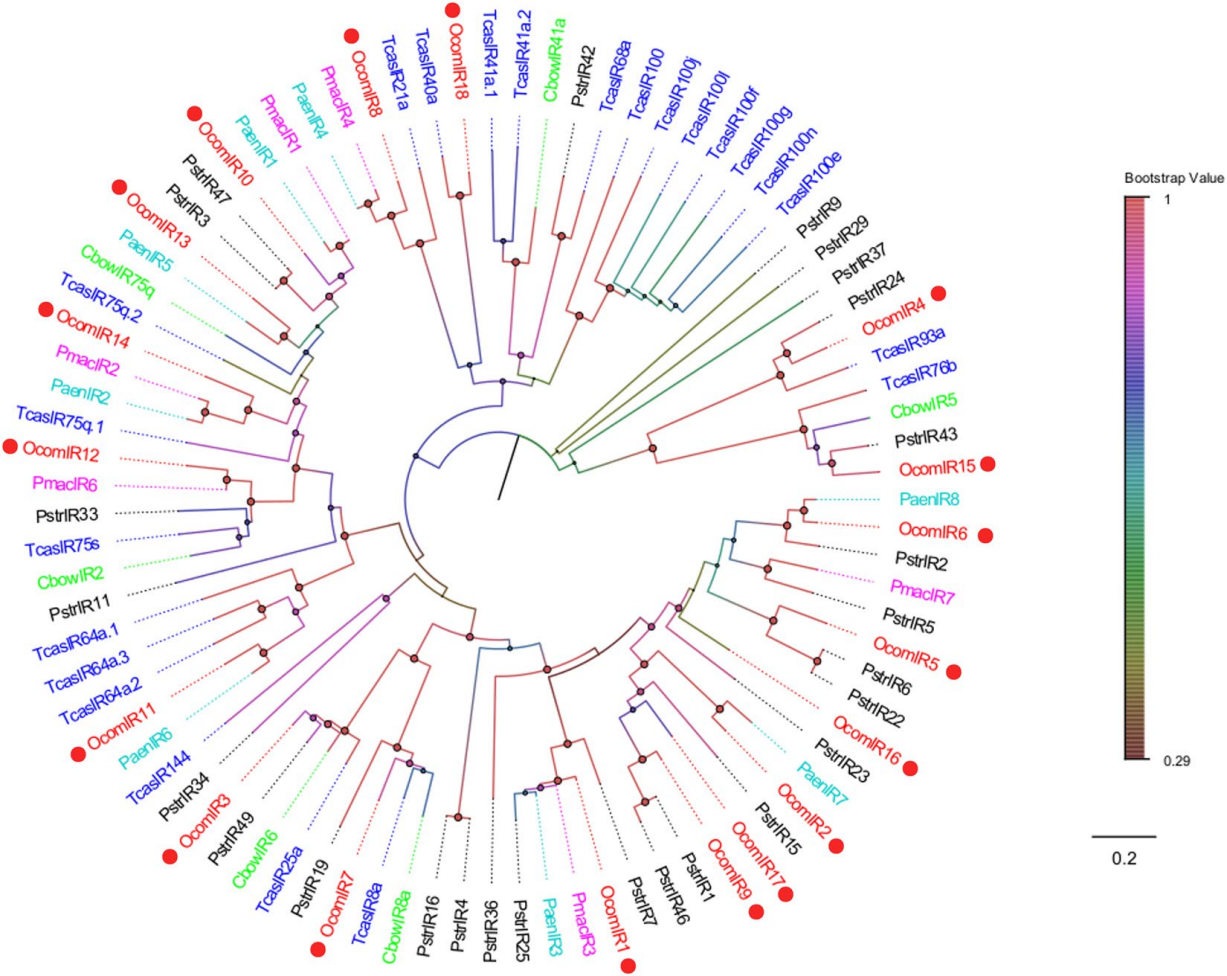
Neighbor joining phylogenetic tree of candidate *OcomIRs* with known Coleopteran IR sequences. Tcas, *Tribolium castaneum* (N = 23); Pmac, *Pyrrhalta maculicollis* (N = 6); Paen, *Pyrrhalta aenescens* (N = 8); Pstr, *Phyllotreta striolata* (N = 26); Cbow, *Colaphellus bowringi* (N = 6). Candidate *OcomIRs* were indicated by red circles.

We identified 18 transcripts encoding candidate IRs in the *O*. *communa* antennal transcriptome. Of these, eight *OcomIRs* contained a putative full-length ORF, with three to four TMDs. Based on the Blastx results, all *OcomIRs* had high levels of identity (>58%) with other reported insect IRs, indicating IRs were relatively conserved in Coleoptera insects. In the phylogenetic analysis, *OcomIRs* were grouped into different clades with high-level bootstrap values. *OcomIR3* and *OcomIR7* clustered with the IR8a/IR25a clades (including *TcasIR8a*, *TcasIR25a*, *CbowIR6*, *CbowIR8a*, *PstrIR19*, and *PstrIR49*), indicating they may be the co-receptor of *OcomIR*s (Fig. 8). Information, including unigene reference, length, and the best Blastx hit of all 18 IRs are listed in Supplementary Material S3.

### Fluorescence quantitative real-time PCR

**Figure 9:**
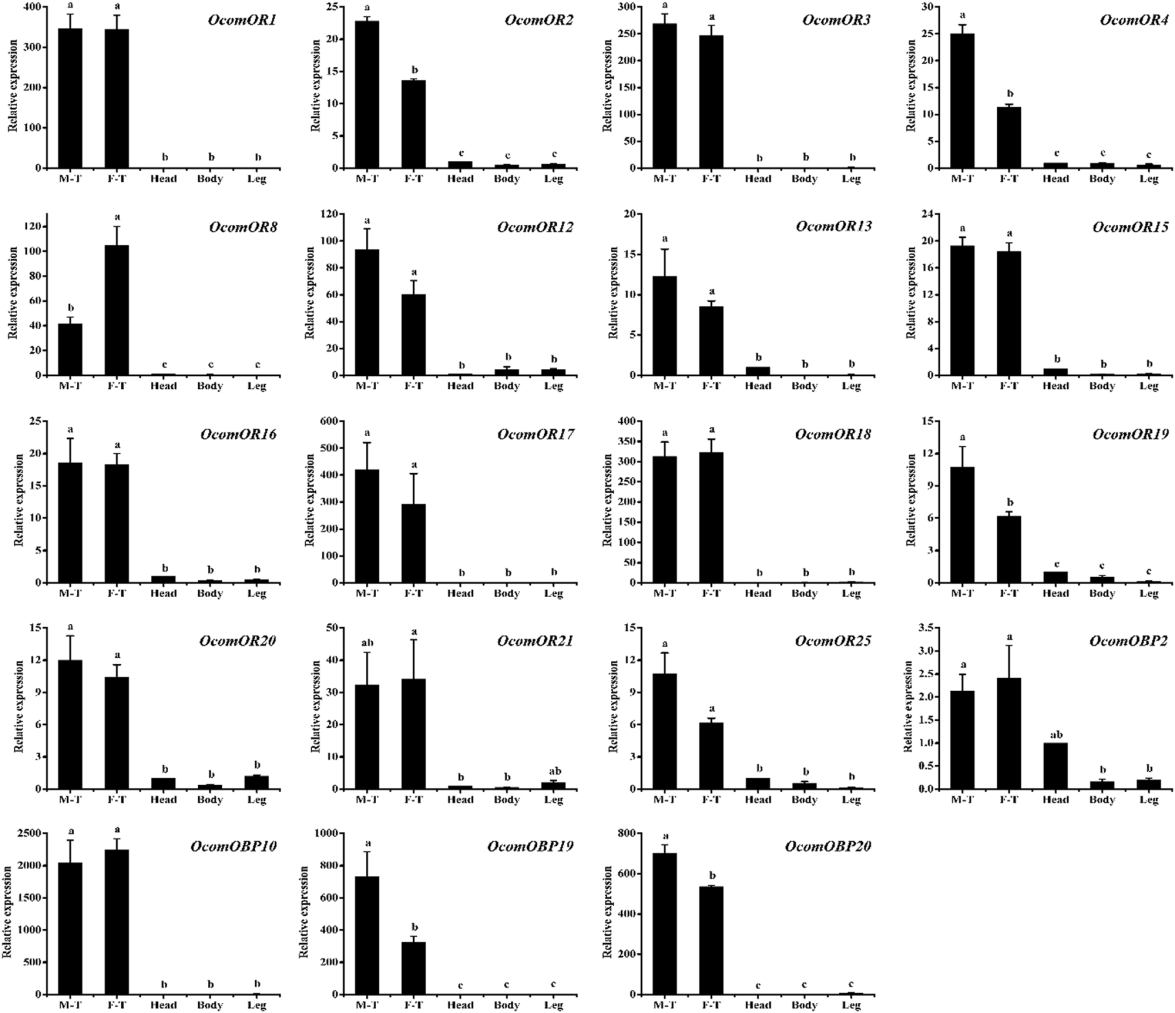
Relative expression levels of 15 ORs and four OBPs in adult antennae, head, leg and the rest of body using qPCR. M-T, male antennae; F-T, female antennae. The relative expression level is indicated as mean ± SE (N = 3). Different capital letters mean significant difference between tissues (*P* < 0.05, ANOVA, LSD).

To verify the expression of olfactory genes in male or female antennae and characterize the expression profiles of chemosensory genes in different parts (including male antennae, female antennae, heads, legs, and the remainder of the body), 15 ORs and four OBPs were selected for qPCR. The qPCR results showed that all 15 *OcomORs* were predominately expressed in the antennae, indicating their function related to insect olfaction. Although we did not find apparent sex-specific OR genes in *O*. *communa*, we found *OcomOR4*, *OcomOR19*, and *OcomOR2* had significantly higher expression levels in the male antennae, whereas *OcomOR8* had a significantly higher expression level in the female antennae. Furthermore, *OcomOBP19*, *OcomOBP10*, and *OcomOBP20* were specifically expressed in the antennae, whereas *OcomOBP2* was expressed not only in the antennae, but also slightly expressed in the head, body, and leg. Importantly, we found *OcomOBP19* and *OcomOBP20* were expressed significantly higher in male antennae than in female antennae (Fig. 9).

## Discussion

Compared with Dipterans and Lepidopterans, the molecular underpinnings of the olfactory system of Coleoptera are poorly understood. Based on the deep RNA sequencing, we analyzed the transcriptome antennae of *O*. *communa*. Among the 92,259 unigenes identified, only 47% gene translations shared significant similarity with entries in the NCBI non-redundant (nr) protein database, and only 36% unigenes could be annotated with one or more gene ontology (GO) term; this is similar to that reported in other Coleoptera species^42,43^. Thus, this amount of *O*. *communa* genes did not have any GO term because they were specific or fast-evolution genes of *O*. *communa*. However, the N50 of *O*. *communa* antennal transcriptome reached 2,068 bp, longer than those in *P*. *aenescens*^31^, *P*. *maculicollis*^31^, *C*. *bowringi*^30^, *L*. *decemlineata*^28^, and *A*. *quadriimpressum*^32^. The high quality of our transcriptome sequencing laid a foundation for olfactory annotation and further exploration of the molecular chemosensory mechanism of *O*. *communa*. On the basis of *O*. *communa* transcriptome results, we identified 105 candidate chemosensory genes, including 30 ORs, 25 OBPs, 11 CSPs, 18 IRs, 17 GRs, and four SNMPs, and this analysis substantially extended our knowledge of olfactory-related genes in Coleoptera insects. Moreover, we validated the expression profile of 15 ORs and four OBPs in different tissues of *O*. *communa* by qPCR, which facilitated the exploration of the function of these olfactory genes.

OBPs play an important role in odor processing by insects, facilitating the transport of odorant molecules through the sensillar lymph, and serving as the liaison between the external environment and ORs^1,44^. In our study, we identified 25 transcripts encoding OBP genes in the *O*. *communa* antennal transcriptome. The numbers of OBPs are clearly lower than *T*. *castaneum* (49 OBPs)^24^, *G*. *daurica* (29 OBPs)^32^, *P*. *aenescens* (31 OBPs)^30^, and *P*. *maculicollis* (36 OBPs)^30^, similar to the number of OBP genes in *C*. *bowringi* (26 OBPs)^30^, *L*. *decemlineata* (26 OBPs)^28^, but higher than that of *A*. *quadriimpressum* (16 OBPs)^32^. This is because the genome data of *T*. *castaneum* provide more a comprehensive list of olfactory genes than antennal transcriptome of other Coleoptera species. On the other hand, some genes may have been missed in our transcriptome results because some genes were expressed in other tissues than antennae^45^ or at different life history stages^46,47^. Relatively low coverage of the RNA-seq may also results in missing low transcripted olfactory genes. Based on the phylogenetic analysis, *OcomOBP19* grouped together with *HelePBP* and *AglaPBP1*, indicating that *OcomOBP19* may be involved in the pheromone recognition process. Further, the expression level of *OcomOBP19* in the male antennae was significantly higher than that in the female antennae, which confirms that the function of *OcomOBP19* may be related to pheromone identification in *O*. *communa*. Similarly, *OcomOBP20* expression in male antennae was significantly higher than that in the female antennae, indicating the function of *OcomOBP20* may be related to sex pheromone recognition or male-specific behaviors like *OcomOBP19*.

CSPs were another class of soluble proteins in the sensillum lymph with abundant expression^48^. A total of 11 candidate CSP genes were found in our transcriptome data. All of them were predicted to have a putative full-length ORF and amino acid sequences range from 118 to 261. In addition, the high level similarities found in Blastx best-hit results demonstrated that CSPs were highly conserved proteins between insects. Comparing CSP gene numbers in *O*. *communa* with that in other Coleoptera species, there was less than 20 CSPs in *T*. *castaneum*^24^, 15 CSPs in *L*. *decemlineata*^27^, and similar to CSP genes in *P*. *aenescens* (nine CSPs)^30^, *P*. *maculicollis* (ten CSPs)^30^ and *A*. *quadriimpressum* (ten CSPs)^31^. Thus, the number of CSP genes identified in our study was comparable with that of previous reports on these latter three beetles. SNMPs were first identified in pheromone-sensitive neurons of Lepidopterans^49^ and its function was thought to be related to pheromone detection^50^. Generally, SNMPs were classified into two families, SNMP1 and SNMP2. Two SNMPs were identified in *P*. *aenescens*^30^, *P*. *maculicollis*^30^ and *A*. *quadriimpressum*^31^, whereas there were three and four SNMPs identified in the *C*. *bowringi*^30^ and *L*. *decemlineata*^28^ transcriptome, respectively. In this study, four SNMPs were identified in the *O*. *communa* antennal transcriptome as well.

ORs are important to insects olfactory system, which determine the sensitivity and specificity of odorant reception, being the centerpiece of peripheral olfactory reception in insects^1^. The numbers of putative OR-encoding transcripts identified in *O*. *communa* are close to the number reported in the antennal transcriptome of *P*. *aenescens* (26 ORs)^31^, *P*. *maculicollis* (22 ORs)^31^, *C*. *bowringi* (43 ORs)^30^, *L*. *decemlineata* (37 ORs)^28^, and *A*. *quadriimpressum* (34 ORs)^32^, but much lower than the number in the *T*. *castaneum* genome (341 OR-encoding genes, including 79 pseudogenes)^24^, suggesting the antennal transcriptome data may have missed some OR genes. Obviously, *OcomOR1* was grouped with *PstrORco*, *CbowORco*, and *TcasOR1*, and formed a specific co-receptor lineage, indicating that *OcomOR1* could be the ORco of *O*. *communa*. Similar to the reported OR genes of *T*. *castaneum*, *M*. *caryae*, and *A*. *corpulenta*, a species-specific expansion of ORs (*OcomOR5/9/24/26*) was also found in *O*. *communa*, which may suggest that these distinct species inhabit different ecological niches. The OR gene function in beetles was first characterized in *M*. *caryae*^27^. *McarOR3*, *McarOR5*, and *McarOR20* were sensitive to three compounds of male-produced pheromones in *M*. *caryae*, indicating the function of these three ORs may be related to pheromone recognition^27^. In the phylogenetic analysis, *OcomOR2* and *OcomOR4* clustered with *McarOR3* and *McarOR5* in the same clade, and *OcomOR12* and *OcomOR28* grouped together with *McarOR20*, indicating that the function of these four ORs in *O*. *communa* may be pheromone identification similar to that of other lepidopteran pheromone receptors (PRs)^51^. In addition, qPCR results revealed that the expression level of *OcomOR2*, *OcomOR4*, and *OcomOR12* in male antennae was higher than in the female antennae, and the difference between *OcomOR2* and *OcomOR4* reached statistical significance. This evidence further demonstrates that *OcomOR2*, *OcomOR4*, and *OcomOR12* may play a role in pheromone identification in *O*. *communa*. *OcomOR8* expression levels in female antennae were significantly higher than in male antennae; therefore, *OcomOR8* may be related to female critical behaviors, such as oviposition cues or male-produced courtship pheromones. The sex-specific functions of these *OcomORs* need to be further investigated in the future.

Furthermore, 18 putative transcripts encoding IRs were identified in *O*. *communa*. The IRs number of *O*. *communa* was greater than in most Coleoptera species, such as nine IRs in *C*. *bowringi*^30^, ten IRs in *L*. *decemlineata*^28^, eight IRs in *P*. *aenescens*^31^, seven IRs in *P*. *maculicollis*^31^, three IRs in *Dendroctonus valens*^43^, and four IRs in *A*. *glabripennis*^41^. In addition, the IR number of *O*. *communa* was similar to that of 20 IRs in *A*. *quadriimpressum*^31^. Similar to the ORco, both IR8 and IR25 were considered to act as co-receptors because they were co-expressed along with other IRs. From the phylogenetic tree of IRs, IR8 and IR25 formed a conserved IR clade, which agreed with the analysis results of *C*. *bowringi*^30^. *OcomIR3* and *OcomIR7* were clustered into conserved IR25a/IR8a clades, indicating they belong to this co-expression group. Furthermore, IRs in insects were more conserved than ORs, we can predict that the function of IRs is probably conserved among Coleoptera. We identified 17 candidate GRs in *O*. *communa*. The GR numbers of *O*. *communa* was greater than most of those previously reported in beetles, and we also believe that there are more GRs expressed in other tissues, such as maxillary palps, proboscises, and legs. In the previous study of *P*. *aenescens* antennal transcriptome, *PaenGR12* was predicted to be the CO_2_ receptor^31^. In the phylogenetic tree of GRs, *OcomGR16* grouped together with *PaenGR12*, so we predicted that *OcomGR16* acted as the CO_2_ receptor in *O*. *communa*.

## Conclusion

Using next-generation sequencing technology, we first reported large-scale olfactory gene information for *O*. *communa* and identified 30 ORs, 25 OBPs, 11 CSPs, 18 IRs, 17 GRs, and four SNMPs. This large number of insect chemosensory genes will provide the molecular basis for the olfactory systems of *O*. *communa* and will advance our understanding of olfactory mechanisms in Coleoptera. In addition, homology analysis and qPCR were performed to confirm the tissue- and sex-specific patterns of these chemosensory genes, which can help us to predict their function. Further analysis is needed to explore the function of these genes using integrated functional studies.

## Materials and Methods

### Insects

*O*. *communa* adults were collected from Laibin City, Guangxi province, southern China in June 2017, mixed, and reared together with common ragweed plants in cages in an insect breeding room at 26 °C, under 14 h light: 10 h dark cycle, and 70%-80% humidity. After the beetles laid eggs, the adults were removed from the cages and the next generation reared on common ragweed plant in the same breeding room. After eclosion, the male and female adults were separated under microscope and kept in separate cages. The antennae of the unmated male and female individuals were collected two days after eclosion. The antennae were pulled off with tweezers by grasping at the very root of the antennae, and subsequently transferred to Eppendorf tubes. For the study of gene expression profiles in different tissues, male antennae (M-T), female antennae (F-T), heads, legs, and the rest of the body were collected. All samples were immediately frozen in liquid nitrogen and stored at −80 °C until RNA extraction.

### RNA extracting, cDNA library construction and Illumina sequencing

Total RNA was extracted using TRIzol reagent (Invitrogen, Carlsbad, CA, USA) following the manufacturer’s instructions, in which a DNaseI digestion step was included to avoid contamination of genomic DNA. RNA quality was checked with a spectrophotometer (NanoDropTM 1000, Thermo Fisher Scientific, USA) and 1% agarose gels, and its concentration was measured using Qubit® RNA Assay Kit with a Qubit® 2.0 Flurometer (Life Technologies, CA, USA). The complementary DNA (cDNA) library construction and Illumina sequencing methods followed Li *et al*.^30^. Briefly, The mRNA samples were purified and fragmented using TruSeq PE Cluster Kit v3-cBot-HS (Illumina) according to the manufacturer’s instructions. Random hexamer primers were used to synthesize the first-strand cDNA, followed by synthesis of the second-strand cDNA using buffer, dNTPs, RNase H, and DNA polymerase I, and then end repair and the ligation of adaptors were handled. The cDNA library created by amplifying the products using polymerase chain reaction (PCR) and quantifying precisely using the QIAquick PCR Purification Kit (Qiagen, Valencia, CA, USA). The cDNA library was sequenced on the HiSeq 2000TM platform.

### *de novo* assembly and gene annotation

The *de novo* assembly and gene annotation methods followed Li *et al*.^30^. All raw reads were processed to remove low-quality and adaptor sequences. And then the clean reads were assembled by Trinity v2.3.1^52,53^ using the default parameters to generate unigenes. The annotation of unigenes was performed by Blastx searches ((http://www.ncbi.nlm.nih.gov) against nr, Swiss-Prot, KEGG, and COG protein databases(E-value < 10^−5^). Blast2GO program^54^ was used to obtain the GO annotation and WEGO software^55^ was used to get GO functional classification of these unigenes.

### Sequence analysis and phylogenetic analysis

The ORF finder (http://www.ncbi.nlm.nih.gov/gorf/gorf.html) and the NCBI-BLAST network server (http://blast.ncbi.nlm.nih.gov/) were respectively used to identify the ORFs and perform the similarity searches of the candidate chemosensory genes. TMHMM Server Version 2.0 (http://www.cbs.dtu.dk/services/TMHMM) was used to predict the TMDs of ORs, GRs, and IRs. The signal peptides of OBPs, CSPs, and SNMPs protein sequences were predicted by Signal IP 4.1 (http://www.cbs.dtu.dk/services/SignalP/)^56^ with default parameters.

The amino acid sequence alignment of the candidate OBPs, CSPs, ORs, GRs, and SNMPs from *O*. *communa* and other insect species were performed using the ClustalW method^57^ implemented in the Mega v6.0 software package^58^. The OBP dataset contained 33 sequences from *P*. *maculicollis*, 31 from *P*. *aenescens*, 29 from *G*. *daurica*, and 47 from *T*. *castaneum*. The CSP dataset contained 10 sequences from *P*. *maculicollis*, nine from *P*. *aenescens*, 12 form *C*. *bowringi*, and 19 sequences from *T*. *castaneum*. The SNMP dataset contained two sequences from *P*. *maculicollis*, two from *P*. *aenescens*, four form *C*. *bowringi*, two from *L*. *decemlineata*, two from *P*. *striolata*, and five sequences from *T*. *castaneum*. The OR dataset contained 18 sequences from *P*. *maculicollis*, 23 from *P*. *aenescens*, 30 from *C*. *bowringi*, 36 from *P*. *striolata*, 34 from *M*. *caryae*, and 70 sequences from *T*. *castaneum*. The GR dataset contained six sequences from *P*. *maculicollis*, 12 from *P*. *aenescens*, 12 from *P*. *striolata*, and 44 sequences from *T*. *castaneum*. The IR dataset contained six sequences from *P*. *maculicollis*, eight from *P*. *aenescens*, 26 from *P*. *striolata*, six from *C*. *bowringi*, and 23 sequences from *T*. *castaneum*. All amino acid sequences of *O*. *communa* and other insects used in the phylogenetic analyses are listed in Supplementary Material S1. The phylogenetic tree was constructed using the neighbor-joining (NJ) method^59^ with P-distance modeling and pairwise deletion of gaps performed in the Mega v6.0 software package^58^ and the dendrograms were colored in Fig Tree v1.4.3 software package. The reliability of the tree structure and node support was assessed using a bootstrap procedure based on 1,000 replicates. To ensure greater accuracy in the analyses and make sure that the analyzed transcripts corresponded to individual genes, incomplete transcripts without sufficient overlap in alignments and protein sequence length less than 100 amino acids in length were excluded from the phylogenetic analyses. Six group candidate chemosensory genes were named “*OcomOBP*,” “*OcomCSP*,” “*OcomSNMP*,” “*OcomOR*,” “*OcomGR*,” and “*OcomIR*,” and were followed by a numeral in descending order of their coding region lengths.

### Quantitative real-time PCR validation

We selected 15 ORs and four OBPs to verify their expression profiles because their relative high abundance from fragments per kilobase of exon per million reads mapped (FPKM) data in antennal transcriptome. The expression profiles of 15 ORs and four OBPs were analyzed using qPCR experiments. Total RNA was isolated from the five tissues as described above. The concentration of each RNA sample was standardized to one ug/ul and the cDNA was synthesized using a first-strand cDNA synthesis kit (Transgen Biotech, Beijing, China) according to the manufacturer’s protocol. Ribosomal protein (RL4) was used as an internal control and its specific primer sequences were RL4-F: “TGTGGTAATGCTGTGGTAT” and RL4-R: “TCTAGCACTGCATGAACA”. The qPCR was performed on an ABI 7500 (Thermo Scientific, Waltham, MA, USA) with TransStar Tip Top Green qPCR Supermix (Transgen Biotech, Beijing, China). The PCR reaction programs were 30 s at 94 °C, 40 cycles of 94 °C for 5 s, and 60 °C for 34 s. All qPCR primers were designed using Primer Premier 5.0 (PREMIER Biosoft International) and the efficiency of these primers was validated before gene expression analysis. All primer sequences were listed in Supplementary Material S2. Each qPCR reaction was performed using three technical replicates and three biological replicates.

### Statistical analysis

Data analysis was performed using the 2^−ΔΔCT^ method and data were analyzed using SAS 9.0 (SAS Institute Inc., Cary, NC, USA). Statistical significance was assessed by an analysis of variance (ANOVA) followed by a Tukey multiple comparison tests. A value of *P* < 0.05 was considered statistically significant. Figures were made using OriginPro 9.1 (Northampton, Massachusetts, USA).

### Data deposition

All the Illumina sequencing data of the antennal transcriptome in this study have been stored in the NCBI SRA database, under the accession number of SRR8372148 (*O*.*communa* male antennae) and SRR8372149 (*O*.*communa* female antennae).

## Supplementary information

Supplementary Material S1

Supplementary Material S2

Supplementary Material S3
