# Supplementary material for: Identification of candidate chemosensory genes of Ophraella communa LeSage (Coleoptera: Chrysomelidae) based on antennal transcriptome analysis
Source: Sci Rep. 2019 Oct 29;9:15551. doi: 10.1038/s41598-019-52149-x (PMC6820725; doi:10.1038/s41598-019-52149-x)
Supplement: Supplementary file 1 — Supplementary Material S1 [file 41598_2019_52149_MOESM1_ESM.pdf]

Identification of candidate chemosensory genes of *Ophraella communa* LeSage (Coleoptera: Chrysomelidae) based on antennal transcriptome analysis

Chao Ma[1], Chenchen Zhao[1], Shaowei Cui[1, 2], Yan Zhang[1], Guangmei Chen[1], Hongsong Chen[1, 3], Fanghao Wan[1] and Zhongshi Zhou[1*]

Supplementary Material S1: Amino acid sequences of *O.communa* and other insects used in phylogenetic analyses.

```
>GdauOBP1
MYTLWMISVITMFAITVSASEFDDMRDRIMETKEYKECSQESGATYDDFMAFKNTTE
VMCLFKCSLEKKGSLDKDGNIDLDNIKERLSGNTHLDDTKKEMFLKCAESVGKIDKC
DDLLEFRWCLVNITKKQ
>GdauOBP2
MKYFVILSLFFVYVNAIDPKVVQAFMEKVTQFAGQCMAETKATDDDMAKLMAHEEPA
THEGKCMISCMYKKFGIQNDDGTMNPDEGMNMISKIKESDPDVYEKMVAVVNKCRDS
PVDEDHCITALDAAKCLIKEAKEMGLPPDMMGM
>GdauOBP3
MYKLTCFLLTVVVSSYPIKLPPEHQEFLDNLHRICIDKTGVTEADYAAYDMVNNPHD
PKLMCYMKCLMIEAKWMNPEGAIQYDYIIDTIHPDVKDIIVTALSKCRSIPEGENLC
QKASNLNFCLYGADPVNWYLV
>GdauOBP4
MIQDYFLFSLLVIASVILDGNCAPSDNSNVTVKDFCIKETGISLQKVKQMEDESIDE
LDEDCYCYLHCIF
ISLKLIDDKGKLDVAKTLEEFPDFNEECLKKVPQIMECTDMAAFDDCET
>GdauOBP5
MLIKMLKVLLLLGSLLLSVLALTEEQIALMNSLHSECVSKTGVAEDLVTNAKTGNLA
DDPKLKCYSACIFDELGVIDDNEKIDVDGVVAILPEEMQEFAPAVLKKCGTQSGADL
CDSIFNTFKCYYETDKRVFFLP
>GdauOBP6
MTEKQMNATKKLVRNSCTAKTKVAPDVVDAMHKGDFSNGQCYILCIMNTYKLIRADG
SFDWEGGIATVNANAPPHIAATAAASIKNCKDSMKNTSDKCLGSAEIAMCIYNDDPP
NYFFP
>GdauOBP7
MVKFVVYIVVAVLIACVCCTTLEYKPAKMAECLSEVGVTEDDIKEIPSNDKNKIACF
WKCIMEKTGVVSADGVVHPDKVEIAFPEVAPKLPAAVLNEFKTCLGTVGNINSCEDV
QKIRSCLKND
>GdauOBP8
MAKLLTLITIGVMCLSPSFAQKEDFIACLQESGLSDISEVKNLNTKDLSCFIKCTMT
```

KTGEIDSEGNVNMDKINLNLDKMKLTETIKDSLKTCFAGIEKIEKCEDAQKVTKCWS
GVFNDTKWFDEQ
>GdauOBP9
MKSFLVSCFLAVMLTYCAYGHMDPEKYGEIFGDIANRSHHFCKAITEVPQELIDGVR
EGQFPDVNSLKCYTTCLWVFAGSMNLDCTMNEKVLEKYMSGMNRNEDIEIMLECRKE
ACESDAKSFCEQGYELEKCIYEKVPNDSFIFY
>GdauOBP10
MSSMKYIFLSLCLFFIYENVQTLMTEKQIAATKKLIRNTCINKQGVAPEKVDGMYKG
EFDFSDKNSMCYVHCVLTTYKLIKKDNTFDWEEGISVMQLNAPPSIATPVIETIKNC
KNAVKTTNHKCTAALEIAKCLYDDDPVHYFLP
>GdauOBP11
MFYSLIIFILVCAATVLGAELEQTNYTNKCEIPASAPRKVEEFINQCQDDIKLAILS
EALETLNVNENHHSRAKRAAFTDDEKRIAGCLLQCVYRKMKAVNEYGFPTADGLVSL
YTSGIEHKEYIRTTIQSVNVCVKIAEKKYLVTPNSLDELGKTCDIAYDIFDCISDEI
GKYCGQTP
>GdauOBP12
MKYFIVLISAGIFGFSYALKCDIQKTNGDKIRNALSKCVKNNNTDSFWAMLSENEEN
SSEETQNNMEDTVNNSRSANSSERPKRASNDMLKNKTMQEDDTDNSTSKVADISENC
IIQCVLSNMDLVDSKGMPDQEKLVNEIVKTATTRELQTFLQESIDQCYQEMDKDNNL
DDCSSSMKLVKCLADKGRENCADWPAGGLPF
>GdauOBP13
MVVSYHLIFLVVYRKNIMMNLTAFLIVAFVCYANAEITAEQKEKLKEYHKACIVESG
VNPEVVKQSRQGQFAEDEKFKIHLFCISKKIGFQNDAGEIQTGVLSKKVGAILNDQK
LADQLISTCAQAKENAAETTFQTVKCFFEKSPEHISIL
>GdauOBP14
MKPILIFLCVTTLFAATLCKINFSDSYEENMDKIIEECQSNPATKATEDWLETEDLP
TADKEGPHILCRLTKIGSLDENGDINVEKMREDLKTGINDTKVIEVIMEKCGTREPN
TTATESAVANFRCVLSTYDDLNSQVD
>GdauOBP15
MNKPIIFAVLCIGVASCFVPETEFGIKLLKLGKEAHEKCIEETGVTQAAIERAKQGK
FDDDDIQIKDYNNCLWTFSKAINKNFEINVELIKELLPAKIRDVQLKAIMDCHEEIK
EGPILSLLEKTYLLSACVFNKNPENWIYF
>GdauOBP16
MVKGVLLFVVVCLIPSAFSLVQEDQYAPTLLAIYKDFLQRCKMYTGVTEDLIKQVRE
GNFPEDNSIKRYNDCLWTHHNYLIKPDRSIDERKAKYLLPKGSDPIVQVVVKCNSDN
AGEANDTEFFWKMHKCYHANIDSSMYYFL
>GdauOBP17
MKFVIIILCCIAAVSIDCLTDAYIARIKSLVDQCQANPTTRVPDEWLDLKVLEKNAE
QAGPHILCRLQKLGTIKENGDVDAAALRKDLLDAFEDPTKVDKAVTKCARREPNWSA
EKTAIENFKCVLNFN
>GdauOBP18
MIFPIIFASLCIIEAVKADVLSPEARTKIIENAKKCQKESEVDMKLLLKIKEGVFTD
DPKLKEHMLCVSKSFGIQQQNGEFNEAVMKVIFKKNGADDKKAEELSKKCLIKKDSP
ENSAFETMKCIHSIIPPAEIESLFENA

>GdauOBP19
MRNLTALLIVAIVCFANAELTEEQREKLREHNKECSAESGVNPDVIKLIKEGGEFPD
DEKLKTHVVCLAKKIGFMNDAGEVQPEVIREKIKAVLGDKALTEIMEACTGATEKDA
DTTFQKVQCFLQKAQKHFITS
>GdauOBP20
MFRELLIFVIFSTVSSNPLMEITDPKVKNLTETLHKVCVSKIGVQEASIEQGKKGIF
DRDPKLMEYWTCVWTTSGLMDQKGNIDFELLHSLAPSKVADATTKLVGACHNKVAGE
KVLTSLVLKMTQCIATTNSELFIIF
>GdauOBP21
MKTIFVLTCIVVVALGHGLPNLPENERLKLAQVHQACQSNPKTYCDENKLRNLLKNI
DDTEVGTHMLCMAVKAGLMKQNGDFNLDTMKNKIGLVIHDQSKVDGFVRKCSSKGEN
SGKSANLMWVCLVQNDVQYYHQL
>GdauOBP22
MYFHAFVFLFVAVISAEALSQLPGRFNKAHDKCQSDPASYVDEAILEKVRRGEKVTA
PNLAKHTLCMNVESGVQDQNGEIIVENLRKFLERAGNTKEKVDEAISKCGTRTSAIA
EEAAVALTNCVIKFRPERRHNR
>GdauOBP23
MNKSIIFAVLCIGVASCYLPESEYGIKLQKLAKESHDECIEQTGTTQAAIERVKQGK
FDDDIKIKEYNNCLWIFSKVLNENFELNSEILKEILPEKIKDVQLKALMDCQEEIKG
TAGADQSLVNKTYSLSECVCNKNPKTWIFF
>GdauOBP24
MKSVIMFIIICFSFVLEIFAETRTAPNLEYQEARRKCQDDPKTTLDRETLKNAFRGD
INTALFGGHAVCIYRHLGILDEQANINKIKLRDSLDYIIVDQRQLDAAVKECSVKKN
TPEETAVEIFKCTKRAMAPYLPHYEP
>GdauOBP25
MKTVFVFLCVLVAVLARGLPNLPENERIKLAEVHRSCQSNPKTFCDENKLKNLFNNI
NDPQVGTHMLCMAVKAGLMRPNGDFDIPYLKQKVGLVVRDHSKVDGLIQKCTHKAEN
SGKTANLMWKCLVENDVQYYHQL
>GdauOBP26
MHISVFALFFVLAVVSAEITHEQIEAFKKIHEKCQSDPETKIDDAIYDKIKRGEKVT
DPRLGKHTLCMNVGSGVQSQNGDINLDKLKQIVERASSNKERVDEIISKCGTRTSTN
AEEAAVGLAECLMKFRSGSLHGEHHGHH
>GdauOBP27
MVKEILLLVVVCFISSSFSAVPEHHFSPTLLSIYQDWQRRCRMYTGTTEELIKQTQE
GKFPEDESIKRYTDCLYTHHDYMVGPDNIVDTRKLKYLLPEGSEAILESVRKCNLDL
VNAGETNLTELLWNMHKCYHDNIDPSLYYFY
>GdauOBP28
MKTYVTFFIVVVALSDAIRNEEQLKEVTGFIRKCQEEYGLSNEFLDEIGSKHETDTQ
KAGQFSLCFTKKVGILKSTGEIDESKIKDFFRIFKAEDQMIQKILSKCKVPVHSTPE
ESALTFIQCLEGVYLK
>GdauOBP29
LTDEQIHKLDTYREDCAKLTGVDKELAIKARNGDFVEDEKLKEHLYCFSKKIGFQNE
AGDLQLDVIRQKLAQEIKDTKVLEDVVKKCAVKKDTPQETSFQVAKCYSEHKPVGD
>PmacOBP36

MKLLASTSILVILLISSSFGLIDYKRFGKKFTALIYRSHRLCKDTTGVSQQLIDGVK
VGQFPDNEPSIQSYTFCLWAATDGLFDDNYKFNKDVVNDYLSEINAIQDADKYLTCN
ENALKLDGTPTYKVWEMEKCIYKNVPTENFIYF
>PmacOBP35
MLKIVLMVAVCLAPTAYGAVPEHQFNPTLLSLVPDWHQKCVMYTGTTEDLIQQAKDG
KFPEDDSLKRYNNCIWTHHNFLVDDKRVVNLKKLLYLLPKGWESVGKTIKQCNTDVL
NTGENDDVEFYWKIMKCYHDKVDPSQYFFY
>PmacOBP34
MKTSQLMVISCFIISANCWLSPSGYSDTLKEKMHEWHAKCQRYSAVPEELIEEMKKG
KFPEVESAKRYTSCLWTLDGPFDRDYKMNSLKMLKYVHDNHSEDGHDYVRCNREALA
SGAKSFELFWTMQKCIQKRVDESHYIFF
>PmacOBP32
MKFFILFAVCVAAVNCIDDSEFSEKSRNFMKYLHDTCYAQTNVKDEYIEAHKKGNFY
VNDISLKKYISCLWTTSKALNANFDLDASLLYGVLPPRVAYYLVPIYMDCAKQVKFT
VPFDTPIEEKTFLVNICVYYRNPYEFVFF
>PmacOBP31
MFCGSIIFISVCAIIVLGAESKQTNYTNKCEIPASAPRKVEEFINQCQDEIKLAILS
EALQTLNANENAHSRAKRAAFTDDEKRIAGCLLQCVYRKMKAVNEHGFPTTEGLVSL
YTNGIEQKEYIRATIESVNTCMKMAEQKYLIKPDSIDENGKTCDIAYDVFDCISEEI
GKYCGQTL
>PmacOBP30
NCSQSFSKSKMMKQATSFLTSLLISSFLMVNGDKNDFKEALSKCQVDPESISKAFQS
GVVPTSFAPGIACTYKALGIVLPSGEINKDLFREKIKYFVDDVDEIERLTDICGVKK
STIEETSLHIFNCILDNLKP
>PmacOBP29
KALLKASSAPVTSKKPEYNTHITMMSFSIVLCVVLALSSALPRPDDASEKKMQESHE
ECLKESGIEEIDLRKVLETEAPEEALENYSYCFAKKFGFIGEDNKIDRDIAKQQLEA
MVPGSSEEFLKECIPEEEVTKKIALEITRCIDSKYKELKKD
>PmacOBP28
MKLFISFLIVAIVCCANAELTAEQKEKVKGYHKECIDVTGANPDLVKQAREGKFADD
EKLKNHILCVSKKIGFQNDAGDIQTDVLKHKLGAALGDQAVADQLISTCAQPKANPA
ETAFQTLKCYYEKTPTHVSFI
>PmacOBP27
MKSLLVCLLIITLAMTDETKDLIEEALISCGAEIEGPNNIGTLVVQENADETKLGTI
LFCANKKIGLQHDNGEINIDVLENLMETGNRDEATAKKILDCGRSKGDNGAAKAFNF
LKCHELAVRP
>PmacOBP26
EETPGLNNNSTSTMKTVLIISMLLAVSLAGKRREEIKEAVRSCGGDAGGSDDLRKHV
TDENVDQAALGAKIFCVNKKIGLQNDHGNIDESVFRKDLKAVVGDEAKVNEIVAECG
KRKGDTPEAAAFNFLKCYEKNVPK
>PmacOBP25
MKFVTSTSILLILLISSSFGLLDPKRFGKKFAALVYRAHRLCKDSTGVTQQLIDGVK
VGQFPDNEPSIQYYTFCLWGASGELDDNYKLIKDQLIEYLTEMDLLEDAGTYLSCNE
NATKLVGVPIHKQFWEMQKCLYKNIPTENFIFF

>PmacOBP24
MKILALSGILLILIIISSSLGLMDPKRFGKKFAELIYKCHRSCKVITGVPQQLIEGVK
VGQFPDNETDIQYYTLCLWASSGELNDDYTLKRDQLVEYLTEMNITHQADIYETCNK
NAIKLVGLPLYKQIWEMQKCIHKNIPTEDFIFY
>PmacOBP23
MKFILLAAVCIGGVSCIILESEFNEKLQKISKEAHRICVKETGITQEAIDHVKKGNF
YDDLKTKKYNNCLWRNTQVMGENFKLDEKLLMELVPEKARDLLFKGISECFEEARAA
PADLDFIEKTYSLSKCIHDKSPENWFYF
>PmacOBP22
MKTALIFILCLAAAHCIDLKEFGDEFVQKTKKLHDLCVAKINVTDSEIEQFKEGVYS
VQNKLFKEYIACLWLESKAVDENLNVHEDVVKNFLPPSKTGHLAPAYVACIKLVKKT
VPANTPVEEKFFLISNCVYFRNPEDWIFF
>PmacOBP20
MMVKSLNFCYVGLLWFINSVTGLSEEMQELANMLHTTCVEETGAIEDDILNARKGIF
ADKEEFKCYIKCLMAQMACIDDDGIIDVEATIAVLPEEYRDKAAPIVKKCGTKSGKT
PCENAWLTHQCYYKEAPDDYFLI
>PmacOBP19
TGRGVNCPLKMFKILVLGSLLSSVLAFTEEQIAMMKSLHSECVSQTGVAEDAISNAQ
AGNIADDPKLKCYMKCILEEIGVIGDDEKIDVDGALAILPEEMQEFAPAVMKKCGTQ
SGTDLCDAIFNTFKCYYAADKRVFFLP
>PmacOBP18
MNQNQFLIALVVLACFIFGGNCAPPDNANVTVKDYCIKETGVSGEKVKKMEDESLEE
LDEDCYCYLHCIFITLKLIDDKGNLDVTKTLKEFPDFDEECLKKVPKIMECTDIAAV
DDCES
>PmacOBP17
MATHTSNMKWLVVGLCLYLIITRDGWCAMSEKQMNATKKLIRNSCTAKSKVAPEIID
AMHKGDFSDGQCYLHCIMNTYKLIKPDGSFDWEGGITTVNANAPPNIAVTATASIKN
CKDALKNRSDKCAGAAEVAKCIYEDDPPNYFLP
>PmacOBP16
FFFFFFFMRFRQVIDKIEMCNIKYLLIIILLLAFTFIGITGLTEKQMKAAIKLVTNVC
QPKTKATAEDIEKMHKGDWDVDHTAMCYLHCAFNMYKLLNKDNTLNYDSAMQQIVQL
PESYRDSAKMCTEKCKNSAVTLTDKCVASYELSKCMYFCNPEKYFLP
>PmacOBP15
PGVSFIKLVCVSSNMYKLIYFAFTLLVSSYAIKLPPELQEYVDELHRICIAQTGITD
ADHAAYDIVKNPHDPKLMCYMKCLMIEAKWMNSDGVIQYDFITDTIHPDVKDIIVTA
INKCRSIPDGENLCQKASNFNFCLYGADPVNWYLI
>PmacOBP14
MKFVVIIALFFVYANAIDPKIVQEFVEQVTKFGEKCMEETKATTEDVAQLIAHQSPS
THEGKCMISCVYKAFGIQNEDGTINQEEGLKLASKIKESDPEVYEKMETISNKCQDI
PVDEDHCITALNLASCYIKEAKAIGLTAEMFGM
>PmacOBP13
TSLNMKGIKFVFCLIFLVEISTGISMFDQKWKQTTQVLLGDCRTKTGATKEDFETLR
LRKIPTTKTGLCLMECLFEGAGIMDNGTFDKEGTIVALTPSVNGDLSKIRKFRELAT
ACESELGHKRLQNCQTGKKILQCLATNGSKYGVQFINTKKMNV

>PmacOBP12
MFQSLFKFVFYLFLVGYFTSEAAAPKLNHPKIQDAVVKCSKDKGIDRKTLLEALKVD
QVDAKLKDYNLCILKGSGVVEPDGQINLVAAKEFSEMFFPDKVDIIMKTCCVMKENA
DETALWISKCLLNLKLI
>PmacOBP11
GDQSVLLIITISMKIIIILFIAFILVIGKAAKPPFDHPKAQEAITKCTKEHGIELKV
LLDTLKTDKEDVKIKDYNFCIYKNSDCIGDDGKLKTDVLRSYFKIFYPGKEDLVADT
CLKMKENSVETTYGISKCVTDLHLI
>PmacOBP10
IKKHLATMMSYSIVMCVILALTTALPSPNNSIKERMQESHEECLKENGIEEIVLSKV
LEAKVSEEALENYSYCYGKKYGYIGKDNKIDRDIAKQEFESMVPGASEEFMKTCIPD
GEVTKKIALEINRCIDRKWKELKKN
>PmacOBP9
FSKLFGFNRNLKYCSAMMFFVIFACLCILELCQAEVLAPEMRSKLLEHGEKCYKEAG
TDMEVLLKIKEGIFPDDPKLKKQLFCLNKSFGIQQENGEYNEEVMKTILKNNGADDK
KADELTKKCLVKKDEPEDSALEMTKCIYGIIPPEEIRSLYNKM
>PmacOBP8
MKVIVFCLMLSTFALANETKELIEGSLKSCGAEIDGPNAIRTLVVQENADEKKLGAI
LFCANKKIGLQYADGNINLDVLERLIEAGNNDEETAKKYMDCGRLKGETGEEKAFSF
LKCHETI
>PmacOBP7
AASNKENTSKFTMKTILVFSLLLAATLAGNRREEIKEAVTACGGENDGPNELGKLIL
NENADQAKLGAITFCVNKKLGVQNDQGDLDVDVIRKDLNTFIKDESEVNKIVEECGK
RKGDTSEAAAFNFFKCYQKHITK
>PmacOBP6
RELSDISIMKIFFIVSLLIAATLANEYIEKLETAAKACGLGGLGELRALEAKGEKTT
VGSTLFCINKEIGIQDSNGIVKAEEVANHIRKTHQDKDVSVQNKIIENCDKPTGDSG
VDKAYNLFLCIIKNLH
>PmacOBP5
MKILSLICFFAVFVSVYADSYFVFCAKYITYYIMCGNDAATSLNRDDLKNALKDKPF
NAKQLEGYLFCLNKHIGVQNEQGDYVPEQLKEFFKHVVVNQDNLEKIVHSCSTRPIG
SSAKDAALSLLNCAKKLDTLNFPFLNSTLQTDISSI
>PmacOBP4
MHCFHKYLRIFVDEIRIVIKARACIIFVLVQILKRFTMHFSVFVLFFVFAVASAEIT
PEQQAEQFKKIHDKCQSDPATHVDDAVFDKLRRGEKVTEPNLAKHTLCMNVGSGIQS
ENGDINIEKLRTILERGPANKAKVDEIIGKCGTRSSTNAEEAAEALANCVRQYRPER
PEGDHHGPHDHHHHH
>PmacOBP3
MKLWIVISLIIAGSVSIDCFSDAYLARIKTYVDQCQANPVTHVPEDWLDLTVLGRHA
DEAGPHILCRLKKVGSIKENGDIDVDALRKDLQEALVDTAKVEEAVNKCSKKESNWT
AEKTAIENYKCVLSIK
>PmacOBP2
MKTIFVLTCIVAVALAKNLPNLPENERVKLAQVHQSCQSDPKTYCDENKLRNLVNNI
DDPVVGTHMLCMAVKAGLIKSNGDFDLETMKNKISLVIDDQSKVDGLVKKCSHKTEN

SGKTANLMWVCFVQNDIQYYHSL
>PmacOBP1
MKTVFVFLCVLVAALAHGLPNLPENERIKLAQVHRSCQSNPKTFCDENKLKNLSNYI
NDIEAGTHMLCMAVKAGLMSPNGDFDIPSIKRKVALVTRDYSKVDGLVQKCTRKAEK
PGKTANLMWMCFFQNDIQYYHQL
>PaenOBP31
MKAAIILALCLAIAHCLVDVEELGEEFVEKAKHLHDVCVAQTQVPNSVINLYKAGIF
PVQNNLLKKYLTCLWLESKGANRNLEFNDEVIKNFFPASIAAHLVPIYVGCAKLVKQ
SAPMSTPIDDKFYLISNCIYFKDPENWVFF
>PaenOBP30
MRNTFSLFFLLSYFMLIQCDLEGEQVGENLKHEIPTWHQNCVILSDVTEEDIIALRK
GKFEDEDNEAVKKYFSCIILESKAMDANFKVNMTLVDYYLPKQISDDVFPTYLTCAK
EARELDASFEDKAWSFFKCFYDSDPEHFILP
>PaenOBP29
MIKFSILVTIAAICVCTSMAQKGSFVACLQESGLPEDFSKFGSPDSKEVNCFVKCMM
TKMGEIDEEGNVLMDKIEENLANIKIPDTAKDNLRKCLSAIDKIGKCEDSKKVTECW
TSLFK
>PaenOBP28
ENSVLKTIEYFSLFISIMKTFVIFFLAFAAVALAITDEERLNEIDDYIKQCQTKYGL
ADEFIEQIGTKHESDIKKAAQFSLCFTTSVGLQTETGDLVMSKIREIIKLFHLDENL
AEEVSQRCKPQQNIPA
>PaenOBP27
MKLELLIFTFCFISVKLQNPSGLAEIMAAHRICTSRTGISPALATGIISGRFPNDPI
LKQHLLCVNQQLGVQDQSGRIQTNRVSQLATNLSNSPSADISAIVSRCAVQKSTPQD
TAFDLVKCMWQARRSGRLQ
>PaenOBP26
MKILALICFLGIFLSVYGDSHFLFMAKYAKYYSDCDSEPATALNRDDFKNAMSGKPF
NAEQAAYFFCLNTKFGVQNEQGDYVREPLKKFFKHIVVNQDNIEKIVEICSTRPGGS
SAKDAALSLFNCAKKIDNLHFPFQHFTIEA
>PaenOBP25
MKLLASTSILVILLISSSFGLIDYKRFGKKFTALLYRSHRLCKDTTGVSQQLIDGVK
VGQFPDNEPSIQYYTFCLWGASGELDDNYKLIKDQLIEYLTEMDLLEDAGTYLSCNE
NASKLGELLVPLQIFAAPPNFK
>PaenOBP24
MKILALSGILLILIISSSLGLMDPKRFGKKFAELIYKCHRSCKVITGVPQQLIEGVK
VGQFPDNETDIQYYTLCLWASSGELNDDYTLKKDQLVEYLAEMNITHQADIYETCNK
NAIKLVGLSLYKQIWEMQKCIYKNIPTEDFIFY
>PaenOBP23
MKFVLLAVLCIGSISCFKESEHNEKFQKASKEVHRICMEETGITQEAIDNVKKGNFD
DDSKTKKYNNCLWTRIKVMNEKFELDEKLLKDLLPERIRDVQLKFIIECLEETRAAA
ADLESVEKTYLLTKCIYNKKPENWFYI
>PaenOBP22
MKTALIFTLCLAAAQCIDLKELGDEFVQKTKKLHDLCVAKINVTDSEIEQFKAGVYS
VQNKLFKEYVACLWLESKAVDENLNVNEEVVKNFLPPSKTVHLAPVYVACIKLVKKT

VPANAPVEEKFFLISNCVYFRNPEDWIFF
>PaenOBP21
MLRVMFLVFVIFSTTTSSPVVEMNDPKVKTIIATLHQLCVSQTGVQQSSIEEGKKGI
LDRDPKLMEYWTCVWTTSGLMDKKGNIDYDLFQALCPADIYDVMKKLLSTCHDKASD
EKVLTKLVLKMTQCVATTDFEHFVIF
>PaenOBP20
MMVKNLHFCYVGLLWFINSVTGLSEEMQELANMLHTTCIEETGAIEDDILNARKGIF
ADKEEFKCYIKCLMAQMACIDDDGIIDVEATIAVLPEEYRDKAAPIVTKCGTKSGKT
PCENAWLTHQCYYKEAPDDYFLI
>PaenOBP19
RTSRGVNCPLKMFKILVLGSLLSSVLAFTEEQIAMMNSLHSGCVSQTGVAEDAISNA
QAGNIADDPKLKCYMKCIFDEIGVIDDDEKIDVDGAIAILPEEMQEFAPAVMKKCGT
QSGTDLCDAIYNTFKCYYAADKRVFFLP
>PaenOBP18
MNQNQFLIALVVLASFIFGGNCAPPDNANVTVKDYCIKETGVSGEKVKKMEDESLEE
LDEDCYCYLHCIFITLKLIDDKGNLDVTKTLKEFPDFDEECLKKVPKIMECTDIAAL
DDCES
>PaenOBP17
KSCARIHTSNMKWLVVGLCLYLIITRDGWCAISEKQLNATKKLVRNSCTAKSKVAPE
IIDAMHQGDFSDGQCYLHCIMNTYKLIKPDGSFDWEGGITTVNANAPPKIAVTAAAS
IKNCKDSLKNKSDKCAGAAEVAKCIYEDDPPNYFLP
>PaenOBP16
YTAIDDFDSFFMRFRQVIDKIEMCNIKSLLIIILLLAYTFIGITGLSEKQMKAATKLV
TNVCQPKTKATAEDIEKMHKGDWDVDHTAMCYLHCAFNMYKLLNKDNTLNYESAMQQ
IGQLPESYRDSAKMCTEQCRNSAVTLTDKCVASYELSKCMYFCNPEKYFLP
>PaenOBP15
RGVSFIKLVCVSSNMYKLIYFAFTLLVSSYAIKLPPELQEYVDELHKICIAQTGITD
ADHAAYDIVKNPHDPKLMCYMKCLMIEAKWMNSDGVIQYDFITDTIHPDVKDIIVTA
INKCRSIPDGENLCQKASNFNFCLYGADPVNWYLI
>PaenOBP14
MKFVVIIALFFVYANAIDPKIVQEFVEQVTKFGEKCMGETKATTDDIAQLIAHQAPS
THEGKCMISCVYKAFGIQNEDGTINQEEGLKLASKIKESDPEVYEKMETISNKCQDI
PVDEDHCITALNLASCYIKEAKAIGLSAEMFGM
>PaenOBP13
KGMKFVLCLIFLVENSMEISIFDQKWKQTTQALLGNCRTKTGATKEDFETLRLRKIP
STKTGLCLMECLFEGAGIMDNGTFNKEGTIVALTPSLKGDLSQIQKFKELATACESE
LGYKRLQNCQTGKKILQCLAKNGSKYGLQFINTKNMNV
>PaenOBP12
MFKLLFKFVFYLFLVGYFTSEAAAPKLNHPKIQEAVVKCSKDKAIDRKTLLEALNVD
QVDAKLKDYNLCILKGSGVIESDGKINLAVAKEFSEMFFPDKVDIIMKTCCVVKENA
DETALWISKCLLNLKLI
>PaenOBP11
ISMKIIIILFIAFILVIGKASKLPFDHPKAQEAIRKCTKEHGIELKVLLDTLKTDKE
DVKIKDYNFCIYKNTDCIEDDGKLKTNVLRSYFKIFYPGKEDLVADTCLKMKENSVE

TTYRISKCVSDLHLI
>PaenOBP10
PGVLQLSRIKKHLTTMMSYSIVMCVVLALSTALPSPDDSSQKRMQESHEECLKENGI
EEIALSKVLEAKVSEEALENYSYCYGKKYGYIGKDNKIDRDIAKQVFEAMIPGSSEE
FMKTCIPDGEVTKKIALEINRCIDRKWKELKKN
>PaenOBP9
MMFFVIFAYLCLMELCQAEMLAPEMRSKLLEHGEKCYKEAGTDMEVLLKTKEGIFPD
DPKLKKQLFCMNKSFGIQQENGEYNEAVMKTILKNNGADDKKADELTKKCLVKKDDP
EDSALEMTKCIYSIIPPEEIRSLYNKM
>PaenOBP8
MKVIVFCLMLFTFALANETKELIEGSLKSCGAEIDGPNAIRTLVVQENADEKKLGAI
LFCANKKIGLQYADGNINLDVLERLIEAGNNDEETAKKYMDCGRLKGENGEEKAFNF
LKCHETI
>PaenOBP7
RVLTLIIPNTNKFTMKTILVFSLLLAVTLAGNRREEIKEAVTACGGENDGPNELGKQ
ILKENADQAKLGAITFCVHKKLGIQNDHGDIDVDVIRKDLNALIKDESEVNKIVEEC
GKRNGDTPEAAAFNFFKCYQKHVTK
>PaenOBP6
MKIFFIVSLLIAATLANEYIEKLETAAKECGIGGVGELRALEAKGEKTTIGPTLFCM
NKKIGIQDSNGIVHAEGVANHIRKTHAEKDESEQNKIIENCDKPTGDSGADKAYNLF
LCVIKNLH
>PaenOBP5
MKTLALVCFSAIVLPVYADSYFSFYANYITYYMLCDNDAATSLNRDDLRNALRDKPF
NAKQLEAYLFCLNKHFGVQNEQGDYVPEKLKEFFKHVVVNQDNLEKIVHSCSTRPEG
SSAKDAALSLLNCAKKFDTLNIPFLNETSES
>PaenOBP4
RGLVQILKRLTMHFSVFVLFFVFAVASAQINPEQRESFKKIHDKCQSDPATHVDDAV
FDKIRRGEKVTEPNLAKHTLCMNVGSGIQNENGDINIEKLRTLLANGPGNKEKVDEI
IGKCGTRSSTNAVEAAAEALSNCLRQYRPDRPEGDHHDHHDHDHHHHH
>PaenOBP3
MKLWIVILFTIAGSVSIDCFSDAYLARIKTFVDQCQANPVTRVPEDWLDLTVLGRHA
DEAGPHILCRLKKVGSIKENGDIDVEALRKDLQEALVDTSKVEEAVNKCSKKESNWT
AEKTAIENYKCVLSFK
>PaenOBP2
MKTIFVLTCIVAVALAKNLPNLPENERVKLAQVHQSCQSDPKTYCDESKLRNLVNNI
DDPVVGTHMMCMAVKAGLMKSNGDFDLGTMKNKISLVINDQSKVDGLVKKCSHKTDN
LGKTANLMWVCFVQNDIQYYHKL
>PaenOBP1
MKTVFVFLCVLVAALAHGLPNLPENERIKLAQVHRSCQSNPKTFCDENKLRNLSNYI
NDIEVGTHMLCMAVKAGLMTPNGDFDIPSIKRKVALVTRDHSNVDGLVQKCTRKAEK
PGKTANLMWMCFFQNDIQYYHQL
>TcasOBPC1
MKTIICFVFVLAGAWALTKEQIDKLEPISKECRELNGISEDTILKVRRGEAVNEPKL
KNHVLCVSKKTGLASETGETNVEVLRTKLRKVSENDDEVNSIIQKCVVKKSTPEETA

FEIFVCLRKVKPNFSPAN
>TcasOBPC2
MNFVCVIFILVAIIGAHGLSEQQTEKLNQLSKECRALTGVSQETITNARNGNFEEDP
KLKLQVLCIGKKVGIMNESSQIDENVLKAKLRKVSDNDEEVNKIYNKCAVKKPAPEE
TAFETIKCVMKNKPKFSPVE
>TcasOBPC3
MKFLVCLLFVIVAANALTKEQKEKLDKISKECKNQSGVSQELIDKARTGELINDPKL
KAQIYCVSKKAGLATEAGEINMDNLKTKLKKVAANDDEVNKIIQKCVVKKPTPEETA
FEVYKCLHANKPNFSVVD
>TcasOBPC4
MKAFIVLVAVAVCAQALTDEQKEKIKNYHKECSAVSGVSQDVITKARKGEFIEDPKF
KEHLFCFSKKAGFQNEAGDFQEEVIRKKLNAELNDLDATNKLIAKCAVKKDSPQQTA
FETIKCYYENTPTHVSLA
>TcasOBPC5
MKFLVVISTVLMANIVQGLTDEQKSKLEEYSKECLKESKVDESVLKEAEKGVYLDDP
KLMNHVYCLVKKINSQKDKGELEVTQIKEKLMMQINDEKEVDKLIQLCLVQEKSARY
SLGKCEVSS
>TcasOBPC6
MKYLLFLTVITLTCGIFAFSLSNREQAIFLSTYSTCLETSKVDSERALRTASGIIDD
EPKLKEFLFCINKQNGVQDDAGNFVKDAVRKRIEHPLLTDKTMEIIVNKCTRKRETG
EETAYQFLKCSYFTIMNEKHQ
>TcasOBPC7
MENRLVLLIVINTLLLAQAAAKQDFHKKCLASSGANADTIAKVRNGKFSNDPQTQKY
FGCMLRSVGVVNQAGQLQVAALRKQVPKDMKRDEAMKIYMSCKDKKGANNDETAYLL
YKCFWEASPRHVKIDGQ
>TcasOBPC8
MQKILFLGLFVSSSLSTRFFNHDEIQKLECFPDIGGGLKAELWPNVVSCVFHRKGFT
DDKGEFKIDVLKQKLSKFQDDKYLVNEIAELCVDKHDFTMTAAMKSALCLNKHAPWF
SPYQD
>TcasOBPC9
MRASAVFLSSFIISIQAAAFNNPEDELRRSAACLEQSKVSSESIKNLQIGNFDDDER
LKEYLFCVSKNAGYQDPAGHLQHEMIRLRFKGGRYSDDTINEVLQQCGHQKDTPQET
AFQFMKCAYQNAFPRNYK
>TcasOBPC10
MKLFILLSLLSVCYARKEWFDKDPQDVAKWQKECFEASGVSMESMNKLPNITLSEDP
KLGENAFCLLKKLGFISEDGTLLIEKLRTSLKNQWGDEIANKLVNECARQKSTPQET
AHEMFLCIPAKLK
>TcasOBPC11
MKIVLCLLALATVALAKKCFLAEDTDKLEVMINECKTKTGVPDDILQKARNGEKIDD
PKLREHALCMMKKSEMMNDAGEMQMDKIRARIKHAVSNEAEGTRIMNECAVKKDTPL
ATAYEMICCLIRNKNSVDE
>TcasOBPC12
MKVFVCLAVFALVAAAQAETAKEKLRKYSDECKSVSGVSEELLNKVRNHEDVHDPKL
DEHGFCILKKAGFMNEAGDILADTIKTKLKENSEHPDTVDALVEKCNEKKDTPQHTA

SHLFTCLVDKKVHSH
>TcasOBPC13
MKIVLICVLIGLVVAKQQKQDTLDEEKEKMKKWTQECIQESGVTSEILQQLRNQKRV
EDPKLKEYTFCTFKKNGFMNEDGKLQYDVIKSTLMKVSGSEEEANKVVKDCVVEKST
PQDTAFETVDCWYRYKKN
>TcasOBPC14
MNIYTCLVLVVIAASAQHLTEEQKNNWRKWSNECKVLIGVSQEAINKIRNNEFDSVD
DKIKKHGLCFAKKASLADSSGNIIINQIKIKLKRVIEDDEEVDRIVTKCTIRKNTPE
ETTFETFRCLRENSSKFVPV
>TcasOBPC15
MKMCVIFTLLLLVVLASAEEDNVGKIESVEKKCQEKTGVSEESLQKIMRLEEVDDPL
VKENALCTLKAYGVMDDDGNIFPDKFEEKLKPEIGADEAKRVAEKCAVKKDSPEETA
HQTLWCATEENALTDTSQEQ
>TcasOBPC16
MKISTLVAILVLAGSAVCADEDNLNTENVQSIEEDCQKETGVSDESLQELSETGDSD
DPLVKKNALCILKAYGVIDDQGEISEDKLEEKLEPDRGKEEAEKVAKSCAVKKDSPE
ETAHEALLCMQQKSQK
>TcasOBPC17
MKSFVIFVLIIVITGQINATPSLDDFKKVQKDCQKKTGVSDESINKVNNLEPVCDDL
LLQENALCILKTYEVMDEEGKICPDKLMEVLEPKFGKEKAEKLIEKCTLEKDTPQLL
AHATLFCLSVQKYVV
>TcasOBPC18
MNFFAICLCFVASTVGVSSEENDINEIRSVEENCQKQTGVSVEKVNNFELVDDPLVK
ENALCILKAYGIMDEDGNIYEDKLKEQITSELGEKNAEQVAKKCTIKKESPQETAHE
SLWCVGEQKPIPGASPDEKN
>TcasOBPC19
MSKTVFIFVIFFYLDFYATGDESVYLSNHEACVKLSGVDETLLETIYEGDVFEDMKF
KTYIHCFFKKSGFQDENGVMHFDAIKSSFHKDFSQTENIDKTITECEEKKLNGESAL
ETAFLHFKCFMGEL
>TcasOBPC20
MKKTILLCLLSQLLLLKAAELQPEDRHQIALQCIDIVGIDQKVVEDAINIEIPKNNP
KYKEFLACSYKKQGYQNENGEILMENIKKFLQKFYHPSDLQELNSCSGHNGTNHAEN
AYQALQCIYNRLSNMTVVGN
>TcasOBPC21
MKQIYCLITVVVLIPTLTALENEGQNPDTANCVALGGQRIKDSEIAKMAHCILTKTN
LMTDKGTFNSNLLKERLRQSVHSDELVDKVVMMCTVEKETPLKSAFSGYKCLRYLVP
WFPLD
>TcasOBP1
MILKASIFLILAVATFGAILEDSELMKVVENCVKKTNANESEFSSPNFLETTPSQPA
LCTAKCLLESLEIVNSEGNINMETLKEYAQPFESPAREAVATCGEEIKSVTTCDDME
KYRKCVEPLIKNS
>TcasOBP2
MNPITSVILTFLFVFSFGEKESEEALQQIFTELDGPAAELRDQCLEKNSMKVTDLKT
YNTSNDIPEKELCFYKCFYEGVEFIDANGNLNVNNMKEIPAISELGDEVLNEITACV

EKIGKIRCCGDLRKIEQCYQNITM
>TcasOBP3
MWSFVTLLFSFLVLASAQKKGKYWTTISECLTEHSMGVEDMKKFDLPAEKMSEEMLC
FNKCFYDKLLITDENGEINTDNLMSIPLVNAIDASKHDDLVTCLKKVGKIEECDGVK
KIEQCFVEFI
>TcasOBP4
MCRLFVVLSLFVASQALDVEKIRNELMADKNFVELRNKCLDKLGLKEEDLRDLKFDG
DVSEDLMCFGKCIQEEDGLLDSEGNLNEEKLEKKIETMPFLSRVSDDTKNNIMECLK
EIGKIETCQDFGKQRDCIHKYV
>TcasOBP5
MAKKQLVLFFLAFIFLQSSWAYFFMSQKFAEVREECLSENSMTMDELHEGWKMENLP
ESHLCFLKCLLEKREVIDENGVPQKEKIDEILTVKQLSDEKREEISTCITNVEKIEN
CETMSEIMRCFPKKRRD
>TcasOBP6
MSPLLLIFISCLFPRVFGISEEMQELANTLHATCVDETGVSEDAIESARKGNFAPDD
KLKCYMKCIMEQMACIDDEGIIDVEATIAVLPEEYQAKAEPIVRKCGTKIGANACDN
AFLTNKCWYEEDPEDYFLV
>TcasOBP7
MYKTRVIYVLFALCLVEIFAIEMDDDMKELINNLHNTCTGETGATDDQIENARKGNF
AEDDSFKCYFKCVFDQMGCMTDDGKVDSEAVIAVMPPELADKIASTVRGCTEVGANP
CETAWLANKCYQKSNPDMYFVP
>TcasOBP8
MIRYYIVLLLYFFAPPVLGISEEMQELVNQLHSTCVAETGVSEDLINKVNSDKVMID
DEKLKCYIKCLLTETGCISDDGVVDVEATIALLPEDMKAKTTPVIRSCGAKMGANPC
ESAWLTHKCYLETSPADYVLI
>TcasOBP9
MKAILLLLVATLSFYHVYCAMSEAQMKAALKLVRNVCQPKTKATNEQIEAMHTGNWD
LDKNGKCYMWCILNMYKLIGKDNSFDWEAGIATLKAQAPESVRDPAIASVNNCKDAV
KTTSDKCEAAYEIAHCMYLDNPEKYFLP
>TcasOBP10
MKTVAVLLFLALAACTKQEDDDRQETIRQYRDDCIAETKVDPALIDRADNGDFTDDA
KLQCFSKCFYQKAGFVSETGDLLFDVIKDKIPKEANREKALAIIDKCKELKGADSCE
TVYLVHKCYFLHSYGTDKKTE
>TcasOBP11
MSFLILLICVIPAIFCRSFSHDELDTDLSFIKTCNRTSPISMRTMNEVLINKKLGHG
ESSAFKCFLHCLFMKYGWMDSDGGFLLHDIKQTLEESDVEIASLEFILYKCTATESN
NRCERAFVFTQCFWDKMAEQQPSEDQFFYNIEDKK
>TcasOBP12
MKLLITLATLVVATYAIDKEFVQELRQKLRSHVEACAKEVNAGPDDVSAIFAHKLPA
THEGKCIFFCMHKLYNAQNEDGSLNMAGALANLELIKDMDPDVYTKVSTSFKNCESA
PFDSDPCLYAANLVTCIVKEGRAVGLDEVLVE
>TcasOBP13
MKFLLVFLSVAILCTFAMDESFLQQTRDRVKAIVKECVTEEKATDSDFDDIMALKIP
TSHEGKCVFFCSHKKFNMQHPDGSINKEGALDTFEVVKDVDAEFHDKVITVYNHCLS

TPVDPDPCVYSVNLFQCFMKEAKAAGIHELIIK
>TcasOBP14
MNSVLFLLVCALVACSGELDKEFLMQFLQKIKKVSEDCIAETQATKNDIKTLLEHKI
PDSHEGKCMIFCFHKHFQIQNEDGSLNKVAAISLLEPIKDHSQDIYDKVVKIFNTCF
DSAERDDDSCIYASNLAECAIRESKSLGLDDLLVIE
>TcasOBP15
MNCFVIFALSLSATVFGQSLSEDEMRENARKLMTSCKDKVGASDADVEALKMHQMPE
SREGFCMLECVFDSAKIMQDGKFSKSGMIEGFKPLIGDDKAKLESLEKLSATCESEL
GDGEDKCETAKRLVECVIKNGKTHGFEVPPPRE
>TcasOBP16
MQLLVVVLAVCVLGANAGLDPKFLEKLTQEVQAVGTSCGEKEHATADDMIEIMEEKF
PPTSHEAKCVVACFYKHYKMMKEDGTFDKDAAVKAFDEIKAQDAEIHAKILKVIDAC
DAKKQMSDDHCVSAASMAGCVKTEAIANGLTKEAFMAS
>TcasOBP17
MKSTWFFLLLACSLTCALDQEFVDEFLEKMQEFGAQCAEETDATSDDIAELIARKLP
PSTHEGKCMIFCMQKKFNMMKENGGIDRAGAIAALKPLQKADPELHQKVLKIFVTCG
MRVKPSPDPCDTATELALCGKKEAEAIGLEDALLT
>TcasOBP18
MKLFILAGILFTGVCAVDQEFVEKFLQKMEKIGEECAEETHATSDDIADLIEQRDPK
THEGKCLIFCYHKKFNTMKEDGSLDKVGSVLALEEVRDADFELYKNILTIFVTCGDK
AKIYDDPCETATALTMCGRDEAKALGLQDAIFG
>TcasOBP19
MKYFVVFASLFLATNALSQDFIDKFVAKVKSIGETCVPETNASKDDISSLLAHKMPD
SHEGKCLIFCFHKQFQIQNDDGSINREGAIKALEPLKADDAELYEKVISIFKKCEST
PVDGDSCLYAASLAECAVKEGRAMGLDNLIVLEIE
>TcasOBP20
MATRFCFGLLILFVGTVLVFAENEHEILEVRALCMNETGVSEETARNYKPAEDPASE
EILCMVKCIFEKIGCLKDDGSFCVDTMKKKNYIMDVINEENEEKIYECLRGVGKITN
CRDMAAVEECFVKNDSK
>TcasOBP21
MLRLVSLLCLFLLVQGENLDMFDPAGLQACMKKLSVGETELAKALEDKSKDPPEKIM
CLFKCALEDSGFLQDGVVDKSKWPMPECVQDVVKITNCNDMVALKHCFD
>TcasOBP22
MKPIFAIITLTLCTTVHALDCGIHINKNDALKATINKCLISNKTLEDLWDMAPMSSE
SDSSSEEVPPVDGKMLQNFRIKRASVRLTNTETNETTPEPKAVSSEAQATENCIIQC
IFDNLQMTDSTGYPVHTKILDGLLKNTTNRELRDFLQDTTDECFQVMDKEDTMDPCS
YSNKLVTCLAEKGRSNCADWPVGELPFKP
>TcasOBP23
MKYFPHLCLCLIFFELSEAAMSEAQLKAAVKLVRNMCQPKSKATNEDIEKMHHGDWN
IDRTAMCYMHCALNSNKLITKENVFNRDYAITLAEKNLPTALKTASIEAANLCKDSA
KTLDDKCVAAYEISKCLYESNPEKYFLP
>TcasOBP24
MSRMLPAALFVVLATLTFATAEIVVPDDLKDYINELHDHCLKEMGLTEGDHKNYNIH
VKDPKMMCYMKCLMTTSKWMNMDESIQYDFILSSVHPAVKNILLPALDKCRDIPKGT

MECEKAYNFNMCLFNADPENWFFI
>TcasOBP25
MPLKNLIILIVCPLFVFAKVDIPPDLQAEIDGYYDICYKQIGLTKDDLKAYKIGDRD
PKIMCFMKCVFVEAKWMDENENLQYDYIKNTIHHSIRHITLPELENCGKKAEGDKCE
KSFSFFNCMNKAEPEDWVLIQ
>TcasOBP26
MMHLKNFVVLVVCPLFVFAKVEIPPDLEAEIDEYFEQCFEPNGVTMDDIKAYKMGDK
DPKIMCFMRCLFVSGKWMDENENMQYDYIKETIHHAIRHITIPELENCGKEAQTGDK
CEKSFNFFMCMNRAEPEDWILDYKS
>AglaPBP1
MLIRLGAACGCVLLVLPLVLSISEELQELVDMLHNTCVGETGTSEEAIENAKKGDFA
DDEKFKCYLMCIMVQMACIDEDGIVDVEATIAVIPEEFQDLAAPIIRKCDTQKGSTP
CESAWLTHKCYYNENPDAYFLV
>AglaPBP2
MNLSVTLLYLLLCLTSIKGLSESKFIAAKAEARAACLASTGVSEDLVMDINRDGKFA
DDENLKCYVKCVHEYLGLMAEDGTMDYEKLIVNIPEEFRIKYASRIRACGTIYGSDV
CDTAWLTIKCYGENIPKLPHP
>HelePBP
MSEEMEELARRVRGDCVGQTGVDEALITTVKDQKGFPDDEKFKCYLKCLMTEMAVVG
DDGIIDVDAAVGILPDELKDVAEPVMRKCGVKMGANPCDNVYQTHKCYYDTDSKNYM
IV
>OcomOBP1
MKLFILLIIVGVFGTFEALKCELQKTNIGKIRDALSQCLPGNNTDAFWETSAESDED
STEEPENKTESTANKNKAATPANKTKRATKPESKNDTMQDTTSKDVTPETTENCIVQ
CVLKNMDLMDSNGMPDHEKILKEIVKSTTNREVHDFLQDSVDQCYQKNQDDKLDSCS
YSTNLIKCLADKARENCADWSADELPF
>OcomOBP2
MYTIPFFILVIGTSVVKLNAYKFDNPDFNQLLANDFEELYSNSHLVNIRNRRDASDE
TEARCPKRGPHLCCEDKSLRKLGDSKMEYKIECYKEVTGMDKPDKAPHDKAFDPFDC
EKMKQRKSNMLCISQCVGQKKNYIDADGKIKPEEFEKYIKDSLAEFKYLDPVVDKIV
AGCIADINNVTITDGKCQPDGLMVL
>OcomOBP3
MKTNLSGLIVLFLMSAAVVLSAERNETNYTNKCEIPASAPRKVEEFINQCQDEIKLA
ILSEALQTLNMNEDSHSRAKRAAFTDDEKRIAGCLLQCVYRKMKAVNEYGFPTIDGL
VSLYTSGIEQKEYIRATIESVNICLKLVEKKHLVKPDSVNENGKTCDVAYDVFDCVS
EEIGKYCGQTL
>OcomOBP4
MSPFLSITPEDVHTQVQYSISFGSRSRIPFFPLLYLFFVAFSVVTSSPLLEIKDETV
KSIQVALHDLCVAKTGVKAASIEDGKKGILDRDPKLMEYWTCVWTSSGLMDKKGDIE
YDLVGAMCPPNSKDAVMKSLKNCHDKASGEKVLSSLILKMTQCISTTDPEHFLMF
>OcomOBP5
MKQLKFVFCLLFLIEKSSEISPFESQWKQTSQVLLGNCRTKTGATKEDFEALRLRKI
PSTKSGQCLMECIFEGAGIMNNGKFNKDGMVLALTPTLKGDFSKIQKLKELGKVCEK
EIGYKRLDNCRTGKKVLEYLAKNGSKYDLQFVSKTKINV

>OcomOBP6
MLLKSFDKINMYTFKYFIYICIITYTITDVTGLSEKQMKAAVKLVTNACLPKSKAKV
EDVEKMHKGDWDVDHTAMCFMHCALSSYKLFNKNNTLNYESALQQISQLPSSYKDSA
KTCTDQCRDSAVTLNDKCIAAHEMAKCMYFCNPEKYFLP
>OcomOBP7
MKWFLLGICIYFIMEDVWCAVTEKQLNATKKLVRNSCTAKSKVAPEVIDAMHKGDFS
EGQCYIQCIMNTYKLIKPDGSFDWEGGVAAVNANLPSNLAVAAAASIKNCKDSLKNK
SDKCMGAAEVAMCIYNTDPPNYFLP
>OcomOBP8
MKKLLNFRKINMKLSEKCWTFFNFECQAGVMSAELRDQLIESGKKCYAEVGPDMELV
LKIKDGVFTDDLKLKKHLLCITKSFGVQQKNGEYNEAAMKAILKKNGADDKKAETVA
KKCLVKKDSPENSALELSKCIYNLIPAEEMKSLFEKM
>OcomOBP9
MKFFVTFALCFIYVNAIDQKVVDEFVEKATIFAQKCSEETKATSDDVAEIMAHKIPT
TQEGKCMISCVYKALGIQNADGTLNEEKGLELISVIKESDPEIHAKFESMIEVCKNT
PVDEDPCTTAVNFADCFVKESKKVGVSLEMLGIN
>OcomOBP10
MLVFCIVYQLNMHKLIYFISVLLVSSYAIKLPPELQEYVDDLHKICIAQTGITDADH
AAYDIVNNPHDPKLMCYMKCLMIEAKWMNPDGVIQYDYIIETIHPDVKDIIVTALNK
CRSIPDEADLCQKASNLNFCLYGADPVNWYLV
>OcomOBP11
MKKLILFAILCFGSVSCLILDEEFTETIKNKFNDIHNICVGKTGITQATIDRVKDGI
FDDDIILKKYNHCLWVETKAMNPNSIIEDDIFKEFVPEKMREEQLKSLKECTEELKF
KLLAPTEIMDKTFSLTKCIYNKKPENWFYF
>OcomOBP12
MKAVFIFITCIVAVNCFIDPKEFSIEFKEFTDKLHNDCVAKTNVPEFFIDQVKQGIF
VDFFPPIKIYTSCLWLDSTVTDDKLNVNVPVMAKLLPETVTPILTPLYNHCFQKIKE
TVPEEESLDNKLYWVSQCIYFGNPELWIFF
>OcomOBP13
MKFIIVLAICCSLFVIKVTTETRTAPNFKYQEARKKCQEDPKTTLDRETLKNAFRGE
INTATFGGHAICIYRHLGILDEHANVNKRNLRNSLDYIIVDQERLEAAVNECSVKKS
TPEETAVEIFKCTKRAMAPYLPHYEPQ
>OcomOBP14
MLFYVIVACLCLVKLSEAGVMSAELRDQLIESGKKCYAEVGPDMELVLKIKDGVFTD
DLKLKKHLLCITKSFGVQQKNGEYNEAAMKAILKKNGADDKKAETVAKKCLVKKDSP
ENSALELSKCIYNLIPAEEMKSLFEKM
>OcomOBP15
MKTVFVFLCALAVVLAHGLPNLPENERVKLAQVHRSCQSDPKTFCDENKLRNLSNNV
NDPQVGIHMFCMAVKAGLMKPNGDFDIPSMKNKVGLVVRDHSRVDGLVQKCTHKAEN
SGKTANLLFICFFQNDIQYYHQL
>OcomOBP16
MKSLAVILIVAIIYYANADLTPEQVEKVKGYHKECIAESGVNPDLVTKARQGTFVED
EKFKNHLFCVSKKIGFQNEAGDIQIDVLREKVGATLGDAALTEQLISTCAKTEANPA
ETTFQTTKCFYEKTPRHTTII

>OcomOBP17
MNFFVIVCFLVLALVSAQTPEQIQGIKKIHEKCQSDPATHVDDSVFEKLKNGEKVTD
PKWSKHMLCMNVGSGIQNENGDINVDKLRTSLEKAVDNKEKVDEIISKCGTRSSTNA
EEAAAALSACLASFRPSHHHH
>OcomOBP18
MKSLAVILTVAIICYAQAELTAQQIEKIKRFQKECIAESGVNPDLVTQARQGTFVED
EKFKNYLFCFAKRTGFQNEAGDIQMDFLREKVGAALGDQALTEQLISTCAKTKTNPA
ETTFQATKCFYEKNPKH
>OcomOBP19
MLKIVVLGSLLSIVVALTEEQIALMNSLHAECISQTGVAEDTIVNARAGNIADDPKL
KCYMKCIFDEVGVIGDDEKIDVDGAIAVLPEEMQSFAPAVMKKCGTQSGSDLCDAIY
NTFKCYYDTDKRVFFLP
>OcomOBP20
MKYLVLVCVFCALFSPRDCMTDALNEEILKIHEKCQPTSEVNDMVNRAMTGDFSDDP
AFKNHLLCIAKGMGLFTESGEVIMEKVESELDRSITNEALKSEIKSKCLQQLSTPQD
TVFNAVKCMFGYKDQV
>OcomOBP21
MNFSLLVFFLVFSLVSAITPEEIQKIHEKCQSDTATHVDDDVIDKVRRGEKITHPNW
PKYILCMNVASGFQHANGDINLNQIRTNLEKTLDDKAKVNEIISKCGTRSSTNAEEA
AVALFDCLHSFNI
>OcomOBP22
MVKYLILVTFTVMWVSTSNADREFIVQCLEEVGLADRISQLANLDLKEAQCFAKCMF
TKTGQIDADGNVNMDKVNEITERMKVGDSKKDNLKKCFADVKKIQKCEDTKTIIECM
DGVFKS
>OcomOBP23
MHRNHIVVTILLVVSFILEGYCAPPDSSNVTVKDYCVKETGITKEKVQQLEDESLEE
LDEDCYCYLHCIFVTLKLIDDKGSLDVKKTINVFPDFDEECLKKVPKIMECTDISAL
DDCEK
>OcomOBP24
MTKSDVNPVLLQKLKEGQFSDDVKLKNHIFCVSKKIGFQNNAGEIQTDVLNQKLGEF
LSDQALAEQLITACAPKLGNGAEGAFESIKCFYNEGSKLVSR
>OcomOBP25
MSPFLSIKPEDVHTQVQYSISFGSRSRIPFFPSSIDAALTPVFATHRSCKATCMDFT
VSSLISNRGLEVTTEKKEFLIVTQNLWNIGLVYGHLLV

>GdauCSP1
MKLSLVVCVILAVVVVSAKPEEKYTTKYDNINIDEIINNDRLLRSYVDCLLGTKPCT
KDGEELKKVFKEALDSKCSKCSDAQKEAAKKIGIYLIKNKRPWFDELVEVFDPDHTK
LEQYKDELKAEGIEL
>GdauCSP2
MNILRNARMLKRYVECLLDVPDTCTKDGDYLKQVLPEAIKTNCAECDAKQDDTAVRV
MAYLMKYHDDWWKKIDARYQASTSEFLLARKAKIEEYKNTL
>GdauCSP3

MNRWCISVVLMVLVVVAAVHCAPKSFDENLKVLKKIDINQVLNNDRIIRNYVDCVLG
KKRCTNEGNALKESWKDGLDKGCDDCDEEDKRKVKKILKHMYTKHRDLYDELAAHLD
KDGKYRDKYQAQIDEILKDPTL
>GdauCSP4
MGLIRLIFLFAVVSCSLAQTYNTRYDNIDIDRILGSKRLLDNYLQCLLDENVKRCSP
EGREFKRYIPEAISTNCAKCSDSQKRIVKKTAKYIITNRPQDWEKIKQRFDPQGKYH
QSFNDFLNSP
>GdauCSP5
MFSLVVVLCLAGLSSAAVTEKAKYTTKYDNVNLEEIVHSDRLLKSYVDCLLEKGKCT
PDGLELKKNMPDAIATDCSKCSEKQREGSEYMMRFLIDNKPDYWNPLQEKYDPSGAY
KQRYLESKKQEVKVEPITKT
>GdauCSP6
MNFVFVLCAFSLIAVVSAEENNEKYTTKYDNVDVDKILQSDRLLRNYIDCLLGKTQC
TKDGQELKNVLTDALKTKCEKCSEIQKKQAIKVITYLLKNKRSWWNEVEAVYDPTHN
YRQLYQKEIKEAGLEL
>GdauCSP7
MKTFLLIFIAATGSYVFAEKYTTKYDNVDIDTIIKSDRLLLNYVNCLLDRGKCTPDG
LELKKVLPDALLTDCSKCSETQKKGSKKIIRHLIDNKPNWYKELEEKYDKNGTYKIK
YEKEIRA
>GdauCSP8
MKLAIVVCVSLVIVAVAAAPADEKYTSKYDNINLDDIIKSDRLLRNYIDCLLGTKKC
TKDAEELKRVLPDALKTKCAKCTEAQKNGAKKILRHLLKNKREWFNELEAVYDPEHV
YVKSYEKELKEEGIEL
>GdauCSP9
MNFFCVSILIGFLMVVAVSGAPKTFEENVATLKKVDLKAVLQNTRIMRAYVDCVVHN
THCTPESTALKESWKEGLDQGCADPCDEEAKKRVQIIAKYVYTEQPEWYKEIIEALD
KDKKI
>GdauCSP10
MVPLICILVALSGLVVSAPVPEQKEQYYTTKYDHVDIEMILSNRRLIYYYTACMLNK
GPCSPEGLEFKRLIPDAIQTNCKRCTEKQKVGTVRAIKGLMKEYPKVWDQLKAEWDP
DDIYVEKFLATHGNFPNINMISNRFDADEPSEPTQTSVSNSTESSSNDSSTTPNASS
KPSSTTPRQSSTTSSTTLGIYYPPSLDPGTIPIANTIGQGIKATVSLGNNIVRKVIK
DIETIGNTVVLTGAKIAENIGNSVIQTRNRIATVLREATRPQRKV
>PaenCSP1
MKFVLVFVVLGVIAVTARPEDDKYTTKYDNINIDELIQNDRLLRNYVDCLLGTKPCN
KDGEELKKVLKDGLRTKCAKCNDTQKEAAKKLAIHLIKNKKPWFDELTPVYDPDHTI
VELYREDLKAEGIEL
>PaenCSP2
GSLLQNRVLTAVSKMKLAIVFGVLLVAIVAVNAKPADDKKYTSKYDNVNLDDIIKSD
RLLRNYIDCLLNIKTCTKDGEELKKVLPEALKSKCEKCTDVQKDGAKKIIRHLLKNK
RQWWDELEAVYDPEHVYIKSYEQELKAEGIAL
>PaenCSP3
MVPLICAIVVGLSGLVVPAPVLEDGEQYYTTKYDHVDIEMILNNRRLVSYYAACMLN
KGPCPPEGLEFKRILPDAIQTNCVKCTEKQKATTLRTIKRLMKEYKKIWDQLKAEWD

PDDVFVKKFLETYNKPTSTVSSIFSNRFGDEEFSEQTQAPLNKPKEPMKSSTTTTTT
TSTTSTTSATLGVFLPPTATRAPAPNAVNPLANSLGQTIKATVSLGNNIARQVIKDI
ETIGNTVISTGVEIGNRVIQTGTQIAGALRGIPRPVNRFNN
>PaenCSP4
PGVILSNRSRYRDLLIELKMKAILAFLFLWYLCGISDCDKYTTKFDNVNLDEILKSD
RLLKNYVDCVMDRGKCTPDGKELKDHIVDALETDCEKCSEKQRNGSVQVIKYLVKNK
RHMFDELSEKYDPKGSYRNRHKEELAKEGIVL
>PaenCSP5
GELFLKKFQSVCIRFYVMFSLVVTLCLAGVSFATVTEKSKYTTKYDNVNLDEIVHNE
RLLKNYVDCLLEKGKCTPDGLELKKNMPDAIASDCSKCSEKQKEGSEYMMRFLIDNK
PDYWNPLQEKYDPSGAYKRRYLESKKQEVKVEPITKS
>PaenCSP6
PTLILQVIAEMYKYSLFIEGKLLNYCKMKTFAVIFIVIIGTAVCAEKYTTKYDNVDI
DTIIKSDRLLLNYVNCLLERGKCTPDGLELKKVLPDALLTDCSKCSEAQKKGSKKII
RHLIDNKPDWYKELEEKYDKNGTYKKKYEKEIKS
>PaenCSP7
GRYSPTYRSPEPMSRRCMPNPSSRILEDASIMCNSWSPRHNGDIFSGLNDGLISRAE
ALAAVDIKHQGTGGHGGLPQLKHDMMYHHSMGAPPPVSRP
>PaenCSP8
VVVAVLGTPKTFEENMAVLKKIDINAIIKNDRLIRSHIDCVEFNKRCTPEGLALKES
WKESFEKGCVDCDEQTKKNVKKLIKHIYVNHHDWYEELINALDKDKKYSTKYQSYID
DLLKDDSI
>PaenCSP9
MNRWCMLFIVLVVVAAVLGAPKKSFEENLKVLRKIDINTVLNNGRIIKSYVDCVTGK
KRCTAEGTALKESWKEGLDKECDACEEEEKKKIKKIAKHIYTHYRPLYDELAAALDK
DGKLQERYKAYIDEILNDPTI
>PmacCSP1
MKFVLVFVVLGVIAVTARPEDEKYTTKYDNINIDEIINNDRLLRNYVDCLLGTKSCN
KDGEELKKVLKDGLRTKCAKCNDTQKEAAKKLAIHLIKNKKPWFDELTPVYDPDHTI
VELYREDLKAEGIEL
>PmacCSP2
NAKPADGGSLLQNPVLTAISKMKSAIVFGVLLVAIVAVNAKPADGEKYTSKYDNVNL
DDIIKSDRLLRNYIDCLLSIKTCTKDGEELKKVLPEALKSKCEKCTDVQKNGAKKII
RHLLKNKRQWWDELEAVYDPEHVYVKSYEQELKAEGIVL
>PmacCSP3
MVPLICAIVVGLSGLVVPAPVLEDGEQYYTTKYDHVDIEMILNNRRLVNYYAACMLN
KGPCPPEGLEFKRILPDAIQTNCIKCTEKQKATTLRAIKRLMKEYKKIWDQLKAEWD
PDDVFVKKFLETYNKPTSTVSNIFSNRFDDEEVSEQTQTPLNKPKEPMKSSTAPSTS
TTSTTSTTSATLGVFSPPKTTRAPAPNAVNPLANTLGQTIKATVSFGNNIARQVIKD
IETIGNTVISTGVEIGNRVIQTGTQIAGALTGIPRPVNRFNN
>PmacCSP4
MKAILAFLFLWYLCGISDCDKYTTKFDNVNLDEILKSDRLLKNYVDCVMDRGKCTPD
GKELKDHIGDALETDCEKCSEKQRNGSVQVIKYLVKNKRPMFDELSEKYDPKGNYRN
RHKEELAKEGIVL

>PmacCSP5
MFSLVVTLCLAGVSFATVTEKSKYTTKYDNVNLDEIVHNERLLKNYVDCLLEKGKCT
PDGLELKKNMPDAIASDCSKCSEKQKEGSEYMMRFLIDNKPDYWNPLQEKYDPSGAY
KRRYLESKKQEVKVEPITKS
>PmacCSP6
VIAEMYKYSLFIEGKLLNYCKMKSFAVIFIVIIGTAISAEKYTTKYDNVDIDTIIKS
DRLLLNYVNCLLDRGKCTPDGLELKKVLPDALLTDCSKCSEAQKKGSKKIIRHLIDN
KPDWYKELEEKYDKNGTYKKKYEKEIKS
>PmacCSP7
PDKMMGSKGPTGLHGPITSTGACFAGRYSPTYRSPEPMSRRCMPNPSSRILEDASIM
CNSWSPRHNGDIFSGLNDGLISRAEALAAVDIKHQGTGGHGGLPQLKHDMMYHHSMG
APPPVSRPHHQMGHMDGLEMLDPISSSSMTTLTPMSETPTHMHSYGMNHVMNHHHHG
GPLSGHSAPGHHPGHHGGHPG
>PmacCSP8
KYDNINIDEIIQNDRLMRNYVDCLLGTKPCSKDGEELKKVFKEGVDSKCAKCNDKQK
EAAKKIAVHLIKNKRQWFDELSAVFDPDHSKIEEYREDLKAEGI
>PmacCSP9
MNKSVLFVFLLAIGYSAAAYSHRYDYIKVMDVLHNTRMLKRYIECVLDVPDTCTKDG
DYLKEILPEAIKTNCAQCDAKQLDTALRVIGYLMKHHPDWWQKVDSKYGASGSQFLA
NNKVKIEEYTNTL
>PmacCSP10
KYDSKFDSIDYKSILASERLLQNYFKCLMDEGPCTADGRELKKIIPDALKTDCSKCS
EKQKAAGKEIIHFFIDQKPDMWKKLVEKYDPEGVYIEKYV
>CbowCSP1
MKYFVIILSVLIIAAAVGEKYTTKYDNVDIDSILNSERLIKNYMDCLMERGPCTPEG
KELRDNLPDALKTECHKCSDKQKEVSKKVLRHLVKNKRKEFDELTGKYDPEGVYKNK
YKEDLAKEGIIV
>CbowCSP2
MSRLLFSCLLLGVVVFVLADEKKSRANVPKYTTKYDNVDLDAIINNDRIFRNYIECC
LGKRKCTPDGLELRNHIRDAMDNECDKCSETQKKAMKKVGRKLYKEKPEWWKELCDH
FDPDMKYRTKYQKFIDEALAEEDK
>CbowCSP3
MKFSLMLCVLVALLVFADARPEDKYTTKYDKVDLDAILQNERLLRSYIDCLLDKKKC
SKDGEELKKILPEALKSKCAKCNENQKKGAKKVIRYLLKEKRAWWDELEAVYDPEGI
YRKTYEKELKEEGIQI
>CbowCSP4
MNIPLGICFLMMVIFVRAGEKYTAKFDNIDYEEILRSERLLKNYIFCLLDKGPCSPD
GLGIKNILADALETECSKCSDRQKEGSTKVIRFLIENHAGWWKELTEKYDPDGIFMQ
KYRDQWNSNN
>CbowCSP5
MKASALFFVAFFAVVATKPAEKYTTKYDNVDLDTILKSDRLLKNYVNCLLDKGNCTP
DGAELKKVLPDALQTDCSKCSDVQKRGSKKIIRYLIDNKAEWYKELEAKYDKNGVYK
KKYDKELEDAKV
>CbowCSP6

MGLSKVLFCFFVIVTVAFCQTYNTKYDHVDIDSILANKRVLSSYIKCIMDEGPCTSE
GREFRKHIPEAITNNCAKCSDAQKRIIRKTSRFIRRERPQDWDRVSRKYDPQQKYTA
NFNKFLSEN
>CbowCSP7
MKIVAALLFISLLAFVTAQNSYSRKYDNVDVDKILKNERVLSNYIKCLLEEGPCTAE
GRELKKTLPDALANECEKCNPNQKNTAEKVMKHLMSKRARDWERLSKKYDPQGNYKK
RYQHLVEKVAN
>CbowCSP8
MMNMRTFLVFTFVCVATKAQILSRNNYIQKQLLCTLDRAPCDHLGSQIRDALPEIIG
NNCKSCDQRQTANAKRIAVFVQSKYPDVWNALVKKYSRLE
>CbowCSP9
MEVSYHLIVCIVVFSYVSEDRFVSTTSLNRVERAVEKYSNKYDKFDVAGVLASARLV
KRYGDCLMDRGPCPPEGRFLKDIVPDAIATECSKCNNIQKKQAGLILQHLLLHYRPL
FLELCDKYDPTGKARKQYGIDTNEADEYEDYDEA
>CbowCSP10
MRTIILLAVLGVCVSAYNTAFDRVNVEDVLKNKRLLKRYVDCLLGVPKTCTKDGQLL
KDTLPNALKTKCEDCSEPQRKGAKRVANYLIDCKPKWWSDLAKIYDSDGIYTKQYHD
ELLAEGINIDGSSKDTEHKTQCYN
>CbowCSP11
MQIKYQDAILVTVVLCAIVTIVNALPQSQAISDEALESTLKDKRYLLRQLKCALGEA
PCDPVGRRLKSLAPLVIQGSCSQCTPQEQRQVRKVLSYMQVNFPKEWNKVLKQYSG
>CbowCSP12
MKEGKARKLSQCYSFHGHEQCKYISLKMFLPFVVLSCLITLSISAVPEKSRYTTKYD
NINLEEIIHNDRLLKNYVDCLLDKGRCTPDGLELKKNMPDAIETDCSKCSDKQKEGS
EIMMRYLIDNKPEYWNPLQEKYDPSGSYKKRYLDAKKTEVSVEPIVKS
>OcomCSP1
MVPLICVIVAGLSGLVVAAPVPEDNEPYYTTKYDHVDVEMILNNRRLVNYYTACILN
KGPCPPEGIEFKRILPDALKTNCRKCTEKQKVTTLHAIKRLMKEYKKIWKQLKAEWD
PDDIYVTKFLETYSKPTDANIFSNRFDGEESSESESTKASTNSTTESSNFSSSTPTT
LGIYLPPVPTLSPMVKPIANTIGQRLKSTVSFGGNIVGQVIKDIQRIGNTVVLTGAE
IAGNIGNRVIQRGTQIAGALRAIANPAFRFNRT
>OcomCSP2
MNRCGLSVLLLGLLLVAAVLGAPKKTLDENLKVLRKIDINTVLNNERIIKSYVDCVT
GKKRCTSEGTALKESWKEGLDRGCDTCEEEEKKKIKKIAKHIYTHYRPLYDELVESL
DKDGKYREKYKSYIDELLNDSSI
>OcomCSP3
MNSFYLSFVVGILVVVAVFGAPKTFEENLEILKKVDVDAVIKNDRIMRNYIDCVLDK
KTCTPEGTAFKESWKEGLERGCGDCDEETNRIVKKLVKHVYVNKRNWYDELVEALDK
DKKYSKKYETYINDILKDTSI
>OcomCSP4
MVSFLLVLCLAGVSLATVTEKSKYTTKYDNINLEEIVHNERLLKNYVDCLLEKGKCT
PDGLELRKNMPDAIATDCSKCSDKQKEGSEYMMRFLIDNKPDYWNPLQEKYDPSGAY
KQRYLESKKQEVKVEPITKA
>OcomCSP5

MKVTIAICVVVLMIVVASAAPADDKYTSKYDNVNLDEIIKNERLFRGYIDCLLGTKS
CTKDGEVLKKLLPDALKTKCERCTEAQKKGAKKMIRYLLQNKRDWWNELEAVYDPEH
VYVKEYENELKEEGIVL
>OcomCSP6
MEMCFILFILFTAISVRGDEEKYTSKYDNIDYRSILASERLLNNYFRCMMDEGPCTP
DAQDLKKVIPDILQTECSKCTDKQKAAGREIIKYLVDKKLDMWKKIVEKYDPQGIYK
EKYRDEWIKEGFPEL
>OcomCSP7
MKPIFLVLLIVWYLYGIVICDKYTTKFDNINLDDVLKSERLLKNYFDCLMDRGKCTP
DGKEVRDNISDALETDCGKCSEKQRTGSMKVIKFLVQNKRPMFDELSAKYDPNGNYR
TKHKEEFAKEGIVL
>OcomCSP8
MNKSVLFVFLLAVGYSAAAYSHKYDYINVMDVLHNARMLKRYVECLLDVPDTCTKDG
DYLKQVLPDAIRTNCGECDNKQKDTALRVIGYLMKHHSDWWQKIDARYSASGSQFLA
VNSAKIEEYKNTL
>OcomCSP9
MGFARLIFLLAVVSYSFAQTYNTRYDNIDIDRILASKRLLDSYIHCLLDDNFKKCSP
EGREFKKYIPEAIHTNCGKCSDSQKRIVKKAAKHIIQNRPQDWDKIRKKYDPERKYQ
QSFNTFLNSP
>OcomCSP10
MKTFVVCIFAAGFFLIHAEKYTTKYDNVDVDSIIKSDRLLLNYVNCLLDRGKCTPDG
LELKKVLPDALLTDCSKCSDVQKKGSKKIIRYLIKNKPDWYGELEEKYDKDQTYKKK
FEKEIST
>OcomCSP11
MQTFQPGLILLIVFVSVIVVKAAITEKSSSTERPSISDEALEASLKDKRYLLRQLKC
AIGEAACDSVGRRLKSLAPLVLQGSCSQCTPQEQRQIRKVLGYMQVNFPKEWNKILK
QYSG
>TcasCSP1
MLILQIAHLCAQFCLLAAIFTCVKPQLTRISDEAIESTLNDRRYLLRQLKCATGEAP
CDPVGRRLKSLAPLVLRGSCPQCTPQEMKQIQKVLAFVQKNYPKEWNKILHQYAG
>TcasCSP2
MKIIIILAVLIATAVAATYDVYPTKYDNVDIDAILHNKRLFDNYLQCLLKKGKCNEEA
AILRDVIPDALITGCRKCNDHQKVSVEKVIRFLIKERNSDWQQLISVYDPKGEYQTQ
YAHYLEKI
>TcasCSP4
MYSYLIPLYLFLFVHYGWSEDTTHKYTTKYDNIDLENVVKNERLLKSYVDCLLEKGR
CSPDGLELKKNMPDAIETDCSKCSEKQKEGSDFIMRYLIDNKPDYWKALEAKYDPDG
TYKKRYFESQKDEVSKVEA
>TcasCSP5
MKTFVILFFGVFFIIFSDFVNGKTLHRSTRDDKYTTRYDNVDVDRILHSKRLLLNYI
NCLLEKGPCSPEGRELKKILPDALVTNCSKCSEVQKKQAGKILTFVLLNYRNEWNQL
VAKYDPDGIYRKQYEIDDDYDYSELDSAKK
>TcasCSP6
MIPLIAIAGILAVSAAPAEFYESRYDHLDVESILNNRRMVNYYAACLLSKGPCPPQG

VDLKRVLPEALQTNCAKCTEKQRTAAYRSIKRLKKEYPKIWEQLRAVWDPDDVFIRK
FETSFESGKPSGVISTNTSPPSPILSNRFGENEEADAASNVISSTPLPPTTSTTTRT
TLTTKFTTKPSTKPTNKPVVVTKPPQAPPFATVGANLQATVSFGTNLVGGIVRSLGT
LGSRVVESGTKLANMVISAAIRP
>TcasCSP7
MKLISAVILCAFLVAVSAAENKYTNKYDNVDVDKILNNDRVLTNYIKCLMDEGPCTS
EGRELKKTLPDALSSGCTKCNQKQKETAEKVIRHLTQKRARDWERLSKKYDPQGQYK
KRYEEHVATSRAA
>TcasCSP8
MPLVKSLVVVVLLIGVVYQVQGQLGLAGNNYIEKQLLCALDKAPCDALGNQIKGALP
EIIGKNCERCDSRQVANARRIARYVQTKHPDVWNALVKKYSV
>TcasCSP9
MTAIVFLLALACLKTYVSSQEYLVPQNIDVDEILKNDRLTRNYLDCVLGKGKCTPEG
EELKKDIPEALQNGCAKCNEKHKEGVRKVIHHLIENKPNWWQELESKFDPQGEYKKK
YDELLKKEGLAN
>TcasCSP10
MKTFVLVAFAAVLGLALARPQEKYTTKYDNIDLEEILKSDRLLKNYFNCLMERGTCS
PDGEELKKALPDALHSGCSKCTEKQKEGSRKIIHYLIDNKRDWWNELEAKYDKDGVY
RQKYKDVIEKEGIKL
>TcasCSP11
MKTLVPLLFFVIAIASSLAENSKYTTKYDNVDLDEIIKSDRLLKNYVNCLLEKGKCT
PDGAELKRHLPDALHTECSKCSETQKNGSKKIMRHLIDHKRDWWNELEEKYDKEGEY
RKKYEAEIKGKKD
>TcasCSP12
MKTLVLVLFVAVLSVVFAADKYTTKYDNIDLNQILKSDRLLKNYVNCLLDRGKCSPD
GQELKNNLADALQTSCSKCSQRQKDGSRTIIRYLIKNKRDWWNELEAKYDPTGIYKN
KYADELKAEGIVL
>TcasCSP13
MFLAIVLVVCACTNVLSEEYTNQYNDELDAALKSERLMKSYFECLLGTGKCTPSGEE
LKKDIPDALKNECAKCNDKHKEGIRKVIHYLVKQKPEWWEQLQKKFDPQGIYKKRYQ
NYLDKEGLKA
>TcasCSP14
MFATSALFAFICIQGLVSAEEYLVPQNIDLDEILKNDRLTRNYIDCILGKGKCTPEG
EELKRDIPEALQNECAKCNEKHKEGVRKVLHHLIKNKPNWWQELEAKFDPKGEYKQK
YNKLLEKEGLQA
>TcasCSP15
MIFKIHFLVFGALLTYVSSVEYLILREIDTILKNDQMTRNYLDCVLDKGKCTKEAEK
LKKGITETMKNGCVKCEQKQKEDVHKVFQHLMIHRPNWWHELETKFNPHHEIKLQHL
HQSKFNPHEEVKLQHLHQFPHHDFLEREGFIR
>TcasCSP16
MTAIVFLLALACLKTYVSSQEYLVPQNIDVDEILKNDRLTRNYLDCVLGKGKCTPEG
EELKKDIPEALQNGCAKCNEKHKEGVRKVIHHLIENKPNWWQELESKFDPQGEYKKK
YDELLKKEGLAN
>TcasCSP17

MFKVLFVVFACVQAYVYAEEYTVPQNIDIDEILKNDRLTKNYLDCILEKGKCTPEGE
ELKKDIPDALQNECAKCNEKHKEGVRKVIRHLIKNKPSWWQELQEKYDPKGEYKSRY
NHFLEEEGLN
>TcasCSP18
MLFTVFLVLTCAHVVFLEEYVIPDNIDIDDILSNERLLKNYVNCLLDKGRCTPEGKK
LKSTIPEALSTDCAKCNEKVKANVRKVLHHLIDNKPDMWKQLEAKYDPSGEYRSKYK
DELEKNGIHV
>TcasCSP19
MKFFIAFLMLLGAVWCEQYTTKYDNINVDEILASERLLKNYFNCIMDRGACTPDADE
LKRVLPDALKSDCAKCSEKQKEMTKKVIHFLSHNKQQMWKELTAKYDPDGIYFEKYK
DKFDS
>TcasCSP20
MRFFVIFFVACVSVALARPEDQYTIKYDNVNLKEILQSDRLTENYVNCLLEKKPCTP
DGEELKRVLPDALKTSCAKCTDKQKQGAKTVIQHLYKNKQDWWKQLEAKYDPEHTYV
KAHEDELKAL

>CbowSNMP1a
MRFPVKLAIGSISAFIFIILVGFVLFPRMITSKVKGMVNLAPGNEIRDMFIKVPFAL
SFKIYLFNVTNPMEIQSGEKPIVKEVGPFCYEEWKEKMNIEDKEEDDTISYNQKDTY
LKKWWPGCRNGQEEVTIPHPLILGIVNTVARQKPGALSLINKAIKSIYSDPSSIFLT
AKVDDILFDGVVINCNVSDFAGKALCGQLRTAEALTKVGEVEKFSLFSSKNATLQKR
IKAYRGKKNHRDVGRIVEYNSSKMMDVWPTEECNSIEGTDGTIFPPLTKPGEGLFMF
SPDLCRSLIAFFVRKSTYDGIPCGEFTADLGDMSKNEKEKCYCSTPETCLKKGMMDL
YKCSGIPIYASFPHFYNSDTSYLKGVGGLSPNKTKHEIKILFESITGSPLYARKRLQ
FSMPLESTQKVELFKNFTGTVLPIFWIEEGVGLNRTYTGQLKSLFTLTKVVKVSKWL
ILIGSLGGLAAAGYLFFKVDGRADITPVHEIRRHESKSGSTVNGAGGHVLSGNGLEK
Y
>CbowSNMP1b
MRLPLKLGVAGFLLILLSVIVGFIALNPIVRFGIRQQTALKRKSEIRNIYLKLPFPL
DFRVYFFNISNPMEVQKGATPILTEIGPYCYDEFKEKIDVLDNDAEDSLTYYPYDIY
KFNAEKSGKLSDTDYVTILHPALVGMVNQATRDSPALLSIVNKAIGPIFRDPESIYL
TAKVKDILFDGVELNCKVTEFAAKAVCTQIKSQIPGIKSDPEKSIFLFSLLGVKNAT
VGKSIKVSRGISNSRDLGKVLEFDGKKVLKLWYEEQCNHFKGTDGWIIPPLLKPEEG
LWSFSADLCRNVVAEYVEDSVTKGVKTRRYEATLADMQNNEEDKCYCPTPKTCLRKG
VFDLSKCMGVPILATLPHFLEADEIYLQQVKGLNPILDKHIIRIQLEPMTGTPIEAR
KRLQFNLPVSASEKITLMRNVSTSLHPIFWIEEGVELDGALLEKVTEVFTFLGVFQV
FRWLGLLIGFVSIAYAVYHHMKHSRSVHITPISGSSSSDHVDINRSTNELVGKMKEV
FQSDKGHTNPVMTGHEFDRYS
>CbowSNMP2
MKMFGASRFCNVKILFVTTVVATVVLIGVLLLSFVGMPLIVNDQLAKKLRLENNTEQ
WDRFVELPVPLNLNVFVFNVTNSDEVTNNKATPILQEIGPYCYEERITRKILSANST
EDSITYEQSFNITFDEKRSGQWKESDKIVMVNPLFLILSQITNVIERFVVMGCIDKL
FPPKYSTMFFEVDIKTIMLEGIEFGVASDDIGPACNIVRNKLLEKTLPMKNVERIPS
PTDPSVINSLKFAFLQYKIRGPDGQYTTNRGIDDITQLGHIMRWDHSAEIDVWGRGE

STNNATCKEVKGSDSTIYPPHVTKSTKLDIFSTDICRTVQIRYKGTGTYQGDSGYYF
GIDENTFRPATPSPENDCYCIQQTMAPDGEPSCFLDGVVDVYPCFGAPILLSFPHFL
YADESYLDGVIGIDPPNSSIHEIFLLIEPNTGTPLQGMKRIQLNVVLRPVEFVEYTA
NLPSTVLPLIWIEEGVNLSQDLLDKLDKMYFNVIKAADAAKYAAIGVLTAFVLISGG
FFVRKRYFK
>CbowSNMP3
MKFYSVLFVVKDRANMLNKFNITVSGKIIVILGVFGLFCIFAGFYVGFKAVPDVITD
KIWDMKVLKENTEQWGMFMKTPFPFTFKVYLFDVQNPQEILQGAKPVLRETGPFVYK
VYKWKSEVEWDTPDDISYFSYMRFEFDRKASGIFSEDMKVTLFNTAYYGMLQKIDET
QPEVLSTVEGVLPSIFGENHGLFIKVKVKDYLFDGLKICENEGKDGGFVAGMVCKQM
IARLPESKNLRLEDNSILFSNMHYKNNTHQGRFTVKSGGQNRTETATLTLFNGKSYI
SSWTGEKSMCNKIRGATTVFPVNIEKNMTFEAYSEDICRTIPLEYSAEETVKDIVGY
KFSAMNDSFSSTKKENFCYCTNTTRTLDGEYGCLKDGVTDLKTCIGSSILVSFPHLL
YGDEEYLDSVIGLNPEKSKHETTVILEPISGFPLSVTQRIQFNTFLRPIDNVISLEN
VSKSLFPLLWVEESLILDDQYTDMLKNELFRTIKIVDIVKWVTIGSGAACVLIALIL
RMSSKTT
>LdecSNMP1-like
MRFPVKLAIGSFSAFIFIIPVGFVIFPKLLTSKVKGMINLGPGNDLREMFIKVPFPL
SIKVYLFNVTNPMEIQNGEKPIIKEVGPFCYEEWKEKIDVTDLDADDTISYNPKDTF
LKKTRPGCRTGQEYITIPHPMILGLVNMVVRQKPGALSLVNKAIKSIYGNPSSIFVT
AKADDILYDGVIINCGVSDFAGKAICSQLKGADMLKKISENELVFSLLGPVSSNDGK
NQKKKLFIHFSIVDCHYINFNL
>LdecSNMP2
MKVLKENTDQWDMFMKTPFPFTFKVYMFDVQNPDEILQGAKPIVRETGPFVYKVYKW
KSDIKWDTPDDISYYSYMRYEFDEEASGVYTDDLKVTIFNTAYYGMIQKIEDIQPEA
LPMVEGVLPSVFGENHGLFIKVKVKDYLFDGLKLCENGGKQGGFLAGMVCKQTVDRL
AENKNMRMENNTILFANMHYKNNTHLGRFTVKSGIKKREETATLTLYDGQDYISVWT
GEKSICNRIHGTTTVFPVNIDKNMTFEAYSEDICRTIGLEYSEDETFKDLLGYKFVA
KNDTFSSKKEDNSCYCTNKTRSLDGEFACLKDGLTDLTTCRGASVLVSFPHLLYGDK
EYLNGVFGLKPDKSKHETSLILEPVSQELLTMTIKI
>PaenSNMP1a
MKLRLSVKLAIGSICAMFFIILVGFILFPRMITSKVKAMVNLGPGMDIRGMFLNVPF
PLSFKVYLFNVTNPMEIQTGAIPKVEEVGPFCFEEWKKKVDVTDEEEDDIITYLSVD
TFLKKSSPGCVSGKVIVTIPHPMILGLVNAVSRAKPGALALINKAIKSVYENPTSIF
LTSTADEILFDGVIIKCGVTDFAGKAICSQLRESGSLKIVNENDLAFSFLGMKNGTE
QKKIKALRGTKNYHDVGRIVEYDGSPVMSTWPTDECNRIAGTDGTIFPPMLLKEEGL
VSFAPDLCRSLKAFWVQKTKYDGIPVNEYTASLGDMSKNENEKCYCYTPDTCLKKGL
MDLYKCAGVPIYVSMPHFYDSDESYLKGVKGLNPNKTSHEISILFEQLTGGPVSAKK
RLQFSMPLEPIQKVDLFKNFSTTVIPLFWVEEGVDLNNTFTKPIKMLYTMKKVVKIS
KWLILLSSIAGLVASGYLFFKSNQTISITTVKDIKKGPTSGISTVNGHMNRSMSDNE
VNKF
>PaenSNMP1b
TQLCRNIALDYVEDEIIKGINVRRYEGNLGDQQLVEADKCYCPSPKHCLKKGVFDLN
KCLGAPIIASLPHFLYADEIFLTQVKGLEPIKEDHVLAVSIDPISSAPLSLRMRIQM

NLEIGPNPKITVMSNLTDALHPIFWLEDSLDMEGPVLKKVSEIYIAIKVANILKWFA
LVMFFVFFAFGIYLHFKNRKSAKITPVQRRPASDKDTFYKSTNEGQNNIMSGHEFDR
YN
>PmacSNMP1a
MKLQLSVKLAIGSICAMFFIILVGFILFPRMITSKVKAMVNLGPGTDIRGMFLNVPF
PLSFKVYLFNVTNPMEIQTGAIPKLKEVGPFCFEEWKKKIDVTDEEEDDIITYLSVD
TFLKKSGPGCVSGKVIVTIPHPMILGLVNAVSRAKPGALALINKAIKSVYENPTSIF
LTSTADEILFDGVIIKCGVSDFAGKAICSQLRESGSLKIVNENDLAFSFLGMKNGTE
QKRIKALRGTKNYHDVGRIVEYDGSPVMSTWPTDECNKIAGTDGTVFPPMLLKEEGL
VSFAPDLCRSLKAFWVQKTKYDGIPVNEYTASLGDMSKNENEKCYCYTPDTCLKKGL
MDLYKCAGVPIYVSMPHFYDSDESYLKGVKGLNPNKTSHEISILFEQLTGGPLSAKK
RLQFSMPLEPIQKVDLFKNFSNNVIPLFWVEEGVDLNNTFTKPIKMLYTMKKVVKIS
KWLILLSSIAGLVASGYLFFKSNQTISITTVKDIKKGPMSGISTVNRSMSDNEVNKF
>PmacSNMP1b
KEKINVIDNDAQDTLQYDSFDTYIFNKTLSGKLSDEDYVTIIHPLLVGIVNTVNATS
PALLSIVNQAINKLFKEPKSIYLTDKVKNILFDGMEINCRENDFASKAVCTQLKSQI
SDIKESTTQKNVLLYSLFGQRNATIVDTIKIMRGIKNYRDLGRVLEVNGKSDINLWG
SEHCNRFKGTDGWIIPPLLKPEEGIRCYATQLCRNIALDYVEDEIIKGFNVRRYEGN
LGDQQLVEADKCYCPSPKRCLRKGVFDLSKCLGAPIIASLPHFLYADEIFLSQVRGL
EPIKENHVLAVTIDPISSAPLSLRMRIQMNLEIGPNPKITVMNNLTDALHPIFWLED
SLDMEGPVLKKVSEIYVAIKVANVLKWFALAIFFVFFAFGIYLHFKNRKSAKITPVQ
RRPASDKDAFHKRTNGGQNNIMSGHEFDRYN
>TcasSNMP2
MGCSCCTIKVLLVCVVISVALLIVSLALAFKVFPDLLESEVNKAVRLEDGTKQYDRF
VELPFPVDFKVYLFNVSNPQQVLDGTEKPKLEEIGPFVYKQYRKKTILGKNEEEDTI
SYTQKETFEFDAEASKPLTEESVVTVLNPALMSIYQLAEDLHLAGAADTCIKQTFEN
NQGKVFIEANVRKLLFDGFSFCKNTSPGICGLVNDLICAIAATKRNSDLVLPDYSLI
FSYLNYKRKPDDGKYTVKRGLTNIEKLGHIVAWNDSLYTKFWGEGTTCSEVKGTDST
LYPPRVTTDSAFYIYSTDICRFVKINYKGEESYKGIDGYLFETSEDTLRSSAPEEDC
YCSKLSRDMEGKKSCFLDGVIDMQTCFGVPVLFSFPHFLWADNKYLSAVEGLNPVEE
KHKTYLVVEPNTGTPLKGMKRIQLNGVIRPIVGIKSMLQTKRALLPLLWIEEGVSLP
QKYVDELKSSYFDKVQIVDGVRYALIVISAILVGAFGIIILRKRSHAKHHV
>TcasSNMP1a
MRLPVKIAIGCAIGLVVIIVFGFIAFPKMIKGKVKSMINLNKGSEIRQMFVKVPFAL
DFKIYMFNVTNPMDVQKGALPVLKEVGPFCFEEWKEKVDLDDNDDEDVMFYNPKDTF
YKANGPGCLDGSQMITMAHPLILGMVNTVVRTKPGAISLISKAINSIYGNPDSIFMT
ASAMDILFDGVVIKCGVKDFAGKAVCSQLKEAPDLRHVDENDLAFSFIGPKNATPGK
RFKVLRGVKESHDVGRILEYDNKKEMEVWPTKECNQYKGTDGTVFPPYLTKEEGLAS
YAPDLCRSLVAVYSGDTKYDGIPVRIYTATLGDMSKNADEKCYCPTPDTCLKKGMMD
LFKCAGVPVYVSLPHFYESDESYVKGVVGLNPNKKDHGIQILFESTTGGPVKAAKRL
QFNMPLEPNPKLPIFANLPNTVLPLFWVEEGVALNNTFTKPLKDLFKIMKIVKIAKW
LIMLGCLGGLGAAGYLYFSKKGEANITPVHKVKPAENGVSTLGGEVNHAMSDNEIEK
Y
>TcasSNMP1b

MVKWQRQLKPGNEVRDFYIKLPIPLDFRVYFFNISNPEEVKQGEKPILKQIGPYCYD
AYKEKINVEDDKDNDTLTYNPYDTYFFNQMRTGDLSQDDYVTILHPLTVGIVNAVAT
QKPQYLSAVNKALPVIFKENSSIYLTAKVREILFDGVLINCNVKDFSANAVCSQFKG
QPAMVEVEKNIYSFSLLGSRNGSIPTRITIHRGVKNAADIGRVVTIDNKTDLDVWPE
PECNAFRGTDGWVFPSFLEKEDGIWTVASDLCRSFKAQYVEDLKFHGVVVRKYFADL
GDMSSNPAEKCFCPAPEKCLPKGVMDLTKCMKVPLYCTLPHFLRADEKLLQQVEGLS
PELERHIIKIYFEPLTGTPMLGQRRIQFNLQLMPIPKVAMMKTVPEALHPILWIEEG
VELEGFLLKKVTSVFTLLKLMTFVRYIMLGLSIQGILYGGYKLYQESKSKKVSPVQN
GTTESKNHNQGKTGGIELPSMNKRNKENTKNA

>TcasSNMP1c

MSYKKITIISACCVVTIIGVAYIYAIRDISHRRNVRYKYIDRVNNVSNDVNGGVVSV
GYCYDYKRIDVDNADSTYTYDIYNRSGNSDDYVTIIHVVSVNYVSVKTHYNDAGKSI
TAKVRDIDGMINCTSRDTAMAVCTIRTKIGISKDYKYAGNGTTRITVRGIKSNGKVA
VDNVTKSDWSNCNYKGTDGWISGRKTIWMHATTCNIHADVGATSNGAVNKYYSDNIC
TNCSCGIDVTKCTAIYISHRSDSIRGVKGNDTSHITRIGTSMAIRNVVKKITIMNVS
VIHVWVMGVVNGWRMIKTYTAVMKYISVASGTAYGGYHYKNKKYSKNIVSSK

>TcasSNMP1

MTSTARRRNIMKKVYKIMDRVYNITNSVNGVVKVGYCYDAKKIDVNGDSTYTYTYND
KSGRTADDYVTVHIVGIVNTVSRDSIVDRAIKSIKDNIYITTKVRDDGMTINCKVDS
ATAVCTKAIGIIKNVYKSIGRNGTNRYKVRGMKKWHGRVVNHKSTVWSTKKCNRRGT
DGWIIDKVGWTYSSDCRNMHVVTSHGVAKYYADGDMSSNDKCYCKTCKGMMDTRCMG
VIYATHRVDKVRRTVRGKITDHIVRVIIGTAKRMNIVKKISMKTAHIWIAIVGKMIK
VVVAKVDVVKYCAVCAVAGSYCYKRKKKAVTVSKTAKA

>PstrSNMP1

MQLSVKLILGGAAMLGATVLVGFLGFQPLVNVVVKDQTSLRKRNQMRKLYLNIPFPL
DTRIYFFNVTNPMEVQNGSKPILQEVGPYCYDEHKNKINVADNDAEDSLRYDAFDVY
RFNKNRSGNLSDEDYVTIIHPLLVGMVNRVASDSPALLSILNQAFETIFKNPQSIYL
TDKVKNILFDGMELNCEGADFAAKAVCTQLKSLVPGIKEKPTNKNVLLYSLIGPRNA
TVASTIKVLRGTRNYKDIGRVLEVDGKKQITLWGSDFCNRFRGTDGWIIPPLLDPAD
GIQSYTPHLCRNIDLKFVKDDVIKKIQVRRYETTLGDQTNNTLDKCYCSPKRCLKKG
VFDLTKCVGAPIMATLPHFLETDQSYLSQVDGLHPNWEDHILNINIEPMTSAPLDIK
IRIQMNLEIGPQPKISVMKNLPVALHPIFWLEDGLELEGELYEKIANIFVLLKMAQI
LRWTFIVVSIAIIAYGYYLIMKNRKSVKITPVHSASSYDSYDNEAFNNRSTNAIISQ
LKTEMNYPKNYRNVSNNNTNNNNVGGGHEFDRYS

>PstrSNMP2

MVNKNGLKLSNKVLIFVSVLGALMVIGGAYLGFKVVPDIIVNKIWETKILKENTEQW
EAFMKTPFPYSFKVFVFDVQNPDDVLQGAKPRVKEVGPFVYKVAKWKDDVQWTSPDE
ISYHSYTKFEFDEASSGEYTENTEVTILNSPLYGILLKVEATKPEVFGLVEQAVPVA
FAGHSQLFIKVKVGDLLFKGIKFCENAGENGGFATSIFCRNVMQKANESQSLRLEND
AILFSNLHYKNNTHLGRFTVKSGGKERKESAALTLYNGKPFLSTWPGENSSCNRIRG
FTTVFPANIKTDMVFESFSEDICRHVALEYDSKDAVKEIAGYKFVAKNDTFSSKTNK
ENSCFCSNRTKTFTTAEGCPEDGIIDLTPCKGGPVMVSFPHLLYADEGYARSVEGLR
PVKSRHEPFVILEPLSGLPLYGSQRIQFNMFLRPIEGMENPWNVSRSLLPLIWVEES
FVIPDQFIGKLNDNLFSKLNMINIVKWIVIVSGGAGLLLAGLTLVYRQAP

>OcomSNMP2
MKKILKSKYCSVKVLLICLAVLLVVLVGVLILSFYGIPKIIDVIVHQNVELKDNTEQ
WDRFIDLPIPITLNAYIFNVTNSDAVLRGEKPILVEVGPYVYKEQIHRNVLSTDHDS
ITYQQSIGITFDQELSGNLKETDEICIINPALLVLTQITSPLEQLAITGCLDKLFSP
EYNKLFVKVNIKTVMFDGIPFAQVSPDVGYACNVIRNQVIEKTKKIRNVQRIYNKVY
NDVVDGLIFALLRYKSAAPDGVYTINRGIDDVSQLGQIMRWNYSSHLPYWGRSQSKN
IDSCQLVHGGDSTIYPPHVTKDDILSIFATDVCRNIDIFYTGKDTYKNIDGLKFEPK
SSTFRSTLSNIEDDCFCIKLTKNAQGQLSCYLDGVLDMYECFGVPILLSFPHFLYAD
ESYINGVEGLTAADPQKHGIYLLIEPNTGTPLEGRKRVQFNMITRPVEYMSFTKKIY
STVMPLVWIEEAVDLTDDLVEMVNNKYFKNVKIATGVKYGLIAVSASLVIVVTTILV
RKSLF
>OcomSNMP3
MVNFFVFNVSNKFLIPLGIVGSILLFGGVFVGFKIVPDTITDKLWETKILKENTQQW
DIFLETPFPFTFKVYVYDVKNPEEILQGGKPVVQEVGPFVYKVFKWKKEVAWTTPDD
IAYNLYTRFHFDKDASGFYSDDTEVTILNTMYYSILLKIEETQPDAFNLIEGELPTI
FGENDGLFIKVKVKDYLFDGLKFCENEGADGGFASSLLCKRIIGKMNESRNLRLENK
TVVFANMYYKNNTHLGRFTVKSGQNNREEAGMLNLYNGKDYITSWAGDKSICNKIRG
VTTVFPVGVRRNMIFESFSEDICRAVALEYSSDETVKTIKGLKFVAKNDTFSSTKKD
NSCFCLNKTRTFDSSLGCAKDGITDLSPCTGGPVMVSFPHLLYGDKEYQTSVQGMEP
NKSKHESFIVLEPISGLPLYAAQRIQFNMFIRPMEGMESLNNVSRALMPLIWIEETV
VIGDQYIEMMKSQLFHTMNLANIITWVVIISGSGCLLLAVLIVIHKQAP
>OcomSNMP1b
MKLSLKFMIGGVVLLLGTIIIGFVEFQTLVEFVVKDQTALRKRNEIRDIYLKIPFPL
NFKIYFFNVTNPLEVNNGATPILQQIGPYYYDEYKEKINVIDNDAQDTLQYDSFDTY
IFNKTLSGKLSDEDFITIIHPLLVGMVNTVTSTSPALLSIVNQAITSIFKQPKSIYL
TDKVKNILFKGMEINCQGSDFASKAVCTQLKSQIPDIKESTTQKNVLLYSLLGKRNA
SITDTVKILRGIKNSKDLGRVLEVNGKSEIGLWGSENCNRFKGTDGWIIPPLLKPEE
GIHCYTTNLCRNIALEYVKDDNLKGINVRRYEGNFGDQQSVAADKCYCPSPKPCLKK
GIFDLNKCLGAPIIASLPHFLYADEIFLTQVKGLKPVKEDHILTVSIDPITSAPISV
KMRVQMNLEIAPNPKITVMNNLTTALHPIFWLEDSLDLEGPLLSKISNIFLLLKIAY
VIKWIILIASCGLFAYGGYLHFKSSKTVKITAVHQRSENTLNRSTNELVAQLRNEEK
HGISNNIMSGHEFDRYN
>OcomSNMP1a
MNLVQFQKFLLSQNKFNNTIMKLRLPVKLAIGSFCALFFIILVGFIIFPKLITSKVK
AMVNLGPGMDIREMFLNVPFPLSFKVYIFNVTNPLEIQNGAKPEVKEVGPFCFQEWK
KKVEVADDEKSDIISYISVDTFTRTSGRKCVSGKVVVTIPHPMILGMVNTVSRAKPG
ALALINKAIKSIYENPTSIFLTDTVDNILFDGVIIKCGVTDFAGKAICSQLRDSGTL
NIIDEKNLAFSLLGPKNATEQKRIKALRGTRNYHDVGRIVEYDGSPVMTTWPTDECN
LISGTDGTVFPPMLLKEEGLVSFAPDLCRSLKAFWVQKTKYDGIPVNKYTASLGDMS
KNENEKCYCYTPDTCLKKGLMDLYKCIGVPIYVSLPHFYDSDESYINGVIGLKPNKT
EHEISILFEQLTGGPVSAKKRLQFSMPLEPNPKVDLFKNFTNTVIPLFWVEEGVDLN
NTFTKPIKMLNTMKTVVKVSKWIILVASLAGLAVSGYLFLKNDQTISITTAKVIKKA
PASGISTINGHINRSTSENEVN

>PstrOR1(Orco)
MMKFKVAGLVADLMPNIRLIQASGHFMFNYHADNSGSLHTLRVAYSCMHLVFCLVQY
GCTFGNLLLERGDVNDLAANTITVLFFSHCITKFVYFALRSKLFYRTLGVWNQSNSH
PLFVESNNRYHALALKKMRTLLICVFTTTVLSAAAWTGITFVGDSVHNIKDPENENL
TIVEEIPRLLVKSWYPWNAMSGGAYYITLVFQVYYVLFSLMHANLLDSLFCSWLIFA
CEQLQHLKEIMKPLMELSASLDTYVPKSADLFRASSANSQDNLIENDYNQKNEELNL
KGVYNTRQEMGANFRSGALQNFGQGGGGVGPNGLTKKQELMVRSAIKYWVERHKHVV
RLVTAIGDAYGVALLLHMLTSTVMLTLLAYQATKINGVDTYAATVIGYLVYALAQVF
HFCIFGNRLIEESSSVMEAAYSCHWYDGSEEAKTFVQIVCQQCQKAMSISGAKFFTI
SLDLFASVLGATVTYFMVLVQLK
>PstrOR2
MVFIFQNIVVFWNDIICLNLDTFTSGLLMQIGMQCDFLSHSLNNLGAYRVRNGILVE
QNNIAETSLCRKTFSEIVMENLVVCIQHHKKIKRTANEIEKLHKKTVFILFLGGGMI
ICSGLFQLSTAKLGSIQSFMLISYTACMLIEQFMYCWFGNEVIDKSSKIFNSAYNIP
WIQCDMKVKRTLLQFMTLTKYPIKLKVGGMIIMSNAVFVSVVKSSYSIFTLLKKIQK
>PstrOR3
MSGDRVTFDFRKFFERDFFMLKFLGIYYLTENAKLGQILYSFIVSLPLMVVATMVQI
INMFCVEADLRKMAASGYLGASCLMAVIKELFLFKNKKFLLNIQNDMNRSEFQPKDA
EGVELVERCLSGYRKTRKVVLIVCSIAVSGCLTMPFLSSTQLLPLSAWYPFDVTYSP
VYELIYIHQSVSLIYLAYMNIYIDILVSGFTTFVSLQCALLCHKMESQRNINSVKEF
VIHHKWILNFVFNIEKLITEMYFLQFSACTVIFCLSWFLLTMIDVNSFEFVYLFAYQ
TAIGSLLLIPCWYSSEMERQSQLIPFSVYSLPWRTNTHQFKKDVYFFLQGVQKPIFI
NIVGLFPLSVDTFTKILRSSFSYYTVLNNMNLKSQANN
>PstrOR4
LMMEIYVLSSDLKFNIFLDILVVLIIATGHLVKIVIFKVKTKVWLDIFDQLKSPCFN
DYPEEFEYNNKRHVNFSKNFGNLFQFSCCWSTGMMIFQPLFSNVDLPMNCSFVTEPF
IPYMYAGQVMFILLCAFSHSSIDVLITDLIGIIVGQLDVLGEKIKAYDTNKTKEKID
LKQCVQHHVEIIKFVNNLRQATSVIILIEYVKSALVVCFLGVQAVHIDPMSLTFLKM
PVYLIVMLFQLLIYCFFANKIIDKTQEIGDACYFSMWYFYKNTCDKRSLILIMERSK
RPLYLT
>PstrOR8
MPDSYPEDFFVVNRWILKFAGMWRPDTDNGIIQALYTTYVIGIFLFVNIFFTSTEFL
SLLYTYGNEYDLIKNISFALTHLMGAVKVVFFYYQGRSLKSIMSTLESDELRYESCE
KSGFFPGLVAKSYKRTGIKYTVIFFMMAHATLTSSYLPPTLAALKYNEDDPESLLPD
RLPYYSWMPFGFDTAGTYLIALGYQAIPMFSYAYSIVGMDTLFMNIMNCVGMNLEII
QGAFLSIRDRSLEKIDGAPLTADGLYNTTALNGVMRAEMRKISRHLQTVYRMCERLE
DVHKFLTLAQTVATLFIICSCLYLVSSTPIGSKAFLAEIVYLIAMGFQLTLYCWFGN
EVTLKANEMPFYIWQCDWLTADDDFKKSMIMSMVRSKKPVYLTAGKFAPLTLPTFVS
VSITHEKEHSLVKFIFTDTEGFVLVLRSHQKHQRVESSNQTFFTV
>PstrOR9
MGEMRDINFKEIVSVQVFMLEGFSYMLPQYYTIPKVAYFCAAFLIYIVGFFYSQLAC
EFFKIYFNAENVTDVLSDSFLFLTHLVQTTKLGYMYYYRKRLWDLIESLNQPAFRPV
SLAQRDTLGRYIGMAKFISYKFQILCLLTCMFWTFYPFTLEELMLPLDSWYPFNTTY
NPNFGIAYFHTSVGSWLNGTSNIAADTLFAGLIMAACAQLEILRDTLTNLRDYAKAR

LGGLGENSGKSGGIPAILMDEMSALLVDCVNHHRCILNFVREFQFIFSYAILAQFVV
SVIIICTTMYNLTLIPFGSMQSISLIIYQYCILLEIFLWCYFGNEVMIQSNLLCDAA
YKCDWTDCSPTFKKHLLYFMTRSQMEMNIYAGSFFTLSLTTFVKIVKSSWSYFAVLI
SMNK
>PstrOR10
MLPETRSKHGYPADFFEANELIRKITGMWLPTKHHSIITKTFYFSYVFCLYGFGFYF
LVCEIIIVNDVSTEISKLVSYIGMLFTHLVGCLKFSILVFGRTKLQKIMNILQDSRY
FYEPNENFSPGLFLVEGKKTSAQFSMLVLIMYSCVGASAHISSLVTFDRIIEGDSLE
GTNFTCQDFMQYYFKIPFETNTKERCKLAFFFMDFGLLVFALVIACYDGVLVTILNC
LKCQILVVCNVFKTLRSRCLSQLGLPVWYETFSDHEHVALEREMYRQLSHTTRHLQA
LLSVTNDIEFMFTFVTLAQTLASLLIFASCLYVASSVSMTSPEFFAQMEYFSCVLVQ
FLLICWYGNEITTSGELIRSSLYESDWYSSSMRFKNSMIITMMRMQRPLYLSIGKFS
PLTLVTFVGVCRGSFSYFALFKSVQ
>PstrOR11
MTSEGIVPECKHLKFALPYIIVGGVWPNIFNSSKFWEIVYKAFSHFMFYASVCIVLG
CAGQFFVLLQDRPLKIEELLSGFSVTTIWTMAIARANGLRKPVFKKLIQSIIDSEKR
IFFSGDKDFINIQKSYISKNNFICGIYSTTLSLYTYAFSLKFMFINSEVVDPLLKET
VIRQPHTIQIWLPFDKDLHYRSAISLEGFLTVMITYLLIGSEVCSYSIQTYVVSQLK
MLNHILMNMDKFELRVMKQIGCGEEEAMFIVLRETILFHQFLLGYIEDYNKSMDIVA
LIDFILLSAQLAACVLPEVIGLPPVIGISYLFLGLIILSKFFVYYWYAEEITSEADK
VAESIYNSNWTEKPLKIKYMFIFVMMRSMKMTGLSIGPLGVMSMRVYLMIIRSTYSF
VALVHSFF
>PstrOR13
MENDIKLASMSFSIKVFQFLGGFPKDQEFTNPSKVFYFKYVMRAVLTSVLLVSSSLH
LTQNIIENIFNHIELDIAYTTAMFTGYSLMFSFLLKMDLIVRLYKELSNFEEYEKPM
DFEETNKKYNKYSLMHLIYLQIIIMMSTLGSNIFKVDTCRQENVEKGIHEVCGLYLY
TWLPFDIDFTPVRQIYLIWQLVSAQYIYTFIGVCSWMVFESVEHVNIRFKHSAHLYS
LVPEEEHAQKRRLLFNKAVRYHQYSIRLAEWIAETYSVFMFAHMFMTSVILGYDLYA
FLKTYDVSTFIMSIAWVNGVFMVSHGGQRLQDMSRQVGDEVYASNWTDYDISMQKEV
SFVIMRCQKHMSLKAIFIGDVGYMPFLSVLKAAYSYVMLMTNTGS
>PstrOR14
MTSEGILPKCKHLKSALPYIILGGVWPDIFNNLSLFWKIVYKMYSLLIFYSSVGVTL
CSFAQFYILLQDRPLLITAELLTSFSLANLWSMASVRAYGIRKPIIKKLLKGVIDSE
KRIYIGDDKDFIDIQNTYISKNNTICFIYSNCLSIYLVFYFLTFYFVTYKMSDPALL
QTDVIKKPHIIEMWFPIDRDKYYATTIFIEGIVTVTVTYLVVGVDCYSFSVQTYIVS
QLKMLNHIFENLEKFELRVKTQLGCDENEAKFLVLRESIIFHQYILRYIDDYNSTMD
IVALTEFILLSMQLATCVLPQVLGLSPIIGVFFLLLGVIISSKFFIYYWFAEEITSG
ANDVTDSIYNSNWTEKPQKIKYMFILVMMRSMKMTGLSIGPLGVMSMRVYLMIIRST
YSFVALVHSFF
>PstrOR15
MHYPIEKGKPYYSTLLSMKFLGVYPKSSSEASLKASMVTTVCTIIFAVVVICVGGMG
HLIASFKGSKSVEMSEDLAVAVGSMAFLLCALFFKLNWRNWANFFHSITDFKTYGKP
EDFDSVTIRCNFLSMMYSIYISGGLCVYAVISIFETKCVRDEQNNFCGTLTQIRLPI
EGEISTLAINIIFLCQLSLCIWACVESGNVFFLSHESSEFIICHITALKKHIIGIFN

YEDEMSKREQLLHCIRYHKQIIEWGYDLNRLTKSTLGHMSLIAAIQVGMIGNQILHK
YKMFGAAIYLAGYIMAIFLVSHAGQRLSDESISIAKAIFDSDWTKASTGMRKDLTFM
LARSQIPLFISCLPLGNFNYALFVTMLKASYSYLTLLKQSTSNKDD
>PstrOR17
MKHLQIAKWIMIVTGFWTFKKEYFTKQQHLFYRAYGIIMQLYFSCFIGALCVGLKAV
QDNIQLIDAIGLLIFCSVMLIKIIICQRKSVRSIMNRIVDFDDESDTLTGKFRDIYL
KNSRYNVIYGLSLFLVSAMCGVTLVFCDVAIYSYDMKRVKSGDLNSSDVRRPLPYHI
WRPIDEKKHYYWAFLLDVLTATIGCTYNTATQIVYLSILTFILGQIKILQVKFEQMG
AVCSRLGSEQERFMYLRSLIVEHQNIISFVKDLDENMKYLLFVEFTINPLQITCNLY
EVLVFKVDGMFLFRVVLLLCMLIQMFTLTWHSNEIQVLGMAISQSVYDGEWYDLSEK
FKQRLLIVMMRAQRPLTLAVGPFFILTNSTAVTSVKAAYSYLALLNNKRGD
>PstrOR20
MLLLSAFLQVLLRDCSFSDLLDSCIFVVNETTFLFKLLQIHQKAELLEKLRLYLQDI
RATDVSKRMMENLLVRQSKRNKIALIYRSLLCLFVVEHFLMLFIQTERKFLPIMTWI
PYDYSKFSLYYPTFSFQIVVVSISALSDISLDCIYYALIDVVCCQLDILIYYFKALN
FSKNRDFIKTKLKMNIVYHQKVIDFVKDIEKLYSNIIFSELLKSLIEICFIGFRLTV
LDVSSMEFFMQLFYFMGMFCEILCYCWFGEILTLKSKEIGDACYMTPWNEFDKELKN
MLLIMITRSQVPLVIRAGFMKLSLRTLTGVLKSSYSYAAVLKTLYK
>PstrOR21
MTNQKLLRKSFWQIEFLLKLNGLLPGERYQILYSFYTSCLLFVFKIVFPILGYTFIY
KADPSERMILIGNSFVYIEVLVLVFKHWPFITNPDLTKRLLNQWNESIFNTEVEIDK
HIIEESMRQRKIKHNVYFYSATSAPFVMLINVILSDHSDLKLPIWMPVDVLNSTSWY
VCTNAFVFSAYLHGVLGHFCVDMLIVSYMLYCAAQLKLIKYKLENMEQYLDTDLTTP
DQIGLDDLVQKKIYAQITQCVKLYDAVFSYVQELENVYSIGVFAQFLVASIVLSICL
YNMSQLESLEMNLAYVMSFFVPMTYLLYLYCNLGTLVMEESTTIGDAILRSQWYNYD
INSKRSLMILIERSKRPIRFTAGKLIDLSLDTFVLMVKRAYSLLALLKNFN
>PstrOR24
MIMDEIAYKIMKTKYYGKMVIWLPKILLGLVVMWPKAKVSLMRRFIVTSGSIFIISF
TAIGLLNTLNFTFESINHNLIGSLLIMSCFQGVSKIILMSIKFKDIADIIDNISLKF
WPDDLTGYVEVDKEIKKFYIFTMSTCLVFLGTVAVFFSIFFITPLLIEERILPFNVE
YSFDWKVSPNYELIYLSQVFYVHILALYVIGTDFFYLCTVVSVIIQFKILQQCLSLL
NSNEMVEIMNKLDINSIIKSDKMDDLCKEYLKKCVKHHLLLLRFIKKLNAVFNLIIL
TEWSTVMLSMCLLIYMLVQNYAVNTFFETALALSMVIAYLQQLAFYCIVGSIFNYQI
NLLPEHLFGSKWIVINNEIKKDLTFVLQHSQQNLTLNVYNIYELNMVSYLQVLKLAF
SVYTLFSNVSN
>PstrOR26
MDLVYPQPDQHRVTDDPLGRMRSCLTLHGFQGKRKYFWHLAGILKAAILFGRTIYAC
QTLNQPKKLAELVATYPIRFLAVVKISVLFIDRKRVKYFYDSVSRDFWNFDVAGPEI
EKQVKRRFHWVNLTALCQFSIAIVALIIFIMFPLVEMPEGKRTLPNIIWTPFDTNPS
PVYEIMYVVLLWNLFMSVLGNAFYDATFIYSTQHLFVQFTLLKELIRNVSSGIMSES
SDLERFDSEHFQKAVNERLKICIEHHVKLLKYGKNIEEFSTTVMVPQLIMSYAALVI
NGYIISVDHADVAKTMGLINLTCGSVVQLVLYSLLASDIKAQSISVIDEIIKTDWYL
FKAPIKRALIFMMMNVKDGIVITAGGMANVDNEVFVAVLINNLDVYEFIAVEFSRWF
LKQYQPLLY

>PstrOR27
MQGNNFLKYPILMSKATGQWQFEHTSALVSIYNKFSDFLLIIFIFCTHYIVLALPFY
YKCEKALIEIIFYYVHFIIAIVLTVLLKSKSIRSFLRWIIRYEKSTRFEHQQEIEIY
QYYCKMNNNITVFFVIVINIVSWMWYALNKRVSPSEELGPHCPDIRGYVFKIWYPFE
IEDFPWICATFDFVTVFCGMMLFCYYKIMPMSLIIFLMCQLNLLKFRIRSFDETKDR
NGLARSERLAGIFRMHQLCIRLMNWIQYSLKEIILIQYCSYVLDTAAFMIQLLTEET
MRGKMPCFAGFMMTVIQMYIFFWFANEIQEESKTLSDVIYNEINWIDACKSEKKMLM
LMMMRSQQMLTLKVAAIGDLSLNSFTKIMRLCYSVVTFFTTVYDK
>PstrOR31
MTRRNAETEECSYEFLRTDDCSKKTLLLPECILRFIFMWPRSDVKKQKVFYLFSMIL
FIFTQWGLVRFLIVNFDDYTRSLNVVSSMSTIFQASLKMSVLLYHSRSLSLILNDIS
HRFWPYDLLPNCTDRLRNSYRTKLVAMCTLSFTGLLFSLGSIVMPLLSHNAKELPYK
CLYDFDTSVTPLYQIMYITESVLNAYIINCTVLGFDFLFMGVGENLINQYIMLRLTI
ENFGTKWVTNFNNKIRKLGLAEHLEGEDNTVFLKNYISHHQIIIRSTKIVENIFNLI
AALQLCSSVVAICVSAFIATRENVGTAQIATMGSYLIGHLIQLNFYCAVGNDLLYES
NSLTNHMFASNWYRIKNISLQKDFIFVIKNAQVPAKITAFKVFTLDYSTYIKVLRLS
FSFYTLLSSLVEVKN
>PstrOR33
KRYQLQTAKNAKIKGYCKKFLPQCMTEKKSLIRENFYKTEMMLRNQGIYPVEGYPIA
SLFYAYLLYFGFTALIPLLGFCNIIFAESSERMGMLSNSFIFFEMGVSMFKHWPFIR
HPDITKKMMDKWTTEMFTSDEPMEMPIIKKSLKAIDFVFYLFLAFSIGGPVGLTLNV
LFSSHDELKLDIYTFGVDVIHNKFLYVLTNVYLIIGYVHGTLGHFAVDIFIAGLMSH
CSLQLSLIKFRLENMDKYLDKDLRSVQDDNTFTEEVNDKIYRRISHCVDHYDTVMNY
VKEIEDMYSVAAFSQFLAGSLVYAICLLYFMQLKALDFNFVYILTFFFPMIMVVFTY
CYYGDRISEESSSIGGAIFSSPFYNYNKKCHSSLIILMEKTKRPIKFTAGKLLELSL
DTFVLIIKRSYSLLAVLRNYNN
>PstrOR34
MFFDRLAYRLMKTTDFCKLPLWFTKLLLQSALLWPVQKASATKKLLLFAVIMICCTF
IKFGLLVTLTSKSSNIVESAAQIRDITLILQASVKIVFLYYNHKKFQPFINMILNDF
WPSNLFDEKSENRLKTFYNAVITMMILNTMFSTAYCITVTVSALLKGEFQMDTIYPF
LDYKSSPFYELNVMLQYVSLQYFCHVGIFGADFLFMSICTCVISQYKLLCRALSAFG
TDEMMEINEKLRGIGTDQLIGRNYGLHKEYFVRCVNHHRMLLMLTENVNSIFSFMTF
LTLTVCTAGVCTGVFTLSSLETITVPMVLIIANYTMGFLTELFVYCIIGNEFYEEAL
LLPEFIFAGNWNEYANNGMTKDLKFMIHRSQSVSPLHAYGLYNINMDSFAKVLKLSF
SVYTFLSTIKNK
>PstrOR35
MYANAVFGIWPFIFEDYPILKKIYDLYCKFTLTYYAFFITTGYIKFFQLITEFPLNV
TEVMLNVSITFLYSCTFVRAKRMRNPRMLKNIQNIMDYEKKINDSKDEEMMKILEYY
AWQNKIVSQAFIITVIIVTSLFVVHPLGLDPVEKFDSRTNTTRTIKPLPLSSWFPFN
EQVHYLPAYIWHIFDCYVGASFVNNTDIFSFGLITFPLGQIVVLNHILAHFEDYVVK
VQKELGVERDEASFVAFRECILMHKNIIDYIYDMNYEMSMIALMDFLQSSLQLAAIV
LELMFFEVNLFNTVYSFMFVICMLTRLFGYYWYANEIIVESLNIPTSIWNGKWYEEP
LKTQKMMLIMMKKCSRPLTMDIGPFNIMSINTLIGILKATYSYSMLIYRGKQKK
>PstrOR37

MSWSKRIIDIYSYILHFLGFSERQTKFDMFLIYFSALGAILNDCFILYNLKYTAFTI
ENITNFTETAFCFLEITFYSAIAIFKRSDFTALLEMQSLCWDHAAFDRQFSRHTAKI
FKLCLAFSVFFGLLGEITAISSSVLAFLDRGVPFYCYVPDGIVWYFIVFITQAYCSS
YICVFAVSMLIVYATIMFEMHYQLRMLNARFAEMATMGDLRRCVEHHGFLYGYFQRS
RRMFSGVLLMLYVRSIGLSCMELLIAVDKNKPAGLRFKALCFVPLIFLELCLVCVPI
SIVKNESENTPDAISRSDLLLHGDLRVRKNAIFLLKRCQKAFTFKAGDLYELDFNTP
VQIFKSGLSIYTLMKSFEN
>PstrOR38
MFLRDIFPKNEHFRVTMYSCSILGLWPFVFPKNPLYQKMYFRYSKFIYIFTWLFLST
YVVQLIKLLLDEVIFVEEIMRNISITAIQSLSLIRAYALKSKRSKNMINEVLSTEQR
ILGSQNSGVIGIYNEFAKKNNSFITIYMASMVLDTCFFVIYPLCAPPIEIFYPTRNE
TVTKRMLMLSAWFPFDEQKHYMAAYLFQFSCGPITEFYIILSDALTIGLVIYAIGQF
RILNELFANFDKYAIQVQNQLKCSKEEACLTALRECTIMHKKMLSFIDDFNAVMGNI
MVLDFLQSSFQLASIVLQVLTTKVTLINFLLGFQFALSMVMRLLIYYWCANEIIVES
SRISVSIYNSCWYEQPEVVKKSLLLLMQRCNKPCTLEIGSFGIMSLETFISIMRATY
SFITLIYNVNNEK
>PstrOR43
MENEIKRVSMSFSIKLFQYYGGFPQNHEFLNPGKIFYIKFVVKAVLSSFLLIGSILH
LVHSIIVNIFNHVELDVAYSTALLIGYFLMLSLILKMDKIGKFYTELSNFKEYEKPM
DFEETNRRYNKYALMHFIYIMIFVLTSSLFSNIFKIETCRRENQEKGIHEVCGLYLY
TWLPFDIDFTPVRQMYLLWQIISGQFVHSFIGVCAWMVLESSEHVAIRFKHSGYLFT
VAADEEDDQKRRVLLNKAVKYHKNSIRLAELLEDIYSVFMFAHMFMTSVILGYDLYS
FIQTPQLSTFMMCIGWVVGVFLVCQGGQRLQDTSLKVAELVYASNWTNYDISMQKEV
SFVIMRCQKHMSLKAIFIGDVGYMPFLSVLKAAYSYVMLMTNTAN
>PstrOR46
MFFDRLAFRVAGTTDFAKLFTWIPQMLLQFIFFWPKQKTYLKTVLFAIAMVFLIFIN
VGLAVEFSSTFDDLVRFVMHVTYITVTVQGTAKTLVIYINNGELEVILDDIMTKFWP
YDLLETKLKKELKSFYYVIVIIMISLVTFGVLYSSMIMITPWASRKFPFDVSYPNID
YNASPYYELIYLVQIFTIQYFLFCLVLGCDYSFLAICSCVIAQYKLLQNALMAFHTP
LMEEVNRKLRRIGNDGLEGKMYPIHKEYFIRCVNHHHLLLGITKKMNSMFSSIEMVQ
LSCSMTGVCIGLFSLTALEDPPVSSLVIILSFTAIYFNELFVYCAIGNELHYQASFL
PEFVFKSNWNELADKELTRDFMFMLQRSQDIPQLSAYNLYDINMESYIKVFKLSFSF
YTFLTTMKEK
>PstrOR48
MEEKTYFFPIARFFMEITTIWQPNKMTETSRKVYTALAYLFKGNYCIFIASYFIGLI
TTDSEEDRESSLQYLPTMIGIAVRMLFLHTKEIKAIFSGIYRLETKLASEENKKLRN
LFFDEVKYNKNLTKLLIALNSFTNIQMFILSIYLCMKLKMILLAVAWYPFDKHQYIY
LVALHQIFSTSYSTVVYLGLDITIVPLILFATTRLKLLCYKFEHFKQFTDNIGLSPK
DYLSLLIEEHQDIISYVVSVNHSMKWCFIVDFMLKSYTFTQYLYTVLKSFNEDRARL
GLALFAFIVVSVEIWYISYHGNELILASQELSKCIFANNNWYELDIGLQNDLLMIMV
RSERPLSIVVGNFYTIDNNLFLKIMKAGYTFLLFYNV
>PstrOR50
MTNQDSALRKSFKLDEFFLNLNGLLPGEKYKLLYSFYSFGMLLVYIIMIPILGYVEI
FLAEPSRRVNMVDKSFVYLEIFVSVFKHWPFITKPARTKRLLNRWNESIFNTAVEID

EHIVQDSIRYRIFFFQIYFTSGVISVSVLLISSIFSFNDRELKLPIWLPDQVLNNTF
LYVIANIYIITGYTHAFFGHASTDSLLQTYLLYCATQLRLIKFKLKNIHKYFKKVAT
SEDSHKTTIEGLPQKKIYDQIVQCIKVYEAVNGYVQEMENMYSVGIFMQLIVVSLIF
SICAYNLTTVRIYLRVSQERLGS
>PstrOR52
MYHENLNWCLKVYTFLGVHPRKKGIFRTIHYVFNVSCPFVITSLTLIMLHLNRPFNI
EYLGEASTALTTFPHAIMKLTTLFLQQSKIIDLLERTRSQFWIVDDDNVEIKRAKRI
GKVLKNAYFYSVAFFMFTTILKTILTHDLAYRCYQPKWIPKTLLIIYQDYTCCVILF
TLMSFDIMFQTLLFQTQLQFKMLNKKYKHLFDYENLGPTALKSMLKECVDHQSFLID
FVNRIKDTFSLSLLLFVGNIIVSLCMSVYIILSDNSKLNLKIEAVVHTIAGLNEIGL
CYSIPAQMFMDEAAEIRNSIYFSNWYQRPRLAKEIIPLLIREQKPLTITAGGFVMID
LQMFLVACKTILSYSMFLNTITQLQ
>PstrOR53
MRNQQSELRKSLKLLEFLLNVNGLLPGKKFKFFYSIFSYAFVIFFVIMIPVLGYVNI
YLAEPSKRVDYLDKSFVYLEMFVLVFKHWPFITNPNLTKILLDRWNESLFNTQVDVD
KHIIAETLRRRRIWIVRNVLITAISPIIILIQSMIFSSHDELMLPIWLPVDVSKNPVA
FTVTNMYIVIGYMHGFFGYFAVDILVISYIMYCATEMRLIKYKLKNVEKYIEKEATN
NSDKTTSDGLPHKKIYDQIIQCIKVYDAVWKYAQELESTYSFGIFIQFFVASMVLSI
CMYNLSKIKSIIEILYTASFLLPTILVIYYYCDQGSLIIDESTTIGDAIMKSPWYTY
DEKTKHLLITFMERTKRPIKFTVGKLVDVSLETFVLVNNRK
>PstrOR57
MPPLPYDFKDHVEINVNFMYFFGIMRPKLDTKLENFLHNVRVAVLSVFMFGGIMSAE
LANLYYSFGDLEQLMRALFLTLSNLVSIAKFHAVWRNQRRILDLIDAMNVAALRPRS
RRQLAALGRYARLGKALTLGCAGAVTATCAFWAVYPITDDDGPFLPIPAYVPFDVRN
STFRFALVYSFEIVATAVGGQMDLAADCLMAVLLMTFCAQLNVLNDSLVNLQPMETN
RTKSLVKNMNAKLVHCIRQHRLIIEFTETWSGMFTYTIFSQFVVSVFVICATFFEMT
QVPASSIRFFSMALYQMCMFLEIYPICYYGNEVIKESDKLTDSAYHCDWSECPLEFQ
KNLVFFMTRSQRPIKLYAGNFFALSLETFIKILKSSWSFVAVLIQVNNDKID
>PstrOR58
MKYLGFNPPKRGYKRAVYIIFAAFHILCISIVLNALDWLEFFRVFRYNDMKLIMLRA
TMVLFSLVMGQRILLFACTFKKYQVIKDAVKRSSFNFECFGINNINVDSVSNGSRRK
EKLSFSSIWKGAKLSFSESNELESFRLKSMVEMGARCSAILNAAIALCVILSIFWNY
KETHGEFFEDYNPYLNKTSLYRKTQLRFYMPFDSSLEGHDALATCVTLYTRFGILLL
FLPIDSVLPSILAFLIAQTRIVQEAFKYVDKNISPLQSPRNVFLIKEIRIVKCINEL
QEIYRILDMLQNLYSFQTLLQCGLVTFLLCTLGYIAPMVSDGSELFCFGTFLIAVTE
EIFILSYVSNTLTIELQNVAKDLYNIEWDCYPIRVKKLIGFTLRRIQKPASLTACKL
FNIDLAVFIQMLQTAYSFYTVINSTSKKLVA
>PstrOR59
MSSPDRPSRYFDTHRYLFNAFGIWTNKRFIANSLILTVIYAILVVALFIIAPQFCHV
VYMYKARNDVVAFADEFYVSIASIVVVFKDYVLIKDGGAIREMLHTMDEALMRPSES
QRRKFERTMGFWDKLFKCMLAGNYFFCACILVTPLTNRMELGTREVPLVDCYPFYIY
STPVYQFMYVYQSFLLVYLISHTVLFDTFVAGIVSVCSGECDILYENLVGLQSLRDE
SDYERELVKCIKHHEEIRKLVRNIEKRFSLIILEQYFTSLISCCTTMFKLSMAEPMS
MEFFRTLSYQLNIFLQMYLFCWCASEVTEKSNRLPTAVYEALRMESSKSTKTTMCLF

LGKTQKPLTLRILIFDLSMQLYLQLIRSSFSYYTVLMSLNEQ
>PstrOR62
MYGNMMRKFRMVNKMFQTAIKLITHNKRAANVSLKNEMLKSSKTLKHICLTFHFIFS
PIAYNLYMFFQVTETTLPIKIMKALSLNRSYLLKFYHIMIFYIIPIHSITYIPTPVY
TIVYCSRHIKFSLKQVVEEMKNFQRNDDQLKASITERLHSNKYQQKVKKELIYYTKQ
YNAIKRAADYVNHCIKWRFLSIFIVGGLSAAFNLVLILQVDFKSQIFQAGIVLIILA
YNVITAECGEQIQLECESIYTNAQQFNWYNWNKSNCQLLFMLLMQTREPLQLKCYDM
AVMNRVMILAFYQRVYSFISIFGAR
>PstrOR63
MHPLKGYIKPLIVFNAILTAYVTVLILLRLFLSKELVTVESLGVFFQVWVKFFVLTI
KKDEIFKVIQYTQLFWKEDPAGSENSKILASLRKTEKYFLIYILFSTCMFLFKPLLV
QGTTIYYYYKIEQIPFVVSYLIEFYVTLATMSMVIGVNLFICITINLGAGQFSNLNA
KMRQLDLSETQQSGRGLEMCTVELREDIEYHECLISYVRQLDGIFSWLFTLLISIIT
SLLCMNMYVLSQPHNTVVDLIRCGTMVCAFTSEFLLLYGVPAQKLIDEAENVANSVF
YHCKWYLPNIIRLRKAMSFMIFRSQKMVCLSALGFIDINRQTIVAMVKTAYSFFTFL
QTMESPENAQN
>PstrOR65
MVLSEILKLLRSLNDLEKLMDSLFLTVTNLVSIVKFISIVRNERKIMKLLAQIDKRE
FRPKSQHQKNILESNIKAANVIALIVYSGCFVTVLFWVISAVIGDEVSLPVPSYIPF
ELTSTPVFVITFIYESIATLIGGFSNLSADCLMISFVIVISAQFNILNDTIENLEHF
CEEEFMEKRMRIDRTSPEFQNLMNKKLIGCIEHYQCITEFSKTTNDLFSVTIFLQLF
LSIVIISSTLYEISTGSITSQVKTFSMCAYLFCILGEIFPVCYFANEINEGSSRLTT
SAYTCDWVDCSVEFKKNLLTFMTRTQKILRLYAGNFVEISLQMFMKIIKSSWSYWAV
INQVDK
>PstrOR70
MRFEMIELINKRPYYVCTKAMVYTCLLPKKNCPMSIFYLYSTVYRLILTIPYALIIL
NTFLKIKNYQKATGVNFSEELAMCCSYGGMYLTIIIFQVKHKDWLRFIDKVVDHSEF
GIPPTFDEAQRNSNIVTAMMFLYAAIGVTFYALVPLFGIEECRRMNQEKHLKESCSG
LPVWLPFETTNNKQLFILTFYELASSMSICVPASSVAFMAYNSSKILISKIDQFKEL
LLEALKEKDVRVRRDKLRYCVKYHNHILSLGDELKRLVRVTVGSYGIMAALSIGGIG
NQIIQEQSARSIVHFFGFAVAAFTMCHAGQIIATESVSIKEVIYATDWYLCDAKTMK
DIRFMLARSQIPITLEMLPLGWLNYEFFTMIMKTSYSYITLLSKAT
>PaenOR1
NVQNMETLSGLEPHRLPNYSAIFNNIFNTFGVWTEGKRLANSLIITTFYSTTVILLF
IIAPQFCHFIYMY
KARDNVIAFADEFYVSIASLMIVCKDYCLIKHRSEIKKLMKSMDSEIFRPKNTGQYR
RIFKVIMDKYKKL
FWMITFVNVSFCVCVVIGPFFDLIKDGAKLLPLVDCYPFRVTESPVYEMMYVYQILV
CFYLMMHNFPFDL
FILDLIGFCIAECDVLCENMNELKKEESAIEYDRKLINCIKHHEEISRFVRNVERIF
SFPILQQYFCNLI
SSCTTMFKLSIAEPMSVDFFKTLSYQCCLLLQMFLYCWTASTLTEKSQKIPIL
>PaenOR2
MLRFSIFDYFRPNSHTTDYGKSMLWFVKFVLEAIFMWPDTERPELLIISCIILWLAG

IFTWCCVLSYIIV
NIEDFDKAIYGMCLLSLPTMSVIKTPFVFYKFKALKNLLKQIETEFWPYDVVDLETK
KELKEVYSISIGF
MLLFFVIVQIYYGAFLVVPAFLKERVLPLPCSLPFEWTDGIPYALIYIVQFICLELG
TTFGVLGIDILTL
CLCICTSNQYRILQKCFLIYNTVDMRETNDLLRTITKRSMDNYSLEKEYLVRLIDHH
NLLLRFTTKLNEV
LSPLELGHLVISIMGICLGTYVLSMKELSTLHRALALIFFTGFNMQLSIDCTVGAEL
YHQATLLPHCVFH
SNWMSLEDTNLKKDISFVLQSSQRFPELRAYNLYSMDMVTFLKIQRFALSVYTLLSN
MSTIKLNET
>PaenOR3
VVISTMNQFFNFKDIISISLNFLYFFGIMYPEFDTHITTIKYVIRVSIFLGFFFGGI
VIAEIINLYLSIG
DLQQMMKALFLTLTNLVSICKFYAVCKNQKFLLNLVDKINRKEFQPKSKKQAKSLKK
YIRLSKIISISLY
TGCTMTCGFWSIYPYTDDEGPFLPIAAYIPFKTDNPRVFGLVYTYEIIATVIGGYTD
LSVDCLMASLIMV
VCAQLHILNDSLVNLNSMVKEELERNKYEGIGKLNKEDVINGKLIECIKHHRAIMDF
SENVIELFTTSMF
WQFVVSGFVICATFFEMTMVPVMSVRFFSMVLYQYCMLLEIFPICYFGNEVIIESNR
LTNSAYHSDWINY
SFKVRRNLIFFMTRTHRLLKLYAGGFFTMSLDTFIKILKSSWSFVAVLIQIQKT
>PaenOR4
LMYVSCAIRMTVYGFTIGFRNFKDYSKRSLWLPEFMLKLIFMWPAQDAKYRKVSFTA
SILCVLFVQWGLL
SYSYRYFRDYNQTIISVTVLSPLIQTLFKISMLRYHSVSLQNMIEDIGSKFWPCDFF
PEDNKRFRTRYRS
GLLAMIVLAMSALIFTLGHSLLPLLTPNRQLPYRSLYPFDCLMSPFYEVIYILHCFV
CNYLANLSILGYD
FVIISICNNLVTQYTLLKKCFMAYGDIEKVKKYNEKFNQLSISPNLNKNKQDENKLF
LVRLVKHHQLLNR
TAKSTENVVYIIGALQLCTSITGICLSGFMSTRGDVGVMEVIAMGIFLIGHLIQLFM
YCAVGNELFHESS
SLTDNIFASKWYEIEDNRLKRDIIIAMKNAQVPAKITAGKVFPLDYSTCIEVLRLTF
SFYTLLSSLIDK
>PaenOR6
EISRYKFRKIKKMLEYKENPKNPMVCMSVTLNIMRRFLVLPPEHAMLDPGAFFFFRF
VVVSIFGAVPLMS
STTHLMHTIITGEYHLMETDLTYIVSFITSYVAVWAFLLKLRHVAEMYIFLSDFEAF
GKPKDFDVNNENF
NKYAKMHFVYLEIVVWLIVLASNMVKSESCHQDNIRLNLTEVCGLMTNSWLPFDIDY
TPVKEIYLIFQIF
GGHHFYMLAGLMAWLVFETVQHIVVRIRDIKHIMLEALSQEDPLLRKQQFHFAIRYH

NALFGLVDRANEA
FSIFMFTHMVVTSIIMGIGIYTFLHAKSVSTIMVCVGWFWGLFMDCISGQRLQDETS
ELAVAIYDSPWYD
CEEELKRDILFVLHKCQTCQVLCAGAFGIMDRAMFLAVLKLTYSYITLLR
>PaenOR8
RHVVMVNTFVNMTTVNVKIMDLVETFDTEKRYLTLGGFYPISTDSRLKKYFHHFRGT
FNTAISWLEWILL
IIFLYKIFPDIVKISEGLLFYMTQSAFVCKLVNFIIKKNRMFTIENQLQNPLLTEVD
EKEEKILIGFIKE
VKLLAKFFRVLCVGCVLFYSLVPILDRSPDGIRKLPLPIWVPFNTQKYYHFLWFAIM
FSLSIGAWTNSNI
DILTIMLISIATAQFEILKKRFENVVPPNDSTIPESVVKRRLTKCIVHFNEINSFVV
TLEKTFSNGIFIQ
FLCSVLVICLTGFQMLVISIKSIQFVLLITYFNCMLCQIIMYCWYGQLLLNSSDDIT
KACFSSDWFLCSA
KIQKIFITIMERSKCPVTFRAGKFFTLNLKTLMAILRSSYSYFAVLQHVYRNRMHAN
NLD
>PaenOR9
RNMELEIRDDVDVREIININIKFLSFIGAIPPNLQLTKESLLRLIYITITVSLFAGV
TIALIIDLMVSFN
EDIKQIVTSAYISIAALFAFVKSSIVFGHRKRLMTLINTLNRKEYKPKNAHQREILK
GYIKSAKIITITV
LILISMCASFFLLYPLTVEGDLYLPLPSHLPFDTNRSPIFEIMYVGEIIGLLTTSLF
NLGIDSLFATIFM
IICAQLTLLNDSLANLDEDKKNQMNTNLIKCIEQHRSILEFSNEFQSMFSVVLFGQF
AISVLILCTTLFQ
LSMVDKFDLSLVYLLIYQACMITEIFLPCYFGNEVTLKHEQLKNSVYHCEWLDADRS
FKRKMLFFMTKTQ
NPYEIRPGGYVTLSNDTFVKILKSSVSYYTILTKITDKQN
>PaenOR10
MSEMDNIDYRNFFTFNVRVFKICGVWKPDSNSNNKILITLYNSFCISLWILFLFSQF
MFLYNSPGIFDEL
LSTSHITIPEYVCSLMFIVIYRKLDLIKLILDKLNEPMFQPKCEEHLQLAKSLENFY
KTYNYSVTYLAIQ
TSLLFLILPLWQDEQTLPAKAWFPFDWEPSPNFEMIWVFQSVSVCIVTVNVACLTIY
TTGIFMLIGLQCD
YLCITFNNLTQFHVENGILLNRGEKHMQISNPKFSDVMMENLVVCIRHYTEIRKMAL
EIERIHEMGVFIL
FLGGATMISTILFSLSTVDIGSPQSLMLMSDTLCMLVMQFVYCWFGGEITNKSANVF
MTSYNTPWTDCNI
RYRKVLLQFMTMTQNPIEMRVGGLLVMSNAVFTSIVKSSYSVYALLHDIEE
>PaenOR11
MIVCFQQEKTFLPLKSWTPLDLNVDSFYYLTVIFQFSVIGILITCDTSLDCLYYALA
DVACCQLDILKEN

LRNIEPRGNLNVKQELIVCIKYHQEIIQFVEQIESVFSIILFLEFFKSIVNICFIGF
ELTMANFLSLHFF
MGFIYLNGLLMEIFSFCWFGHALILKSTEVEDACYLIKWDECSISVQKIIWMIMMRS
HKPLAVRALFLTI
KISTLTLILKSSYSYATVLRTIYD
>PaenOR12
MKIKRGYLKAFNREKRVLQMFGVYPVKSSQIKYSLLRFIICFAVNSIQLYLMMMLLV
TKDISNFLEAWHF
IIITIIVLFKISFLVLASTDMKDVEDYLNNFDTVDIPHGIVDYVIGEAYFRNRLYLP
QWTTVVSAVTFQL
SACLIFHNLGLVLFISWSPFNLQHPTYYYLTLIFQVVVFFSNGMPNVTIDTMYYILV
DVACCEIDVLMYK
LSNLDPLKNPNETLKDLKEYVVCHQKILRYITLIQRNVYSKLMFLQCIGSIMVICAL
GFQFTMTVNWFLF
IQHATYFSSMIFQIFGYTWFGQKFMGKSQEITQACYMSRWYECDIRIQKMLLIIMTR
TKKPLVLSSYIFD
LTLETFLGVLKSSYSYMALLRTMYNK
>PaenOR13
NKAWCFVPIQKKMYLNFLQSFKTELAIVKSCGVWDYVFKCRYQFLYVLYFIIVNIGL
AMYNLMKFNDMLQ
SNTLETAVAAGFVLPIALMGNIRSLCFFMNRKEFFELLTSMDDEIFRPKNTAQMVMA
QKMLKYYNNFKLG
MYAFSILPSFGCPIGRMIFGESGQKYCEAVITSSRGTAIYLFQAVSLGMISVINVVT
NYFMVGFSLFIAL
QCDQLCYHLEHIDVTKNFKIGEFVQHHRRILRFAENTEKLFSFIYFSFIIMCLLAFC
TTLFMISIIEDRY
SFQCLHLIFYQLSIFIMLFIPCWFSTQVNIKSEKIPLAAYSCAWTENPRSFKHDIII
FMNNSQKPIQFKA
WNLVDLSLETYMAVIKTSFSYYTVLNSLIFEED
>PaenOR14
IDKTVPFSVTLRGMSLLLAYHENYSFLKMALLICSKICTLLIIPVLSMIHLIVVKKE
QVDLSITENISII
ISVTSMAIISLIIPFYSKEWKSLLEKISDFQSMEGFSNRKEIKKTIIFLNEFSIVII
IYCFIGPILYATA
TCFEFKECIKISEERNLNFFCITMSPVWLPFQKTKSAEIAIFAIQFITTAITTMSGG
LSTYITYEFAICT
SLRINCLKTKLFDAINNSLPEEQEKKLAEWIQDHQSIINILQEAKRLIKICHGFGSL
SFALVCASIANQV
IQKSKLVGAVLHILGWLGLMFIHCHGGQTIMCDTESIGETVYDSKWYEANIPTRKTI
LMIIMRCQKPSTW
DALPQGIISYPLYLMVIKSSYSFLTYLSNTN
>PaenOR15
MADYYKDVKWCIKLCNFLGVNPFKENNIIQTLLITYFLVSLMGSIVTAVIFIFSNGD
LSNIRYLIDVTNN

MLKVPNGSFKLLTLLTKKSIIRDLMNETKNRFWESPETEGIKRSERFATFIKNFYFY
CMLLCVLSSLIKP
IIAGEILSTYNHYKPHSVPRYVFAIIGNTSSLISVMSMVGLDTLSFVLLMRVEVQFR
TLNYHLERLFNFD
KQNYVYSPANFSRKLKECVDHQNYLNQFVNRMITTFAAVTLVTFGLTTVSMSLRMYI
LSGDNRLNYKIEA
LFHLIAGINVIGVGYCIPSEAVMNQADKISESIYFSEWYNYPQEAKNVLQILQKETK
PLRITAGGLFVVN
LERFMIIEKTIISYCMFLRTMEVAG
>PaenOR16
RYNFSNNNVSIKENFPGTLLIVSTLKIIKKVELKLINMAKSVKFGYSTNFFHANDII
KTISGLWRPASET
SIFIRILYLAYIVFMYFNGCAYILCEWMMFKQMLKDIPKLLSYIGMCLTHFLGITKL
GIFIFGKNIERIM
NSLQDDRFKYESLNDHGPSAMFQAEQATSSVIAYGTLGMYTFVGGSAHISALIKIFR
EVPGDTFEGTNKT
CYDFMPYFFQIPFSAETKWRCILASTFMDVGLALWAFIISYYDGLFVGLLKCFKIQL
IIAAHVLITIRER
CIRKLGLNKNFEVIHDEDYPELEQEMHKQLGKVIKHLTLLLEVRNDIETLSTFVSLT
QTLCSLFIFASCL
YVASTVEMMSPEFFAQLQFFCCVLIQLSLICWSANEITVAGQNIGTALYSSDWFSCS
KRFKTSMILTMIR
MQRPIILTIGKFTPLTITTLVNVCRGSFSYFAVFKTVK
>PaenOR17
ELLTISNALFMIVNIELLPCYLCFCLIVQAIIFYSVFYQTYSLYMEDMMNSPLYSLP
TSSCSRRVQRLIK
KEIESSDINRIIFHLFATCIIVTLYPFIGDEDKIMYGIFLIKKYLYFGIFNTTLRIL
FYASLHLLTFYTV
ETVVLIVRTMKHLKYHYFVLDEMIRNLEFEINSEMLYIKIVACVRHHVNIKRFAKSL
SFGSVRLGVFLEF
VYFLTVILIVASFYFILDDISPTSNFRFIIITLFMISVSSFSIICGQTLKNASEQIF
FTA
>PaenOR18
MSNFFVTVESVGVFTQIWVKFFVLTTKKDKIMQLFEDMERFWKTDPEGSENSKLLKE
LRRKEKTFLIYIL
FSTCLFLFKPLLVKGTTIYYYYKIEQIPFIISYLIEFYVTLVTMSMVIGVNLFISIT
IVIGAGQFSNLNA
KMRQLDLSKSQYEACRREIKENIDYHDHLITYVRHLDGIFSWLFALLISVITALLCM
NMYVLSQPNNTVV
DMIRCGLMVLAFISEFLLLYGVPAQKLIDEAQEVANSAFYHCEWYLPGIVPLRKSLS
FMILRSQNMGCLS
AMGFLDINRQTIVSMIKTAYSFFTFLQTVETAPEKGGP
>PaenOR19
MNTENVKVIKIMEHPIRILQIPYLFPSATEINDPKANIYFKYLLVNFSSIYYSVGAL

LHMIFNLKRGIAD
HIDKDIGNILSYHGASYFLFRYLLNLKFIIILFKQFSDFETFGKPNNFDERNKSINK
WSKVYLGYQTTIL
VTMLLPAFLYIPQCKQENLEKGLQEVCGLPIPTWLPFRFDYFPVKQIVYLYECYSAF
VVYQTAGMLSYTM
FASCEHLILRLEHVKHQLVDALNEKDVVIRRQKFNKAVQYHQAVIEMGTLLNKCFSP
CMMVHISLTGPFI
GVVGYTFLTNIPLDSTALLVGWLVSTYIVCYGGQRLMEASISVGNIMNRIHWYDLET
DLQRDLILIIIRS
RRPIFLTAGPFGPITYSTVVTILKTSYSYVTLLNQTM
>PaenOR20
LIYGNYSDRLISDMWFPFDYTRSPLLEIIYVYQIVSAFYIGGIYIYVDIICLGLFIF
IELQCDLFSSYIS
KINRLDEYLLKKLIKRYENLLRISTLIQEVFSEILIFQLMVTYCGLCFTIFVILMAE
NFFELFSYLVMMI
LVFIMFLFPCWFATCVKVKSERIPIEIYSMPWYDCDSIKLKKDIVRFIHMTQKPIEY
SAYGLVPISNDTF
IQIIRSTFSFYTFLDFLKKKYSE
>PaenOR21
SSIVHLRFMSKFPIKKNCHFNEKMFKPDFKKAFDKEKSILQFFGVYPISDPKVNFER
IRCFISYSLNVIN
VSLMMMLLLVNDIENFIDASDFIVITFTFLWKFTLFVLGKGMDLIEDHINLIDVSSI
PAGIMEHVRKEEE
GRNWIYFPQRYVVQGAALFLVISCFLHQSDRRLILISWVPFDLQDLIYYYLTIFFQT
IAFLASGVPNGCI
DIMYYILVDIASCQMDILIYNLVNLDSSKGNVELYQELNKIIINHIEIIRFAKNLQN
VYSNIIFVQCIGS
VMVFCILGFELSVTIPFPSEKFFLRALYIFSMIFQIFCYTWFGQKLMVKSEEVAKAC
YLSKWNQFCPEIQ
KSLLIVMTRCKKPIILNAGIFNLTLETFTMILRTSYSYMALLGTVYGQYITKK
>PaenOR22
NGYVANICFMANVKAYYLFKERKEIKTIFEDLNNAHFQPINEEAKLIAEKSLTFFRK
VKVALTAVSLMSY
LCAMTIPIFSSKNKSLPIIAWYPFNISATPVHQLIYLHQAVAVYFESMVNIENDVLA
SGLFTFIGIQCDF
LCYKLYNGTEANMKNYIEDHQRVQNFAKRCENVFSKIYFFQYFATTSGLCMTLFLLT
VVAPNTFEFFFLV
VYQMAVFNLLLVPSWFSSEVKRKSENIPVAAYCYPWVDAKQTVKRDLLIFLRSTQKP
MQLKALGFFNLSL
GVFLKVGNYKKIIMLIVEL
>PaenOR23
VNLNRSNNRKNMTEKTLMFDYLGWYSNDIIVLRLAGLYCVDENGELIKNQLIFTLFV
NCFLVLLGNVAQT
AYLFLQKQDIVTIATSGYVVAISWLCSINFYYLFKHRRLLASLFADLNNDVFQPTNK

QSIEVVKRSYKIY
KFVKNTLVVICTTAVTASVITPLLRKNEAIEVRFAVKLWYPFDISVSPALEFVYVLN
AFLNIYYTLMNIY
IQIMVAAFTTFIGSQCDLLCSNMDAATDIKSIKDFIFHHRSIVDFANRTEAVISEVY
MFIFAASNISICM
TLFLLQVVERFSYEFFFLISYSFGITFQLFLPCWFSSEMKRKSEKLPSFVYSLPWHD
APNNLKKDVYLLL
VSSQEYIIILTVMNLFPLSVDTFIKLLKSSFSYYTVLSSMNEAGN
>PaenOR24
AGLYCVESILSYLGKRYANLRGTMTKKKMLMFDYLDWYSNDIIVLRLAGLYCVDENG
EMKKSQFIYAIII
NCFLVLIGNVGQTLYLFAQKQDIVTVATTGYVIAITWLCTFNFYYFLKHRKSLARLF
ADLNNDIFQPTNE
KSVEIVKRSYQLYKIVKNGLVFICITAVVASVTTPILRNSDDIEAKFPLKVWYPFDI
SVSPMFEIVYLHN
CFLNIYYTLINIYLQIMVAAFTTFIGAQCDLLCHNMDAATHITSIKKFILHHRSIVN
FANRTEAVISDVY
FFIFAGSNISICMTLFLLQVVESFSYEFFYLMFYSFGITFQLFLPCWFSSEMKRKSE
KLPFFVYSLPWHD
APNHVKRDVYLLLVSSQKYIILSVMNLFPLSIDTFIKLLKSSFSYY
>PaenOR25
MRDDPLGIILKLVRLGQKPNRIWIIEYISLFIFIVIEFHILGGFYLVTDNVNFMEYL
SMISLMQFECTGF
HLIGVYLTQSQRTLTTLRDISKNLDLLSPSFEIKFLKKAQFLMAFLMGLLIFNVCMS
ISCTPFLIDDGFV
DVTYYKLKRFYGDGPDFVYVFFAVVFLIAGSFASMVPVMHFWYSTFHIQFQLNWLSS
YVKQNLHEKLNDR
SMENEKCQKLINKYLINVVKYRLSFKIAFDSVLNNGAMVILLLLSLWINVIVLYNLI
NRTHLGTSFKMYM
ILILSIILVVMATVCGQGIIDESLTLRDALYECPWMYWNLKNRKILLIIMTNVMEPW
EISFYDLFALNHQ
TLTKGAKLVYSLTAVVSKFK
>PaenOR26
LINQSKTFENMEDSYVEDFFVVNRWILKFGCLWKPDSQNNTIQSLYTIYVIATFLFV
NMFFTFTEFLSIL
DVYGNEYDLIKNISFALTHFMGAVKVVFFYLYGDTLKSIMNTLQSSELHYEYCCADD
LFFPGKTSKLYKA
VGIKYTIIFFIMAHATITSSYLPPLIAAIKSEANNTEKMLPDKLPYYSWMPFKFDTP
CTYLIALGYQAIP
MFSYVYSIVGMDTLFMNIMNCISMNLEIVQGALISIFPRAVAKTNGPLMTPDELENT
KTLATHLRAEMKK
ISKHLQIIYKACDDLEDVHKYLTLAQTTATLFILCSCLYLVSTTPIGSKQFFAEIVY
MIAMGFQLTLYCW
FGNEITIKANNLPFYIWQCDWLTADKDFKMSMILSMARAKRPLHLTAGKFAPLTLPT

FVSILKVSYSFYA
VIKNTE

>PmacOR1
METLSGLEPHRLPNYSAIFNNIFNTFGVWTEGKRLANSLIITTFYSTTVILLFIITP
QFCHFIYMYKARD
NVIAFADEFYVSIASLMIVCKDYCLIKHRLEIKELMKSMDSEIFRPKNTGQYRRIFK
IIMDEYKKLFWMI
TFVNVSFCVCVVIGPFFDLIKDGAKLLPLVDCYPFKVNESPVYEIMYVYQILVCFYL
MMHNFPFDLFILD
LIGFCIAECDVLCENMSELKKEESAIEYDRKLINCIKHHEEISRFVRNVERIFSFPI
LQQYFCNLISSCT
TMFKLSIAEPMSVDFFKTLSYQCCLLLQMFLYCWTASTLTEKVC
>PmacOR2
MILRFSIFDYFRPNSHTTDYGKSMLWFVKFLLEAIFIWPDTERPELLIISCIIIWLA
GIFSWWCVFSYII
VNIEDFNKAVYGMCLLSIPTMIVMKMPFVLFKFKALKNLLKQIETEFWPYDVVDSET
KKELKEVYSISIG
FMFFCFFIVQIYHGATLVIPVFLEERVLPLPCSLPFEWTDGIPYALIYIVHFICLEL
GITFGVLGLDILI
LCLCICTSNQYRILRKCFLIYNTVDMRETNDLLRTITKRSMDNYSLEKEYLVRLIDH
HNLLLRFTTNLNE
VLSPLELGQLVISIMALCLGTYVLSMEELSTFHRTFAFIYLIGFNLQLVIDCTVGAE
IYHQATFLPHCVF
HSNWMSLEDTNLKKDILFVLQSSQRFPQLMAYNLYSMDMVTFLKIQKFTLSVYTLLS
NMSAIKQNVT
>PmacOR3
VVISRMNQFFNFKDIVSISLNFLYFFGIMYPEFDTPITTIKYVIRVSIFLGFFFGGI
VIAEIINLYLSIG
DLQKMMKALFLTLTNLVSICKFYAVCKNQKFLLNLVDKINRPEFQPKSKKQGESLEK
YIRLSKIISISLY
TGCTMTCGFWSIYPYTEDEGPFLPIAAYIPFGTENPRVFGLVYTYEIVATVIGGYTD
LSVDSLIASLIMV
VCAQLHILNDSLVNLNSMVKDELERKKFEGIGSLSEEDVINEKLIECIKHHRAIMDF
SENVIELFTTSMF
WQFVVSGFVICATFFEMTMVPVMSVRFFSMVLYQYCMLLEIFPICYFGNEVIIESNK
LTNSAYHSDWINY
SLKVRRNLIFFMTRTQRLLKLYAGGFFTMSLDTFIKILKSSWSFVAVLIQIQKT
>PmacOR4
LIRMTVYGFTIGFKNFKDYSKRSLWLPELMLKLIFIWPAQDAKYRKLSFTASILCVL
FIQWGLLSYSYRY
FRDYNQTIISVTVLSPLIQTLFKISMLRYHSVSLQNMIEDIAVKFWPCDFFPKDNNR
FKMRYRGGLLAII
VLAMSALIFTLGHSLFPLMTPNRQLPYRSLYPFDCLKSPFYEVIYILHCFVCTYLAN

LSILGYDFVIISI
CNNLVTQYIILKKCFMAYGDIEKVKKYNEKFNQLSISPNLGKNKQDENKLFLVRLVK
HHQLLTRTAKSTE
NVVYIIGALQLCTSITGICLSGFMSTRGDVGVMEVIALGIFLIGHLIQLFMYCAVGN
ELFQESSSLSDNI
FASKWYEIEDNRLKKDIIIAMKNAQVPAKITAGKVFPLDYSTCIEVLRLTFSFYTLL
SSLIDK
>PmacOR5
MEGNVKVNSLNGYPDNFFYTNSVCLKLVGMWIPSKEYSLLFRIIYGIYVTLIYSEGF
VFIICELLIFRET
MKKVSNFITYIEMLFTHIVGIIKYFVLILGRHKIRNLMNTLQDDKYFYEPINGISPG
KIFSNGKETNAKI
SKLTFVMYICVGVSAHISSELILNNEIKGQSFENTNKTCADYFPYFFKIPFDVTMKW
RCELALALMDMGL
IFHAAIIACYDGVFVALLNCLRCQLSIVCHVFRTLRPRSLKSVGLSETHMELLDTQN
PILEKEMYKQLSH
ATEHLKILLRVGEEIESMFTFVTLCQTLASLLIFAACLYMASSVPMNSPEFFSQMEY
FSCVLLQLMLFCY
FGNEITSASEEIRTALFDCDWLNSSKRLKQSIILTMIRMQRPLYLTIGKFSPLTLST
IVAVCRGSFSYFA
LFKSIQ
>PmacOR6
YKFRKIKKMLEYKENPKNPMVCMSVTLNIMRRFLVLPPEHAMLEPGAFFYFRFIVVS
IFGSIPLMSSITH
LMHTIITGEYHLIETDLTYIVSFITSYVAVWAFLLRFRHVAEMYIFLSDFEAFGKPK
DFDVNNENFNKYA
KMHFVYLEIVVWLIVLASNMVKSESCHQDNIRLNLTEVCGLMTNSWLPFDIDYTPVK
EIYLILQVFGGHH
FYMLAGLMAWLVFETVQHIVVRIRDIKHIMLEALSQEDPLLRKQQFHFAIKYHNALF
GLVDRANEAFSIF
MFTHMVVTSIIMGIGIYTFLHAKSVSTIMVCVGWFWGLFMDCISGQRLQDETSELAV
AIYDSPWYDCEEE
LKRDILFVLHKCQTCQVLCAGAFGIMDRAMFLAVLKLTYSYITLLR
>PmacOR8
TFQILAKNHWDINMTVDLVEAFDTEKRHLSLGGFYPISNYSTLNKCSYYIRGTFNTA
ISWLEWILMIVFS
LTKFPDIIKVSECLLICMTQSAFVCKLVNFITQKNRMLTIENQLKNSLFTDVDEDEE
KILIGYINDVKLL
AKIFRLLCVSVILFCGLHPIFDRSSDEIRELPLPMWVPFNTEEYYSLLWLATLLSLF
IGAWTNSNIDILT
IMLISIATAQFEILKKRFKNIVSPNDSTIPDTIVKSRLTKCIVHFNDINRFVVTLEK
TFSKGIFIQFLCS
VLVICLTGFQMLVISFKSIQFVLLITYFNCMMCQIIMYCWYGHLLMESSDNVTNACY
STDWFRCNAKNQK

IFITIMERSKYPVKLRAGSFFILNLKTLMAILRSSYSYFALLQHVYRNRMHANNMD
>PmacOR9
FEIDVGVREIIDLNIKFLTFIGAIPPNLQLLTKESLLRLIYITITVSLFAGVTIALI
IDLKVSFNEDIQQ
IATSAYISIATVFAFVKGLVVFGHRKRLMTLINTLNRDEYKPKNAHQREILKSYIKS
AKIITVTVLILIS
MCASFFLIYPLTVEGDLYLPLPSHLPFDTNRSPIFEIMYVGESIGLLTTSLFNLGID
SLFATIFMIICAQ
LTLLNDSLTNLVEDTKNQMNTNLIKCIKHHRCILEFSNEFQSMFSAVLFGQFAISVL
ILCTTLFELSMVD
KFDLSLVYLLIYQTCMITEIFLPCYFGNEVTLKHEQLKNSVYHCEWLDADGSFKRKM
LFFMTKTQNPYEI
RPAGYVTLSNETFVKILKSSVSYYTILNTMTVKQD
>PmacOR10
MSEMDNIDYRKFFTFNVRVFKICGVWKPDSNSNNKILITLYNSFCISLWTSFLFSQF
MFLYNSPSIFDEL
LSTSHITIPEYVCSLMFIVIYRKLDLIKLILDKLNEPMFQPKCEEHLQLAKSLEHFY
KTYNYSVTYLAIQ
TSLLFLIIPLWQDEQTLPAKAWFPFEWEPSPNFEMIWVFQSVSVCIVTVNVACLTIY
TTGIFMLIGLQCD
YLCITFNNLTQFHVENGILLNRGEKHLQISNPKFSDVMLENLVVCIRHYTEIRKMAL
EIERIHEMGVFIL
FLGGATMISTILFSLSTVEIGSPQSLMLMSDTMCMLVMQFVYCWFGGEITNKSANVF
MTSYNTPWTDCNI
RYRKVLLQFMTMTQNPIEMRVGGLLVMSNAVFTSIVKSSYSVYALLHDIEE
>PmacOR11
KRINSRSTFIANMISLDFVKAFKHERFVLSVFGLYPGEKTDSVGKFSIQITYVTAAT
IQLSLASIQMCVS
KDFTALVDCCIFFISITNFILKAIVLYFKKDVLESVTAYLENIDESKVPAKLLDMLK
KREIIRGKFGTIY
RLAIFLYISLQVMIVCFQQENTFLPLKSWTPLDLNVATFYYLTVIFQFSVIGILITC
DTSLDCLYYALAD
VACCQLDILNENLRSIDPRGNLNVKRELIGCIKYHQEIIQFVEQIESVFSIILFLEF
FKSIVNICFIGFQ
LTMANFLSLHFFMGFIYLNGLLMEIFSFCWFGHALILKSTEVEDACYLIKWNECSIS
VQKIIWMIMMRSH
KPLAVRALFLTIKMSTLTLILKSSYSYATVLRTMYD
>PmacOR12
MKIKRGYLKAFNREKRVLQMFGVYPVKSSQIKYSLLRFIICFAVNSIQLYLMIMLLV
TKDISNFLEAWHF
IIITIIVLFKISFLVLASTDMEDVEDYLNNFDTVDIPHGIVNYVIGEAYFRNRLYLP
QWTTVVSAVTFQF
SACLIFHNLGLVLFISWSPFNLQQPTYYYITLIFQVVVFFSNGMPNVTIDTMYYILV
DVACCEIDVLMYK

LSNLDPLKNPNETLKELKEYVVCHQKILRYITLIQRNVYSKLMFLQCIGSIMVICVL
GFQFTMTVNWFLF
IQHATYFSSMIFQIFGYTWFGQKFMGKSQEITQACYMSRWYECDIRIQKMLLNIMTR
TKKPLVLSSYIFD
LTLETFIGVLKSSYSYMALLRTMYTK
>PmacOR13
MYLNFLQSFKTELAIVKSCGVWDYVFKCRYQFLYVLYFIIVNIGLAMYNLMKFNDML
QSNTLETAVAAGF
VLPIALMGNIRSLCFFMNRKEFFELLTSMDDEIFRPKNTAQMVMAQKMLKYYNNFKL
GMYAFSILPSFGC
PIGRIIFGESGQKYCEAVITSSRGTAIYLFQAVSLGMISVINVVTNYFMVGFSLFIA
LQCDQLCHHLEHI
DVTKNFKIKEFVQHHRRILRFAENTEKLFSFIYFSFIIMCLLAFCTTLFMISIIEDR
YSFQCLHLIFYQL
SIFIMLFIPCWFATQVNIKSEKIPLAAYSCAWTENPRSFKNDLIIFMNNSQKPIQFK
AWNLVDLSLETYM
AVIKTSFSYYTVLNSLIFEED
>PmacOR14
KISDFHSMEGFLNRKEIKKTIVFLNEFSIVIIIYCIIGPILYAIAACFEFKECIKIS
QERNLNFFCITMS
PVWLPFQKTKSAEIAVFALQFITVVITTISGGLSSYITYEFAICASLRINCLKKKLV
DAINNSSPEEQEN
KLAKWIQDHQSIINILQEAKRLIKICQGFGSLCFALVCASIANQVIQKSKLVGAVLH
ILGWLGLMFLHCH
GGQTIMCDTESIGETAYDSKWYEANIPTRKNILIIIMRCQKPSTLDALPQGIISYPL
YLMVIKSSYSFLA
YLSNTN
>PmacOR15
FDRYNMADYYKGVKWCIKLCNFLGVNPFKENNIIQTLLITYNLVSLMGSISTTVMFI
FSNGDLSNIRYLI
DATNNMLKVPNGSLKLLTLLTKKSAIRDFINETKNRFWESPETEEIKRSERFATFVM
KFYFYCMLLCVLS
SLIKPIIAGEILSTYNHYKPHSVPRYVFAIIGNTSSIICVMSMVGLDTLIFVLLMRV
EVQFRILNYHLEN
LFNFDEQNNVYSPANFSQKLKKCVDHQNYLNEFVNRMITTFSVVTLVTFGLTIVSMS
LRMYILSGDNLLN
YKIEALFHLIAGNAVIGIGYCIPSEAVMNQADKISESIYFSNWYNYPQETKYVLQIL
QKETKPLRITAGG
LFVINLERFMIIEKTIISYCMFLRTMEIAK
>PmacOR16
NFSGTLLIVSTLKITKKVELKLINMAKSGKFGYSTNFFHANDIIKTISGLWRPPSDT
SIIIRILYLAYIV
FMYFNGCAYILCEWMMFKQMLKDIPKLLSYIGMCLTHFLGITKLGIFIFGKNIERIM
NSLQDDRFKYESL

NDHGPSTMFQAEQATSSVIAYGTLGMYTFVGGSAHISALIKIFREVPGDTFEGTNKT
CYDFMPYFFQIPF
SAETKWRCILASTFMDVGLALWAFIISYYDGLFVGLLKCFKIQLIIAAHVLITIRER
CIRKLGLNENFEV
IHDEDYPELEQEMHKQLGKVIEHLTLLLEVRNDIETLSTFVSLTQTLCSLFIFASCL
YVASTVEMMSPEF
FAQLQFFCCVLIQLSLICWSANEITVAGQNIGTALYSSDWFSCSKRFKTSMILTMIR
MQRPIILTIGKFT
PLTITTLVNVCRGSFSYFAVFKTVK
>PmacOR17
FSETSNLKVLGKYIVLHKNHDHLKSLINDTELFWNVENFPFKEEKDSATSYLRHFKF
VFIYYTSFGIFAS
LFFTLNPILSGQRILPLTSYVPDYPPYAFLFMVEECAYLVTFFLIIPFDTIVGSLTV
LTAVQWTLLNRRF
QEILNSEVDTPEGMFLLQRELKKCVDHHNFLIRFVSRLNDALFFALAPYVLIIVVSN
CAELFIISLSPPT
ALLIKCVSYVIALLNEYIMFYCIPGQKLTLEAEKTVKYIFASKWYENSSNFKKQLIN
ITTSISDRQIGIS
IAKIMFLNFENMLKSFRMIVSYYMFLKTLEMRRNGGAA
>PmacOR18
MSMIYPKQGEYRISYEPLSTLKKCIQLHDFKGTKKYFWHAAALLKVFILLGRTVYAG
QTLNQPKRLAEIV
ATYPVRFMAITKIYMLYFDRKRVNYFYNTVSQEFWDFHIAGPELEKQIKKRFFIINF
AVACQFVAAMLVV
TLFLIFPLVDMPEGKRPLPNIIWTPFDTDPSPLYEIIYVIMIWNLTLSVLGNAFYDV
LFTYSLQHLFVQF
MLLKKLIKNITKGILEETSDCDKFNSEYFQNKVYERFRICAEHHAKLLKFGKNLKMF
SSRALLPQLIMSY
AVLVINGYILSIDHKDVMKTTGLLNLTGSCLVQLAVFSLQGSEIKFQSLSLLDAISN
SEWYLFRAPVKRA
FTFMMLNSKVPLTVSAGGMANVDNEILLEIVQKAFSAITLLRALTAEAEMKG
>PmacOR21
MLEWNWYSKRSVENFLLIEMDVKMTENDDPFLDVRKLIYLDVLENRWFKAVLISFTF
AHIISALKFLSQV
EGQAAICNTTLQLPFIINNFLILLIRIHYCNAVYQYCYSTRYCDDKLKKKIAKENRD
AVFYLKMWMGILQ
VCVAVATINFLTGNYKILRIDNFGVFGEKFKYLSNFITIVYILGTMWLHSIILNNFT
YIVLHCKIQFLLI
IEQIENLKRFKNGDISKENEGYQQAVRVTLKKAVIHHIQIKQMIKYMSHTNKTMVVF
LSFEGFLIMIGSL
WIVIVEGKTAFILAFTVVTITTTTLYRFCTRGETLISSSMDFYITLCNTDWIHWNQS
NAKSLLILLQATA
TPIAVSFFGIITVDCNLIKQFYKWSHQAISLIVKFQRT
>PmacOR22

MNTENVKVIKIMEHPIRILQIPYLFPSATEINDPKANIYFKYLLVNFSSIYYSVGAL
LHMIFNLKRGIAD
HIDKDIGNILSYHGASYFLFRYLLNLKFIIILFKQFSDFETFGKPNNFDERNKSINK
WSRVYLCYQTTIL
VTMLLPAFLYIPQCKQENLEKGLQEVCGLPIPTWLPFRFDYFPVKQIVYLYECYSAF
VVYQTAGMLSYTM
FASCEHLILRLEHVKHQLVNALNEKDVVIRRQKFNKAVQYHQAVIQMGTLLNKCFSP
CMMVHISLTGPFI
GVVGYTFLTNIPLDSTALLVGWLVSTYIVCYGGQRLMEASISVGNIMNRIHWYDLET
DLQRDLILIIIRS
RRPIFLTAGPFGPITYSTVVTILKTSYSYVTLLNQTM

>CbowOR1
MIGFLKRQTLVDKLKIHVTFFFILILDVLILVKISTTHNKTLEDIMTSYEAVGSYLQ
MTTKILTLIIYNG
DLKQILAMTNQFWKYDKFGPVISNKQQKYPRMMPSFITAYFFFCICTLTVLMLKPVL
FHELPRSCCIPEG
EVWFYVVSAIQNETLFYCTFTVFAFDAMFALLYTEAAMQFCLLNEAFSRMKNHGDLK
ECVDYHVFLYNFV
KKLNDVYWMFLLVQSFDCLSETCFQLLTMVHTQENLTLRVKAVLYAIALYMQLSFFC
FPVGFLQDESQAS
STAISACPWYLKDAKFKRSVFIVMIRAQKKISVRAGGFFEMDRQAFIYLCKSSFSVY
TLLKSIN
>CbowORco
MMKFKVSGLVADLMPNIRLIQASGHFMFNYHADNSGALHALRLGYSCMHLVFCLFQF
GCTFGNLVVERDN
VNDLAANTITVLFFTHCITKFVYFAVRSKLFYRTLGIWNQANSHPLFVESNNRYHAL
ALKKMRTLLVCVM
ATTVLSASAWTGITFVGDSIHHIKDPDNENETIIEEIPRLLVKSWYPWDAMSGTAYY
ASLIFQIYYVFFS
LAHANLMDSLFCSWLIFACEQLQHLKEIMKPLMELSASLDTYVPKSADLFRAPSANS
QDNLIENDYNAKN
EEINLKGIYNTRQELGINFRSGALQTFGQGGGGVGPNGLSKKQELMVRSAIKYWVER
HKHVVRLVTAIGD
AYGVALLLHMLTSTVMLTLLAYQATQIGGVNKYAATVIGYLVYSLAQVFHFCIFGNR
LIEESSSVMEAAY
SCHWYDGSEEAKTFVQIVCQQCQKAMSISGAKFFTISLDLFASVLGAVVTYFMVLVQ
LK
>CbowOR3
MATITNSLFVILEIGICIIKFLPFKNDPKKIRKTLFALNQDMFNRATESQRRFIEET
EAACRNIFAIFMT
FCLLSLFSWPIKVLFYEQRRFPIDVWLPFDPFENVSIYLGVFAYLFIATGNAPIGNA
AIDTLIAGLIIHA
ACQFRILKDNLRCLSQRADEKLNGLPQELKEMKRNEIVYRNIRECILHYDAIYDFVK

EVEKTFSVVIFSQ
FAVSILVICISCFQLSIAEPLTITFFAMVIYVVSLLLEIFLYCYYGTVLYEESNTLI
AAIFDSEWYDLDE
KSKKALFILMERAKRPMMLTTGKLLSVSLETWTMIIRRSYSLLAVLKNHQ
>CbowOR4
MLFYVCGFAVVICEYMMFKESIKDIGKFVSHIGMVLTHLAGIVKFCLLTIGHGKILK
LMHVLQNKDYQYC
SLEDSKPGEVLRKGQTVNNVIAYSTFVMYTLVGITGHISSVRNLNEQIKGDNFEGTN
KTCYDFLPYMFYI
PIPSETKWQCEMVFNLMDIGFALHAFVIAAHDGIFAGLLICLKSQLLIVCDVYKTIR
QRSLKNMHLPENY
TITNDMENPALENEMYRLLVHSMEHLKILLWVRDELEYIFTMVVLTQTVASLFILAS
NFYVASTILTASL
EFFAKLEYTFCIFFQLSLICWFGDDITRASDLIKLSLYESDWLSSSPRFKHAMVLTM
IRMQRPVFLSIGK
FTPITLSTLVAVCRGSFSYFALFKSIQK
>CbowOR5
MIKMIQIRFHTLGFLACMKIFMLSFGQLLSKTLPLKCYSAKWIPFHVMWFYQSFLTS
VCIIMPIIAMDLL
LMTFISLTHIQFKMLNLEIDRVFRRSERAKKSEIARLVDHHNFLIDFSNRINNTFST
MLLAYIFVFVISM
CVEMYKSSANPSFSVFMNAVTYLSAAVFGLIFLFCIPGQNLTDEANNIPNAVYFTDW
YRDSKQSTSVLMM
ISNGQRDISIKAGEVIKINLATSLSTIKTLLSYFMFLRTVVLDE
>CbowOR6
MYTIKKSQPFYSSLRTLRFFLVYREFVQKSTFMLLSFFSSFMSFSAFLVFVCGCILH
AVMSIRENIGGDI
SEDLSVSIGGLAMMVNVAMFKYHQDKWSNFFKDVTNFEKFGKPTDFDATKDRANLLS
TLYMIYCITGTIV
YSCVGVIESSCDELSEETKQKVICGTLAPIWLPFEDVSLTVRNTILLVQYVLANYII
TPSAVICFLPFET
TELLICHINFLKDKLLKVFGNEDGMIRNDKLRFCVAYHTHILGMADQLKYVVKFSVG
HMSLVCALVFGCI
GNQIFRAKPVGAVIFLLGYMVSLFLLCYAGQRIMNESLSIVDVIYNSKWYKGNTQIK
KNVRFMMARCQIP
VTLDAWPFGIFSFPLFMMIVKTSYSYLTLLRQST
>CbowOR7
MISPTSYLIKLIIFKSKSVHVLEMLSFLELTEFNNYPKGLSGIVERTVKFSRYLGYA
YQFMCCLVITLYS
TIPLFTKADLPIRFSHDVGKLKPAVYIFQVIGLSSAASNNSCLDVLAMSLMGICSAQ
IDILNKKLITLGK
NEDEDETDGSNNSYLRLKKCAKHHVEIIRFQKALERVFSSIFLAQFATSVMVICNIG
FQLVHVQPASVQF
ALMLFYFIAMNTQLVMYCWYGNEIIVKVYHCLSILSSAIRDACYKFEWFDSNMETKK

LLLIIMEHSKKHL
YLTAGKISVLSLESFTSVMRTSYSYFALLQTLYRNNQD
>CbowOR9
MLTFSLIHITSLVQVSETLSFNLTQLAYLCKLLNFQIHSKRLLELEDFLRKTTLTNV
TVEEEAIIRNTMK
GSRRLATVYRSLCVIIVFLYALFPLIDENSGDEKKLPLPMWFPFDTNNHFGKVWFFE
IFSIAIGAWTNSN
LDVICVTMITLTTCQFNIMNSRLSNLRKSTDDVEEEDTVQKALKECVIHYNDIISFQ
ILVETTFSLSIFG
QFVFSVLVICMTGFQMLVISFKSVQFVLLLSYLLGQTCQIVMYCWYGQSILDSSEAI
NDACYSSEWFNCS
KETQKMFLIIMERSKRPVKMRAGKFFFLNLDTLMSILKSSYSYFAVLRHIYSSKFT
>CbowOR10
MMVKQTKNEAKRFFRYIGMLFTPTQALGCYVLLRIKQHGIEKVREELLDEQFHYKSC
GSFRPGKIFNDAK
SFCDKFVVITIIILYSLVVASAHISAYVTLNLAFEGEYFPANITCYDFMPNYFVIPFP
TPTKSSCKNALTY
MDVSLNVYATLLASYDTTFCSVLICFKTKLQILSGAMRSMRERVTTEMNLPLNSSLE
LDDPEVEAKLYEE
IKQCARHLESLLSVCKQIEDIFKYGTLMQIVNALVIISSCMFVLSITPQSDPDFFVM
IHYIIALFVQLFT
VCYFGNEITEVADELNNSLYQSNWLSCSKRHKQCMIIMMSRMQKKIHVMIGKFSPLT
MNMFVAVVKGALS
YCAVFRAVDNAEI
>CbowOR11
MGAVKVLFFYFRGDKLIKIMATLESTDLHYEKCQKRKFFPGSISTNYKKVGIKYTLL
FFMLAHATLISSY
IPPTIAAIQSELDNPGKSLPDRLPYYSWMPFKFDTSTTYLIALGYQAIPMFSYAYSI
VGMDTLFMNIMNC
IGMNLEIIQGAFLSLRERAADKIAGPLMTQDGLHNSHELKTALNREMKKVCRHLQII
YRLCEDLENVHTF
LTLAQTVATLFILCSCLYLVSTTPASSKQFLSEIVYMVAMGFQLILYCWFGNEVTLK
ADMIPFFIWQSDW
ISADREFKHAMIFTMIRAKRQLHLTAGKFAPLTLTTFIAIIKASYSFYAVIKNTST
>CbowOR13
MDYGYPKNFFEANDVVKRISGIMLLQGKEDNIFFKWYQIIYIIFVYSSTVVFTVGQY
IMTKNSVNKISNL
VSSLGVLLTTHVGHFKFWLLLSKKKELENLKNDIEGENYQYATIGNSNPGLLLTNEK
KFCTVCTYVYLAG
CYLIGIFGNITTVVRLNGALTGNNTFESINMTCNDFASFTFYIPFFTANKWQCIISS
TVMYSGMAFESGI
HAACDLLFFAMIHCLKIQLRIIADVFRTIRRRSLLKLNVPEDYTVLHDEENSALEEE
LYRQLSHSTEHLN
ILLRVTQEIEHLFTYVLLAQTLSSLLIVASFLYLTSTISINDADFFLQMQFLLVILI

QLALLCWSGNEIT
EGFQLIKTALYESDWLSCSHRFKRSMILTMIRLQRPVLLTLGGFSPLTLATLVGVCQ
GSFSYFTLFKSFQ
>CbowOR14
MATEFQKAFETEKWILSLFGFYPQWNPEPLWKHARRVFCIAVTLTYIISMCTGPFLE
NSVMILCTVMGLS
KMVQLLTSKRQFREIEHYISNMKPSIIRRSSLIGAFRVSVVTLVGFLGIMPLSMKNQ
RMLPYKSWLPYSV
EGASPYYSTFIFEVISIVMAAFTNSTIDMMYYCLVDICCAELDVLKLNIIEIDMSDH
VDIVEDELKKIVI
HHHKIIRLVGIIQEIFSSVVFVQCMASVLVICFLGFQLIYVDKLPSVKALIELSFIA
CMLIQIFCYCWFG
HNITMKSSEVGDICYHTKWFESDLMIRKIILIIMERCKKPVELRAKIFTLNLQTLLA
ILRSSYSYMAILR
TLYTDE
>CbowOR15
MKEVHFKNTVLNFYEFYNTDFKLLKFFGIWIPDSSNSKFHKIYFIVINFVFCAIFNL
AQVSNLLHEINNL
KNLAACGYVVAIACMANVRSYYFLKNREEFLYLIRSLNDSHFQPESEDQICSAKKSL
RFYSKVKMIVSIL
CTITVFISMSTPVFYKKNELNLPFASWYPFDVSSYPIYQIAYVHQCISVIYVTSINT
YVDIIMAGFNTFI
GIQCDLLCSRLYNISKDHSSEENETTLLDCIRHHKLIVRFANNTEILFNRIYLGQFI
ACTSALCMALFLL
TLHQESRFESSFLVFYLTAIFSLLFIPCWFSSEMQGKSENIPEAAYSCNWVTASKLF
KKDLIFFILRAQK
PLKFYAVGFFQISVETFVLIVRSSFSYYTVLNNMIMKEG
>CbowOR17
MNIEHIKEIEVLENSLKFLRIFFLFPRKSEINDPKKNVYWKFILLSLSTAYFSIGAA
IHLVVNVRSGAFV
NVDKDVGTIISYYGALYFISRYLGNIKYIIILYKQFSDFKTYGLPNNFEKTNKLLNK
FSKIYFVYHMFIV
TGMTTSTLLTIGTCEEENLENNINDICGLVGPTWLPFEFDYFPLKQIVYGYQVYCSF
VIFQLAGHLSYTL
MESVEHLIIRFEHVGHTFVEALNEKNSYTRREKFYVAIQYHNDVIQMGKLLNSCFAP
SLIVHISLTGPVL
GVAGYRFLTEIPLDSTCLFFGWMFSTFIVCRGGQRLSEASLAVGDVIYRVNWYNLET
DLQRDLKMVMLRS
RKPVYLRAGPFGPMTYSTIVTILKTCYSYITLLKQTM
>CbowOR19
MFRVGDAIAFQSTINYMTFFKVFTVQTDTRYVGTILRFWSFCLICLFNTFHLVYVKI
ENIDVDTSEDLVV
ILGGMGILLICIFSASSSRRWTSFLHNLIDFEKYGKPDGIEHAIERGNYWAHFFGLY
YIIGTVIYGIVTY

MEAPSCHRLNNEKNLHLICDTFVAIWLPFDIPLKSIRLSVFFIQFVLITCNVNPAAM
ACFLTWECTEILR
CHLRHLKKHFHKMVKEGDVRKRPDGIGYWIRYHNHILSLSYELKSLFKISVGHTSLI
SGLVIGCTENQIL
KSKPLGASLFFLGWMAAMMLLCHAGEILMEETLSVADTLRDSQWYLADLETRRDMVF
IMLRSQKPVHLEA
MPLGVMNYALFVMILRTSYSFMTLLNQSS
>CbowOR20
MRDVEEAEKSLMVTGWFPFDTREYFAVAYLFQLQIAIIGGLFLVALDSLIISLIMVA
PLRLKVLANYFRH
FGDKKSMNSLLSLKNLISEHQGIIRYVEDLNASLKWLFLADFVVKSYNISIVLSNAV
SIYEFRNIVIIIY
YVLQTRGRNKSELAFSALFLCFLLSQLYCFYFHANEILLESTNLAENIFKSKWYEQN
SQIKRSLIIVMIR
SQKPLQITIGDLHAIENILFVKIVKAAYTFLLFQYLGL
>CbowOR21
MKTQFQKAFQKEKWILTSLGSYPQWEPEALWQHCRRFFCMTVSITYITLMCTGPLLE
NSIMILCTVMGVV
KEMQLLFSKQQFREIEEYIGSMKPCKIPISRLLGSFRNSVVTVIIFLGLMPYTKRSL
RMLPYKSWLPYDV
SRAPVYYVTFVAEVLTIIMAAFTNTTIDVLYYCLIDICCAELDLLKMELMEIDMSES
YEVVQNKLKNVVI
YHQRIIRLVEVIQDVFSSVVFIQCMTSVLVICFLGFQIVYVDEIPSGKATIEVSFIG
CMLLQIFSYCWFG
QSIMMKSLEVADVCYNSNWYDADLRIRKMIFIIMERCKQPLELRAKIITINLQTLLA
ILRSSYSYMAILR
TLYSD
>CbowOR22
MSSFSESPENEEQPFSATTKMMRLLCVYPLGFEKWQMVRFYVNVVVVKLFSFFFCCV
LCLLHLVMTKIDG
AHKADLSEDVSMIMAGTGMLATNLLFAYKVKKWNSLMGKVADSPEMRNIQNFEAIKK
RCNRLARLFTMYC
VIGAGIYLLSGYYESLVCIRKNEENGSNEICRTLMPVWLPFRLSSAAELTLFALQAF
AGINLSLPGANMP
FLVWEITEMISLRISHLKKISESIVVEKNIKSRQERLKHWVMSHQQIIECISLLNEQ
VRLCFGHISTIAA
LVLGCLANQAINSVHLGAMAELGGWMVGLFLLCSSGQKITDITESVAEAIYAMEWYS
TDVQTMRDIRFIL
MRSQKPLVLQAGPLGALNYPLYMMMVKASYTYLTLLANTI
>CbowOR24
MMTEKDEKMPKKLEIVCMPTSIRIFRFYCAFPPSDKLLNPGKMFYIRFALIALFSSV
VLVGSTMHLIKNV
KDRTYNHIELDFTYIVSNLAGYGLLCSYFTKVNAAVQLYLILSDFEEFGKPINFDNT
NKKFNKYAKYQYC

YLESITVCILLGSNMFRGAQCRKDNAELDQHEVCGLFAYTWLPFDIDFFPVKQIYLA
CQLFGIHYVYMMA
GLASWMVLESVEHIATRLRHVSHFFNEALKEAEQRKRREKFNFAVRYHVAVLDLESK
LNQTFSVFMFTHM
VMSGIIMGYGVYSYMKGKNVSTILIATGWLIGLLMDCYSGQRIQDESTLVGTALYDA
DWSDADDELKRDI
RFVMMRCQKPMIVQATSFGIMDHPLFLAVLKATYSYVTLLSQSDL
>CbowOR26
MKHKKLYNFYTIFCTSVWVTFILSQLVYMFSSFSNMDEMTSIIYVAGTVTIDLVKML
AIYSNMDRIKPLL
NDLNNPLFQPKCKEHVELALAVKKFHSRLFYFCLYFGVQTYICFSAIPFILEENVTL
TQGWFPIDWTYSP
NYEIVYAFQNIVILWNTLIFLNLDTFTSGLLMQVGLQCDFLSTTFNKIDAFHVSSGV
LIENGEQMALSLK
DNHEFFNRVMTENLIVCVKHYRKIRRLATEIEDIHHISVFILFLGGAIIICADLFQL
SIVQTGGVEFVLF
VSFLMCMLMEQFMYCWFGNEIIFKSDNIFAASYNTPWLDCDLKFRKILLNFMTQSID
PIGLKAGGLFTMS
IKAFVSVLKSAYSYFTLLQRIQEKECSELN
>CbowOR27
MIPSEDNSLSSMWLTKLILKSIFMWPDDYSDTKRKTFYKISMTICLFIQSGLVLNLT
QNYHDWEKNLAVV
SSMSTIFQTVFKMTALYQNSDHIKFVLMCMCRKFWPHNLDNENSEYIFKQSHSRRMR
LMVFLLASGFLFS
LGSVISPMFTRDTPFKSDYPFNWRRSPFYELIYLIQVAANGYLINMTVIGFDFLFMD
ICAALTNQYVLLG
SCFERLGTENMQDFYARIRERGCQKWPPKVGGARRFLGICVQHHQLLTQITKVVGHI
FNVVAFLQLCSSV
VAICVSGFIATKDDVTTSQIATMGSYLIGHLIQLYIYCSVGNELLFQSSTLTNHIFG
SNWYNLDSTTTKK
DIIFIMKKAQIPAKLNAFKVFPLNFATFIAVVRLSFSSYTLLTSITNK
>CbowOR28
MYASNLDWCLKLNFLLGVHPAKQKSFTQTLQYLFIIFGSCAIMILTVLLLYYKEDAV
SMKDITDVSTNFT
MFPHGMIKLTTLYMKRAEILDLLRRTKEHFWQIKDDREDVKRSYKLAKLLKNLFFNS
VVLFIISAIVKPI
IIGGNTLTYKCHKPELIPRWLFLIFQDAMCVAILFTLSCTDVLILTLLILTQIQFRM
LNEKIQTIHDNEV
DFHDRLKECVDHQNFLMDFVDRFSKVFSKTILLFIGNIILSLCMCMYIITTESANIN
VQMEALFHLIAGL
NEICLCYSIPAQTLMNEADEVGKNAYFSKWYEHPKDAKLILQIMIRDQKRMVITAGD
FVRIDMEMFLTAC
KTIVSYCMFLRTMSMVDQ
>CbowOR29

MTCNEFASFTFYIPFFTATKWQCIISSTAMYSGMLFEAGIHAVHDGIFFGLIYCSKI
QLQIIGDVFRTIR
QRSLSKLNIPEDYSVLHDEENPALEEELYRQLSHSTDHLKILLRVQDEIKKLFTYVL
LAQIISMLFTLAS
FLYLVSTISINDPGLFLQFFWFMTILLQLTLFCWSGNGITEGFDSINTALYESEWLS
CSRRFKKSMILTM
TRLQRPVLLSLGGFSPLTLATLVGVCQGSFSYFTLFQSYQ
>CbowOR31
MMAFGYPKNFFHMNEATVRFLGVWLPSKKHHILIRLLHPFYFIFVYSTLIYFVIGQY
MKVEMKNVTTIIS
SLAVLLTTHAGHVKGSLVVFGGRRIQEIKDILQDVNYQYYPVGEVNPGSTFQKEKTF
YTMLSYALLVGFM
MPGASGTVTTASRLMIEMKGNNTFESIDKNCNDFITYTFYVPIFIETKWECIISSSL
MYSGMTMYEGIAH
AAHDGLLAGLLICLKTQLLILGDIFRTLRQRVLSRLNIPEDYSVIHDEENPALEEEM
YRQLCLCTEHLKI
LLTARDKIEKTFTYMLLMQTIASFPVFASSLYAASQTPLSSTDFYTNIDFFGCVLVQ
LAMFCWFGNGITE
AGEAIRSALYEGDWYSCSPRFKKSMILTMTRMQRPVYLSIGRFSPLTITTLVSVCQG
SFSYFTLFKSL
>CbowOR34
MTFFFKIKGLSPPKTTAVKIIYLSLALPHILMCTFVLILSEWMAFALSQQTFKERMF
NMSVATLDTILVF
RTTVWAFNKAKLDEIRAIITRKSFNFRCFDLLKVGCEQVLTVGRTKEVEKERGLSCK
EIKQLWQKAKFVT
EKKCNEMDVFRKELMLNTRLLCCFIHLAITIISVLTYTLSFVNDFTNDTYEAYNPIL
NRTSLYRKFQYPL
YLPFDTSFDGYYWLAYFYNCYAHLGNIITFLPIETTLTCSLIHLISQTAVLKEAFKY
VDENNFGYQSFGE
ASIIKEIRIVKCINEIQEIYRAVELLENLCNVQLMVQYGFATFLLCSICYVIPLVEN
TMEGICCLIFFAA
SLGQIFTFSYCCHTLALELQAIGVSVYNLDWTNYPSKLKRTLNISILRTQKPANLTA
GKIIVIDLLFFIQ
VVQKSYSFYTLITKTN
>CbowOR35
MSHNEIVCMTTSIKILRTYGVFPSQSRELHPGMVFYIRFLVLAVVTSLTLVGSTLHL
IKTIQNNEYNYTE
MDLVYIVSFVTAYALIGSFVMKVKASGEMFVFLSNFEEFGKPINFDKNNKLFNRYSK
YHYVYLESLILII
LFSSNIFKSKTCRLENELYNLKEVCGLFTYTWMPFNIDYTPVREIYLTIQLLGNHHI
YMLAGLVAWQVFE
TIQHIIIRIRHVKHLFVEALQEGDVKVRRKKFNFAVRYHNAVLSAFFSLEDKLNAAF
GIFMVTHMVLTAA
VIGTGIYCLFRRRSLSSFLVCMGWFWGLFMDCFSGQRLQDESLELAVALYDSPWYEM

DKEFIKDIMFVLS
RCQIPMKLRAYAFGVIDRAMFLAVMKGTYSYITLLRSQ
>CbowOR36
MYFFYVSFLYGTGVIFFVCEFMIFNETIGKISKFVSHIGMLFTHVVGILKMSILIFG
RYRILKIMNVLQN
EKYHYAPLEDSQPGLLVVKEKFVSSGISILVFVLYTFVGVSAHISSLITINEEIKGD
SFEGTNKTCHDYM
PYFFYIPFPTETKGQCGIAFAFMDVGLGIFAWVIACHDGVFVGLLNCLKTQLLIVCN
IFTTIRARSLKAV
NLPKNYKILHDEYNPALEKELYRQLSHCTEHLKLLLVVRDDLEIMFTFVTLSQTLAS
LLIFASCLYVAST
VPMTSPEFFAQMEYFLCVLVQLSLICWFGNEITRASELIRLSLYESDWLSCSRRFKS
SMILTMIRMQRPV
YLSIGKFSPLTLATLVAVCRGSFSYFALFKSVQ
>CbowOR37
MYFNTIQKVLPILYIIGADPREGFTKSQFFLYFYNIFSAIGMVYLLVLKFANAENKV
TVKDITDAVICLF
LFCHGMVKSTTMFVKKNSVQTLLAQMEKHFWPMNNYKYSYIHNGILNICKTIRNTTN
FIWFMHFCNAMGF
LVGPLITKDPVLPFECYRPEWMGYYTLLLFEDVTSIITILCPVLAMDVFFVTIIKLT
QIQWKMLNSEIQS
MFDLSPSGKISREDEENIMVMKIKKCVVHHNFLLNYQQLLNDTFSIPLFFFLIVIVL
CMCVEMYVISTVS
DWESLRTAIVYTATGCLEFMLCYCYPCQDLSDEADNISYSIYFSNWYRNPEYFRDTQ
LIMQKGQKLVAIR
PGGFMIMDLKTGLSVGIFPSP
>CbowOR38
MFAGCIFTCTFWAICPFTEDVASLPIAAWIPFKTDSSPSFELAFAYEIIATVIGGIT
DLNADCFMAGFIM
VVCAQMKILNDSLLNLRHFAVEELNAETGGNNDDDGIAEELQKIMNRKLVECVHHHR
YILEFAEEANSLF
TTSILGQFAVSVIIICTTLFEMTLVPFASIKFISLILYQYCMLMEIFIVCYFGNEVI
LESSKLTKYAYHS
DWRDCSQEFKRNLLFFMTRSQRALKLYAGGFFTLSLDTYVKILKSSWSYFVVLIQVN
KDSG
>CbowOR40
MYEKEFRNVFWLLNFVGMHPLKKYVTPFIVFNAILTFYITVLIILKLLWDKELVAVE
SLCVFSQIWLKIF
VLTTKKRKIKQVIDDTQQFWENDPENSENKQLLKNLAKLERIFLTYISCSTCMFLFK
PLLVKGTSIYYYY
KIPQIPFYVSYPIEFYVTIVTMALAIAVNLFISIVIVIGAGQFSNLNAKMKQLDLSI
AEDCQDGLRTCTL
EMNKNIEYHDFLIKYVSHLDEIFSMLFVVLTGIITSLLCMNMYVLSQPTTTAVDMIR
CGTMVCAFTSEFL

FLYGVPAQRLMDEAEEVANSAFYHCQWYLPNIIPLRKSLSFMIHRSQKSVCLSAMGF
IDINRQTIVAMIK
TAYSFFTFLQTIETTGGAK

>TcasOR1
MMKFKVTGLVADLMPNIRLIQASGHFMLNYHADNSGALHTLRLGYCCMHLVFVLVQY
GCNFVNLVLERGD
VNDLAANTITVLFFTHCVTKFVYFAVRSKLFYRTLGIWNQPNSHPLFVESNNRYHGI
ALKKMRRLLYIII
IWTSFSAIAWTGITFVGDSVHNIKDPENENLTITEPIPRLLVKAWYPWDAMSGMPYY
ITLVFQIYYVFFS
LAHANLLDSLFCSWLIFACEQLQHLKEIMKPLMELSATLDTYVPKSADLFRAPSATS
QDQLIENDYNEKN
EDLKGVYSTRQELGGHFRGGALQNFGSGGVGPNGLTKKQELMVRSAIKYWVERHKHV
VRLVTAIGDAYGV
ALLLHMLTSTIMLTLLAYQATKITGVDKYAATVLGYLLFALAQVFHFCIFGNRLIEE
SSSVMEAAYSCHW
YDGSEEAKTFVQIVCQQCQKAMSISGAKFFTISLDLFASVLGAVVTYFMVLVQLK
>TcasOR6
MWSLLPVLNGWTWQKKLPFPARYPLDVTKSPYYELAYVYQFICIWYITVANLNLDTI
IIALMMYTSCQCD
LLCDDLKNLTETRFFDKKLIECIKHHKAILVFAEKSNSLFNMIVLGQIATSTVVLAL
TMFQLSMVSPLSS
EGLNHLFYIGGIIMQILLYCWFGNEVEAKSSNILYAIYESTWSEASKNSKKNLIIFS
IRCQRPIKATAVK
LFALSLRTFITIVRSGWSYFAVLYNVGSE
>TcasOr7
MNKLQKFDWKATIRPNIAFLHYLGIWPEGEEYYKLNFYTLKTILYIIILVISTIVFQ
VINIFFT
LDDLTSLTANIYVLLTEILYFIKLCFLVKNMPALKLLMKTLDHKLFQPKANQIVIIQ
PLLNFW
KLIFLAFVITCSFTVLFWAIFPILDSSEEEKRLPLLAWYPYDTKISPNYELTYLHQV
ASYIYIC
YSHLNIDTFITALNTYIQCQFDILCDNLKNIKSDTKNVDTKLAKCIKHHLLILMFAN
TSNEF
FSWIIFFQFTSSAAITGMTLFQLTVVKPFTTEFYNFMAYVTAEVVQIFMYCWFGNEV
QVKS
SNIPYAAFGSDWTEFSPNKQKSLLFLITRSQKSVKMSAFNVFDLTTDSFILKSAWSY
FALLN
QVNS
>TcasOr10
MEDFSWEATLSQNINFLKVCGLWPPGDEAYKFNLYGIYAGFCVLGFLCVHTGTQTFN
VYFILDD

LEAFTSSIFVTFSCVACVFKTYYLLKNMKLLKVLFININKEIFQPKNKEQQLLIQPS
ILF
WKRFYLVFRILCYNTCFFWCAYPILDKRIKQHKLPFLAWYPFDSSVSPLYEITYFYQ
AVAIW
YIVIISFNIDMLIGALNMFVGAQCDILCDNLRNLGKSDINELNPDLIKCIQHHKAIL
SFVSKL
NIFFNWIVLLQFFSSAVSVGFTMFELTLVAPFSGQFYSFICYGSAITTEMFIYCWFG
NEIEIKS
SKIPYAAFECNWVGTPLGVQKSLIIFTIRTQRPMQVSALNLFYLSLDTFKTVLRTSW
SYFTV
LNQVHS
>TcasOr11
MEKYDWMQAIKTNILILKIVGLWPDSEDYKFDFYALHASVWLSTLLVASTFFQGINI
IFILD
DVKALTGTAYVLLTEILAVIKTYFVVKNMKMLKHLMQSLNNNKLFQPRSHEQIKLIQ
PSLK
FWKLLYNLFHSLVGGATLFWILFPIVDKKEKRLPFLGWYIVDTKVSPYYEIVYGFQF
CSCC
YMSALIINIDTLIAALNVYIGNQIDILCNNLRNLKAGCSIERDLITCIKHHQEILNF
VQYANK
FYRWIILLQFFVSAVSIGITMFQLTIVVPLSSEFYSFLFYANSIISQIFMYCWFGNE
VQTKSNK
IPQAIFESGWTDFPLKTKKDLVFLLMKTREPIKVSAFNLFSLSLDTFMRILRTSWSY
FALLN
QVT
>TcasOr12
MQKFDWRSMIKMNIVVLRCVGLWPSGEESYKPGVYTIYASTVLTLFLLGHIFFQAVN
VYFI
RNNLSAVTGTIYILLIEILLVFKVYYLVKNMTVLKQLLKMLETEMFQPRNSTQINEI
QADM
KFWQMLIRFLWVSVMCSNLFWAIYPLVDNAGKEKRFPFLAWYPYDAQKSPYYEITYV
YQ
TISVNYMSSIHVSVDALAGALNVYNGNQFDILCDNLRNLHRLTKNGTIDAGRNFRYC
LKH
HKHILDFAKKCNNYLNWILFMQFFVSTISIGITMFQLTVVRPFSNEFYSLFTYISAI
IGQIFM
YCWYGNEVEVKSSKIFYATFESDWIEFSEEVKKELIFFVMRTQKPVKLSALNLFYLN
LDSF
MRILKTSWSYFALLHQISNRNS
>TcasOr14
MLLKWSSVIEFNLFLLKWIGLWPGEDYQLNMYSFYGFSVIILILCGHTLSTGLTLIL
DSGDI
DTFTETMFILNIEFMTAWKALNFALNRKKFMQLLDAIDKTTFQPRNGKQVTLVLRNI
DGW

KVMFKMFGISLGLSFIFTGLLPIFSKTYKDRKLPMEAWYPFDSTKSPFYQLCYVYQM
AAV
AVAVMVILNVDTLVAAMNICIGLQCDLLCDNLRNLHTNTSKSMNQKLIECIKHHQNI
ISFAE
KFRQAFNWSIFLQFFVSTTSLGIVMFKITRFSLYVSEYYRFISYACSVLVQVFIYCW
FGNEVI
VKSSKIPYALFESDWTQDSLEMKKNMIVFILRTQKTLKITVCHVFDLSLPTFLTILK
TGWSY
FAFMNRVTSPH
>TcasOR15
MLVKWSSVIKINIFLLKWVGLWPGEKYQLNVYSFYAFTVIILILCGQTLSTGLTLIL
GSGDVDTFTETLF
VVNIEFMTAWKALNFALNRKKFIQLLNAIDKPMFQPRNDKQVTLVLRNIDGWRVMFK
MFAISLALSLIFT
GLLPIFTKTYKQRKFPYEAWYPFDSSKFPIYQLCYMYQMASASTLVVVILNVDTLVA
AMNICIGLQCDLL
CDNLRNLHFDTSKSMNQKLIECIKHHKSIISFAEKFRQAFNWSIFLQFFISSTSLAI
VMFKISRTTNYGS
EYYRFISFACSVLVQVFIYCWFGNEVIVKSEKIPYALFECDWTPEPLEVKRSMIIFI
IRTQRILKITVSY
MFDLSLPTFLSILKTGWSYFAFMNQVTEVNTSK
>TcasOR16
MDKYDWRSHIKINILVLRFLGLWPKNTYHGFYIFHLIFMMGTFLLGHLFFQAANIYF
IRTNLEAVTGTIY
VLLVESLVVFKVYHLVKNMAMFKQLLEILDTEMFQPKNKKQIVAIDETIHVWKTIYK
SFLYTCFGTNAFW
AIYPLLDKSEGGKRLPFLAWYPYNTTITPLYEITYVYQIVSVSFITTVHVNVDVLVA
ALNIFNGSQFEIL
CDNLKNLHNGPVKENLIECIKHHKEILKFAERCNNFLNWILLVQFFIFAVSIGITMF
QLTLVIPFSTEFY
SLLTYGMAIILQIYMYCWFGNEVEIKSNKIPYAAFECNWVDFSPEVKKNLIFFIMRA
QKPVKLSALNLFY
LTLDTFMMIIKTSWSYFALLHQVSSRK
>TcasOr17
MDDFNWISTVKTNLLLLHIGGIWPRGDGTHKLNLYTIYAIFITFTFTTYHCFSQIIN
FFFVDD
LQALTESIFISLIQSMALVKAFYILKNMRILKNILKNLETNKMLQPRNLKQIKMVQP
SLTQW
RLLSQMFWISAVFAMCLFGAFPIVESTYKEFRLPYLAWYPFDTKSSPFYEIMYLHQF
VSSY
TIAIVDIGADTLIAALNVFVATQCEILCDNIRNINGSVEEMDSKWKECFTHHKEILK
VARHC
QKFFNWIVLMQFCASVICIGLTMFQLTLVVSFSSEFFSSLFYFGAITVQIFMYCWFG
NEVEL

KSSKILYATFEANWVEAPHQVKKNILIFAIRCQNPIKMSSLNVFYLTLETFMAIFRT
SWSYFA
VLRQIQNRISEE
>TcasOR23
MANFNWTKIIETNFVVLKVIGLWPEKTFFACKLYNIYTHFMVTCLLVIHLLLQTIQL
ALIIDEFQLFLTA
LPLLLQQYHLLIKLFYFMVKFPILRYILHSLNNHQVFQPQNQDQKIQMEDRLSFMKK
IYFSFYSMAGVAI
SFLVAFPILDILNGGERQILFVCWFPYDYMTSPFYEFTYFYQSASIIYAGVIVLQID
TLVTLLMTYLGFQ
CDLLCENLTQVGYNNSKENNTELEFVKCIKHHQELIKLKNHCVDFSSGLIFVQVATS
SIAIGLTLFQMTL
NVSTFNVIFLVLYGLSVTFQMFQYCWFGSEVIHKSDKIAYSAFEMNFVDAPLSVKEN
LVIFMACTQKPIK
MPVLKVTHLSLQTFTKVLRTAWSYFALLVQVSK
>TcasOR24
MRRLKKLLLVDIRQKLFKPRNRQQVVMVQSRVNFWKKIYFMFTGMGVATMFFWALFP
IMDGTVKEHRLPF
LAWYPFSVNKSPFYEITYIYQIVSVFFIVIVNMNSDMLLVALMNILGVQCDLLCDNL
KNIQFRERINEEF
LRCVNHHMQILSYASDCNKFFNTIVLAQFFTTVVSLGLTMYQLTIVTPFTSEFYSFI
VYGGAVLMEIFLY
CWFGNEVEFKSLNIPFASFGFDWTIGSVGLQKNLIIFIAKSQRPIRMSALNLFHLSL
ETFVKILRTAYSY
FALLNNVNSLN
>TcasOr27
MLETKQIVFHSFKLNVTVLSLIGLYPPKNYSILYKIYAVILFLAVHTPQLVLGLLHY
FLMGDF
TSIDYSDFVTVGMMFYAFKLLPFVTSVTKIQKCINYFDTLGYKILKSEEKIIEDCVG
SCRRN
TNVFFVGCCLSWMGFVAQVFLRDEPQQLPLKVWFPYSRDESPVLFYCIYILLIFGPG
YSVL
ACGTIDPMIGGLAYHAAAQLQRLKRNLQYLDEYIKEKNVGKSKENKRGVIYEEIISC
VQRYQE
IATFVDLFKDSFSQVVFSQFMGSVFLIGLCCFQIITATEVDINFVITANYIWVILFQ
IFFY
CYYGTMLIEENYTLTNAIYLSNWYEYSIPEQKALFMLMERSKKPMIVTAGKILDLSL
DTFT
MILRRSYSLLCCLK
>TcasOr32
MCLSTSEQSFSINLKIMKLCRLFPPTEGKKFYKIQAYLLQFLLLLPIPILGNLHLLL
DENLDM
EKVNYNAVFLAQVTCFVIKLMAIIANSEKIKKCITELDSPKFAAVRENHKIILQHCI
KVCKR

NTLIFVVFVICGASSWATKPLFWSRRNLPLDVWFPLDTTSTPVYCSLYIYLLIGVYF
TSFAN
MVIDPLIAGLAYHATSQIKILKDNLQHLNNVYANEEITSSKNKIIYMKIKRCVQHYD
DILSF
VKEFEECFSLAIFSQISASVFVICFSCLQLSKIKTFGYYFIQLVFYFGVILAQIYFY
CFYGSTLF
EESSSIINAVYSSKWYDFDVPCRKALLILMERAQTPITVAAGKIMDLSLVTFATILR
RSYSLV
AVLNNYQ
>TcasOr36
MKGLVEKSFRVNLLVMQVMGFYPPQKYKSLYKIYTYVVYCAFTTLIPVLATLELFLA
ENIN
LEQISDNAFIVCEAGCFIIKYLPFVRNADKIKKSLFLIERPMFHIYTKRQEHIIEEC
VAICRRN
CRLFLTFCTITVINWSITPFFLPGNNLPVEIWSPFEHKASRKFYFLSFVYIVAGVGN
AAVSSG
VIDPLLAGLISHATSQLKVLKNNLQFLDEHAEERIASRNISFIERKRFKADFIYQQI
KLCVNH
HIAITEFIDVYEDTYSSSVFIQFAASVVVICISCLRLSMVEPFTFTFFVMALFLWTM
LCEIFLY
CYYGTILYEENHSLTNAIYMGKWYNYDIKSMKALVILMERSKRPMIVTAGKILDLSL
ETFT
TILRRAYSLLLVLKNYESTPTE
>TcasOr39
MSNQHEIDLTEFVKLNIKNMHFFGYFFPRFGHNKTRKTLYTMYSTLFVGSTFVLTAL
SQIA
NMINSFGDMERMTEASFILFTNVVQCFKIYSFLTYGPRVWNLIDGLNRNIFKPINTD
QHRIL
VNDIYMSKKISKIFLLACTLTCMSWAISPFFDKRGDVLRLPLSGWYPFNTDKSPAFE
LVYIY
QILTTWIGGMGNISMDTFISGIIMAISSQLSILNNALKNITKNNELVRCVFHYRIII
KFSDEVIY
LFNTCLTTQFIVGVIIVCISMFQMSLVPVLSFQFVAMLLYQMCILLEIFLWCFYGNE
VMLKS
DQLTQAAYMSDWTKSPNHFKQNLLFFMTRTQFPLKLYASGYFTLSLETFKAIVKSSW
SYF
AVLNQVHSRQTQ
>TcasOR40
MIISVTGFVTKPFLFEERGFPVDVWLPTSLKDRLDVYWGFYIYVSIGVAYPVIASGV
LDPLIPSLLCLAT
GHLKVLNDNLEHLDEYSSEENGSKDSNLYKNIQKCIKHHIEILNFVYNHQKCFSLMV
FSQFLGSPMILCF
TCWNVSMREPFSLEWFQSLAYFLGLLLQLFFYCYYGTRLSEEFEHVTTAVYMGKWYK
YDVKSRKALIIILM

ERSKKPTIVTAGKILDLSLETFTIILKRSYSLLAVLKNQN
>TcasOr41
MDNTLDIDLTEFVRFNVNSIHFFGYFLPEFGKHPKKKIIYVIYAVIFVGTTFGLSLV
SEIANMI
NAFGDIEKMTDASFLLLTNLVQCFKMYSFLTHGPRVWKLIHSMNNSDFKPKNLEQRN
ILV
EEIKMSKRISKTFFMACTIVCSLWGISPFIDRGNSEKLRLPLSGWYPYSTDTSPGYE
ITYAHQ
TLTTWIDGLADVGMDTFLSGVIMVIAAQLSLLNNSLKNLTKNCKNDGKKANTNLIEC
VIH
YRTIISFADEVTYLFTSCITAQFIIGVIIVCVSLFQMTLVSLRSFQFFSMFLYQGCV
LMEIFLW
CYYGNEIILKSDELTRSAYMCEWIEESREFKKNLIFFMTRTQFPLKLYASRYFTLSL
ETFTAV
VKSSWSYFAVLNQVHTK
>TcasOr48
MPHATLSKMVVQKIDLLEPFDNVTRLLKILGLWYSPNETIVYKIYKNFVMATCFLYT
LTCT
VYGFKFMSFETLEIAFGAVEGVLKSLMFRLKFQKIAESWQQIRQQEFQPRNEHQRTV
LKW
YIEVTKSLFLVYFFGVYIGCISALTVSSWLRHKDFPTDHWFPFNYRRPFLYQYIYVH
ITVGF
YLTAFLNCASDSCFYLSLLHITAQCEILADTLKNVHDLHKLNAAKKNSGQKGEDEVM
NQI
LIECMKHYNLIKKYTSLVADCFKEIITLQFVPTIVMICIAMYKISTLEPSNTQFWFF
AFTELG
AITQIFIYCFVGNLVTSTSQKLFYATFESQWYNASQKFKKNLITVMMAVQRPVIFYG
WNIF
AINYATFKSIVQTSWSMCVAFRSTQDL
>TcasOr49
MVVEKINLREPFENVTRLLKILGCWYFPNESLVYKMYKNFALITCCMYTVTSIIYSF
KYMS
IDYDKAYESLEIGVGTAEGVLKGIIFRMKFQKITESWQQIQQPEFQPRNEKQKMLLR
RYIY
VTKFLFKVYFFVVYIVCVTGLIVSSLLRHKDLPTDHWLPFDYRKPFLHQYIYLHLTA
GLYL
NSLTNCAVDSCFYLSLLHITAQCDVLADTLKNIHDLDKLNAKNAPERENKDQVMNKI
LVE
CMKHFNLIKKFTNQITDCFKEILTLQFVPTVAMICMGMYKISTLQASSSQFWFFVCT
DLGA
TTQIFIYCFVGNLVTTTSEKLFYATFKSQWYNASQKFKKNLLTFMMAVQHPIIFYGW
DVFA
INYETFKSIMRTSWSICVALKSTQDL
>TcasOR50

MFTDSDKAYETLEIAVGSAEGVLKGIIFRTKFQKITESWQQIQQPEFQPRNEKQKSV
LRRYIEVTKTFFK
VYFSLVYVGCVTGIVVSSWLRHKDLPTDHWLPFDFRRPFLYPYVYVHVTVGLYLNSF
TNCVLDSCFYLSL
LHITAQCDVLADTLKNIHDLDKLNGKNVPERENVDEVMNKILTECMKHFKLIQKFTN
VITDSFKEILTLQ
FVPTIAMICISMYKISTLHPSNTQFWFFIFTDIGATTQIFIYCFVGNLVTTTSEKLF
YAAFESQWYNASQ
KFKKNVITVMMAVQQPIIFYGWNVFAINYETFKSIMRTSWSICVALKSTQDL
>TcasOr52
MSQIDLKEAFKQNIVLLKAMGLWFFQNERFYKLFKCFVQGSLVFDSTSLIIYVALNI
RIKNV
TDTIYSLPGSLEVVLQAILFRKNFHLIRKSLNNLKQKEFQPKNDTQEKILKDSIALS
RRVFY
SFFWLVFVMIGMWMVLPLTKKGKYLPTKYWIPFDYRLPVVYELLYVFECSCIIFHAF
SNVA
LDTFFSIAMIQIGAQCDVLCDTIRNMDEQEKTNTMDRILIECVHHYRLIEDFAKSIA
TSFKEI
LMVQFVCSSLMLCVSMYELSLSEPMSGHFFQVLLFQISATNEIFLYCWFGNEVIIKS
ERLFY
AMFESKWYDSAATHRKNLMIFAHQVQKPISLLVWNIFPVDLKTFGGLLQKCWSFFVA
MK
NIQEIQE
>TcasOR53
MGFNFQSRTNKILRRLRYVGTWFENDSYDLYFLYAVLLNIYYNLHNIAQTMNVFYHL
DDIEEWSSSGLLT
LTTLLTNFKAYCVLTNKKRILKLNQILTRSVFQPRSDHQVKMATDKFKIFDTMYSLH
SSGPTLTVVFFSL
YSLAELENRKLPFNAWYPYDFKKTPNFELTYLFQFTACMVQALIHVNTDSLAFNFIA
ILVIQLDFLADNL
RNMCQKAESMEQSLLDCIRHHKEILACRNELYHILNVNLFGQFILSTTALCMTFLQM
TVVNPTSTHFIAI
LVYGMALLVELLMFCWWGNELIIKSQLIPQAAFESNWMEGSIFFQKNLVFFICRAQK
EMMLYAVGFFRIS
LNTFILVSTSVSENVVY
>TcasOr54
MNLQKLDPLEGFKPTISMLKIFSVWNSSNMFYKIYKNVTTLSLAITYTCVMICVVVN
FNV
SEINENFYYIPALSTAPFKLVIFQKSFKKIQNLLFLLQSQYTKIRSEKQAKMVEDSV
VLSKRV
VKVFAVLVVPTCVGLFGMPLLKDEIKLPLIIWIPFDYHEPVVFGLVYFVISFSGSFT
AYINIGT
DTFFYNCLIQIETQCNILSDTLRNLHEFGRFEAEIHTILIECIEQYKTILKFTKILS
KTYQGILS

VQFICSLLSLCLTMYRMSLADPGSEEFLRYFVFQWGVLPEIFLYCYFGHRVLDSTKN
LYYS
TYELQWYNTSAKFKTNLLIFMGQIQNPIVIYVAGIFSLDLETFKKIMQKAWSFFTAL
RNIHE
Q
>TcasOr55
MKAFSFNIKLFQIFGQWCYENESFYKIYKYTATVLLFLDWLFTMIFVLVNFQESEVV
DSLYI
SPSMTTSMLKYVIFRVNFSQVERMLKIVEEQYLKIDSRRVGFLVERGTKSSVFVIKA
CFYLV
MATVVSLITQPLLQEDIDIPLVIWLPFDYHRSGIFELIYVYVSVSYLYFAYVNVATD
CFFYIS
AIQIGVQCEIVGFMLENLNEIAKQEEENVRRLFLSCVTYYNNILECVKIISDCYREI
LIVQFF
CSFVALCMTMYQLSIVEPFSDVFFKMCVFQSAVICEIFLYCFFGDLVLEKSGKLFYA
SFSFG
WYNGSAKFQKELLIFMNQLQKPIIFHVGNVIPVTCETFKSIMQKSWSFFIALKNTQN
R
>TcasOR57
MSHSNPLEAFKLNTFFLKALTVWHVENPTYRLYKIFVVFSFAVTFFSAWICALVNYN
VSEISENFYYLPA
MSTGPLKYAIFQKNFTNIVNLTHLLETQYAKIRTENQKKIFDESVIFERKVMKNFAI
LIIPTCVAMFIVP
YFQDRREMPLIVWFPFDYKQPVVFDLVYFILAFACISIAYTNVSTDAFFYTCLIQIE
TQCEIVSDTLRNL
DKIVTNGFRNVAESRKIFIECIEQYNVILRYTKIVSDTYQGILVVQFFCSLVALCLT
MYKLSLADPGSQD
FIKYFVFKLGVISEIFMYCYFGHRVLEKTEDLYFAIYEMHWYDASKQIQNEVFIFMG
QLEKPIVFYVANI
FSLDLDTFKKIMQKAWSFFTALKNMHDIRNN
>TcasOr59
MDEEFLIGTFETEKKFLRYGSFYPCGKRIKFIFLGLFMFVYSWTEFLSMITVLFVER
DNLTK
LSETLLFCMTQAAFLFKLVNFLYHNKTMLRIESILKNPILNCLDQFEKNIIEKYMIR
VKYLA
RLFRILCILTVSFYGLFPFIDEDPDHMLPLPGWFPFDVKTHQIELVIAQTCGIAIGA
FLNSTLD
ILPTILITLGSAQFDILKIRLENITSVDTSKSWLVKKAIKKCVIYHTILLNYITQIE
ILFHKGIFV
QFTASVVVICLTGFQMLVISVRSIQFILLMIYFSTMTCQIALYCWYGNELMYRSMGL
SDAC
YMSEWNKCDTSVCKSLAIIMERGKRPVVLKAGNIFSLKLTTLMTVLKSSYSYFAVLQ
RLY
ATSE

>TcasOR60
MIIQSVIKRNDLMVLSETLYFLTTHLTFVCKLANLEYHKKLLLDIEDMLKTTRFQKT
LSLDLIEKTGMNE
KIRKFNLVAKTFRIVCVWCVVLYVLVPYFDPGKSKTLPTPGWFPFNWTDKYYYGTYF
FEVAGISITAHMD
SSIDILSWLLVTIASFQCDILKENLKNIYYNYDKEHDIRETFKDCIRHHEEIIKFTT
KVEQSFSQGILLQ
FLCSALVICFTGFLMLVVPVLTFQFANTIMYFCCMMIQLGMYCWYGHEIMTTSDEIG
QYFYLANWYDSSL
TLRKDFAIFLERAKRPITLTAGGFVVLSLNTFTRILRSSYSYFAVLKHLYNKS
>TcasOr63
MGFMIQDYDLRNAFSLERKLMLVVGFYPKRDNKHEILYWLSAFFNLLISYGQLTTMI
IQM
VFDRSDLSKLTESLLYFFTHFTFLCKLLNFQYYSKDLIEIENFLTDPIFYGYSFEQL
DIIKAKI
RSCAFISNAFRICCTFTCSFYCLVPFIDESRKKILPLPGWFPYDTTNYYYSTFFVQS
LSLFISA
YCNTAIDILTWKLITLASAQFEILKENLTKIDYEGGFNETKGALVRCITHHAKIVNY
TERVE
AIFSKGIFLQLFGSVIVICTTGFQLIVVPIPSVQFAVLGTYLCGMTTQVATYCYYGH
EVMTTS
DAIGMSLYLSNWYASHVKIRKIVMIFLEKTKKPTIVKAGNFITLSLATLTQILRSAY
SYFAVL
QRLYKDS
>TcasOR64
MMSDEYVKDVFIANRWMLRCAGLWTPSTRSKLVQIPYKIYAIVVFLFVNVYFTSTEF
LSLFYTHKNLYNF
IKNVNFFLTHFMGAVKVIFWFFKGHVLRDLMRTLESPEFHYEPCEGFQPGLIWRKYR
RIGFKYSLGFLAL
AHMTLSSSYIPPLLTVTLNPPPYQNGTLSPFYQKLPYFSWMPFSYSTPRSYLLALGY
QAGPMFSYAYSIV
GMDTLFMNIMNFIAAHLVILQGAFASSKMRVLDPGQMNNEMKRNCRHLQTILRVSED
LERVHRYLTLGQL
TATLFILCTSLYLISTTPASSKQFYAELVYMVAMGFQLYLYCWFGNEVTLMASEIPV
NVWKADWYDCDQS
FKKSMIFTMTRMQKPIYMTVGKFAPLTLQTFVYILRTSYSIFAVIKNTSI
>TcasOr65
MTATKSLKEIPPIYLRVHLTVLQILGIDILPVESVPQNLFYTYTALIISTMCLFTIA
EFLDMVL
NYEDIYRLTFGLCYCVTHVLGTVKMFLMLYLRKKLWGNLTTLEEGIFKPNPTRGGPE
ELQI
VNDAITMCNRQGYVFYTLVFLIIGARLLYASLANWPYDKHNYFDGNVTVIVNTKEMP
YTT
WMPFDYNDSPLYETIFAFQIFSTTVYGFYIGAADAVICGFMMLIKAQFLIVKRELET

LIERA
QKAAIAENPDNEDNFGREIERIELLDKRTQDYVAKYANECVYHHQELIALCDHAEED
FCY
LMLLQFISSLLIVCFQLFQVSTLSPDSVEFFSMVCYLLLMLFQLLCYCWHGNEVQIV
SGEL
SRYAFGINWIIMRESPKKTLLLLMMRAQRPCYFTAGKFSLLSLQTFMTIVRGAGSYF
MFLR
QMNI
>TcasOr67
MDFTIRDFDLRNSFSLERKLLLVLGFYPIRDKEKHRILHQLSAFLNLLLYYGQLLTI
IIQMVI
DRNDLSKLTDSTLYFLTLFTFLCKLFNFQYYGKDLIEVEKSLTDPIFYGYSFHKLQI
IKAKVR
SCTLVCLAFRISCTCSCFIYSVVPFIDRSGQKTLSIPGWFPYDTAKHFYITFFLQSL
SLFISAH
CNSATDTLPCKLISLATAQFELLKDNLRTIDYENSFEETKHALVKCITHHRKIVNYT
KRVETI
FSKGIFLQLFASVLVICTTGFQLVIVPFGSLKFAIHGIYLCAMTAQIAIYCYYGHDV
MITSDEI
GTSLYMSNWYASHIKIRKIMVIFLEKTKKPTIVLAGNFITLSLVTLTQILRSAYSYF
AVLRRL
YADD
>TcasOR68
MLAFVLYMLFVVSVPILEVLNLVLQEKITFKQIIDNAFMIAELGCLIPKYWPFVRNN
DRLVKCIHYFDSP
AFQPTKKKHREILQNCVKVCRAITIFFVAAVSSGYVSWSSRPISWKNHIFPTDLWLP
YDPKVAPKLYNFL
VYTYLIKILKDNLQHLGEDTEAEFNQQSVINRLPKSEIMYRKIVKCVEHHNLILDFV
KEFQYCFAQCAFS
QIAGSVVVLCVSCLQLTIVDLLSFDCLAMILFLVSMLSEVYFFCHFGTLLYEESSTI
SDAIYMGSWYDYD
KKSKQALTILMERTKRPVIVIAGKLVQLSLITFSMILRRSYSLLAVLENYNIEIN
>TcasOr70
MPSIIDISFKININVLCLAGLYLPDKFKSLYRVYTYLVYVFIVIPVPTLGCVYLLAQ
EKITFRQ
IADNLFLIAELGCFIPKYWPLVRHAERIKRCIHYFSAPIFKTDRKEHQEILDDCIKV
CHQWS
AFYFASVTAGFVSWSIRPISWENHILPTDIWLPFDPHTASSAKVASVYFYLVLGKGF
GLGIKI
LKNNLQHLGEYVDEELASLEPCRKAQLTYQKIRQCVIHHEHILAFVEEYEECFSQVA
LSQF
VGAVVIFCVSCLQLTIVEVVSLDFLAMMMYFIAMLCQVYLYCHFGTILYDESDTISD
AIYLS
KWYEFDKRSKKALCILMERLKRPMTVTCGKIFTMSLVTFTMILRRAYSLLAVLENYN

IELN
>TcasOR72
MAKLEYLTGATFTLKCAVLYPIDSNNPKIKKILYAVWAIFFILTFVTGFIQCFVFVC
INPFDLVQEAMII
MSLVFYSTTFFYFIVFYKNWQNMVALVTNINKNFHRATDNVIEKISMDQASELSDKL
AYVWTSSLAVGSV
VPVVLAIATGNLEMPMPAWFPYDYNKSPVFEITYLWQVFCLITLAIIYGASDMFFPC
ITIIIGQQFKILA
SNFKNNFYTSLIKLGAEESIVQNFSKDIKTHEFRSFYIKYGNIFKILNNAKFQTLNR
AFLKRNIKHHKLL
LRFCEDLNKILNTFLLIRVSAIVFNLIFIGFNIIISTDRTLMLGFCNYFCFGSTELF
IHTYSGQILTENA
DFLWTLYECPWYLCDVTYQKMLILVQMRVKRMVSTKAGNFFTMIAPSFIAFQRAVFS
YITLLKEVTDLGK
D
>TcasOR73
MSQVQHLKFNVLTLKLIMLWPPNGHMTLNYHLKSLSLLIIVYLSAWTCIYGILRPLF
TESDGYDVLIQRA
IATIDFIGCIYMRYCFLDKIQNVTSLIRELPTFEKFCGKVEIETTEKKVQKYSKAVT
VYWFFGNLMNCLA
PLHERTKCENLRKSQLYIERDPCGLMARCFYPFDVSKKLFPMAYFIQVYTCVVISYY
VIVLTMTLVGIMM
HILTQLKNCRNLIHNLHEEITERESLKKKIQRIIVYHIKIINLTMTRKHIFLNFTVT
ILKLSFLWPSNDN
YDQWRLVKDASLIVSLMPCALPILAHFVLQITGDVYNMVTITENLIALICIIGMIYM
TICFVKNRKLVKT
LVKNLPAFTKYSKTTDIILTDKKANLYTKIFVFYGVIGNVVYMIMPYLNIEKCQQRQ
NNDVPCGLVTRCW
FPFKFDYSPVFEIVFVHQFYTCLMVSVIILDLTMLICGFLMHITNQLKHLRGFIKRF
DCSSQKIAEDVIY
CVKFHTAIITYSEKTNEAFGTMMMLHITLTSLVISALGFEILIVDNFNDSLRFTLHL
LGWLVLLLLICYY
GQLLIDESIAVAEDIYYVPWHLAPVDVQKDIYMILMRSQKPLTLNAANIGVMSFPTF
LRVISSAYSYFTL
LLNIKS
>TcasOR76
MMESTVTRLKRMYLWPTASVTSRKPAFFLITFSCFLLYGSVMHLIVNDISMEEVHVI
ETTAGQFGVLYYL
TLFTIYRKGILEIYADLSNFTKFGKPYNFDKRNKQLNQWSRWFSVVLYFFVISVFAW
PGIFTQSCEDLNV
ALNKTEVCGVVSPVWLPFRFDYKPMKQFVYFWQSFCCLYSNGGAGTISFAMSETIEH
LILRVEDLKILFP
KIVAERSPEVRRKMLAKWVDYHLWLLSIGKLMNDTYRYSFSVIVLCAGTLFGCIGYT
VMKNASTNFNSSF

IFFGWMESVFVICVCGQRLMDAFHSVGTTVYNSEWCDTDVDFQKGVILITIRAQKPV
RIYAGPFSYVSHL
LILTVFQTSYSYINLLNASS
>TcasOR77
MKYILMKKTIAFLSVTGFWPKTKESTKTRAFCILFSSSFLLFGSLGYLIVYRKFGSD
DIDSIETATSHFG
VLYFMFFWILKRDGLVHIVNLLSDFSKFGEPRFFNDRNRQLDYLLQYCIFVLSVATG
GVFLCPIIFVKNC
EMVKQEKNLTKVCGLVSNVWAPFDYSEYPMKRVVSLWESYCCFINFGCGGIMSFTMI
KTMEHLHIRVEQL
KDMFPDVVNEKNLAVRKQKLEKWVKYHLHLYDIGELMNNTYRYCLSVIVLCVGILFG
CIGISTMQPGSSH
NSLFLFMGWFQSICILCMVGQRLLDVFLSVGVMAYDSAWYEKDVDFQKAVLMIMIRA
RRPVLIYAGPFTN
LSHLLILGVLQTSYSYINLLNAK
>TcasOR78
MGHAIMTEILTYLTLMGFWPRSPKSSKASAFLIILSTSFLFFGILFYLIVNRQFGSS
EIDSIETITSQFG
VLYYLILFTWKRNDIVEIVELLSDFSKFGKPPFFDQRSTRLNYRLSCIVLILIVANI
VVAALPVIYIDSC
HKANEQLNLTKTCGLIAPVWLPFDYNEYPRKHLVFAWEVYCCVMNYVGSGIGALTMV
GTMEHVIIRIEQL
KYIFPKILDQPNPRIREQMLKNWVRYHLALFEIGRLMNDAYKWSLSVIVLCVGALFA
CIGISMLQSTASQ
INSICLFFGWFPSIAFLCMWGQRLLDSSLSVGTAVYSSRWYDMDVAFQKSVLMILIR
SQKPIRISVGPFT
HLSMLLLLGVFQSAYSYINLLNATS
>TcasOR80
MGHVIMNEILTYLTFLGLWPRSRKSTKTVAYLIISSTSFLFFGSLFYLIAHRKFGSN
EIDSIETVTSQFG
ILYYWVLFTLKREGTVEIVERLSDFSKFGKPRFFDQRNRRLNYLLSYFVLVLMVAIG
GVVALPVVYIDSC
HKANERLNLTKTCGLIAPVWLPFDYNEYPRKNFVFAWEVYCCIMTYACCGIAALVLV
GTMEHLIIRFEQL
KLMFPEILDEPDRHTRQQKLKNWIEYHLTLFDIGKLMTSNYTYCLSVIVLCVGILFG
CIGVSTMQSASSH
NSVFLFFGWFQSIGVLCIWGQRLLDTCLSVGIAVYSSRWYDMDVSFQKSVLMILIRS
QKPILIYAGPFSY
LSHLLILSVFQTAYSYINLLGAKG
>TcasOR84
MVLLPTDFGRFRHHPHIRSAGFDAPLEAMEEGMDISEDLGIFAAIIGLVTMMVLYVV
KQKDLSSLLQDLS
NFEKFGKPPNFDKVNKRLDFLVKFVFSYAIFGAVVYNLMRIIEIPHCKRNRKIREVC
GVFVPLWTPFDAD

YPPVLVLVASVVFIVVILIDKVTLLVSLQVLEISCHIRLRIEQLNAMILNCFNGDLQ
SSRKRLNDCVQYH
IDIISYSERFNNCFTNGMFIHLATTGIIIGCLENQNVEGATPGGVLHLFGWIISLFT
SCLAGQILLDSST
SVADALYNSQWYTADVKLRKNLILMIQRSQKPLFLATGAFNIMSFALFVTSSMTEEK
ELRLCLWSCYYLK
LSLMWPLKREEFKSSKGLYLRLLVFVIISGSTFTAMIFMHLYKSLKVGSYDVSEDLA
ILASNIGYVLMMT
MYVSRQKDLELLLLDLSDFKTYGKPPNFDKVRKRMDLYAHLIFFYSMFGSFVYNMDK
IILIDKCKEARRI
NEVCGSAIPFWTPFETEDLFTLTLVITYVLINIFVVVKVAMTVSVQVLEISSHINLR
IEQLKIFIAGCFD
RDFKASRERLDFCIRYHNVIIDFSERFSRCFSYVMFIHLAITGIIIGCLENQIVQEH
QPEAMLHMGGWST
ATFIACYGGQLLMDASTSIADEFYNCPWYEADVKMRKDLILIIILRAQKALFVSTGPF
NVLSFALFVSIMK
LSYSIFTVLS
>TcasOR86
MALNQEDAICSKSCFYLRYSFLWPEEAPTRSFYAKFILVLILSFLTAFLPLFIHFLI
LVERGLDPSEDLF
VIISYTGFALIMIIYVIHVKKTSYLIVQLSDFEKFGKPRGFDYWDKKFRLISSGVYY
YVLIASSGLNLGR
WVGMAECRKERDFQVCGIVIPYWLPWKVDSWLFFILLDLYVLKMTLVVNCALFLIII
QILEITTHLKLRI
DHLKEMLVKCFDSDSQTNRKQLVNCIRYHTYIINCSKLFKKCFTHAMFSLIVTMALS
CGCLESQVVKFDL
WALPPISAWIFILFIACMAGQILMNASLSIGDAGYHSKWYQTDANFRKYLILVLMRS
HKALVLSAGPFNI
LCFELFVAIMKFSYSVFMLLNQN
>TcasOR87
MKHVIMDELLIFLTFLGLWPRTPTSPKIISYLMIYSTSFLFFGSSIYLILHRKFGSD
EIDTIEIITSQFG
VLYYLTLLVVKRDGITKIVNLLSDFSKFGKPPLFDQRSRRLNLLLRLFVTVLLAATV
AIVSVPVVFINSC
NKQNLQLNATKICGLAAPVWLPFDYTQNPRKYFVSAMEIYCATMNYAGSGSGAFLVI
GTMEHLVIRIEHL
KNMFPEILNEPDKQIREKRLKKWIEYHLSIFEIGELMNETYKWPLSVIVLCVGILFG
CIGVSTMQSVSFQ
NSSVFLFFGWFQSIFVLCFWGQRLLDSCLSIRKAVYNSKWHEMDVSFQKSVLMILIR
SERPVLIHAGPFS
YLSNLLVLGVLQTAYSYINLLNARS
>TcasOR88
MTEEKQLRICLSSCFFLKWSFMWPTKSEEFRTSKGLYFRLLAFVIISGLTFTAMIVM
HLLKSVEAGDYDI

SEDIAILATNTGYILMMLLYIIRQKDLESLLVDLSSFKKYQKPPKFDEVNRKLEWCT
RMVFGYCVFGSVF
YNLVKILAIPSCKKSRRINEVCGVAIPYWVWFDTENWSIKLPLILHTFLVIIIVDKV
TLLVSLQVLEIAC
NIKLRLDQLNCMLVSCFDGDVEASRRRLNECIKYHKEIISYSEIFSKCFSIEMFTHL
TTTGIICGCLENQ
VVQEHRPEAILHIGGWITAIFVSSFGGQILIDSSLSVAEAAYSSAWYEADVSLRKDL
ILVILRAQKALFV
STGPFNVLSFALFVSIMKMSYSILTILQ
>TcasOR92
MKNQEIKICRATLTVLKYSLIWPSEADEMNPGKWYYIRVVTFILFTCPWVLSVFMHL
IVSIRNNADIHLS
EDVALMVAFTGVYYMTIIYVKKQPKVAFLLRDLSYFQFGKPPGFDETERILGFLSKL
TFCYSVMAVVIYN
YIKYRQKPECERMNKLKGLKENCGMLTPTWWPFEINYSPAFQLIFLYIFTSTQVMMK
LSLMISFNVLEMA
HHIILRINHLKTMILESLDEQDYEASKRKIKTCILYHLEILGFAERMDDCFSNGMFA
HLTITAAICGCLE
KQFVDGDNQLGSLLHIFGWILALFLACLGGQHLINASETISDAIWSSKWYDADLRLR
KDLIFMMARSQVG
LYLNVGGFGILSYALFLSVIKMSYSILAMLTS
>TcasOR93
MTNLEIKICRATLKILKYSLIWPNEADEMNPGKWYYIRVATFLLITSLWVLSVFMHI
VMSIIHDADVHLS
EDVAFCVAFCGLYYMTMIYVKNQPKVALLLRDLSKFQFGKPPGFEEKERILGFLSQF
FFYYCVMAVMVYN
LVKLLQKPDCEKMNEIKGLKENCGLLTPTWLPFDINYFPAFHLTFLYVFISTQILMK
LALIISFNALEMA
YHVILRIDHLKIMITECLDQRNYEVSRRKLKTCILYHLEILSLSNRLNDCFSNIMFA
HLTITAAICGCLE
KQFVDGDNRLGALLHVCGWISALFVACIGGQHLLNASLSIPDAIWSSKWYEADVRIR
KDLLFMMAKSQVG
LHLNVGSFGVLSFSVFFSVLKMSYSILAMLTS
>TcasOR95
MVVKESEIKVSRVTRKILQYSLIWPKEGDEINPGKWYYIRIFTFLSFTSLWCIAICM
HFIIVLKDKIDWD
VTEEIAIIIAIYGTYYMVLAYVKNQKKAARILRDLSNFERFGVPPGFEEEEKRLKVY
IIGIFIYAFLTIT
FYNFFKLSQKGACERFNEEHHLDENCGLLSPVWIPFKVDRFPQFELVFLYLFTCCHL
LMKLPLVVSYNAL
EMVHHIILRINHLKIMITECFDEPEYEISRRKLTQCILYHIEILEFATRVDDCFSNC
MFAHLTLTGAICA
CLEKQIVAGISRFGAILHFIGWILALFIGCLGGQHFINASDTIPESIWASKWYNANL
RLRKDLLLMMMRS

QRDLHITAGPFGVVSYALFLSVLKMSYSILCVLTS
>TcasOR105
MKPALKLANVLGLDPLRNDNYTQLKKMFCALCIVSLFVSAYLEFFSNFTTFETYETA
PESLIPHFQTMFK
MYSLIFSRTEIVELIQMAEQFYKFSQCDERKKLTKLYKRVDLFFYVYASLVAAACVL
FAIVTLIFKPGKP
IFLCYGGLHGLESPEFEIYLVVDLIGIVIISVTVPAFDGLFFYFALYIYTEFKLLKI
AFKTMSGQELREA
VKHHDFLLKYIKKLNSVYSPIFLYQFFCNLLAICFCLFMLSRSGIPPEMVSFSKYFL
CLLAFLVQSYTFC
SIGDLITELSEDVSNAIFYTDWLDDEAYENKTARLIIMSRAQNPVMLTIGKFANMNL
RTFILIVRNAYSF
LAFVNHALN
>TcasOR131
MEYLKYCQTYIRGCGLASNSSPFRKILAIYFFLPPCFLIIAFSVYELWDANNNDIFL
VIEVLECISSYTQ
LTIRKYMIFTQNELMVEIINDCDQLWSFDTCGPELGKKFKQRMKNCWIMVKALVTCG
FTTFILMCISARA
DRDNLLPFLCWIPDFPYATEVLFLSQFMLLMELLYYVMATDGFYLLVCMDIHIQFEM
MQEMLKTIQFDVI
SEKESWEKLTELAKHHNRMLHQKLNQVFSKYYVVQYFMTVAAMTVQTYTLKYRMVNI
QTALKSIMYTFSL
MFQSAYYLFPASNIEIEAENFSTEIYFLNWQDHEDVKIRKHILFMLMKSQENLELMG
EGMVHINRNEYLL
MFRLAFTIATLLDGLNQL
>TcasOR133
MEYLKYCQTYIKGTGLASNSSLFRNILAKYFFLPPCFLIIAFSIHELWDTNNNDVSL
VTEVLECVASYTQ
LIIRKYIVFTQSDLMVEIINDCGKLWPFDMFGSELGKKFKQQMKTCWTLVKFLVVCG
FATFFLMCISARA
AERDNSLPFLCWVPDFPYATELLFFLQFMLLLELLYYVLATDAFYILICMDIQIQFE
MMGKMLKSIKFGE
ISEKECWDKLVELAKQHDRMLHQKLNQVYSKYYVVQYFMTVGSMTVQAYNLKYRMVN
IQTALKSIVYTFS
LMFQCGYYFFPASNIEIEAENFSTEIYFLNWQNIGNIKIRKHILFMLMKSQENLAMM
GEGMVHVNRNECL
MMFRLAFTIATLLDGLNQV
>TcasOR135
MEYLRYCQSFVVGTGLVTNSPSFRKFLGWCFLLPLSLIVFAFSIYKIRDTDNDIFLM
IEVMESISSFTQL
MIRKYIIFIQGELMLEIFNECENLWSFDLFGPQLSEKFKQQMKNCWTLAKVLITSGF
ITIVLMCISALTD
KTKSLPFICWVPNFSYAHELIFLSQFILLIELLYYVVATDGFYLLICMDIQIQYKMM
GKMLKSVQFGVIS

EEESWEKLVELANHHNKMLHEKLNKVFSKYYIIQYAISVSAMSAQVYTLMYSKVVIE
TALKSICYTISLL
LQVAYYFFPASNMEIEAEKFSTKIYFLNWQDNADAKIRKHILFMLLKSQKSLEMWGE
GMLHINRNEYLLI
FRLGFTIATLLSGFK
>TcasOR136
MEYLKYCQSYIIGSGLASNSPVFRQFLARYLFFPLSLILVSLSIYIIKDANNDIYFL
TEVMESLASYTQL
LIRKYMIFTQSKLMVEIINDCENLWSLELFGPELGKKFKQQMKNCWTFVNVLVTSGF
STVLLICITTLTD
KEKSLPFVCWIPGFPHATELIFLTQFVLLMNGLYYIKLTDAFYLLVCMDIQIQFKMM
GKMLKTIHFGLLS
EKESWEKLVELAKHHNKILHKKLNKVYSKYYVVQYVMSVTAMTAQAYTLKYIEVNIQ
LALKSIMYTCSLL
LQGALYFFPASNIEIEAENFSTEIYFLNWQDHGDVKIRKHILFMLLKSQENLEMMGE
GMMHINRNEYLMM
FRLSFTIATLLGGLNQL
>TcasOR160
MSGKTKRITTKTIHLSNPYSSFKKVFSDFAYSKIMIFYTIATLAFHMLSLFLQIYYV
ATNYSVELICRYG
PMMCLAIYVVTAKVVGVFYYKTFTMLENQCLFVLWKTCNSSPTTQRLILNKSLKMNQ
KLHLALMSYFLLA
IVMLPTWGDLNELFIFSQVYERYFKFWAPVLYYFYISTFLWCSYYSFHLPGCILYLT
LLLDVQIKLINDK
ITEIDQNFSQNEISETLRLCISHHIALKRWMSTLAKMVNSVMPVFVLLGALSTVAVS
FFVLNTLQNTTMI
LKIRLAILTVCNFVIVSTFAELGQIFSDQNNSLFEHLIDCPWYLWNVKNRKILLMFM
ANCMKPKTFSWGG
ITLDYSFAISILKTSFSYALILFKLRGETIRN
>TcasOR165
MSDNTKKATTKSLDLTNPYSSLKKVFINFAYSKIMIVYTSATLIFHILSLMLEIYYL
ATNFSVELICRYG
CMMCLITYMVTAKFFGMLFSNQFKFLEEQCLLDFWKAFNSGPTTQRLILKESSKMNR
KIHLALTFYVILA
IIMLPIWEDVNDFFMFSQVYENYFANWAPVLYYFYISTFVWCSYYSFHFAGVIMYLT
LLLDLQFRLINDK
ITEIDQNSTQNEICGTLRLCISHHIALKRWMNKLANSVDTAMPVFILLGALSTIAVS
FFVLNTLQSTSVI
LKIRLATITVCNLIVVATFAELGQIFSDQNNSLLEHLMDSPWYLWDVENRKTLLMFM
ANCMKPKTFSWGG
ITLDYSFALSIFKTSFSYALVLYQLRGNTF
>TcasOr173
MSNVTFDEPFMFFKKVFFDFGYCTSIRFYHLFCFTFHICCQIIENYFFLTEYLSADF
VTRYGC

PMIVIGYTIVCEFFLMKWEEPIKELLDERETIFWEIDSNSKPQILKYSSKVNRIYKF
FLFWVV
ILAIFLLPFWGDLDETFFIIRIQKIYFGKWSTLFYTLYVSTLPFMVYSGIRFPIVTL
YLIMQSH
LQILILSQKIGQISQNNNHMDDVSKFHDVGYQKKIRTSLHVCMCRHVTLKQWISKIL
QIVQ
KAIPVYFSLAIIVLVTVMFCILYNVESASTTTIFKIRLVLVGICGAVVLFTFSETGQ
LLSDDTS
QVFDTLAASPWHEWDPKNRKTLLMFLLNSLKPVKIYWGGFALDYQLGGSVIKTTFSY
AL
VLFNLRKD
>TcasOr175
MRNFQDSDDPFIFIRKVFVGFGCSTIIMYYSRLIFIFHTLSLLLESYHVITNFSLDI
ITQYGSA
MSLMLYSITSQFLLICEQNLITEVVEECKSFFWTMDFLSFIKQTQILKDMTKIKRKM
YLSWI
WFVVFGIALLPVWGDYNEMFLFPFIYQTYFGNWSPLFYYFHASSFPFLAYIAIRIPA
FILYLT
LALHFQTLLLNQKILQIPQNKSGNQEDIFRNLCSCISHHVALKKFVTKTQQSIQKMI
PVYFV
LAILCLVAVMYSCLNSLAMSTSNHFKVRGFFGGVCGVVVLYTFAEAGQLQADTTGEV
FNT
LMQCSWYNWNNRNQKILLLFMVNSLKPSYIDWGGVIVGYSFGSSVIKTCYSYALVLY
KL
KISKEQNVTF
>TcasOr198
MPNVTNKRQKRLFSKTRTKSEDPFVMIKDVFVDGGYHPVTKMLNYICLVIHSCSLLL
ELN
YFVHNYHFDLMMKYCCAMSLMGYIIATMLFAIFQEHSAIDLTKDILSLFWPIDYCGP
RVKE
EIVKKATKINRIHYIVLLFAGALGITMFPIWGDQKEWFLCVQVYQHYFGKWSKIPYY
VYFF
TYPMLAFSSVRLPFMTMYAIVQIRMQVYLLHQHISEISGEYVYDMKNLQILCDQNYQ
NEIYDK
MRLIISHHIMLKRWMRKLVHTVQISMPVFVLLGTMTSISVLFYAIYSFHNINFILKV
RL
ISVSVCTVLVVYMFSEAGQALSTETTGVFDLLMTCPWYVWNIKNRRILLIFMANSLE
PMT
FSLAGVTLDYRFALGMLRTSCSYSLILYKLKTGI
>TcasOr199
MSMTRSKYFQDSDDPFSFIRKIFIDYGYSKKINYYNRVTFTFNTCSILLESYYMITN
FSLD
LFVRYGGALSLMLYHVVTQFLVIAKQKSLEQLLEESKSYFWKADIFNSSVKNQILKS
CNHMQ

RKFCLLWTPFVACGIVLLPVWGDFTESHIFPQVYKAYFGHWSPIFYYFCISSYPFAV
YTSIRL
PAIALYLFLQAHFQIVLLNQKILQISKNNDLDETTIFENMEYQKTIYRNLRSCISQH
VALQK
YITRILVSIQKAIPVYFCLAVLCLIAVIFFVLNNLNMSASNHFKARIFVSGVCGSLI
LYTFTEA
GQLLADTTGDIFNTLMQCPWYYWNIKNRTVFMIFMLHSLNPLKIDWGGFTLGYSFGG
AVI
RTCCSYAVGLYNLRESKY
>TcasOr202
MVEFKDPVIMLKTIFLVNVKEMTKFSQVFLAIFTFYSLVHCVQMYYLYKNFDVNLLI
KYAP
ATTATLFVSNTKLLSSSLNIMPIFSVVSETKLLRITTFIDKTFWPLDSIRKEARIKL
ERKCRAI
NISIYCILLLLSVAVFSNFPCFGRQDDFFLCIKIFKEYFGQWSSIPNYIYFTLFPIF
CYPYFRIAF
SFVYAILETQLQFSLIEEYLFEVYQMVDLNWKYLQDPRYQQEIGKSLQLCIEHHTAL
KKLI
HSIVNITLTGMPIFLLFGIGLFVSCFVFIINFGDTMTMILKLKTPLLLYVATMLSMT
LLMCWN
GQQVIDVTSRIFYTLVRAPFYFWNLYNMKVLLMFITNCTRNENIVLAGICLDYTLSV
SILRI
SVFYTLGLLELRNHSFD
>TcasOr208
MEKLDDPFITLRKMVFIEAKNCKIARFCDVLLIVLYSLAQCLHLYYMCQNFNLNLLI
RYGPI
LISCLLVIVTAVISVGLDKEIFEVYTVCWKISWPLNFLRKDAQTKLRRKCQIINRGI
LCSAL
LFLTTVISTFPCFGSVRDFFICVEVYEKYFGEWSFIPYYFYFAAAPFLYYHFFRVCY
VFAYAF
LHAQLQYFLIEEYLLETYQTNDLKGWKYLQDTRYQQEIGKSLLLCITHHIALKKYVK
ISQN
LVLIGMPFFLVLGVLLLINSFGFITNFGDTMSNILKIRILIFVACGVSITIVMCWIG
QQLIDVTS
EIFVTLGGAPWYFWNRDNNNILLMFLTNCTKNESFILAGICVNYQLFFSIVRLTVSY
TLVLY
NLRESGFI
>TcasOr214
MAEFIDPFLMLRALVSVKFNDYTSLKLCNILLITIYSLIHCLLIHYMFKNLDINLAV
RYVPMI
MFLTLVIVGAIFSVAIEKDILEAQVFLFKANWSLEMIRKDAQLKLERKCRIINICIL
CVLLLIF
ATITINAPLFGSQRELFICIQVFEEYFGKWSFIPYYFYFAAFPFLYYDFLKLWMSFV
YAVLEV

QLQLTLVEEYLFETYQINSSKEWKNLQDTHYQQQIKKSLRLCITHHIALKKFVKMTV
DLTI
KVMPFYLTIGVLILISFFSFIINFADSMSNILKIRIFMFSASIVSITVLLSWIGQQL
VDVTSGIFW
SLVGAPWYFWNLENVKTLLIFLMNCTKNESIVLAGICIDYSLGISILRLSVSYALGL
FNLRK
SSLD
>TcasOr217
MVEFKDPFIVLRKIFFIKFNNCKLTLFLNISIIVFFSLVLCLQICYLMKNFNLNLLF
RYGPVTV
LFTLVTVTAVLSLTLEREIFMAITFFFKFCWSLNIIRNDAQITLKRKCRCVNIGLLC
ILLIIILIAI
VIGFPCFGSQKDFFICLEVFEEYFGEWSFIPYYFYFAASPFLCYHFLRICFTFVYAI
LEAQLQ
YLIIAEYLFEIYQTNPSKRWKYLQDTRYQQQIGKSLRLSIVHHVVLKKFLKRTLHLT
KIGMP
FFLVLGILLLTSSFAFIMNLGDTMSNILKIRIFLFTTSVLCITILLCWIGQQLIDVT
SQIFVSLSG
APWYFWNLENIKILLMFLTNCTKNESIILAGICLDYKLFVSVARLTVSYAVVLFKLH
KSSLV
>TcasOr222
MDKRDDPFIILRKMIFIEAKNCKIAKFCDAFLILFYSLVQLLDIYYMSKNFSISLLI
RYSPITI
MYLLIIIAAVISVGLDKEIIEAYTVCCKIRWPMNVVKKQTQIKLKKKCQIINAGLSC
TVPLFL
VTIISTFPYFGSERDLFICVEVFEAYFGEWSFIPYYFCFAASPFFYYHFFRITFVLV
YAFLHAQ
LQYLLIEEYLFETYETDEAKGWKYLQDTRYQQEIGKSLQLCISQHIALKQFVKKTVD
LVLI
GMPFFLVFGVLLLTSLLAFITNFEDITSNILKIRILLAAGCSLCITIVFCWIGQQLI
NVTSDIFFS
LGGASWYFWNRDNMKTLLMFLINCTENESVVFAGICLNYELFLSVVRLTVSYTLVLY
NLQ
KQ
>TcasOr229
MSARPLHLRNFPYYFLKVLVFDFEQYSAGKVLSYFCAIVHSISIFLQMHYLVKNFTK
ETMF
QYGCVLTVLTYCVVALFFAIASGNFVEKLESEISSFVWPLDICGEDVKAAILKRAFY
TSLVA
YITIIAFPIFSVIMFPVLGDQSDMFLCVRVFNEYFTKWSQIPISLYFYSFPVIAFSG
IRLPGMLL
YAILITHIQMFLLNRRIEQISELSNQRRVFETLCSCIELQAKLKRLIRNVFQLVYIA
MPIFILLG
AVSSVFVLFFVVNSLETASYFLVLRMGCFFGANVLVVFIFSQSGQSFSDETGRIFDT

LVMCS
WYNWDKRNKKVLLMFLANSLEPMSITIAGITLDYKFALAMLRTSCSYALVLYQMKN
>TcasOr231
MEVTGKKVTSGDPFITLKKLYIDLGYHKVTKLVNVFFIVFYGFVYLLQIYYLIVHFN
FEIIA
KYSTILLLSTYLFNVMIFSIIYEKCILDAYKTFSQIAWPCDNASKPLQIIILQRSKT
IKYLNYFF
LGFIFFMACINWPWLGDQNDFLLCIQVFKKYFGSWSPLFLLFYYLGFPIIGYSAARI
FFIILY
GVLHLELQIRLITELFCKISRNATLEDVRNAKYQRDVYWTLREGIRHDTALKKVLFD
LNKE
VKHGIPVFLIVTLLCSVSIFFFAITYLESMGFGIQIRTIAFVGAVIFVLFSYSLLGQ
HLINQTSL
LFDQLYECPWYTWNVKNRAIYLNFMLNTVRPIKITYAGICIDSRFFLSITRIILSNA
FMLYQL
RNS
>TcasOr238
MMSEFNDPFIVWRMIFMINFKKHKITKFCEIVLIVIFSLIHCLLLYYMFTNFSVNLL
IRYGPT
TLFGIFIIAVTIFSVALEKELSGGIDILDEICWPFNMIGKEAQLKLERKCRMRNMCT
AFVVLII
LTTIIVSYPCFGDQRDFFICIKVFEEYFGEWWSIPYYFYFITIPFFCYNYHKLCFTF
VYAVLET
ELQFFLIEEYLLETFKMGYLKRWKYLENTQYQQELGKSLRFTIAHHNALKKMVKAIV
NV
TVNGMPLFLLLGFLLYISCFTFVINLADSMTNILKIRIFVCGASCVSVTVLLCWNGQ
QIIDV
TNSIFSTLTGAPWYFWDVDNVKILLIFITNCTKNDSITMAGICLDYKLFASLLRISF
SYALVL
FNLRKSSLS
>TcasOr259
MMPEFNDPFLVWRMIFTINFKKFKITKFCEIVLIVIYALVHCLLLYYTFTNFSANLL
IRYGPV
MIFYIFMIAATTFSIALEAELSEVITFLDEICWPLNMIAEDAQVKLQRKCRIINMCI
AFLVLII
LSAIIVNYPFFGDQRDFFICVRVFEEYFGEWSFIPYYFYFAASPFFYYNYFKLCFTF
VYAVLE
AGLQFFLIEGYLLQTYKVDYLKRWKCLKDNRYQQELGKSLRLCIVHHIALKKLVKMI
VNL
TVNGMPIFLLLGSLLYISCFTFTINLVNSLTNILKTRIILMGASCVGVTVLLCWNGQ
QIIDVTS
SIFTTLVGAPWYFWNLDNIKILLMFITNCTKNDKIVLAGICLDYKLFASILRISFSY
ALVLF
NLRKASVS

>TcasOr263
MWNNNPFIVIRTIFLDINNYKIVKFCYVSLTVFYSLVHCLQFYYIIKNFNLNLIIRY
GFITSLL
SYVLAAGILSLVVEKRIRKTQIFFDEIGWSLNIVGKDAEMKLEKKCKLINISIYAIM
LFLVIT
LLVNLPFVGSQRDLFLSIQVFEEYFGKWSEILDRLYFTLAPFLSYHGARLSFTCIYA
ILQVQV
QFSLIGEYLFETYQVDDSKSWKYLQDTRYQHDIGESLRLCVEHHVALKKSIKMMVDV
ALT
CLPFLVLLGLSTLISCLAFIMNFWDTMDNILKLRIFMWAAWIVLITIMFCRSGQQLI
DATSDI
FFTLGGAPWYYWNLDNIKILLTFMANSTKNDSISLAGICLDYPLFVSVANTTVSYAL
VLYN
LRESSLDSSNKK
>TcasOr315
MTLVRKLQAAATNAFEIRIKDDILAELFNWPFLVLDSKWSTKFAVFLTVYCVFETLA
CALV
YSTLDVNMMGTYAIVIARFATTFCSFFSFFTKRKQYFEIINENFPHFWPLQSLGKST
FNRIK
MRASSVKFYSFLNVVVMLIGAVILISFTQDESEVYLSVKIYKDYVNKWTTGFVMFFY
VSFI
YIGLVVAAISFVLTYTAFHLIFQCFLLNQKLKQINDSIVENEQKQAKFDEKYQSFIY
KELISC
VKLHQRLILFGKRINHLVYAPLLVYIFGGIVVGVALIYYLKSSVQHIFTSLILLLIA
LINST
TFVINGQMLENEAENIYISLTNLPWYSLNVQNRRVVYVMLMQSQKIIHMSASGLVSL
NYQLTI
VFFRCIYTGMTFLVNVGL
>TcasOr316
MTLMRKLQTAIRNLFEIQIKDDILAELLDWPTLVLFSKWPKNFAIFSTIYCVFDTLV
CTLVY
STLDVEMLGKYAIFIAKSTIALCSFFSFFAKRKQYHKIINENFPHFWQLQSMGESTF
DQMK
KIATTVKFYSCLSVVAMLIGAVILILFTEDESEIYLSVKIYKDYVNKWTTGYIMFFY
ASFLYI
GIVTAAVVFGLTYIVFHLIFQCFLLNQKLKLINSYIVKNGQKLVKLEERNQNFIYKE
LISCVK
LHQRLIYFSNQINDLLYAPIFMYTFSGIVVGVALIYFLKTSIQYILTSLVLSIVSLI
ITTTFVING
QLLEDETENIIISLTNLPWYSLNVQNRRVVYVMLMQSQKIIHMSASGIVSLNYQLTI
VLFRC
IYTAMTFLVNMGL
>McarOr1
MLKFKVVGLVADLMPNIRLIQASGHFMFNYHADNSGALHTLRLGYSCMNLVFVLLQY

GA
IFGNLVAEKDDVNDLAANTITVLFFTHCVTKFVYFAVRSKLFYRTLGIWNQANSHPL
FVES
NNRYHALALKKMRILLICVGMTTILSAAAWTGITFVGESVHTIKDPNNENETITEEI
PRLLI
KSWYPWDAMSGMAYYASLVFQVYYVFFSLSQSNLLDSLFCSWLIFACEQLQHLKEIM
KPL
MELSASLDTYVPKSADLFRAPSATSQDNLIENEYNAKNEELNLKGIYNTRQELGGHF
RSG
TLQTFGQGGGGVGPNGLTKKQELMVRSAIKYWVERHKHVVRLVTAIGDAYGVALLLH
M
LTATVMLTLLAYQATKINGVNTYAASVIGYLVYSLAQVFHFCIFGNRLIEESSSVME
AAYSC
HWYDGSEEAKTFVQIVCQQCQKAMSISGAKFFTISLDLFASVLGAVVTYFMVLVQLK
>McarOr2
MKRNMSYDNFDYTVFFTHNILMYKIFGFWRPDDDMKREKLYNCYTLICTIIWLLFLA
SQY
IFIITNIQNVDEVTATSFVTITFSINLIKMLAIYRNMNRIKQLIKDMNLPMFQAKCA
RHRDIID
YTRIYTIFFYICLYFGNTDRHYFWTIVPFIGDERATLTHGWFPYNETKSVNYEITYV
FQTTV
SVWNTMLCLNLDTFTGSLLILIGLQCDLLCVTLENLGDFHVENGVLCENSEEYQSSL
VND
KVKFSKTMTENLVVCIKHHKEIMRVSKDVEDIHRVSVFILFLGGALIMCCCLFQLSV
VPIGS
IEFFMLLFFLISILTEQFIYCWFGNEVIQKSSRILHSAYCTPWLDCDINFQKVLLQL
MTQTYR
PITLKAGGLFTISISVYISVIRTSYSYFTLLKK
>McarOr3
MSQKVDPQYFKKHLKWLTWLGIDIIPIEKVWYAIPYKLYAFVLLVYVYLYSLLEIID
IVKSS
DFNSMTFGLSYSVTHILGAAKITILILKKKILRDMLIRLEQGYFVPNKARGGEKEQQ
LVNASVIRANLHADIFNTLVYLIIGIRCLYAIFDKGVYVEVLDEKLNVTTLKHIRTL
PYKAWLPVD
LNKSPAYEFMFIIQASCLVLYGYYIGFLDSLIYGMMIHMNNQYLILRNILEHYVELA
KNIVL
NRNPNSVTDDTSTDYIKLHNGIERQKTLAGPVLDVIENIAYHCAKYHLAIIDYCDDI
EKEFS
NLMLLQFLSSLYILCFQLFQLSLVTNYFSFDCISMCLYLILMMYQLFCYCWYGNAVM
IQSL
DISSVIYNTDWLVTNESTKKCLLLMMMRAQRPIIFTAGKFAFLSLPTYMAIVRGSAS
YFMV
LQQMQ
>McarOr4

MAPSYVFDLPKAFEFEKKLLLYTGLYPNTGLVNKYIYYLSGLFHIGITILIEISLII
VISIHIDNL
STITDALMFFVTQIALTWKLTNVCIKRKVFCEIEEILSQPIFYNLSQECENIIHYYV
KFSHRFA
RCFRIICIMVCATNGTLPLVGGKLGHAMLLGWNPWDSEDRIKYYLNSTFQLTALCVS
ACIN
STIDILTVILLAIATAQIEILKNNLVNIKYGEKEAKKLFNENVRLHYEILRFVNAVD
RSLSSGI
LSQIFGSVLVICVTCFQLIIVSVQSIQGAFLLIYLLCMTFQVGLYCWFGHYLIDSSD
TIIQAVY
MSDWYEANNSLRKAVIIFMERCKQPIVLRIGGLFPLSLGTFTSIMRSSYSYFAVLRK
WYEPE
>McarOr5
MTDKGYTPHFFRTNEIIEVYTGAWMYNENLVAPGKKWLLYIWSVLIYIGAVFFLFLE
FLKL
RDTMKVSNDFIRQCGLISCHSLCVVKFVILVLRHRKIKRLMDTLQDKKYQYEPLGDF
SPG
QRFDEARKLTHWCTIGVFCLYSCAAVSAHISAEVLINKDAKRERFDGNITCYEYMTF
YFAI
PFPSDTKAQCEMSFIFMHFCIDIYAWFAAGHDSFYAALLNCLRVQVDILCDAFRTIR
PRVLK
RLELPQDLSIFHDDDFPKLEEALYRELTHLTEHLMILLRVADDLEEVFNLITLAQTV
SSLIVF
ASCLFITSTIPLSSPEFFAQVEYFTCMLIELSLFCWFGSAATRASEAISPAIYESDW
YGTSKRF
KQSVLIIMCRMQNPIYLSIGKFCPLKLDTIVMVFKCSFSYYTVFKAVGE
>McarOr6
MPLSVFFIYFLIIILSSMQPFAAIAYQFYVGIEDMNIISEAFIGISDLVGFLFIYICF
RKHRGLIKE
TIKASAVFLKYCSPNVMEKAEEEVQTYTKGLLIYFSIGLTFNGLIPLYDYENCDQRR
LSDY
YRAHDPCGMPIRIWVPFDARKPVIYYLVFFLHANACLNICYGVLCITMTLVGLLIHI
TAQIK
NLRQNLLQVFDELPEDGDCYSETLVLKLENKLKFCVKYHIIIINYTDQVFAAFNLML
IVHIS
LTSLIFGVLGYQIVTVEDFTEKLRYVMHLGGWIALLFLTCYYGQLILDESTTVANAA
YQSK
WYNGPTYLRKNLCLIIMRSQKPLKLRAASIGVISLETFLSVIKTAYSYFALLLSIAE
>McarOr7PAR
VLLYCTTEFAFLCKLMNFVLSKKEIIELEAILESRLFTVDTPEEEAIIKTSTRQIRK
LANIYKT
LCFLSVTFYALFPLADGGREAQKLPLPGWFPFNVCNHYYEVFIFEAIGIGLCAWFNS
ALDL
LVVIMMILGKAQFELLRHRLMNIAIYGEDGERRRRVKMCAQHYKSILRFVMLTESIY

SNGI
FVQFMSSGIVICFTGFQMLIISLKSIQFVQRILYLSCMMYQIVMYCWYGQVLTDSSN
KITEA
CYLADWINCNVILRKSLLIIMERAKYPAKIRAANIFTVNLETLLTILRSSYSYFALI
YSIYDTK
NETK
>McarOr8
MTVPYADDFFHTNRWILYIGGLWWPDTYKSIYHKILYMSYCAANFLFCNLYFTPTEV
LSLA
STYKSIYHLIKNFSLSQMHVLGFTKVLFFVFKGYKMKAIISVLEDKKLHYEDCDEVN
FHP
GMLTNKYKKIGRVAGIIYLVLPLVVILLAYTLSAIAALRYVEGDSNHQLPERLPFYS
WMPFS
YDTPKKHLIALVYQATPLVSYSFSVIGMDFLFANIMNCIAMNFTIIQQAFRTIRERA
AIRVKE
PLKVKDELYNSEPLQRELNKEMRKIIQHLQTVYRMCDELEDVHKYLTLAQTLSQLFI
LCAS
FYLTSITPFGNQLVIEGIFMIMVISPIVFYCWFGDEVTHQGGEISVAIWQSDWLGAT
KSFKTC
MIINMIRTQKPVYLTTGKFAPLTLATLVSIFKASYSFFTVLKNTSNQ
>McarOr9
MTILGIWPVKANWFKKLRLYEVYHRISFWILFEFYYNADNKNFVQVMGKNFSETAEV
LG
VVIVLLITSFKVKICTSPKIKNLLQQIEDAEKIIIETTELDTRNIYNQHIKMSTKEN
TVQLMIG
VLAISLYSVRPILANRGLEPESKNKMFIFASWFPFDEQTYYAPAYLIQFISGLYSTG
YTISTTM
FLFNAMIFARCEIKILQNQFVNFTYYVKKDAKDNCRTYEESQKVALEDRILKHRKII
NFVN
TLDTSFKTILLLDFTVTSFQFSMVVIQMVQRSQLDVAVVSMVMYLSTLALQLYLVYS
NAH
EIIIESNKIAQAVFESEWYDLPNDVKKAFVIIMLRAQKPLYLSIGPLYQVRCDMLFK
ILHALY
SYICHFLEVNYIVTIV
>McarOr10
MSSYPKKLFFLNRWILCCVGMWPPDNQNKLFRFLYKVYAIAAFFYIMVLYNVLEIIS
LIYT
YNDTVSFMKNVSAVCVHLAGAAKSVIFYLRGDKVVEMMITLESEELRYEDCEARNFY
PG
KISKSCKITVAKLTALCFVMVHIVLLSSFIPPILQILLCIIKRDATVLPDRLPYLIW
IPFKMDTV
SRFTLALVFQIFGMFGGAYNISGMDSIFLGLMHCISQNLVIIQGAFLTIKERSVKRI
KGPALA
ADRLNNSECLNAAMNSEMRKVSRNLQTIFNVCWDLEREYKYLMLLQVLITLLILCSS

LYT
FSSATPNSKLFYTEIIYISAMMFEPFMYCWFGNEVTHKADEMSNSVYQCDWLGTDKK
FKT
SLILNLTRSMKPIYLTAGNFVPLTLATFVAVVKGSYSLFTVIKGSN
>McarOr11
MYFNSLLGVWPFVFERPDFRLWQMMYKMYSNLMLVFGTYVICTQYTQLVMLLQEEEI
W
VQEIIRNLCLTLLHSMGLAKVYAIRSDNLKELISEALKVEEDIYRRGDEDIMEIYRL
YAWHS
RVSNIAFLINIAIETCFYAMHPLYVGELPHFDKATNQTKMIRALPMSAWVPFDIQEQ
YLEAY
LWQSVEGTVTASFVMYTDIFSFSLIIFPLGQISILSHVLRNFDHYVKKAQEKHGCDR
DEASF
FIARECVVKHQDIIRYICVFNNAMKYIMVFDFLQSSMQLATIVIQLFGSELKMVEVI
FHGEF
AFCMLMRLMVYYWYANEIMLKSSDITLAIWEGVWYEESQRVKHMMLMIIRRSNKPLA
L
DIGPFSTMTLQALLGILKATYSYMTIMYNR
>McarOr12
MRANALLGVWPFIFEDNPKLQKIYDVYSRCTFIYYLLFIITAIIKLIFLICDEVFVI
QEVIANL
CITLLYSVTIMRVWAIKTPRVKNIIREIIITEERILKSKDETVITIYNSHAMQSKVS
NIIFLVNIF
LVTALYFIHPLYVEDRAKFYESKNITVIEKPLPLSSWFPFNEQEHYLVTYLWHVLDG
SIGAS
FVTYTDIFTFSLIIFPLGQLKILIHIMSNFEKYVDKIQNQLDCSPEEASFTTLRECV
LKHNEII
KYINDFNTAMRNIMVLDFLQSSIQLASIVLQLLVAEFTILNFAYSGQFALSMFIRLL
VYYWY
ANEIMVHSSDVAFALCTSNWYEQPEKVKKMLVVILMRCNKFLCLEIGPFTTMTLGTF
LGIL
KATYSYMMVIYK
>McarOr13PAR
NTVTSFKEDFFHANRVIYRICSLWLPGKEIPLQLRVMYLTYVFAWYFLFLTFLICEF
LIFKDM
LQEVSKFVNYFGMLFTHLVGTLKLSVIILQYKRINNLMSILQDPEYCYESLGDFQPD
VLLH
KSKIISFIVSVSTFVLYSFVGISAHISSHIVMNQVVKNPTLEKNMSCVDFVPYYFYT
PFTGTT
KLQCESMFLLMDICYFIHATIIACHDGVFAGLLNCLRTKLVILGGAFKTIRPRCLKR
LNMPT
NFTVLHEEENPEIEKVLYAELNHCIKNLHILLQSRDDIEHCFSYVTLAQSLASLFIL
ASCLYN
SSTVPVTSPDFFSQLEYFVCILTQLSLICWFGNEITLASNHIILSLYEGDWFSASPR

FKRSMIL
TMCRMQRPLYLSIGKFSPLTLATLVAVCRGSFSYFAVLQSI
>McarOr14
MDGGILRVQKLFMILSNKWEVNTKSVLINKLCRIQGIFFESYFILFTLYLPFNLLLH
RKCML
IFYELGGYFLHHTNIIIMNILFRKNAMKKTLKYIKNYEQVEYSKETQDSKDIYGYYS
LLNA
RLGKYVIVFGTCIAGISWYVSTISYSIKENTEDCAVLEGVMYQVWYPFKTRYNWLSI
IFDL
SMAYIAVSMHIFNRMSPITLVLFQLAHIKILANKIRNIDTHAEELASLHDVNIEQAL
NIAVDECVKSHQEVMSLMDLLLQATKEMMLIGFFSSSMELASFIIQLFTAASKYHFI
RCFIIFPIDLLQ
MLAFFWFADEIYVESTTLSNVIYNEVDWTRYTKPLRMKLIVMMIVAQKPIYFNATGI
GEM
TLEKFKCILNSCFSAVTFFQTMYYN
>McarOr15
MEKLVHIKLLRRMMIICGQWNFKNYNNPALTIYRAYSRFIIYHVIFMTQMILLTIAM
QWDC
RSRVIEMLMLLYSIHQHLVMIFLTKIYNLEKSLNYMMDYERVKLKQAGEDEKNVYFK
YAR
VNNNMNVLIIIVCILTAIMWYMTSIRNTFTVRGNEVCPISKGLVYQIWYPFNFDNQY
WLIVI
NDLIFFLNVVILLTYTKIISITVTIFMLGQIKILQEKIRNLEQDALVLQRINRTEYD
ESLLLSLK
LCIKRHQEIGWFMEVLQDSTSSIILTQYFSNTFEMAAFLIQMLTEKSLYLIIRSFIV
FCMVILQ
VYIFYWFANEVQIESTAIPDIIYSETKWTENDQIRRYLLLMMTRSQKKLSFKSAAIG
DMSLA
TFTKLIKLCYSIVAFFRTAYDL
>McarOr16
MSGQTQNIFLHAQRYIMIFVGKWYIDFGSNTKNRIYYIYSVLVELYFLFMTQQILVS
MVIYR
GCTERVGELICYYIQYTNSNICSFLSKRSKIRKIFNYIMDNEAEHLKHGTADVYLKY
AKVN
RKVIVLFLVLTGVAGAIWYILVVRDTFFAEENENCLILRGLNFQIWYPFDLFNRCYV
ITLLN
DILMYTSAVGVHIYNKISPVSFMIYILGQIKILQEMLRCIEKDAISMHELQGEKYEE
AILKNI
NNCVKMHQEVIKFMGLIDKGCKEIVLIGFFTNSLELAAFVIKVLMEEDVFGALRTIG
ILCM
TVTQLFMFFWFANEIKVESTYISDVIYYQTNWISYDKHARRHLWLMMIRSQRPLTIS
AAAI
GDMSIDTFKRIIKLCYSIATFFKTVYM
>McarOr17

MVKPIETVCCRTTLKILRTCYMYPEEGKEQNPGKLFLLKWLTLMILSSVTFIGSFLH
LIISLK
DEDYRHLDVDFSITLSMIATYIFTCFFFARVKFASKFYMHLSNLERLEKPLDFEKKN
ERLEK
FALYHYIYMELLVASLLLFSNVIKGAKCKQENLEFDLHEVCGLFTYTWMPFDIDYFP
VKQI
YLFLQLFGTHYLYLIAGTMAWTVVEAIQQIVLRLRHAKYLFTEAIKEVDPVLQRQKF
NRA
VRYHDAVLGLDDRLNGTFGVFMFTHLGMTAPILGTAFFAILHGGSGSSLFICLGWFI
GVSM
DCFSGQHLQNESIDIARALYDTQWYNCSQDIKRDVLFVLMRCTKPMYLKATSFGIMD
RV
MLLGVLKATYSYIALLTQTQ
>McarOr18
MDEIKEEEPFTHSLKMLNVMDAFPLEHNFFSNGIFFVRFWILRTMSFCISCVLPTVH
MVTS
VKDGIKLIISEDLSVIVGTMVSLITTCIFVFKRNSWSKLLSDIADLKIYGSFSDFDI
VKVKLN
LFSRIYFWYCVNATFVYGSVSFIDTSQCEEINKLGKEWREVCGTYLPMRLPFDANIQ
WMR
VSIFFTQMFFTLLSLAPSALACSIIFQSTGFIIAHIENLKKHLVGAFDSTDVQETSN
ILRYCISY
HNHILRLSARLQDLVGTNISHVLLMSAVVFAGIGNQILKTKPVGGTLYFIGYMIALF
LLCHS
GQRLIDETASIGSAAYNSKWYKGNTSMIRDTLLIIYRSQKPCTLEVLSLGSLNYPLF
LLIIKT
SYSYLTLLQQTA
>McarOr19
MFKIQKGAPFYSTLLALSLFGQIPVEFKETYSKSFLVKCILSRFIGFVLVSVAPILQ
YVMATK
GSIEVDISENISITISSIGALLTGTFLTCQYKKWLKFFEDITDHKAFGKPPDYEDLV
KNFNRFS
AFYTIYCTGSVPVYAVTVYFNSMRCDEAALKAGFFCKSFTPIWLPIDGSSLQLRVII
YIVQM
LLGGTIVCTSAVINFMVWESTEMLISHINSLKIHFNKISEKSTDKERSEQLGFCVRY
HNHILR
LSSRLNGLIKWTSGHMSLTAALIFASIGNQISNSKSVGAFLYLIGYVGALFFICHAG
QRIKDE
LMSVGDAVYNADWYATDVKTIRSLRFIIARCQIPFHYEAIPLGVVDYPLFLMIIKTS
YSYVT
LLSQTT
>McarOr20
MIKIRDIVCCTVSIKILQVCFLFPLKGKELEPNYLRGFIFFFLMGFSSLTVIGSFLH
FIISIKNH

VYYHIDLDMAIMISMFTTYSFSIVFFFNIKSAVRLYMTLSDFDEHGKPRNFDKRTKL
IDKVV
TYYYIYIEFLIIFMLSTSNVSSSGKCKKKNKKYGLNEVCGLFSYTWMPFEIDYYPVK
QIYTI
CQLVGTHYLIILAGVVSCLMAETMEQIITRIHHARYLFLEAIKEKDYAKQRQMFNTA
VRYH
IGVLDLEDPLNETYGFFMLTHLAMTAPIIGTALYSILYGGSGSSTFICLGWFIGVMK
DCCCG
QRLQSQSNTVPIAIYDSEWYTCNEEIKKDILFVLMRCRRPMYPKAISFGVLDHVMFL
GVV
KAAYSYIALLSQTT
>McarOr21
MSTDRICVPFSTSIAILNFNLLFTHGKSKSMLIEVMGWSLRLLSFINLCIFPIHHLI
TSIRDGIE
VDISEDISSSAGGFIAVILLCLFKFKEREWSKLINDIVHFSKIQKKSEFEKLTKKMN
KISAITY
SCFTIALLIYAIVVYNSTGHCRQMNKDMGLHEFCGSFTSIRLPFEGNSLIIRLPIFL
TQMFVN
ANALMSAAHITFFVYEITQYLVMHVDILKQNLLQVYDVGSSEEISQKFKSCIMYHNR
ILRL
MIRLNQVTKFTVGELSLTAAIIFACIGNQILKGSSMAGIAYFQGYVTELLFLCHSGQ
IIMDQT
ESIGASLYQSKWYEVEAKLMRNVIPVLRRCQKPMTLQALPQGNFSYALFLMMMNSAY
TY
FTLLAQTT
>McarOr22
MATRYPADYFGMIKIMYGIAAIWLFNPNGSLIVKCLKYSWTLILYSIVVTFVIFEYI
MIEVM
FKDIFTLIAQMGLLLCGHISLLKATVLIKNHKKLAEITDFLEDERYHYKSVGNFDPG
KLVV
DEKRFTNNFLKMLLVCFGLVGVSPHLAAEKIIHQEVKGNYFSENVTCYDYLPFIFYI
PFPSE
TKHMCEVAVLFMDMSISGIAIIIACHDGFLVVLLNCVRVQFVIVGEAFSTLRERVLH
RLNLP
EDFEIFYDEQHPQLEAELYKELNVVTRHYALLLKISEDLEEIFNKIILVQTLLCLLV
FATCIYA
GTTVPITSPTFGASVQCFSCVLAELALFCWFGNGVTTSSEGILLALYKSDWFSASKR
FKSS
MILTMTRVQKPVYITLGKFGPLTLVSLVSVCKASFSYYTLLKKMNI
>McarOr28
MNEGFLKIQRICMILLGKWQFDQKNLMQNKLYKIYGWFIIIYYFAVIQSMPFVIKSR
WACE
DIITKLGTIYLNHMNVFMTNLMSNTDHLKKLVSYILNYEKITYPKETDFVRKTYDFY
CAM

NYKMTALYIVTPSSLAIVYYAFEVTFTFLKDENDPCAPNKGRIYQFWLPFDTNKYFY
VAMP
FELLEITIVAILNTYNKLIPSSMSTFQLGQIKILQEMLRHVDDEAIKLHTNQHIEWE
QAVDIT
VTKCAKKLLEILSLMDILHKATGPLMLLVFSSNALETAIFIIRMLRAKTIVESLISV
GVSFIIF
VQLLSFFWQANEVYLESQNIIHVIYNETNWVDYNVSVRRKLIMMMTKCQKPLSFQGS
GI
GSMTIETYKKLVKSCYSVVTFFTSSYG
>McarOr29
MGRKFLKIHRFYMLILGKWKITPRNTLQNKLYKIYGWFIHIFFIGILQTMPYINRTK
INCWD
VITKRGALYMICNNMFIISLISNSVNMQKLVEFILNYEKLLYPSEKNFVQKTYDFYC
KMNY
NIVMIFIVIPSFFAYVYYGFEVILTFLKDPNDPCFTTKGLIFQFWLPFDTDKYFYIA
ILFEFFL
MSLAICFNTYNKLIPCSMSTFQLGQIKMLQEMLRHVDEEARELNASQCVEMDEAVDA
FV
TECIKKLQDILSLMNLLHKATRPVMLLAFFTNILETAFFMIRMLTAKSDAEAITALG
VSSVIF
IQILCFFWQANEVQLESQNILDVIYNETNWVDYNISVRKKLLIMMTMVQKPMSFEAL
GIG
SMTIETFKKILKSCYSIVTFFKTAYN
>McarOr30
MKLHKLLRLQNHLLILLGRFEESFGNIFVDKLYYIYSWFISCYYILMLQSIPFIVLT
KWECTE
LAMKMASIFLHHSNSFFLSRLAIKPPMKKTLSHILNYENLIYPNEAKDRQNKYNHFA
NLNF
IVSLLAVVLPTQYGWLYYYLEVKRSYIEQENPNCALKKGLAYQLWYPFNVEKYLYIA
RIF
DFLELLMATIYHSFNKSLPIGMACYQLAQIEILHLMLRNLDTDAKKLQHGNIGRDEA
VETLLNECIRRHQIIIDFMGLANKAMRTIMFIVFFTSSMEMAVFLIQMITAQTTTDR
LLTCGVSCV
LALQILSFFWFANEVFLQSMKVSEIIYNEMNWVDYTIGQQKKLVIMMAQSQKSLSFR
ATM
VGAMTLETYKAMIKSCYTLVTYFKTVYE
>McarOr31
MGCRFLKVHRIYMLILGRWKVSSRNTLQNKLYQIYSWFVPIFFIGIIQSMPYIIRTQ
MNCSYI
VTKRASLYMHSTNILIISLLSNNANMQKLLKYILDYEKLIYPSEKKFVQKTYDFYCK
MNY
NIVTVFILIPSIFPYIYFTLEVTLTFLKDPNDPCATTRGLMYEFWLPFDTEKYFYIA
MLVEFY
ELFLAVCFNTYNKLIPCGMSTFQLAQIKMLQEMLRHVDEEARELNASQCVDMDEAVD

RF
VTECAKKLQDILSLMDRLHKATKPVMLLAIFTNSLETAFFIIQMLTAKTTTEAIIAV
GLSCVI
FSQTFGFFWQANEVHLESQNISDVIYNETNWVDYNVRVRKKLLIMMTMVQRPMSFEA
LG
IGSMTIETFKKILKSCYSIVTFFKTAYN
>McarOr32
MNDFLKIQRICMLLLGTWKLDPKNVLRNKLIKIYSWFVIIYYIGIIQSMPFVLKSKW
TCIDF
IKKVGTLYLNHTNVFITSLMSNTDNLKILVAYIMNYEKLIYPTERYFIQNTYDFYCK
INFYIT
AICIVAPSSFWCCLLCYRSYLHIFGGMKNDPCASMKGRIYEFWLPFDTKTYFYMTMP
FELF
EILIAVVLNTNNKLIPSSMSTFQLGQIKILHEMLRQVDDEARELHESQHVEFDEAVD
LLVTK
CVKKIKNEILSLMDIMHKATRPLMLLVFSSNSVETALFIIRMLTAETVGEGLVSVTV
SFMIFT
QLLTFFWQANEVYLESLNIINVIYNETNWVDYNVSVRRKLIMMMTQCQKPLSFEGLG
IGI
MTIETYKKLLKTCYSVVTFFETAYD
>McarOr33PAR
MDPVDKMSDYFKHNMISFKYTGIWLNLFDVRRTSLLVIFYSVIINSLFMMSPQVCHV
IYM
YKARNNVQAFADEFYVSLASLLVVLKSYSLLKNFDIIKKCWKPWTLIFFKQKRLSAK
ANT
AGYGNMEMIYWLYATFALLYVFLLLISVLLERILQGTKVLPLVVCYPFEVNVSPVYE
LMFL
YQAIALSWLVIQNFNLDTFITGLLTVAAVQCDLLCNDLENLTPEKLECQKGEENDIM
DEKL
VDCIKHYQEIRRFVNDISHCFSMNIFQQFSCSVITICTTLFEFSTKEPLSQEYFAII
IYQSSIFIQ
LFIFCWTGSELTEKSKRIPISAYASKWEDASKTFKSNLLIFLHNVQRPLEI
>McarOr34PAR
IVNMIMVLGDIEKMTEASFLALTHLVQVMKLFYVLRYENKLKLLINSINRKSFQPKN
LEQY
VILQKYVRESNIISKTFLSAGFVTCCFWGVSPLTQSGDIVLPLAGWYPFDTDRSPAF
EIIFAY
QFVASVTNALSNISLDTLMSGLIMVVCAQLNILNDSLRNIRKYAEAESDDGRAVSRE
ELQR
RMDERLVECVVHHKHILEFSNEVTFLFTNSILGQFIVSVVIICITLFEITLLPWGSL
KFFSLILY
QFCMLLEIFLLCYYGNEVILQSMQLTKFAYFSDWTDCSTKFKRNLLFFMTRSQVPLR
IYAG
GFFTLSLETFVKILKSSWSYF

>McarOr40PAR
IHTVTAGEYNHIDLDLSYVLSMTSGFLLFTLFSFNITTATKMYMFLSEFKEFGKPPK
FDKYN
AFLNKVAKFHVIYLNINITLFAAGSNVFKGAQCKKDNIELGYKEICGLVTNTYLPFD
IDYFP
LKQIYVGLQFFSIYYVYTISGTITFMVMETMMHIGFRLDHVKQLFDEAISEKNVERS
RKRF
NFAARYHARVLELEHEVNACFSYAMFSHMILTAAIIGCAAFGVMQSGSANPFAVCIG
WFN
GISFVCLSDQHLINKSLEVGTAVYSSKWHQAHPSLQRDLVIVIMRCQKAMILRSAGF
GVM
NRATILAAVQASYSYITLLSRSP
>McarOr48
MSKRIMLLLGLWPATSSHDWKYICYKAYFCAVRFNYGVFIITLIMGIFSKYTLNAGP
LEKA
DIIQNYLLYILLLWKMILITTSQILKLIECISEREMHIMNLEEEDIKNIYAKNVNYN
FKIFLIVT
ATIVVGLTQFCILVSFEIHGLKNNIERKLPLPEWFPFNVGKHFLTSYYFQVFNRIYS
GMIIVAMDSMYFSLIYFPITRLKILGHNLMFFQELCNKNYSETPDNILKALILEHQD
IMRYVKDFNR
LMKWYLFMDFLVRSYHISLVLFKIMALQEFYGNGSDGVLLLTFSINYLMVLLLQMHI
FYY
HSNELRLESMEISSYIFQGNWYDQSPSCKKSFLIMMMRAQKPLEIHIGNLNTITSYL
IVKVL
KAGYTYVILSRK
>McarOr52
MRYEFLKIQRICMLILGKWELNSKNELINKVYKIYGWFVPIYFIGIIQSIPFILRTK
LDCTDIIT
KIASLYMISTNVFVISIVSNSANMRKLVSYILHYEKFIYPTEGFLYKKTYDFYSTMN
FNIITL
FIVCPSSFGIVYYGYETSVTFWRDENDPCAPKKGLLFQFWLPFDTEKYFYIAMMFEL
FEVC
LLVIINAYNKLIPNSMSTFQLGQIKILQEMIKHVDEEARELHVSEYFEMDKAVDILI
TKCAK
KLQEIISLMQILHQATRPVMFLAFFTNILETATFIIRMMTAKSAGEGIIAVGVSCII
FIQIVSFY
WQANEVPLETQNVINVIYNEINWVDYNVRSRKKLILMMTMCQKPLYFEALGIGRLTI
ETF
KKIVKSCYSIVTFFKAAYN
>McarOr54
MVLFPKNDHFKVTMYANALLGVWPFIFEHNPFMRKLYHIYANFTFIYFMLFIVTAYM
ELV
VLLMAKELRVQEIVGNLCITLLYSITIARVYAIKSDSVKNLIREVIEVEEVIYKSDD
EEVIGIY

KEYTHHSHISNIIFLVNITIETIFYFTHPLYVGETIVIDEATNATKVVRALPLSSWF
PFDEQEY
YHLTYGWQMADGTVGASYVMYTDIFTFSLIIFPLGQIRILMNILRNFDKYVKMTQDQ
YGY
ERDEASFLTARECILKHKNIIRYINEYNRVMRNIMVFDFLQSSLQLASIVIQLFVSE
VRLFNV
IFHGEFALCMLIRLLVYYWYANEIMVQSSNVALAIWDGGWYEEPQKVKHMMMMMIMR
S
NKPLVLDIGPFSPMTLSALLGIMKATYSYMMIIYN
>McarOr56PAR
SPHVTTVMSLQPRSKGIFKNLTCYDFMPYTFLYPLFPSQTKRRCEIVALYMDIAISG
VALGI
ACYDTFFAAILNCLTAQLTVVNKAFRTIRQRVLQKLKVASNIKLFYDDENPRLEKEL
YAELT
FTTRHFVALLQIAEDLGKIYNLVILGQTVECLLIFAACVYIATTVPITSPEFAAASQ
YFAAVF
VQLTLFCWFGNGVTTASEDILLALYECEWYSASRRFKSSLLITMIRMQRPVYLTLGK
FGNL
TLVSLVSVCQGSFSYYALFKKY
>OcomOR1(ORco)
MMKFKVTGLVADLLPNIRLIQASGHFMFNYHADNSGSLHTLRVAYSCMHLIFCLFQY
GCTFGNLVKEKDDVNYLAANTITVLFFSHCITKFVYFALRSKLFYRTLGIWNQPNSH
PLFLESNNRYHALTLKKMRTLIVCVVAATVLSAAAWTGITFVGESVHNIKDPDNEND
TIVEEIPRLLIKSWYPWNAMSGMTYYITLVFQIYYVLFSLMHANLLDSLFCSWLIFA
CEQLQHLKEIMKPLMELSASLDTYVPKSADLFRAPSANSQDNLIENDYNAKNDELNL
KGIYNTRQEMGINFRSGALQTFGQGGGGVGPNGLTKKQELMVRSAIKYWVERHKHVV
RLVTAIGDAYGVALLLHMLTSTVMLTLLAYQATKINGVNPYAASVIGYLVYALAQVF
HFCIFGNRLIEESSSVMEAAYSCHWYDGSEEAKTFVQIVCQQCQKAMSISGAKFFTI
SLDLFASVLGAVVTYFMVLVQLK
>OcomOR2
METENVNLKRLYRYPDNFFYTNTVILKIIGMWIPSKDYSIFFRLFYLLYVLFLYGEG
LLFLFYELLFFGETDQISNFITFIGMVFTHMVGILKFSILMLGRHKIRKLMNTLQDV
KYFYGPLDGVFPGEILSKGKATSSKISKLVFIMYTFVGISAHISSDIILNNEIKGQY
FDEFNKTCIEYMPYYFKIPFDVSMKKQCEIALTFMDLGLIFGAFIIGCYDGVFVALL
NCLRCQLIIVCHVFRTLRQRSLKSLDLPKTYTVFVDTQNPALEAEMYKQLSHSTEHL
KILLRVGEEIEAMFTFVTLSQTLASLLIFASCLYMASSLPMTSPEFFSQMEYFSCVL
LQLMLFCYFGNEITLASQEIRSALFDCDWLSSSSRFKNSIILTMIRSQRPLYLTIGK
FSPLTLSTIVAVFFRFAEDLFLILRYSKAFNNTKNSHQGLKIIRINN
>OcomOR3
MSLFSNISWKLEETTRCKIYVRYFNWKQIFIMKTFSGLKPHRLPNYFAVYDKIFNTS
GIWIEGKCIGRSLMFTTIYVASVIFLFIVTPQFCHFIYMYKARDNVVAFADEFYVSL
SSIMVVCKDYCLIKHRKNIKLLMESIDADIFRPANKEQYQRIFKVIMDKHKKIFWII
TFLNVSFCICIAVSPIFDLLKNGTKLLPLVDCYPFSVTQSPVYEIMYVYQIFVCFYL
MIHNFPFDIFILDLIGFCTAECEVLCENIKNLKIEKSPVVYGKKLTKCIKHHEEICR

FIRNIENVFSFLILQQYFCNLISSCTTMFKLSITEPMSLDFFKTLSYQCCLLLQMFL
YCWTASSLTEKSQKIPKLVYDSYRLDVEKSTHTNIKLFIAKSQRPLLLRTLLFSLSM
EQYLQLIRNSFSYYTVLRSLNENT
>OcomOR4
MEDPYVQDFFFLNRWILIVNGLWKPESKNKIIQLLYKALVIASFLFVNIFFTITEIL
SLSDVYDDKYDLIQTVSVIMTHLMGMLKVALLNYQGDELKRIINTLESSELHYEHCS
AKHFFPGKTSRVYKKIGLKYTLLFFITVNMTVILSYFPSFIIALKSEVNNPEKMLPD
KLPYFSWMPFKYDTVRGYIWALVYQAIPMFTTGYSLIAVDTLFLNIMNCIAMNLEIV
QGALLSIHPRAANKIKGPLLTSDQLHNTKALSTQMKAEMKKINKHLQIIYKASDDLE
NIYKYITLAQATASLFIISTCLYLVSTAPIGSKQFYSTIFYLVDMGLQPALFCWFGN
EVTTKADSLSLYVWQCDWLTADKDFKTSMILIMARANRPLYFTAGKFAPLTLSTFIS
LVKVSYSFFTVIKSGE
>OcomOR5
MSFLDRTARNIAGTDDYGKKFLWIPQFLLQALYMWPEQKDILKRKFMYILALCFLSF
YRFGVGHKLTKSEDFNAIFDQSFYLSTIFHAVTKAIVLYIYKDQFKYILYDIHHKFW
PHDLLGNKLAGDIKSIYKWITYVMIFHITTGVIYLINTFVRSWINRRYPFLISYPID
FVDKPLVFPILFIGEMFSLAYQMPLVVLGCDHLFFTLTFSIIVQYKILHNGVLLFNM
PKMSAVNARLRLIGNERLEVIYRDENREFLVRCVRHHQLLLKMIKKINSLFAWIAIP
QVTSTICTVCGVVFKIANEDISAPAQKVLAVIMIMNILTELALFCVMGNELYYQASL
LPEYIFHANWHGLSSNEFKKDFIFLLQRSQDIPNLNAYHIYVFNLDFFIRMLKLTFS
LYTFATTIKPQ
>OcomOR6
MTFQVGFKNFKDYSKRGLWLPEFMLKVIFMWPKQNAENRNLFYACSMFSVVFVLWGG
VSYLYRYFKDYNQTINSISVLSPFFQTLFKLSILQYHSVSLGKIIEDITVKFWPCDL
FPDHAGASKRRYRRELSLMLVLLSTGFSFGIGLCLFPLLSTDRELPYRSIYPFDCLK
SPIYEFLYVLQCLVCVYLINNSVLSFDFMFINICNNLVTQYILLKKCFTVYGDDEKV
KLLNKKFKKFLNLPRCAYETKDENKLFLVRLIQHHQLLTRTTKNTENVVNIIAALQL
CMSIVAICLSGFMSTNADIGRMELLSMGIFLTGHVIQLILYCSAGNELFYESSNLTD
NIFGSKWYKIEDTGLNKDIIIVMKNARNPAKITAGKVFPLDYSTCFEVLRLTFSFYT
LLSTLINK
>OcomOR7
MNQSYFNFKEIVNISLIFLYFFGIMYPKFDSPIITIKYIIRVAVFLGFFFGGIVVAE
ITNLYLSFGDLQGMMKALFLTLTNLVSIGKFYVICKNQKNLLSLVDKINCKEFQPKS
EKQSRALKKYIRLSKIISFSLYTGCTLTCGFWSIYPYTEENGPFLPIAAYIPFNTTN
GKIFGIVYAYEIVATVIGGYTDLSADALMASLIMVVCAQLHILNDSVINLYPVKEKE
LQPNKSKSNSLPNKKDIIVNQKLIECIKHHRAIMDFAENISTLFTTSMFWQFVVSGF
VFCATFFEMTLVPVVSVRFFSMALYQFCMLLEIFPICYFGNEVILESNRLTNSAYHC
DWINYSVEVRKNLIFFMTRSQRLLKLYAGGFFTMSLDTFIKILKTSWSFVAVLIQVQ
NQTQRDK
>OcomOR8
MEMENIDYMTFFAFNIRVFKICGMWKPDSNSNHQIFSTTYNLVCKAFWVLFLLSQFL
FLYKSPNIFDELLSNSHITIPEFVCSLMFIGIYRKLDLIISIVRKLNEPMFQPKCEE
HFQLAKSLTKFYNTYNYSVTYLAIQTSLLFLILPLWQEKQTLPANAWFPFPWEPSPN
FEIIWVFQSVSVFIITVNVACLTIFSSGLFMLIGLQCDYLSVTFNNFSQFHVKNKIL

LKKHNTSIQSTGQIFSNIMMENLVVCIRHYSEIRKIAAEVERIHKTAVFILFLGGAT
MISTILFSLSTVELGSPQSLMLISDTMCMLVMQFVFCWFGSEITEKSGNVFITAYNT
PWTDCNLRFRKVLLQFMTMTQNPIEMKAGGLLVMSNAVFTSIVKSSYSVYALLHDIE
E
>OcomOR9
MSFLDRIARKIVGTDDYGKKFLWIPQFLLQLMYMWPEQRDTFKRKFSYILALSFFSF
YGFGMLHKFTKISIENFDDVFDQTFHCSVVFQGVTKTIVLYIYETQLKFIVDGVHQK
FWPYDLLGRKLAGEIKTIYRGVTFLMIFHLIGTILYLMNLFVKCWISRRYPYLISYP
MDIADNPIVFPILFLGQIFSITFQMGFVIIGCDHIFLTLTSCIIIQYKILHNSFLVF
NTPEMSAVNAKLRLIGNDRFEEIYRDENKEFFVRCVRHHQLLLEMINKINSLFGWIA
VPQVTSTICILCGVVFKFTNEDNPATSQEIIGVAMISVTLSELSLYCIIGNELQYQA
SLLPEYIFQANWHSLNSNEFKKDFTFVLQRSQDIPNLNAYHIYDFELNFFYKGSQTN
>OcomOR10
MNMELEVDLREALTPNIKVLTFLGVLPKLTNGKILHFLHVVITGLIYMIVVIAQLVN
LVLSFKEDIEEIVTATFLTAANALTIIKAFFVFKHRKQIMDLINTLNKEEFKPRNRQ
QCNVLHEYIRKARIIPKILIGSIAISILFFSIYPFTVEGGPFIPSPAYFPYNIDISP
QFEMTYVLEVSGLSISACCSLSIDSLITGLFTVICAQLTLLNDSLENLDKNTENKNL
NEEKSATFNDKMNVNLIKCVKKHRSILQITNDVQSIFSVAAFGQFVVSVLILCTTLF
KLSMVGFDLSFGYIIIYQGCMLLEIFLPCYLANEVTNKHQQIINSVYNCNWTEADRT
FKKKLLYFMTISQKDLIIYAGGLVPLCNDTFLKILKSSVSYYTVLNQMNNK
>OcomOR11
MYYSIQKKQAYYSTLLTLSMLGIYPTSGTQTAFKTSYFVTYGIIILSLVICNIGALG
HLFVSLKGSRGSEISEDMTIAIGGLGFLMSVALFKLRSKKWIHVWQNITNFNDFGKP
DDFLRIAARSNFLSLLYSLYIGLGTMFYTFLSYIETDCTNEDEANNFCGTLTQIWLP
IGKVSRVTIYIIFFLQMLICIWTCIGAGNLFFVSYEAAEFLVCHLKHLKKRILNIFK
HSDEKTRKIELACCVKYHNYIINMGNDLNGLIKFTLGHMSLTAAIVMG
MLAVQIVQKYKPLGAVAYLGGYVVAIFWLSLAGQKLTDESLSIADIAFESDWRKGNI
EMKRDFIFIIRRSQDPLTINALPLGSFNYALFVTILKAAYSYFTLLQQSVFEIE
>OcomOR12
MDTKHIKKIKILEHTIKVLQIPYLFPGEKELNNPKANLYIKYILVNLSTIYYSVGAL
LHMIFNLRKGIADHIDKDIGNVISYHGASYFVFRYLFNLKKIILLFKQLSDFKTFGK
PNNWDDVNKRINKWSKIFVIYQSAVLIIIFSAPFLYVSQCKEENLIKNREEVCGLAI
PTWLPFKFDYFPVKEIVYMYQLYSLFVVYGTAAIISFSMLASCELFAVRFEHVKDQF
VESFEEKVVTIRKEKFNKAVRCHQTVIKMGTLFNECFSPCMMVHISLTGPFLGVAGY
TSLTKISLDSIAFVVGWLISTFIVCHGGQRLMEASISVGDIMNRICWYDVEPSLQRD
LILIIIRSRRPIFLTAGPFGPINYGTIVTILKTSYSYVTLLKQTM
>OcomOR13
MTERSMRPKKSIINFDSFFKPSYSVLKYTGNFLLTENPSEMNYIIYCIFINFTTSTY
NLFQILQYLVKFDFKNFLDMLCITPNAIMGNIFSCLIFKNRREIFILTSQLKEERFQ
PNNPNQLIIARRALIKLNRKVYFFLVVFFVDTLFGILRAIANKTLLFCKWYPFSLEK
SSLFVLAFIHQNLAVFCVGFTGIAYLCVLGGIQLFLGVQCDFLACKIKTIGQKEEPY
PEKSLRDCISYHKTILKLASEAQNIWNSAYLSQFCGAAISSCVTLVIIATNKAYNKD
ILYLMGYQSINFLILYYYSWFSTELTSKSENITNAIYSCGWVGKSKTFQKDILYFLH
MSQSSTIIFTIYGYVTISLETFLSVIKTSYSTFTILQNLLNEG

>OcomOR14
MSKQKILMFNYLDWYKQDISFLRLAALYCVDENGKIVKNQLIYTTFVNVFLILLGNI
LQTVNLFVKKQDVISIASSAYVVAITWLCTINFYYMFKHRKLLTRLFADVNLDIFQP
TDEKGVKIVKGSYQLYKTVKNGLLMICTGAVLTSVVTPILRRNESVEVRFSMKLWYP
FDIAASPVYEIIYLYNCLLNIYSTIMNIYLQIMVGAFTTFIGAQCDLLCHNMNVATN
LNSINKFIYHHQCIIDFKERTESMISSLYLFIFAASNISVCMTLFLLKMVDRSSFEF
IFLMMYSFGITFQLFLPCWFSSEMNRKSERIPYFVYSLPWHDASIQLRKKVYIFIVT
TQEYIILEVMNLFPLSVDTFVTLMKSSFSYYTVLIGINGEEN
>OcomOR15
MVKSVETMHPNDLHLYKKEFRNILRLWNFVGMNPLRGYFKPLITFNTILTLYLTVLI
LLKLFLGKELVTVESVGVFSQVWVKLFVLTIKRDKIVQLFIDMEGFWKTDPEGSENS
KLLKDLRRKERMFLIYISCSTCLFLFKPLLVKGTTIYYYYRIEQVPFVISYCIEFYV
TLATMSMVIGVNLFISTTIVIGAGQFSNLNAKVRQLDLSKAQYDKNEFESRRREIKE
NIEYHDYLIMYVRNLDGIFSWLFTLLISIITALLCMNMYVLSQPNNTVVDMTRCGTM
VLAFTTEFLLLYGVPAQKLIDEAQEVANSAFYHCQWYLPGNVPLRKSLSLMILRSQK
MVCLSAMGFLDINRQTIISMIKTAYSFFTFLQTVEVPGPEK
>OcomOR16
MKIIDKTVPFSVTKYIMSCFLLYHENDSVLRLVLVVFVKIFALFICPVQSVIHLIAV
KKEKLDLSVTENISIIISITSMIIMSSILPFYSKEWKTLLSSIVNFESTAKNSIEIK
NKIIFLNIASIAILIYCNGGMMLYAMAAYYESRKCLKFKEENNLNLICITMFPVWLP
FQINILTEIIIFALQFIMIYLMSVAGFMGSYLIYEFSACTSLRIRCLKSKFFDIINN
TPPEEQDYKLKLWIQDHQSIIKIIEEAKRLTRICLGFGSLSFGLVCASIANQVINLN
KPVGAVLHLLGWIAALFMHCHAGQTIMTDTESIAETIYNSKWYEISVSTRKTILIIL
MRCQKPLTLDALPQGSLNHPLFMMIIKSSYSFLTFLSNTK
>OcomOR17
MFKPNFKKAFDREKFFLQSFGAYPLVDPRVYFSRFRYFIIYSINVINVALMWMLLVV
NDIKNFLDACHFIVISLTLFCKLAMFVLAKRIKFIEDYINSIDVTNIPIGIIRHVRR
EEEGRNWIYFPQRYLVLGTVLYLFLSCSLRQNDRSLVLISWSPFDLQDIKYYYLTIC
FQSVTFLTNGLTNGSIDSLYYILIDIACCQMDILMYNLINLDNSKGEVELCRNLKRH
IMNHMLIVRFVKNVQNIYSTMIFIQCIGSVLVICILGFQLSVTVSFPS
ERFFIQTIFFNSMIFQIFCYTWFGQKLMFKSEEVVKACYLSKWNEFELKNQKSILIL
MTRCTKPIILTAGIFDLTLKTFTKILKTSYSYMAALRIMFNEYVTKN
>OcomOR18
MKVNRGYLKAFNREKRILQMFGIYPLKDSNQKYYFLRFLVCYGVNSIEILLMLMLLV
VKDISNFLEAFHFIIITITLLCKISFVALASKDMKSIEDYLNMFETADVPRGIVKYV
AGEAYIRNRFYIPQCIIVMFAVTFQFSTCLIFHKLRLLLFINWSPYDLKQTAYYYPT
LIFQAIVLASNGMPNIAIDTMYYVLVDTACCELDVLVYKFSNLDPLKNPIKTIKELK
ENVICHQQIIRFITFIHKKIYSKLMFVQCIGSIMAICVLGFQFTTTVNWFLFIQHST
YFSSMIVQIFCYTWFGQKFKEKSQDVLQACYMSKWYECDIKIQKMLLNIMTRAKHPL
VLNAYIFDLTLQTFIGVLKSSYSYMALLRTMYNK
>OcomOR19
MADYYKDVKWCINYCNILGVNLLKKNNIIQNICCCYHFLSLLGIVLVTIILFLSDED
MSNIKYFTDSTNNMLIVPIGLSKLITLLTKKSAIADLINDTKYQFWQPPDVKEIRKS
EKFATFLKNTYLNCLLLCILSSILKPIIVGEAVSAYNHYRPEFVPRYVFVILGNSSH

VVCILSMIGFDVLNLVLLMRVEVQFRILNNQLNRLFDFDHQKDVYSQTKLSQRLKEC
VNHHNFLIEFLTKMKKIFSGVTLIIFGLTIMSMSLRMYILSGNNGFNYKLEAMFHLI
AGLTQIGVCYCIPSQAVRNQAEKIGQSIYCSRWYDYPLEAKNVLHILSKEAKTIIIT
AGGMFPINLEIFLRIIKTIVSYCTFLRTMEVVS
>OcomOR20
MIKLDFVKAFKHERYVLNIFGLYPGLSGSYRKFFTKFTYIIMAIIQLTLALIQLGIS
KDFTNLVDCAIFSIGMINFLIKAVVLHFKKDVLDSITDYLEKIDESKVGASIRQNLK
NHEIIRGQLGNMFRLATFLYVLLQVLIVFFQKGHTFLPVNTWTPFNIYLNIVYFGTV
TFQLLVIGIEITCDTSLDCLYYVLADVACCQLDILKYNLIKINPKGNKVKQKLIECI
QYHQEIIKFVNQIERVFSMILFLEFFKGIVDICLIGFELTMVEFLSLHFFMRIIYLN
GLLLEVCGFCWFGHALISKSAEVEDSCYLMRWDECNTSIQKLIWMIMVRSHKPLAVR
ALFLTFTMSTLTTILKTSYSYTTVLRTMYE
>OcomOR21
MVSKQVKFLLLIIQRMGFLKQQTKLEKFLITLSVIFLIISIVLIWMFLLFNEMNVES
ISIVVIGTSSFSEILTGAFSFIMHKEEFLELLNMQDMFWIYSDDNIARKKQKIDRLF
KIVINMEFGMVALTIFTQASATFSGTPSFICYIPGDNQILSIVIGCIQLFIILHVTF
PIVALDFIAAIVVTELYLQMEQLNITFSNMKNLADIKKCVRYYIFLNKCIRCFQKCM
DVMMFVAYFATMCVCCTQLNLLANYKINFSTAIKCVCFMLGMLFQFGLYCIPVSLLK
DAHENLSQSIANCDLILNGDKNLKVCLIIMMKMVQKELSIKAGGLVEMNRAAYITML
KSTYSFYTLMSQNSS
>OcomOR22
MSLIYPTEGQYRMSYEPLSTLKKCIQLHDFKGFKKYFWHTVTLLKVFILLGRTIYAY
QTLNQPKRLAEIVATYPVRFMSVTKIYMLYFDRKRVNYFYNTVTQEFWDFHVAGPEL
EKQVKKRFTIIHYAVTCQFMAAMVVIILFLIFPLVDMPEGKRPLPNIIWTPFDTNPS
PLYEIIYVIMLWNLILSLLGNAFYDVLFTYSLQHLFVQFMLLKKLMKNITKGIMEES
SDCDKFNSEYFQRTVYERVRICAEHHAKLLKFGRNIKIFSSRALLPQLIMSYAVLVI
NGYILSIDHKDVMKTTGLLNLTISCLIQLAVFSLQGSSIKFQSLSILDAITDSEWYL
FKAPVKR
>OcomOR23
MNMALEVDLREGISINIKILTFLGVLPKLDSNGMVLHFFHVSITDALNREEFKPKNS
QQCNILRRYIRQARMLPAILLGSIVTCAIFFAGYPFAAEDGPFLPSPAYVPYNIDVS
PQFEITFALEVTGLCISAFCSLSIDALISGLFTLMCGQLTLLNDSLKNLDKNTENPN
SNEIKSETVNEIMNINLIKCVNQHRSILQFVNDVQSIFSVAAFGQIFVSLLILCTTL
FELSLVGLDFSFGYIIMYQICMLFEIFLPCYWGNEVTNEHKKITNSAYNCKWIEADS
VFKKKLLYFMTISQKDLNIYAGGFVPLCNETFVTVLKSSVSYYTVLSQMNSKQE
>OcomOR24
MSFLESTARKITGTDDYGKKFLWIPKLYLQLMCMWPEQANNFKMKFISILTLCSFAF
YGFGFGYKFTEIPSESFEEMFQQAYYLSGVFHALTKMSFLYFFKNELKSILYDIQHK
FLPYDLVGEKLAVEIKSIYQGVTCLMMLHITSGTIYLTNLFIHSWIARRYPYLISYP
TGFEDNPVVFSILFIGEIYLIPIQMGTMIIGCDHLFVSITSSIIVQYKILHNLFFTF
NTPKMSAVNAKMRLISNDRFEEIYRDENKEFFVRCVRHHQLLLEMIKKMNNIFGWIA
IPQVVCIICILCGIIFKLNNEDSSSYSLKLEALLMTINMFSELGLYCVAGNE
>OcomOR25
MSLNFLNSFKPELMIVKSCGVWDYVYRTRYQFLYILYFIIVNVGLAMYNLMKFNDML

ESSTLETAVAAGFVLPIALMGNIRSLCFFTNRKEFFELLTSMDDDIFRPKNIEQTTM
AQKMLKYYNDFKLGMYVFSILPSFGCPIGRIIFGESGQKYCEAVITSSRGTGIYLFQ
AISLGMISVINVVTNYFMVGFSLFVCLQCDQLCHQLKHIDVTNGTIKDIVQHHRRIL
RFASNTEKLFSFIYFSFTIMCLLAFCTTLFMISIISENIPLAAYSCAWTEASKSFKN
DLMIFISNVQKPIQLKAWNLVDLSLETYMAVIKTSFSYYTVLNSLVFEEN
>OcomOR26
MSCLDRTARNIAGTDDYGKKFLWIPQFLLQLIYMWPEQKNIFYRKIMYILALCIYIF
YGFGIAHKLIKSQDLDEILDQTVSISVYFHATTKATVLYFYKDQFKAILYDLRHKFW
PHDLLGNKLTEEIKSIYKSVTFVMLFHAITACIFVINTSVKSWIHRRYPFTISYPIF
DVDNPLVYLILCLGQIFSMVFQLTVLILGCDHLFFTLTFSIIVQYKILQNVVLLFNM
PKMSAVNAKLRLIGNDRLEEIYRDENREFFVRCVRHHQLLLRVIKKINILFVWVALP
QVITTVCTVCGVVFKIANEDISAPAQKVLAVIMIMNILTEL
>OcomOR27
MERVKMLIKSINRKEFQPRSKKQKEILQTYINTSKLVSYSFWGACVATCVFWAIYPF
TEDKLRLPLAGWFPFSVANSPNFEYAFIYQFIAATLNGLSNISIDTMMSGLIMVVCA
QLSILNDSLTNIRHFAELEMGHKFRDDNGRNEISPRLQDTMNRKLAECVVHHRCILE
FAEEFEVLFANSILGQFTVSVIIICITMFQMTLTPIGSLQFFSMVLYQYCMLLEIFL
WCFYGNEVILKSRLLTESSYISSWMFCNKEYRRNILFIMLRTQKDTKIHAGKFFILS
LSTFVKIVKSSWSYFALLLNVNNTNGSKHL
>OcomOR28
MLKKEENLKKNHLVCMSVTLNIMRAFLILPSEDEMLKKPGVFFFIKFFIVSVVGSLP
LISSILHLMHTIISKENHSIETDLTYIVSLVTSYVAAWAFIIRIKHVSELYIFLSDF
ETFGKPKDFDLNNNNFNKYAKMHLIYLEIVVWLILLASNIVTVDSCRQQNITYNLTE
VCGLFTNTWLPYDIDYSPVREIYTVIELFGTSELAVAIYDSPWYDCDEELKKDVLFV
LHKCQTYQVLHAGAFGIMDRAMFLAVLKLTYSYITLLRKP
>OcomOR29
MADTLLFYTMFFNLTYTRVITTFFMVYVLGQIKIVQNMVRRIDSTSKVLRNNMDYNA
AVFQAVTQCVKQHYEVQRLMHLINKACREFVLISFFCNSMEMALFLIQLLMAETIWE
VFQQTAVLFMTVSQLFLFLWYANGIKVESGEIANIIYNETEWIKYDIKTQKVILMIM
TISQKPLTCNSIFIGDMTIDTFKNMIKLCYSITTFFKTAYL
>OcomOR30
MTEEITLCSNKCCKSAYHYLQIDVHYGTVNVQKTVIQVNYFVNWRSGDIKGIPTNNE
SSEFYYKRNFKFRIKSLTTNYFIIPKNIFKTISGCTIFIFNILCMLFASSNSYWLLS
PIKRGN

>PaenIR1
MRLKEILLFVILALQLSLQTNYEIIKMISDFVVRLQGPIRVNAFICWRRENKIQLWK
EFSLNNVACDMGNTHNNITHYFPPEHQLFLLDMNCNGSYDILEQGEQMNLFNQPFRW
IILGPISYENLKRYYFRTDSQIFFVEKEEKNGDYFYRIKALYKLSADSSEFIENDLA
EWNSKRGFQKYNELSFSRNRTDLKGINLNISYVVTNEDTYNHLEDFRNTHIDPLSKL
NWLIIKNLLVILNATSTPLFVTTWGYMDSNTSKFTGMIGDLQSDNAEIGGTALFFTI
DRIDVIEYIAPSAPTYMKFIFKAPPLSYVSNVFTLPFDTDVWYSCFALVPLIFVTIY
IIVKWEWKDPIFKEEIQETNSNSIVPLRPGLIDVLVMQLGAITQQGAESEPKSNAGR
IATIFAFIAFMFLYTSYSANIVAILQSTTESIKTLEDLLNSRISLGVEDIVYAHYYF

KTAKEPTRKAIYEQKIAPKGQKPNFMTMQEGIGRVQKGFFAFHVEVSSGYKMIGGTF
QESEKCSLKEIVFINLVEPWVSIKKRSAYKEIIKVG
>PaenIR2
MSAQNLVCQIGKFNENIPSYFPPEHLLFLLDANCNGSNILLQQANELKLFSHPYKWI
VLDQADQKTFEGIFLTVDSRVFIIKNIVENDIYRIVSWYKIEENSSEFLENDVAEWH
PKRGFLLYNELNSVNRSNMMGLNVNVTYTAMFSDSYNHLDDFRDTQLDPVLKTSWIL
TNCAMEMVNMTSTKIIRSSYGEILPNTTRYDGLVGDIQSGIAEIAGNSFYLRSDRIP
IVEYVAPCCPSYLRFIFRAPPLSFVSNIFILPFDLYVWYSCLGLIAIILIVVYLIAV
WESNDPIFKEKEHKSSSPKLEPGIFNALLMELGAITQQGTEAEPKSNSGRIATIITF
ICLMFLFTAYSANIVALLQSTAENIKNPQDLLNYSIALGMRDISFVHYYYNTSTEPI
LKAVYNKMSPKGQKPNFMTLEEGVARMQKEFFCISNRPYVRLQNSRKNLSRKRKMSI
ERTYRF
>PaenIR3
MNRRWGHSGAAAAAVLVLGIVASKFVAVPASSTLPPQVKIGAIFTEDEKNGPNELSF
KYAVNKINKDKTLLPYTSLVYDIQYVPRDDSFHASKKACQLVQYGVHAIFGPSDPLL
GAHIHSICDALDIPHLEARLDMESDVREFSINLHPTQHLLNAAFQDVMTFLNWTKIA
IIYEKEFGLLKLRELVRSPQNNGLEIHLRQANPESYQDVLKEVKDKEIHNIVIDTKP
VNLQHFLKGILQLQMNDYKYHYLFTNFDMETFDLEDFKYNFVNMTAFRIVDTEDVSV
KEILRSMSKSQFNRELRLLNSTFIQAEPALIYDSVFVFAVGLQTLEQSHTLKLSNLS
CDKEQPWDGGLSLINYINSVEMKGLSGPIEFKEGRRIKFKLDLLKLKQPALVKVGEW
HPATGVNITDRAAFFDPGTMNFTLVVTTILETPYVMMHTTNNYTGNNRFYGFCIDIL
DRISQEVGFDYLLDLVPDRKYGARDPSTGLWNGMVLQLMQHKADLAVGSMTINYARE
SVIDFTKPFMNLGISILFKVPKSQQAKLFSFMNPLATHIWLYVLSAYILVSITMFVV
ARFSPCEWQNPHPCEIDNELVKNQFSLANSFWFTIGTLMQQGSDLNPKATSTRIVGG
IWWFFTLIIISSYTANLAAFLTVERMITPIENAEDLAGQTEIPYGTLESGSTMTFFR
DSMIETYKKNVAVHGKQKTERFRPYV
>PaenIR4
YNIKLELSIDKMQPTCFSYLLFIKDVMKSKNVIGEQINSKVIVIARSSQWRVFEFLS
HEESRSFVNLLIIAKSEVGAAANEEAPYILYTHKLFADALGTSKPVVLSSFHHKRLT
RNVHLFPRKLNKGFSGHRFIIAMSHQPPYVISKGVRTIEGEKIFEGIEIRLVNLLSK
IYNFTTDYREATEDARVGSSEAVTKTIESNKANIGIGGIYITPDKISRMGLSTWHSR
DCAAFISLASTALPRYRAILGPFKWTVWLALIFVYLGGICPLAFSDKLTLNHLLRNP
EEIENMFWYVFGTFTNCFTFSGQGSWSRADKFTTKLLIGFYWLFTIIITASYTGSII
AFVTLPVYPSVIDTAQQLASGWYQIGTLDKGEWQYLFLNSTDEVTFKLMKNLELVPS
IEEGLKNTTKYSFWKYAFLGSRAQLDYIVRTNMTTKSKRSVMHISKECFVPFSVSIA
YPINSVYGDVISNGIELIKESGIMNKIENDVEWEMMRSATGKLLSASSSEVLKTLTY
EDRALNLEDTQGMFLLLAFGFVVGGGTLIFEWFGGCYKICRRKSRRASDESIESNPR
IHERQTTRPNNVMLRKPFEKNVNQRRNDENAECKIDYQNHEIFRNDETNKYDEKMDR
IIDEIFDSALKDYRSTPKTRGSSGDLSLIW
>PaenIR5
ISSISSFSFAVENYEIIKLISDFIIKLNVPTKVTAYSCWPKVDELFLWKTLSNNGIP
CEIGKVRMTKRLFFPPEHRLFLLDASCDRSKDLLEIGDRLNLFRHPFKWIIWGETNE
TTFNDNYFRMDSKIFIIYKDAEIYRIKSLYKLSEGSSLYLKNDLAAWEPSKGFLSYD
ELSFYKNRTNLMGLNWNISFVITHQETYKHLEDYRNTYIDALAKHNWFTLKALTSVF

NVTPTHVFQTTWGYREKNSTRFTGMIGDLQSNRADIGGSASFVTEDRLDVVDFVAPS
FSIYLKFIFRAPPLTSVSNVFTLPFNSSVWYACFGLIPIILIVVYLIVKWEWRDVIF
KRKLASNSVTPLRPGLVDVLVMELGAITQQGAESEPKSNAGRIATIFAFVAFMFLFT
SYAANIVAILQSTTDNIKTLEDLLNSRMSCGVEDIPYGHQFFEKAQEPIRKAIYEQK
VAPKGLKPNFMTVQEGINRVQKGFFAFHVEISSGYNVVSDIFREDEKCSLKELTYTK
HTEPWVSVKKGSPYKELVRVGLMRTIETGIQTRHSNRIFTKKPICRTKASNFVSVGI
LDCYAAFLIFIVGASFAIIIYLLEILVYNKINLD
>PaenIR6
MFKNVELYINSDINIMYSNNHHVVDIYNPASTKGGDLKTINLGTYNETSGFSIIKKK
ESKYSLRKNMTGVTLKTIIVLPVDFNGTLLDYLRSKEQPEINTFNRFHYELVNSCVN
YYNFTSEVSRYKSWGYLKTDGTFDGMAGQLERKLIDYGSSPLFYRVDRSKVLDFGRQ
TWTLRAAFIFRDEKKVNWVDVFLKPFTMSVWLSLLFIVVLTIVFLRLIKITEKRNVR
LDAEIGWSYLLINILAVFCQQGIAGVPLFISGRITFLFMLICSLLIYQFYSASIVSR
LLTKPNTEIKSIEDILHSNLKVGCEDTLYTKDYLRYTTNKPTKELYLTKIIGKENSS
NFLSAEDGLVLVKNGGYAFHVELATAYPVITKTFHDRTINELREVQMYMSQPMYFNL
QKKSPFKDTFNTCLQRLAEYGILNRELRLWHPKKPEGIHATMTLTSLSLDYFYPLLT
MLLIGTIMSTIILTLEICLHFRQTTKETETRTPVFQFVH
>PaenIR7
MHKIVILLLCFGIIVSGLKKKTVNVAVFLDDDEHYEDTVIAIASVVRRINLYSSVQY
LLYPHIFKVQKNEILKTGQTACSLLEKGIAAIFGPESIEINDFIQSLSSSLQIPQFQ
TFWNPKLASLPISDQPIQIFNLHPSPKALSKALATLVRENDWKSYTILYEDDNGLLR
LQESLKQLKPDDPAVAFKALGPRENLRSVLKEVQSSGVAHFILDCEAERIMDILRLA
KELKLLSEFHSYILTNLDAHTINWSEVKNIKSNITAMRLLNPDNTNMKNAALVWNQN
MKYSFLETVKDPVILKEIQNLRRMKHFKDIKTINIQTKTALMHDALNIFISTFSKLD
AVYPVAFKALSCESNETSTYGQHFSDILTQINKENPLLLEPLTGPITEFDSMGYRKN
YKLQIVEWVGDRFRISGTWDPALPEKINLTITPAERDEEIMKKIQKRNFRIVSRLGD
PYLMEVADTNGRGLYGNDRFEGYTMDLMKEICKPENLNCTFTFELVPDGNYGSYDPK
TKQWNGIIKELLEYKADLGICDLTITYERRKAVDFTSPFMTLGISILYAKAVKEPPD
LLTFSHPLSFEVWIYIATSYLIISLIMFLTARLNPNDWENPHPCNSSPMELENIWNL
KNCCWLTLGSFMTQGCDLLPKGICTRMVVAMWWFFALIITACYTANMTAFLTSSRMG
VTITSAEDLATQNKIKYGCLKDGATSSFFKDSNFSTYHRMWVQMDSADPSVFEMNNK
EGVKRVLNSKRKYAFIMESSNIEYEMERNCELIQVGSNLDSKGYGIAMPFNAPYRKS
INAAILKMQEMGVLHTLKTRWWKEKKWRWSMYQRQTVRRRGRHRNGSGQRGRRLLGS
RSWCRTFSTYRDIRIPMECKKSRRRPKVDAQRSSCQRIQVRHRYLG
>PaenIR8
MIFAIKTSILVIVLVLFTSVGKIEAEEYKIGAIFDDVNTNQEAALQEAVNEINENDD
DIDLELIVEYIRRDNPYEASRATCHLLEQGVVGILGPLSEDNSNTVQSICDLKEIPH
IEVRWDDYPVNGTLINLHPYPDTLTRTYYDLIVAWGWQNFVILYENNESLQRVGEII
KLFNPRKNRIVLRQLNPVKSGGNFRPVLKEVWKSGATHFLLDCSIEILEEVLKQAQQ
VGLMTDKHHFIITNLDLHTIELMPYQYSETNITGMRFVDPDNKDLTEKAEKIYKQAL
PIFTGYRLRLLEALTFDAVKMFAEALKASEPLVKPTNISCYNDNEKLRSGTTIVNEM
KNLDYTGLTGPIKFDIKGYRTSFALHVFELMEGGQTIVGHWNNSKSPPLNISRLYTS
NEDQGDADIRNKTFRVEITLTEPYGMRVESMQPVYGNDQYEGFVVDIIKELSAIRGF
NYTFVVREDKKNGEYENKTGKWNGIIGDLIDENADLAICDLTITKDRAEVVDFTGPF

MMLGVSILYKKPTKAPPSFFSFADPFAFEVWKLLIVSWVGVSLILFALGRISPTEWE
NPYPCIEEPEFLVNQLDLRNCVWFITGSIMQQGSEIELKSISTRMVAGMWWFFTLLM
VSSYTANLAAFLTTEKPNPHFTNLHELVENADTIGIMVGAKDGGATETFFKSKWLAD
ENSDFGKIYNLMVKDKKKLKVIDNQDGVAAARKGYFAFFMEDKSIEYEIQRKCDLHQ
VGDKLDEKGYGIAMRKNSTYRNSLSTAILLMQNSGKIDEFKRKWWEERKGGGQCPSD
AENGDATPLNLKGVEGVFWVTIGGTILSFFLAMFETTLYCLKKSKRFRYSFFEALKE
EIKFYFRQVTHPSKVDC
>PmacIR1
SSEFIENDIAEWNSKRGFQTYNELSFSRNRTDLKGINLNISHVVTNEDTYNHLEDFR
NTHIDPLSKLNWLIMKNLLVILNATSTPLFVTTWGYMNSNTSKFTGMIGDLQSDNAE
IGGTALFFTIDRIDVIEYIAPSAPTYMKFIFKAPPLSYVSNVFTLPFDTDVWYSCFA
LVPLIFVTIYIIVKWEWKDPMFKEEIQETNSNSIVPLRPGLIDVLVMQLGAITQQGT
ESEPKSNAGRIATIFAFIAFMFLYTSYSANIVAILQSTTESIKTLEDLLNSRISLGV
EDIVYAHYYFKTAKEPTRKAIYEQKIAPKGQKPNFMTMQEGIGRVQKGFFAFHVEVS
SGYKMIGGTFQESEKCSLKEIVFINLVEPWVSIKKRSAYKEIIKVGLRKMLETGIQR
RYINRIYTKKPVCQSKGSNFGSAAILDCYAAFLIFGFGIFLSFFILFFEILANRRIK
SREMCVGVVNSSSGAAKLGGIINFVN
>PmacIR2
KMKLKAIYLFLLGVLRLSLQNNNPDVIGLVKDFLIKLDFPFKITAYVCWSKDIQLHL
WKQMSAQNLVCQMGKFTENIPSYFPPEHLLFLLDANCNGSNILLQQASQLKLFSHPY
KWIVLDQADQKTFKGIFLAVDSRVFIIKKIVESDIYRIVSWYEIEENSSEILENDVA
EWHPKRGFLLYNKLNSANRSNMMGLNVNVTYTALFSDSYNHLDDFRDTQLDPVLKTS
WILTNCAMEMINMTSTKIIRSSYGKILPNTTRYDGLVGDIQSGVAEIAGNSFYLRSD
RIPFVEYVAPCCPSYLRFIFRAPPLSFVSNIFILPFDLNVWYSCLGLIPVILIAVYL
IAVWESKDPIFKEKEHKSSSPKLEPGIFNALLMELGAITQQGAEAEPKSNSGRIATI
ITFICLMFLFTAYSANIVALLQSTAENIKNPEDLLNYSIALGMRDIYLTHYYYKTST
EPIVKAVYNKMSPKGQKPNFMTLEEGVDRMQKEFFAFQTDPTSAYKIIEKTYQENEK
CQLREITVFNAIEPYVAIKKRSPYKEVIKIGMKKIMESGIQRRTIDRIYSKKPKCHG
QGSSFGSAAILDCYAAYVIFGVGVGISFIIFCFEIIFNKKKLNTCLIFGKMSQK
>PmacIR3
MNHRWGHSGAAAAVLVLGIVASKFVAVPVSSTLPPQVKIGAIFTEDEKNGPNELAFK
YAVNKINKDKTLLPYTSLVYDIQYVPRDDSFHASKKACQLVHYGVHAIFGPSDPLLG
AHIHSICDALDIPHLEARLDMESDVREFSINLHPTQHLLNAAFQDVMTFLNWTRIAI
IYEKEFGLLKLRELVRSPQNSGLEIHLRQANPESYQDVLKEVKDKEIHNIVIDTKPM
NLQHFLKGILQLQMNDYKYHYLFTNFDMETFDLEDFKYNFVNMTAFRIVDTEDVSVK
EILRSMSKSQFNRELRLLNSTFIQAEPALIYDSVFVFAVGLQTLEQSHTLKLSNLSC
DKEQPWDGGLSLINYINSVEMKGLSGPIEFKEGRRIKFKLDLLKLKQPALVKVGEWH
PATGVNITDRAAFFDPGTMNFTLVVTTILETPYVMMHTTNNYTGNNRFYGFCIDILD
RISQEVGFDYLLDLVPDRKYGARDPSTGLWNGMVLQLMQHKADLAVGSMTINYARES
VIDFTKPFMNLGISILFKVPKSQQAKLFSFMNPLATHIWLYVLSAYILVSITMFVVA
RFSPCEWQNPHPCEIDNELVKNQFSLANSFWFTIGTLMQQGSDLNPKATSTRIVGGI
WWFFTLIIISSYTANLAAFLTVERMITPIENAEDLAGQTEIPYGTLESGSTMTFFRD
SMIETYKKMWRFMENRKPSVFVPTYEEGIQKVLDGNYAFLMESTMLDYIVQRDCNLT
QIGAPLDSKGYGIATPKGSPWRDKISLAILELQEKGEIQMLYDKWWKKSGDICHRND

KGKESKANSLGVDNIGGVFVVLLCGLAFAVIIAIFEFCYNSKKNALNDKRSTSVPQQ
SLCSEMKSEFCFALRCGGSRQRPALRRKCSKCMSGVSYVPAMLDIPLHPPLPPPPRN
MPSSQMVVTHRSEREVFDH
>PmacIR4
TTVPFSECLEKRSLQKSHEKSREEKWADAFLNRIQLEKQLDLVMLLKKITTQYLSDC
TPIILFDAFTEKNYNQFLKNLIDNLPVAYIQGQIMEDYNIKLDLSIDKMQPTCFTYL
LFMKDVMKSKNVIGEQINSKVIVIARSSQWRVFEFLSHEESRSFVNLLIIAKSEAEV
DANEEAPYILYTHKLFADALGTSKPVVLSSFHHKRLTRNVHLFPRKLNKGFSGHRFI
IAMSHQPPYVISKGVRTIEGEKIFEGIEIRLVNLLSKIYNFTTDYREATEDARVGSS
EAVTKTIESNKANIGIGGIYITPDKISRMGLSTWHSRDCAAFISLASTALPRYRAIL
GPFKWTVWLALIFVYLGGICPLAFSDKLTLKHLLKNPEEIENMFWYVFGTFTNCFTF
SGQGSWSRADKFTTKLLIGFYWLFTIIITASYTGSIIAFVTLPVYPSVIDTAQQLAS
GWYQIGTLDKGEWQYLFLNSSDQVTTKLMKNLELVPSIEEGLKNTTKYSFWKYAFLG
SRAQLDYIVRTNMSTKSKRSVMHISQECFVPFSVSIAYPINSVYGDVISNGIELIKE
SGIMNKIKNDVEWEMMRSATGKLLSASSSGVLKTLTYEDRALNLEDTQGMFLLLAFG
FVIGGGTLIFEWFGGCYKICRRKSRRGSDESIESNPRIHERQTTRPNNVMLRKPLEK
NVNQRRNDENAKCKIDYQNHEIFRNDETNKNDEKMDIIIDEIFDSALKDYRSTPKSR
GSSG
>PmacIR6
MNNSEWNGMIGCLTRKEADIGGTALFFTMDRVDVIEYVAMVTPTKSKLVFRAPKLSY
VTNVFTLPFDRQVWISTVGLVILLGVFIYYILKWEFYKRKESPSPVMKVDQFEVALI
SFGAVCQQSANVIPTSVPGRITTIILFISLMFLYTSYSANIVALLQSTSTSIQTLED
LLKSRLKIGVFDTIYNRHFFPRANDPIRRAIYEQKVAPPGQKPNFLPLDEGVRKIRE
GLFAFHMETGVGYKVVGEIFQEHEKCGLQEISYFVFTDPHIAIQKNSSYRKLLKIGF
RKIWESGIQPREVNLIYTKKPMCTSRSSSFINVGMVDCYSAAVVLVVGLISSLLIWI
VEIIIHNR
>PmacIR7
MEFEIVVGKMLFCVVVAFMVQCYSCEIIVHIGALFEKNENLEKSFNFAIEYVNKNNN
ESEFVFAPLVQNNLTNNNPYLAMTNTCSILEEGVVGVFGPEAFQNVAVVQSLCDLKE
IPHIATRWNYWSARDYIMVNLYPHPQYLSKAYYQIITSKNWKTLTILYEDNESLVRI
LSLIEDAQRGGIPVQLIQLDPFNSGNYRTTMKKLKMTGQTYIVIDCSIRNLSEVLAQ
LQQAGLLNERYSYLLTNLDAHTENLLPFQYSNANVTGVRLVNPEKEYTQTIAQEFFS
EDTTFLEVSAWKLTLETALVIDAMSMFTKVFIDRQKSSSIRIIMENNNSLPCDDTNS
WEHGFSLVNMMKSYEYEGLTGPVKFDNQGLRSNFELTVFELLEGGITDVGTWSSSKG
LNISRPPGANEEDDEESLRNKSFTVQITLTEPYGMLKQTTRHLSGNEMYEGYAIDLI
AKLAEMEGFNYTFIVREDKSNGAYENKTKQWTGMIGDLLNNVSDFAICDFTITSDRE
EVVDFTDPFMSLGISILFRKPETAPPSFFSFADPFGLDTWMCLGVAFFVVSVAFYFM
GRFSSDEWTNPYPCIEEPEFLVNQFSLNNALWFATGALLAQGSEIAPIAISTRMASG
IWWYFCLIMSASYTANLAAFLATENPIKLFTDLQSLYDNQVDIRYGAKHGGATLRYF
TSAEEGTLLWKVGQRMLAHPEDSPKENDVGVAMAETGKYAFFMESSSIDYTIQRHCT
LQQYGDNLDQKGYGIAMRKGSPYRKRLSLAILKLQQNHFLDDLKTKWWEERRGGGAC
SGNKEESEADPLGFKNVEGCFSCTVYGTCLAVVLVVFEHFLYVCKVSLRTKTSLMQV
LKEEWLAYKDFNSDSKPNLMIKSEDEETGRQEEDEEMKSRSTKASRSRSSSISRARL
RHKGRGKSSSSQPYCPESTGSTKSRVKPYGFVLPENNSGAQQSTPQSSHSSKSKRR

SIGFTFSGSNINEKF
>PstrIR1
MRVETCLRFLFCLIFYQLHYNEGSKAVVTIGVLLQELASQINIPLNSTIYKKNMFDQ
RAYFSTKILKVSPSDNFEASQTLCTLLDSDLGVVAVYSDSSRTTPILESTCTFFEVP
FITTSWKAPSPAKRNPPAKRNPEEVRAMINFFPEAELFAKGLAEIVRSLQWPSFIII
YENEEGLIRMQEILKLQEINPDSKKNNIVVMHLGPGPDYRPLLKKIHNTTEDNIILD
CEVDKILPILTQANSVGMLGLHNRYFITSLDAHTLDFSNLNTTANITTIRLHDPKSD
DFLNTVHRWELTEFENHNRRVPLDPYSIKTETVLFHDAILLLTDTVNAMTIKPGINI
KPLACNGTETSDDGFTLRKYMLINTPSMTLTEPLKFNDEGGRADFNIHVVDIIDDSV
IATWHAGNESMELHRNYNQTFDAAVSNLQKITVIVASKIGEPYLMLRQPSYEGEILT
GNNRYEGYSMDLIAGIAKFIGFNFQFEITDKYGNYDYKEKRWNGLIGELLEKRAHLA
VCDFTITPERREVVDFSMPFMTLGIAILHKSQTEKPIDMFKFLEPFSTRVWLYTGTL
YLVISIVLFFISRLTPGDWENPHPCEDDPEELENIWDIKNSLWLTLGSIMTQGCDIL
PKGISSRMAVGMWWFFSLIMTSSYTANLAAFLTKANLEPAIDSAEALSKQTKIKYGL
VKGGSTESFFRNSNFSTYQRMWLSIQQTKPSVYEDSNADGVLRVQTTKLAQYAFLME
STQIEYEIETKCDLKQIGEKLDSKSYGIAMPMNSPYRSAINKAVLVMQESGELGDLK
TKWWKTKRKEASCDNQKSGEEEDANKLGIANLGGVFLVLGVGIAMACVFALIEFLWN
CRNISVEEHITYKQAFMLELKFACNILITKKRVKPLLSEEGSSQQSDKSAENKNGNK
SIINSILHSAGSNLNIVN
>PstrIR2
MFFSILRLSNFIRLLIILSNFSCQYQAQDYREYKIGAIFDETNTKQEAALREAVREI
NEFDNDINLRVIVEHIERDNPYKAIKATCDLLEEDIVGIFGPFSEDNSNTVQSICDL
KEIPHIEARWDDYPQNGTLVNVYPFPDTLTKTYYDLITAWGWKNFVILYENNESLQR
VGELTKLFDPKVHRIVVRQLNSAKSGGNYRSVLKEVWRADATHFLLDCSIEILEEVL
RQAQQVGLMTNKHHFIITNLDLHTIELMPYQYSETNITGMRFVDPENPDLTEKSATI
HSEDPLQIFMGYKMKLSEALMYDAVKMFVEALKASEPLVEPTSISCYNGNDKLRTGT
TIVNMMKSLEYPGLTGFIKFDIKGFRTNFALDVFELMEGGQTVVGHWNESKIPPLNI
SRINLLQEDPGDDIRNKSFKVMITLTEPYGMRVESTDLLFGNDQYEGFAIDIIKELS
ALRGFNYTFLVRADASNGEQDKNTGKWNGIIGDLIDGVADIAICDLTITMKRAQAVD
FTGPFMMLGISILYQKPTKAPPSFFSFADPFAFEVWKLLMLSWIGVSLILFMLGRIS
PAEWENPYPCIEEPEYLVNQLDLRNCVWFITGSIMQQGSEIELKSISTRMIAGMWWF
FTLLMVSSYTANLAAFLTSEKPDPHFNNLHELVENAEFKEIKVGAKSGGATEQFFMD
KYKADETSDFGKAYKLMEKTKETLKINDNKDGVAEAQKGHYAFFMEDQSIEYETQRK
CDLTQVGEKLDEKGYGIAMRKNSSYRNSLSTAILKLQNDGVLDKLKRKWWEERKGGG
QCYDDGASGDATAMNLSAVEGVFWVTIGGSIIAVICALLECLMYTLRKAKRTKIPFS
EAFMDEAKFYFRFNEMTKNVCSNEAESKEGTEENGDLAYGLVNKQNSSETLQSSDSN
KSKRRRSRSKRSKTNLELQPSNVENTYIGWRK
>PstrIR3
MWGVMDRSILDNFYIRLDSRVFIVEKTETDAYRIESPYKVTENSLEYSMNHVAEWNR
IGGFSVYRELSYSRNRSNLMGLNINISYVVTNKDTMNHLEDYRNKHIDPISKLNWFI
MKHLISVLNATSTPLFQPSWGYRDVNDSTKFTGMIGDLQSGKAEFGGTALFFTIDRI
GVIEYIAPSVPTFMKFIFRAPPLSYVSNVFTLPFDTYVWYSCFGLVPLIFVVVYVIV
VWEWKDPVFKEKVGEMHANCISPLRPSFIDVLVMELGAITQQGTDTEPKSNAGRIAT
VFAFIACMFLYTSYSANIVAILQSTTESIKTLDDLLNSRISLGVEDIVYAHYYFKTA

EEPVRKAIYQQKIAPKGQKPNFMTIEDGMSRVQKGFFAFHVEVSNGYKVVADTFQEN
EKCSLKEIAFINLVEPWVSIKRASPYKEIVKVGSRKILESGIQRREINQLYTKKPVC
HSKGSNFDSASILDCYAAFLIFGAGLAVSFLCLTFEILLHRKFKRLMSSNVVLNYEL
PNDDELDKSSVY
>PstrIR4
MLKFIVFIYTCCDVLSSISANQKDWNSQNPTVFNIGGVLSSNESEKYFQETIAHLNF
DSQYVPKGVTYYHTAILMDPNPIRTALNVCKYLISRKVYAVVVSHPITGDLSPAAVS
YTSGFYHIPVIGISSRDSAFSDKNIHVSFLRTVPPYSHQADVWVEMLKHFKYKKVIF
IHSSDTDGRALLGRFQTTSQSLEDDVDIKVQVESVIEFELGLESFKDQLLELKNAQS
RVYLLYASKQDAKVIFRDAAVFNMTDAGFAWIVTEQALEADNVPEGILGLKLVNATN
EKAHIRDSIYVLASALRDMNQTKEITEAPEDCDNSGSIWETGRDLFNFIRKQVLLNG
ETGKVAFDDQGDRINSEYNIVNIQRKKKKVVVGKYFFNKELDRMNLKVEENSILWPG
KQKEKPEGIEIPTHLKVLTIEEKPFVYVRKLMDSQDTCTPDEIECPHYNVSDETSAS
IYCCKGYCMDLLKELSKKINFTYSLALSPDGQFGNYLISNSSVTGKKEWTGLIGELV
GDRAEMIVAPLTINPERAEFIEFSKPFKYQGITILEKKPSRSSTLVSFLQPFSNTLW
ILVMVSVHVVALVLYLLDRFSPFGRFKLANTDGTEEDALNLSSAIWFAWGVLLNSGI
GEGTPRSFSARVLGMVWAGFAMIIVASYTANLAAFLVLERPKTKLTGINDARLRNTM
ENLTCATVKGSAVDMYFRRQVELSNMYRTMEANNYDTAEAAIKDVKEGKLMAFIWDS
SRLEFEAAQDCELVTAGELFGRSGYGIGLQKGSPWSDDVTLAILDFHESGFMESLDN
KWILQGNYQQCEQFEKTPNTLGLKNMAGVFILVGAGIVGGIGLIVIEMAYKKHQIKK
QKRMELARHAADKWRGAVEKRKSMRASANPQRRIKSNGVNEAVTISGVFDKFQRIGQ
FGHERTWPGDADIRQRRTDDVGGGGVQPVPRYLPSYTQDVSHLIV
>PstrIR5
MCLSLFAFCAIGIVACENVNIGAVFEGDEGVEAAFKFAVKNLNDDNPDAEFTFDPLV
YDNFADGDQFVLMRNVCSIIDEGVVAIFGPRSYRNIDVVQSICDVKEIPHIITRWKS
YYIDGDKTIINFYPDSSTLSRAYYDIIANMGWKTFTVLYEGDESLLRIKSLVETAHQ
DGVVTQMMQLDPFYTGNFRDTMRDLKKTQQKSVVIDCHIDSLFQVLTQLQQAGLMNE
QYSYFITNLDTHTENLMPFKYSNANITGIRLVNPDSEYVQKVSSDLLSENLNFLESP
AWKIRLEQALTIDAVNMLAAVVIDRQKSSDVRIITNKNSLLCAEPDSWEHGLSIVNL
LKSYPHDGLTGRVIFDNQGYRSDLILTIFELLEGGITDVGVWSSSYGLNASRPGDVS
GIDDPESMRNKSFVVEIALTEPYGMLKQTTDHLYGNDMYEGYAIDLIEKLAEMEGFN
YTFVVREDKSSGSFDKKTQKWGGMIGDLLDKKADLAICDFTITSDREGAVDFTVPFM
SLGISILFREPESVPPSFFSSADPFALDSWMLLGVSFVTVSLCFYFTGRICSDEWTN
PFPCIEEPEYLVNQFSLSNSFWFATGALLAQGSEIAPIAISTRMASGIWWYFCLIMS
ASYTANLAAFLATENPIKLFTDLQSLYDSQDITYGAKVNGATFNYFTTAKEGLLQKV
GKVLLDHPEYNVLDNDIGVQRAEDGKYAFFMESSSIEYVTQRHCNLMQYGDHLDEKG
YGIAMRKYSPYRKKLSLALLKLQQNHFLDDLKKKWWEERRGGGACEGVVESSEADPL
ELVNVEGCFYMTVFGTILAFGLVLIEHFLYPLRVRKRSGIPFWKIIKAELKAYINFN
SPSKPNLEVLKEIPDEAETEDDKKSEDDKEDSEENRRKSKSASQTRTFTRSRSKSVS
RQSSHSRKKRSKSSGKGTPLSYGFIIPSSLENLKDIEETF
>PstrIR6
MIFPAKIWYFFIFQCALCAVDNVTYLKIAGIFENIQLHQEAFKYSQRISSLKSTPNN
VIVSPVIDPYVLKDEPFTAYKATCSFLRQSVVGIFGPQSALNFDIVQAITDRKDIPH
ILTRWIRPSEMRLQTFNFFPNPDRLADGFVDIINALEWETFTVLYTNEEHLIRSNEF

IRKAKDDGIIVYIENIDPFGDGNYRPVLRNAGRSGQKNFVLDCPIQDLRMLLTQLQQ
VGLLTAEYNYFLTNMDAHTEDLSQFQYSDAVITGVHLIKAKDELALRASQDLCMLYN
ITFKLDCGKPELDIETALIIDSVNVFLQTLNSLGIVEGQYLDCDGEGVWTDGLSVIN
ALRTGSFEGITGQIEFDSHGFRRTFQLTIYQIRDNTTIRGSWNSTDGLSEDIELTYS
DEKEEEDSDLKNKELTVLITLTEPYARLSPTDGEVLKGNGRFEGFAIDLIEEIANME
GFDYTLVVRSDHNHGNFDRKTGKWDGMIGDIIDGRADLAIADLTINKERVDPIEFTL
PFMSVGISILFHKPTVIPPPFFHFAQPFSIRFWEYLAGSYLITVLSLFLIGRLSPSE
WQRPHTCKEDKKYLVNELTLLNSFWFAAAGLFRQPTNVKINSVAAKVIAGAWYIYCF
VLFAMYISYSFANNHVEEKEEMFGNVEEFLRYAEENSIGFGAMKNGATEGFFKNSKS
EVYQEVAKYMEENPNDMMATTTDGIQRVLEGNYAFFMESATIAYTVRRHCNLTSYGG
LLDSKGFGIAVKKGSNLLGPLNRAIIKLQSSGELQRLKNVWWNEKYAGDPCDAGDDS
VPLDKTVPHVNGLIAITFLGIAIAFVSSVLEFTVYAIRLSRKVKAPFGETFGEELKK
CFGKSHTVQHVEEIALTKPENGDSAAKENA
>PstrIR7
MHTDNSFVWAKIGDAKFFYQLSKDILIYGGGCVLIFYDTNTRSEKIFLANSPQGNGD
GIGAVRGHKVLNVFAYSENCAYPRVFVKTYPSFEDIFEIEGNETPESYSYITFTDTL
IIMLGSYPKFTISVRDWVTQQKLCTMDTYAECDKHVLRCNWTKPINMIDLWIGTKKI
AMYEFQMCCKEFHMTQNIVQSSATDGTGNFTSVVWTPEGSMCVCDEFGTIYMMDNEY
SLEKVLDVKISGPSPPAFEWYKNGFLVCGPDKTMRHYVRKLPGEFVLNRSIDIEAEP
ALIYDSVYVFAVGLQTLEQSHTLKLSNLSCDKEQPWDGGLSLINYINSVEIKGLSGP
IEFKEGRRIQFKLDLLKLKQPSLVKVGEWQPGNGVNITDRAAFFDPGTMNVTLVVTT
ILEMPYVMMHTAKNFTGNSRFYGFCIDILGRISQEVGFDYLLDLVPDRKYGARDPNT
GLWNGMVLQLMQHKADLAVGSMTINYARESVIDFTKPFMNLGISILFKVPSSRQAKL
FSFMNPLATNIWLYVLSAYVLVSITMFVVARFSPCEWQNPHPCEIDNELVKNQFSLA
NSFWFTIGTLMQQGSDLNPKATSTRIVGGIWWFFTLIIISSYTANLAAFLTVERMIT
PIEDAEDLAAQTEIPYGTLESGSTMTFFRDSMIETYKKMWRFMENRKPSVFVPTYEE
GIQKVLDGNYAFLMESTMLDYIVQRNCNLTQIGGPLDSKGYGIATPKGSPWRDKISL
AILELQEKGEIQMLYDKWWKNTGDTCQRNDKGKESKANSLGVDNIGGVFVVLLCGLA
FAVLIAIFEFCYNSKKNALNEKRSASVPQESLCSEMKSEFCFALRCSGSRQRPALRR
KCSKCLSGVSYVPAVLDVPPNPPQPPTRGPPPADHSPALGASFDNPRLRI
>PstrIR9
MKLKQILDVGFLLNALIVSVVSAGPAWKEIQRGGGLKIGSGGGKNTSLLRGNGGTSS
GIKLGGSTRGIRTTTTILPEEDHIFAPTTPSVKTGMNHLHIGIMVPYKSFGVREYKK
AASSATAYVQRKLKFFKNHDIQTHLEMQEMTPSPTAILKSLCKEFLSYNVSAILYLM
NYEQYGRSTASAQYFLQLAGYLGIPVIAWNADNSGLERRASQQSSLMLQLAPSLEHQ
TAAMLSILERYKWHQFSVVTSLIAGHDDFIQAVRERVSAMQDRFKFTILNAVLVANR
GDLAALVDSEARVMLLYCTREEAIDILTAARDLHLTGENYVWVVTQSVVENPQQTPY
QFPVGMLGVHFDTSSSSLVNEIMTAVKVYAYGVEDFTSDPDNDGRSLNTQLSCEGAG
AARWDTGDRFFRYLRNVSVEGDQGKPNLEFTQDGVLRAAELKIMNLRPGVSKQLVWE
EIGVWKSWQKEGLDIKDIVWPGNSHTPPQGVPEKFHLKITFLEEPPYINLAPPDPVT
GKCSMDRGVLCRVATDADITEVDMSQAHRNGSYYQCCSGFCIDLLQKFSEELGFTYE
LVRVEDGRWGTNDNGKWNGLIADLVNRKTDMVLTSLMINAEREAVVRN
>PstrIR11
MRACMVILFLSTGLALDPKFVFLKDFFRKKNVPITLVTCPDFKNVTIEAFLSSRTHL

DNSMGFQFYGNRPQIIFNSANHRNWHVIDLIFCGKSSDFLFHMNEINAFKIPFKHLI
LIDISSFDTILLSVCKCDVSIGSEVLLAIFNKTTAEIFETYKFRTTSKCNVLPYGNW
SEKNGITYTTQGASLFQRRRNFQREELVVNAVIKSNKSLQVDFDDIPEATFDEPYFL
ESAASLTYALTYLNASYSFRVYTVFGHLNHTTGKFYGMLSDLAKGIGDITGSALYLS
LERLVVVDLVGNQRSYGTKFVLKEPSMSYVENIYLMTFTDGVWLASGLVLVIFCFIL
FVVINLEGFKKKNDSGKGRKFTISDAVLLSLEALCQQGTSVDSKTIAGRILLVFLFM
VFMFLYAAYAGYILVLLQSTKPIGSVKHLLDSRLECGGVNVSFLTDWYLVNNDPVLK
ELYLKKLKNVGLLSLEKGLSRVREGNFAFHTALNIAYIHIHKTFTDDEICKLQELPG
YLNTDLYYVVPKRSQHKEFFKVSILRINELGFQSRNQYRSSKKPKCYNSIGNFVPVG
FYDCYTIIELFFIGTFLSLIIFIVELGTDKWMRSLKMKRFN
>PstrIR15
MGFISFKIKLLLILFYIFDKTSGLSVGVLLGDYPSQEQRLLNSTISKKFAKGELIFS
ANVQHVSEFDSFGASETLCEVLSSNSGVMAVFGPKYTPATTILESICLEFEIPYISY
SWRPRAERDRGFFMNFFPETDLYANALGEIVRSFGWKSFAVIYEKDETLLKLKNILN
FHQYNEKDKRNKIAFEKLGPGPDHSDLFRKIERSMQTNIILDCKTELIIPLLQQASE
ANLLNVYNNYFLLDLDALTLDYSDLNTTANITTIRLFDDNDESFKESARRIGLAELI
HHRKLKTDIALFYDALWYFHDTMKTTQMLFTDPIYCNGTKKFKKGLELSLAMKNRFF
FTNLTNLTGPILFDVQGSRIDFNIIIVDVLADKKIATWFEQNQTLITNAEFDESITV
VNNLQSTTVIVSSKLGAPYLMLAEPEDGKVLEGNERYVGYSMDLIAEIAKIVGFKFE
FRLAEDGSYGRYEVFEDKWTGIMGDLTENRAHLGICDLTITEEREDAVDFSLPFMEL
GISILFTTTDYFESNSFLFLIGFEQSLWMTIVYCYIAISVCLYIVLRLSPDDWERKY
PCDELDETLVNRWNLKNSLMLTLKALTMQGSDAVPKGKSARLAISMWWLFSLILTSY
YIANIALIANLTSQESAINNVEDLAAQSQIKYGMVMGGSTQEFFKNSNTTVYQKMWN
SIEHLPSVFTKTTQEGIEKVLESNGQYVFFMESTTLDYALERNCKLKQIGGLLDLKH
YGIAMPQNAPYRSTINRAILKLQEDGVLAELKKKWWSVDEEETCDRTVDSSGQLLPE
NIAGLFIILALGIITALSAALFEFLWNVKRVAKLEGITFCEALGDESKMVCSIWTNR
RKLQ
>PstrIR16
MLKFIVFIYTCCDVLSSISANQKDWNSQNPTVFNIGGVLSSNESEKYFQETIAHLNF
DSQYVPKGVTYYHTAILMDPNPIRTALNVCKYLISRKVYAVVVSHPITGDLSPAAVS
YTSGFYHIPVIGISSRDSAFSDKNIHVSFLRTVPPYSHQADVWVEMLKHFKYKKVIF
IHSSDTDGRALLGRFQTTSQSLEDDVDIKVQVESVIEFELGLESFKDQLLELKNAQS
RVYLLYASKQDAKVIFRDAAVFNMTDAGFAWIVTEQALEADNVPEGILGLKLVNATN
EKAHIRDSIYVLASALRDMNQTKEITEAPEDCDNSGSIWETGRDLFNFIRKQVLLNG
ETGKVAFDDQGDRINSEYNIVNIQRKKKKVVVGKYFFNKELDRMNLKVEENSILWPG
KQKEKPEGIEIPTHLKVLTIEEKPFVYVRKLMDSQDTCTPDEIECPHYNVSDETSAS
IYCCKGYCMDLLKELSKKINFTYSLALSPDGQFGNYLIRNSSVAGKKEWTGLIGELV
GDRAEMIVAPLTINPERAEFIEFSKPFKYQGITILEKKPSRSSTLVSFLQPFSNTLW
ILVMVSVHVVALVLYLLDRFSPFGRFKLANTDGTEEDALNLSSAIWFAWGVLLNSGI
GEGTPRSFSARVLGMVWAGFAMIIVASYTANLAAFLVLERPKTKLTGINDARLRNTM
ENLTCATVKGSAVDMYFRRQVELSNMYRTMEANNYDTAEAAIKDVKEGKLMAFIWDS
SRLEFEAAQDCELVTAGELFGRSGYGIGLQKGSPWSDDVTLAILDFHESGFMESLDN
KWILQGNYQQCEQFEKTPNTLGLKNMAGVFILVGAGIVGGIGLIVIEMAYKKHQIKK
QKRMELARHAADKWRGAVEDAKEDKRKSMRASANPQRRIKSNGVNEAVTISGVFDKF

QRIGQFGHERTWPGDADIRQRRTDDVGGGGVQPVPRYLPSYTQDVSHLIV
>PstrIR19
MKEKYQDTITEWYNDFVDSIESLQFETIELNIDTFNKTKACDTLSQGAQIVLDITWG
KQEEAEQLFSSIGIPYVKIDVAITPYLDLLDSYLDLRNATDITLIFEDPWYVDQTLH
YWMNIPKMRMIMTDTLTAGSLKKLRDIRPIPNNFALFATTENMNRLFKLALRENLVI
LPERWNLVFLDFHSNALDRNLIAKGSVNILTLNPELCCYFTSSNDDCNCPRDFDLRK
HFLQKSLTVIADAISGLLKEGTSIETPLCDGKISNEGLQKSFDDRLFSTFANDDFMY
YNYSKIRLRSFGSIGIGNNGSVELIVSYDDGHATLVENKTIQPIKAFYSIGVSHALP
WSYKAKDPETGEMKWTGYCVDFAEKIAEVMNFNFEIVEPKEGTFGEKVNGVWNGVVG
DLVSGRTDLAITALIMTADKEEVIDFVAPYFEQTGITIVMRKPVRKTSLFKFMTVLK
LEVWFSIVAALIVTGFMIWFLDKYSPYSARNNKKAYPYPCREFTLKESFWFALTSFT
PQGGGEAPKSLSGRTLVAAYWLFVVLMLATFTANLAAFLTVERMQVSSPSQTPQKKP
TNLSTAGSSPILGAASEAIENQLHCSEELPNAPVLHQHEVRRGYSLQNVERTDSKRL
HRRQAVPRVGLPHKGAVRPHSAGHQRLEPGGYGRRGLQERRRAPGRRLRLHTRLQRD
QVRDQHELQPDGGRGGVRREALRRGRAARQPHAGRAEQSDSEAAEGPVLRRVAGEVL
ESFGQRGLPEYG
>PstrIR22
MIFPAKIWYFFIFQCALCAVDNVTYLKIAGIFENIQLHQEAFKYSQRISSLKSTPNN
VIVSPVIDPYVLKDEPFTAYKATCSFLRQSVVGIFGPQSALNFDIVQAITDRKDIPH
ILTRWIRPSEMRLQTFNFFPNPDRLADGFVDIINALEWETFTVLYTNEEHLIRSNEF
IRKAKDDGIIVYIENIDPFGDGNYRPVLRNAGRSGQKNFVLDCPIQDLRMLLTQLQQ
VGLLTAEYNYFLTNMDAHTEDLSQFQYSDAVITGVHLIKAKDELALRASQDLCMLYN
ITFKLDCGKPELDIETALIIDSVNVFLQTLNSLGIVEGQYLDCDGEGVWTDGLSVIN
ALRTGSFEGITGQIEFDNHGFRRTFQLTIYQIRDNTTIRGSWNSTDGLSEDIELTYS
DEKEEEDSDLKNKELTVLITLTEPYARLSPTDGEVLKGNGRFEGFAIDLIEEIANME
GFDYTLVVRSDHNHGNFDRKTGKWDGMIGDIIDGRADLAIADLTINKERVDPIEFTL
PFMSVGISILFHKPTVIPPPFFHFAQPFSIRFWEYLAGSYLITVLSLFLIGRLSPSE
WQRPHTCKEDKKYLVNELTLLNSFWFAAAGLFRQPTNVKINSVAAKVIAGAWYIYCF
VLFAMYISYSFANNHVEEKEEMFGNVEEFLRYAEENSIGFGAMKNGATEGFFKNSKS
EVYQEVAKYMEENPNDMMATTTDGIQRVLEGNYAFFMESATIAYTVRRHCNLTSYGG
LLDSKGFGIAVKKGSNLLGPLNRAIIKLQSSGELQRLKNVWWNEKYAGDPCDAEDDS
VPLDKTVPHVNGLIAITFLGIAIAFVSSVLEFTVYAIRLSRKVKAPFGETFGEELKK
CFGKSHTVQHVEEIALTKPENGDSAAKENA
>PstrIR23
MHLLIISSFLQVITCLPDLIPIGGLFHPADDKQEIAFRYAIEKINNDRSILTRSKLS
AQIEKIPPQDSFHASKKVCHLLRTGIAAIFGPQSAHTASHVQSICDTMEIPHLETRW
DYRLRRESCLVNLYPHPTTLSKAYVDLVKAWGWKSFTIIYENNEGLVRLQELLKAHG
PYEFPITVRQLGESSDYRPLLKQIKNSAESHIVLDCSTERIYDVLKQAQQIGMMSDY
HSYLITSLDLHGVNLEEFKYGGTNITAFRLVDPEGQDVRRIVRDWNTEIEIKNRKGE
PASMYKQDVQDNATFVKAETALMYDAVHLFAKALHDLDTSQQIDIKPLSCDAVDFWP
HGYSLINYMKVVEMKGLTGVIKFDHQGFRTDFVLDIIELSREGLKKIGVWNSTEGVN
FTRTYGEAYTQIVEIIQNKTFVVTTLLSAPYVMLKETSEKLSGNAQFEGYAVDLIHE
ISRVLGFNYSIKLVPDGRHGSLNRATKEWDGMIRELLDQKADLAIADLTITYDREQA
VDFTMPFMNLGISILYRKPIKQSPNLFSFLSPLSLDVWIYMATAYLGVSVLLFILAR

FSPYEWDDPHSCRGAPPAVLENQFTLLNSLWFTVGTLMQQGSDIAPK
>PstrIR24
MVSKRIGALLLVLALCDPVKNETFPSLLTTNASIAVVVDREFLVEDYEDARNEIEEY
LVYAKREILKHGGVNVYPYSWTSINVRKDLTAIFSITSCYDTWKLFRLTQKEELVHL
AITESDCPRLPSDTAITIPLVDKGQELPQMLLDLRSEGIYNWKTLVIIYDATLDEDM
VTRVIKAVTQRKYGDVKATGITLMKLEPNLSRTELRDFLSTINPKVLGNNYMCIVSY
HLAGNIMEYAKSLQLTSTINQWMYVISDTNDNFKDIEAFESQLKEGDNIAFLYNSTF
KSSNCKEGRKCHIEELLQAFARSLDQAIQDEYETANQVSEEEWEAIRPTKPERRDFL
LKNMNHYLFKNGACDNCTNWAMKTGETWGVEYDASDQRAEPKVVPVGSWRPGDGPVM
TDELFLHTAHGFRGKLLPMITFHNPPWQILKFNSSGEVIESSGLILDIVKELSRNLN
FTFRLETLNKTSFNSGTSNSTSFDVDSDFTNQVPKALIDMIKNKTVALGACAVTVTE
DLEKVVNFTVPITVLSYTFLVARPKELSRALLFISPFTMDTWLCLAAAIVSMGPLLY
FIHRHSPVYEYKGYPMKGGLASIQNCIWYMYGALLQQGGMHLPYADSARILVGGWWL
VVLVMSTTYCGNLVAFLTFPKVDIPITTLDELIAHKDTVSWSFREGSFLEKELAISN
EPRYKTILERRLKHTSTDNEGLIRSMVEGKHVYIDWKQKLTFIMKRQFLKTDRCDFV
LGLEEFFAEKLALVISPDSPYLPKINDE
>PstrIR25
MSSRWERLGAAIGLVAGVLAVVRAAASSLPPAVKIGAIFTEQEKNGPNELAFKYAIH
KINKDKTLLPYTSLVYDIQYVPRDDSFHASKKACQLVQYGVHAIFGPSDPLLGAHIH
SICDALDIPHIEARLDVDADVREFSINLHPTQYLLNNAFQDVMAFLNWTRIAVIYEK
DYGLLKLRELVGSSQNGGLEIHLRRASPDSYEDVLMEIKNKEIRNIVIDTKPVNLRH
FLKGILQLQMNDYKYHYLFTTFDLEAIDLEDFKYNFVNITAFRIVDNEDVAVKETIR
NMLKFQPNRELRASNGTYIQAEPALIYDSVYVFAVGLQTLEQSHTLKLSNLSCDKEQ
PWDGGLSLINYINSVEIKGLSGPIEFKEGRRIQFKLDLLKLKQPSLVKVGEWQPGNG
VNITDRAAFFDPGTMNVTLVVTTILEMPYVMMHTAKNFTGNSRFYGFCIDILDRISQ
EVGFDYLLDLVPDRKYGARDPNTGLWNGMVLQLMQHKADLAVGSMTINYARESVIDF
TKPFMNLGISILFKVPSSRQAKLFSFMNPLATNIWLYVLSAYVLVSITMFVVARFSP
CEWQNPHPCE
>PstrIR29
MDTDELCNIEFIDLNGVDESEQLRIRNSLQQLDPSVQLPWVKNKKILLRDKQQNNRE
NDGQNVIESVPTSQHHNPYINHPMITQPVYYNQQQSVVVQPIITYPHHTNILYYPTN
VLSPVSPPYPQQVFIPQTNYSTTYPCTIQTGEAEDLQKEENEGILSKENGIEDNGDD
KNETTPNKVPEIIPSSPIIENGNENVETVTSPAPVNKSWASLFSSSKSSNTSSTKPH
PELNIQQTANKKSYENEAFCPIKHPRKSQQFIDPDCYRMGEFLISYAIDGKALSLQP
RGLLNQSNYCYINSILQALVACPPLYNLLTGLAQNISSNGKRKPTPVIDGMCRFVKE
FKYLPPNLRNKDKKAEKNPKKDANVLINTDIPFEPTWIYKMLNGIRTDLIEGRQEDA
EEFLGFLLNGLNDEMLELIKLVKNDKEEPNESVLTTDDNGEKEWKVMGPKNKGSITR
RTDFDRTPISDIFGGLLKSKIHRAGDLSTENIQPFLTLQLNIEKVKTVREALEALVN
KHQLEGLTSSKTNEEVEAWQQVLLDELPVILILHLKCFDYKQAGCTKIIKALEFPVD
LKIDQKLLSSKPQSQKEKHYKLFAVVYHDGKEASKGHYVTDAFHIGYSCWLRYDDAS
VKTVQEEQVLKPQGTRVPYLLFYRRSDTIRGK
>PstrIR33
MRLRWILIFLTETINVTAFSVLFSIIQEYGKDHMISEFSCNGKESFNLLKLASQTYQ
PIRILKIDEKLLQNNVPANTIFVMNLDCNGSRAILRKSDDLNLLSLPYKWVLFHSKP

LGSYEKYFYDLNFSINSEINALQKYKNGSTILRKIYELKGDLQIENMGIWENNIFRS
FDIERITVRRRKNLKNITLNTCIVITNNDSLNHLTDKRDPHIDSISKVNYVLIGHFK
DILNVTLNFTIESTWGYKVNSSWTGMIGLLSRKKIDIGGTSLFFTEDRVDVIDYVAM
ITPTRSYFIFREPKLSYVTNVFILPFDMGVWLSTVALVIIAVFFLYVILNWELTKEI
YLKKSNSYYASNNKPTVLDVVLISFGALCQQASHIVPASVPGRITTIVLFLSLMFLY
TSYSSNIVALLQSSSSSIKNLQDLLNSRLEVGVDDTVFNRFYFPNATEPTRRALYLK
KVAPPGKSDHFMPILDGVKRIREGLFAFHVESGPGYKLIGEIFHEDEKCGLHTIQFL
QVIDPWLAIQKRSPYKKLIQIGFRKLMESGIQIRENTLIYHKKPVCVSRGSAFVSVG
IVDCYPAVVVLVIGIGTSLFVWAIELLNKRFLVTDYIKTMLKNYLKIKESKHEDIVF
DLKE
>PstrIR34
MGLDDTNRTTLVPVLTSNVESCLENRLIARISARLPFITFAMFNMEDVGYSKTRQTR
YNAKFYIFFLDDYRNFTDALAMVRRADSFNPTAFFLVYVEGNFGGNAITAGGILQVA
KINYLPYAAVLVNLDDAFELYQFVYDYGKKKKRKICGTYPKVTVVDTCVNGTMLKPS
KNYNDVRVRENCIVKVVAKEFVPFVLSKTEGFEIDVLQLIGSYLKVTFDVEFYPKSA
LPNWGGKINGTWTGLVKDVAEKTSVGVGNIVVGLMDSKEFSYSQSYHYASLVWVIPK
ALDIPKWRILFALFSPQVWALCLGMVLLFAFVFYLVIKLQGGTPRNSLLTATQILIY
QPVSHLPRNDLHNVFFLGLCVASIILNSVYTSSLLFYMKNPMTEHQASGLHELIDPF
GNPVYEVGGYSKYKSFYNDSETETSRFVYDHYEVSYGENDTLEYWLRRVAHTRRYWT
ISTLLYAKYVDALGKNITRTEDGKSMIFVRSKKPVSVNGVGLIMRRSHPLLGKINKI
IQHMLYGGIQLKIRSKYWRVIEKMESLDNSIDMADEPLTFYHLEGAFAFLILGYVLG
FLILAMEILIYRCKKRKNRKMKDIRHLKRKKNNKVHEIR
>PstrIR35
MAFLLPFSPELWIAIFTSLNITAIAVAIYEWLSPFGLNPWGRQRSKNFSMSSALWVM
WGLLCGHLVAFKAPKSWPNKFLINVWGGFSVIFVASYTANIAALIAGLFFHNTVSNY
HDRSLLSQKVGAPRSSVAEYYVQKANQLLWQHMHKYSLNSIEEGVLKLRNGSLDILI
ADTPILDYYRATDHGCKLQKIGDTINEDAYAIGMTKGFPLKDSISAVIAKYSNNGYM
DILQEKWYGGLPCFKLATEIAQPRPLGVAAVTGVFILLGVGMALGLLILLVEHLFFR
YTLPILRDKPKGSIWRSRNIMFFSQKLYRFINCVELVSPHHAARELVHTIRQGQITS
LFQKSIKRKEHEQRRRRKSKAQFFEMIQEIRSTIRRQQQEEREPPLESVTEVDAENQ
QSPEEKKSKLSPSIIRRTFLRSSPKADNGNKSKSPTTYFNKQLFSPRSKSRNKSTTN
LNVRRFSTDSVFNSTSPGKEDYCTVVGRRLSKDASAFLSSSPPDINSRRSSYLDIIS
SGSKLTGKSPILSIENLSDCSKYSEPMRKLSDSESIGKKLATLPRYQQDSFDKHKLL
QPQYSLTLGRSDEALNSVSKSDTVGQTRSFNNITRPDLNEKSNLSDDEIARRNRETI
IQLQSTAIRNPKPPLKRTKDNNSKPSSSLTNPQIRIQVEDTSEHDTRHPPLSKKPKT
RHESLGDAAGPSETSTSKRNRLTDQSRSLDSANPNSSDRRRRRSGSRSKSREEDLPP
APPPPNCSPRNSDGRSPLERLSKDELVLLWRSSESELRSHLLKALRDKEDAAEPP
>PstrIR36
LSGRRGRLRRDARSLFVCETLDRSLRRESGQAMGSANFGLGVVFVTFVSGILASAEK
IPLGAIFEQGTDDVQTAFKFAMLQHNQNVSSRKFEFQAYVDVINTADAFKLSRLICT
QFSRGVYSMLGAVSPDSFDTLHSYSNTFQMPFVTPWFPEKVLAPSSGFMDYAVSMRP
EYHKAIIDTVKYYGWRQIIYLYDSNDGLLRLQQIYQSLTPGGEMFQVQTVRRITNVS
EALVFLRGIEEQSRWSNKYVVLDCSTETAKEIVVGHVRDISLGRRNYHYLLSGLVMD
ERWESEVVEYGAINITGFRIVDSYKPYVKDFLEGWKRLDPAQSPGAGKDSVSAQAAL

MYDAVFVLVEAFNKVLRKKPDLFRTNVRRGIAFNGTTKSLDCNTNGNWVTPWEHGDK
ISRFLRKIEIEGLTGEIRFSDEGRRQNYTLHVVEMTVNSAMVKVAEWSDIAGFNPVA
AKYVRLKANAHIERNKTYIVTTIVEEPYIMLRKPEPGENLSGNDRFEGYCKDLANLI
AQRLGINYEIRIVKDGKYGTENHEVKGGWDGMVGELVRQEADMAIAPITITSERERV
IDFSKPFMSLGISIMIKKPMKQKPGVFSFLNPLSKEIWVSILFAYIGVSIVLFVVSR
FSPYEWRLLHLSADSRDPNNGQTAMSNDFTMVNNNGQTAMSNDFTMVNLSPRSISGR
IVGACWWFFTLILISSYTANLAAFLTVERMVTPINSPEDLASQTEVEYGTLFHGATW
DFFRRSQLSPYSKMWEFMNSRKHVFVRTYDEGIKRVRSSKGKFALLIESPKNDYTNE
REPCDTMKVGRNFDAKGFGIATPLGSPL
>PstrIR37
MRLNFYSFFFIFAIISSVVCSKTRYVHGMHRYYTKHKLSNFNEETFALASADAEIQH
QHHLRHQHEEVPWPVKKEAVVEGDLVLGGLMMVHEREESITCGPVMPQGGVQALETM
LYTLDRVNKDEEAVLPGISLGAHILDDCDKDTYGLEMAVDFIKGSISNIDGAEYHCN
KTQVRKVISGVVGAASSVTSIQVANLLRLFKIPQVSFFSTSPELSNKQRFEYFSRTI
PSDFYQVKAMVDIVRKLGWSYVSIIYEESNYGIKAFEELEDTLSKYSICIAIKEKLV
KDSGVAKESVYDSIIQKLMTKPRARGCIVFGSDQEVAELMKAVRRCNATGYFSWIGS
DGWSARSLVSEGNEAEVEGTLSVQPQAGEVAGFKEYFLGLNVENNTRNPWFIEFWER
HFNCRYPNSSKTPYNMKIVNVCNGTEKLTANNTVFEDQLQFVSDAVLAFAYAIRDMH
RDHCHGTTGLCDAMKPTNGTELLRYLRKVDFIGLSGDRFHFDENGDAPARYNIKHFK
QVAYGKFAWITVGEYLEGVLKLNMSAIQFKLDQPTPPESVCSLPCLKGQAMKYVEGE
SCCWHCFNCTQYQIRHKDDPTQCVKCPKGTIPDPEHEHCEDIPEEYLRPVSGWAIGA
MAFSSTGVLVTAFVFSVFMKHNETPVVRASGRELSYVLLSGILMCYSVTYTLVLKPT
DVVCAIQRFSAGFCFTVVYAALLTKTNRISRIFNASKHSAKRPNFISPRSQLVICFG
LVGVQVLINGIWMLISPPKAIHHYPTREDNLLVCSSYVDASYM
>PstrIR42
MHSLLNRKLFSFPQKMKLELLTVFLLIELTTMYSIHDDLTETTKDTTFGLLLQEIIV
KSLSPYKCIMIFTDEINSDVFQRKWFKRFGTAVSYVLVKVKDYDDLYAPSPEIQLSL
HVANNGDCQLYIFLISNGIQMRRLLKFGDRYRAISTFTKFILLHDNRLFEKRFLYLW
KRIINVIFIKKFESKVIKITNSTKENAWFEITTVPFPLPLTNILIPKKVDIWAQSKF
RKGADLFRDKTKDLQNQTLKVAIFRHLPGVEKVNNSESKNMRALMSTTHNETSYFAG
TEIEILQTISKSMNFKCEIYEPEGAEEELWGRKLVGGIYTGVIGEIVSTKADIAVGD
FYYTSYLLELMDLTVPYNTECLTFVTPEALTDNSWKTLILPFSSIMWGGLIISLLIC
MVTFHYLATYYVKISKTKSPDSQTNSISKFKRVLTLSLQPDFEKIDPNTKYVLMKEK
YQVIKKEGQPEGLYQFSEPVNNALYTYSMLLLVSLPKLPTGWSLRMLTGWYWLYCLL
VVTAYRASLTAILARPVPKVTIDTVQELVDSKLTFGGWGEINLDFFKTSKDPAVKQI
GENFVIVNDSNGAVDRVAEASFAFYENTYFLKEAIVKRQQRFRSFFVNSNATNATSE
ERKAMLQDVQNDRPLHIMEDCIIKIPVSIALQKNSPLKARMDKYIRRVLEAGFIKKW
LGDVMQKVLIAEIQIEDTESTKALMNMKKFSGALVALLIGYVISILTLISEVLYFHF
RVKKNPHFNTYSKQIVIKKAK
>PstrIR43
MGYAEIFFAGLCLNATCDVDLDNVTTSVPSYFQLKFAELEEELKTETLVLTTIQNGE
LSGYQKIGDDLVGNGIAFDIIQILQNKYKFNYTIVLPEDETFLGASNQRTAKKLLEK
EEVDMAVAFLPIIESIRNEIVYSTSFDVAEWVVLMNRPKESATGSGLLAPFTTEVWI
MIIFSILTVGPLIFLMVLIRARLCKNDDSDVYSLSTCLWFVYGALLKQGSTVNPRTD

TSRLLFSTWWLFILILTAFYTANLTAFLTLSKFTLPITAPEDIGKKHYRWVTNKGNP
VRDQILADKNDEHIYQEKLIEKLGNLKVFANYPDMEILDSYVYKRDMMFIREKTVIN
HIMYKDYRQKANGGMEESKRCTYVIAKFPIVKFSRAFAYSKNFKYKELFNYAIQRLI
EGGIIEFKLKENLPDAEICPLDLGSTERKLRNTDLLLTYIIVGGGLAVALCVFLMEV
LWRVCQRRYRKRKAGRMRANGWPNNHKSRAFRSNLQVTPPPSYQTLFKPPFYYERGG
TKKFVNGRDYWIVDGKNGLKQMIPLRTPSALLFQFSN
>PstrIR46
MRVETCLRFLFCLIFYQLHYNEGSKAVVTIGVLLQELASQINIPLNSTIYKKNMFDQ
RAYFSTKILKVSPSDNFEASQTLCTLLDSDLGVVAVYSDSSRTTPILESTCTFFEVP
FITTSWKAPSPAKRNPEEVRAMINFFPEAELFAKGLAEIVRSLQWPSFIIIYENEEG
LIRMQEILKLQEINPDSKKNNIVVMHLGPGPDYRPLLKKIHNTTEDNIILDCEVDKI
LPILTQANSVGMLGLHNRYFITSLDAHTLDFSNLNTTANITTIRLHDPKSDDFLNTV
HRWELTEFENHNRRVPLDPYSIKTETVLFHDAILLLTDTVNAMTIKPGINIKPLACN
GTETSDDGFTLRKYMLINTPSMTLTGPLKFNDEGGRTDFNIHVVDIIDDSVIATWHA
GNESMELHRNYNQTFDAAVSNLQKITVIVASKIGEPYLMLRQPSYEGEILTGNNRYE
GYSMDLIAGIAKFIGFNFQFEITDKYGNYDYKEKRWNGLIGELLEKRAHLAVCDFTI
TPERREVVDFSMPFMTLGIAILHKSQTEKPIDMFKFLEPFSTRVWLYTGTLYLVISI
VLFFISR
>PstrIR47
MRPVLLLLTLFRVITGDQQSVTVDLISDFLLKLNAPTKINAHICWPKEEQIALLKRL
SSSNFGCRIGNFNNISYSTPAEHQLFLLDLSCKGSEKILNKASELNLFNQPFRWLMW
GVMDRSILDNFYIRLDSRVFIVEKTETDAYRIESPYKVTENSLEYSMNHVAEWNRIG
GFSVYRELSYSRNRSNLMGLNINISYVVTNKDTMNHLEDYRNKHIDPISKLNWFIMK
HLISLLNATSTPLFQPSWGYRDVNDSTKFTGMIGDLQSGKAEFGGTALFFTIDRIDV
IEYIAPSVPTFMKFIFRAPPLSYVSNVFTLPFDTYVWYSCFGLVPLIFVVVYVIVVW
EWKDPVFKEKVGEMHANCISPLRPSFIDVLVMELGAITQQGTDTEPKSNAGRIATVF
AFIACMFLYTSYSANIVAILQSTTESIKTLDDLLNSRISLGVEDIVYAHYYFKTAEE
PVRKAIYQQKIAPKGQKPNFMTIEDGMSRVQKGFFAFHVEVSNGYKVVADTFQENEK
CSLKEIAFINLVEPWVSIKRASPYKEIVKVGLRKILESGIQRREINQLYTKKPVCHS
KGSNFDSASILDCYAAFLIFGAGLAVSFLCLTFEILLHRKFKRLMSSNVVLNYELPN
DDELAKSSVY
>PstrIR49
MIKMIKMIMRLALFLLLVAQEGRCQTTQNINVLFVNEEGNEVADRALDVALTYLKKN
TKLGISVDLRRVVGNRTDSNSFLDSLCSTYNQLLQTQVYPHLVLDTTMTGLGSETVK
SFTAALALPTVSASFGQEGDLRQWRNIDQDEQDYLVQICPPADVIPEIVRSLVLNQN
VTNAAILFDTSFVMDHKYKSLLQNVATRHLITPIKDGNDVVEQLNQLNKLDLYNYFV
LASIANIKRVLDAADTLGFFNRKFAWHAITQDEGELKCICKNATIMYVKPLPNATYQ
DRLGTMQRTYQLNTEPIIASAFYFDLALHSFIAIKDLIADGVWKNAMGDYITCDDFN
GNNAPKRKGLDLKKYLNKVSSQVLRVEPANSVGLVQETSEMFTYGPISVVSNGLSYM
DFQMTLSSVGVREGASDKSLALGTWTSGFDRNLTLVDPKAMSNLTADVVYRVVTVEQ
KPFIFRDETAPKGFNGYCIDLIDKIADILKFDYELVAVDKFGTMDENGKWNGMVREL
MEKRADVALGSMSVMAERENVIDFTVPYYDLVGISILMKLPETLTSLFKFLTVLEND
VWLCILAAYFFTSFLMWVFDRWSPYSYQNNREKYKDDEEKREFNFKECLWFCMTSLT
PQGGGEAPKNLSGRLVAATWWLFGFIIIASYTANLAAFLTVSRLDTPIESLDDLSKQ

YKIQYAPLNGSSVQTYFERMANIEARFYEIWKDMSLNDSLSEVERSKLAVWDYPVSD
KYTKMWQAMKEAGLPDSLDEAVRRVRASKSSSEGFAFLGDATDVKYLEITSCDLTAV
GEEFSRKPYAIGVQQGSPLKDQFNTAILQLLNRRELERLKEKWWNKNPEKKNCEQVD
DQSDGISIQNIGGVFIVIFVGIGLACVTLAFEYWWYKYRKGGRVVDVQEALPQNKQT
NLGDDDAPPFSKGKSFKAKMAFPRARF
>CbowIR2
MVTEFSCDKLGSFEFLKKLITQGIPTRILLIDKDELSYLFPPNCQIFIVNLQCENST
NILKKANSLKLFSFPFRWIIYHHEPINETIFEESFLSLDILVDSDVTLLEENKNKSV
SATKIYKRHRNHPLVIEKMGYWTKTAGLRDDREEKIMVRRRKNLQQIPLNTCIVITH
NDSLKHLTDKRDKHIDSIAKVNYVLVEHLSDIVNVTLNYSIQNTWGYKNNKSEWSGM
IGELTKNEADIGGTPLFFIIDRVDIIDYIAMTTPTRSKFVFREPKLSYVTNVFTLPF
DDYVWASTIALVCIISMVLFIILKWEWKKKDLPSEKDSSNVPELKDSLTDVILFSFG
AFCQQGAPSIPFSVPGRITTIILFVSLMFLYTSYSANIVALLQSSSTSIQTLEDLLK
SRLQVGVDDTVFNRFYFPNASEAVRRAIYLQKVAPPGKKENFMSIEEGVKRMRQGLF
AFHMETGPGYKLVGEMFHESEKCGLKEIQYLQVIDPWLAIQKNSSYKELLKIGLRQI
QESGLQTREVSLIYTKKPICTSRGSSFISVGLVDCYPAAVVSAGGAILALIVWILEL
GLYYRPYMWISVKKVFAKSHEKIPGSIEQWPEWPYLK
>CbowIR5
MGLIEFVVASLCLNATCEPEDAVVPGVSTHLLKLNELAEELKEETLTVTTFENGQLS
GYISQNGSFLGTGVAFDIFHILQEKFGFNYTIVLPDADIFMDGFNKKGAKSLLEAKQ
ADIAVSFLPVIESFRNDVVYSRVFDIAEWNVLMNRPKESATGSGLLAPFTTAVWILI
IFSVLVVGPIMYLMILIRAKMCKDDNNKIFSLPSCMWFVYGALLKQGSTLNPKSDSS
RILFSTWWLFILILTAFYTANLTAFLTLSKFTLPITDPTDISRKNYHWVTNKANGLR
DYIEYEKHDRLSNGRTLVQDIGKDRYYADMKDLDILEEYVKKRNMMFIREKTLIKNV
MYRDYQEKTKRGVDEEKRCTFVMADFPITMFSRGFAYTHDFKYAELFDRTFQYLIEA
GIIQFKLRENLPDAEICPLNLGSIERKLRNTDLMLTYVIVASGLGIAASVFLLEILW
RMSKAKYKRTRKRKATTWLEKNNNLMKAKCLHLHTNSSPPPPYQALFRPPFYYSDRD
GGQKKTINGRDYWVIDKSDGLREIIPIRTPSALLFQISN
>CbowIR6
MNLRTKMGLYKNILLFQLLLGYCEGQTTQNINVLFVNEEGNEVAEKALDVALTYLKK
NNKLGISVDIRKVVGNRTDSNAFLESLCSTYSSMLDAQAYPHLVLDTTMTGLGSETV
KTFTQALALPTISASFGQEGDLRQWRNIDDNEKDFLIQISPPADIIPEIVRTLVLNQ
NITNAAILFDKSFVMDHKYKSLLQNVATRHIITAIKDGNQVVDQLSQLRKLDLVNFF
VLASLKNIKRVLDAADSVGFFNRKFAWHVITQDDGEIKCVCRNATIMFVKPLPNAAY
QDRLGTMKRTYQLNVEPIISSAFYFDLTLHSFLAIKEMISDGVWKSVTNYITCDDYN
TENVPKRNGLNLKKYFNKESTESPTYGPITVLSNGLSYMEFQMQLTSVGVRDGASDK
STILGTWSAGFYNNLTIVEQQVMVNLTADVVYRVVTVEQKPFMFRDESSPRGYSGYC
IDLIEKIADILKFDYEIATVDCFGTMDENGKWNGVVKELMEKRADIGLGSMSVMAER
ENVIDFTVPYYDLVGITVLMKLPETQSSLFKFLTVLENEVWLCILAAYFFTSFLMWV
FDRWSPYSYQNNREKYKDDEEKREFNLKECLWFCMTSLTPQGGGEAPKNLSGRLVAA
TWWLFGFIIIASYTANLAAFLTVSRLDTPIESLDDLSKQYKIQYAPLNGSSTQTYFE
RMANIEERFYQIWKDMSLNDSLSEVERAKLAVWDYPVSDKYTKMWQAMKEAGLPNTM
DEAVAKVRASKSSTEGFAFLGDATDIKYMELTNCDLTVVGEEFSRKPYAIAVQQGSP
LKDQFNTAILQLLNRRELERLKEKWWNKNPEKKDCEKADDQSDGISIQNIGGVFIVI

FVGIGLACVTLAIEYWWYKYRKGSKIIDVREVAHNPTKPPTFPKQKFSEHNPDNTKP
KLPKRSKF
>CbowIR8a
MRNVKIILLENEGQDTILTWYRSIVQAFKSPIKFEEFLISVDGEEFDRERICQAFSN
GAMMILDLTWTGNDLARTVSMEMDVPYLRIDVSLSPFFDLLHEYLNFRNSTDVALIF
DDPSRIDQAIYYWIDNVQIAMSISESLDAMAAKKLRDFRPTPNSFAIFAETKNMEKM
FKIALEENLVTLPERWNLVFLDFHHKSFDRGLLKKMPVNLLTLDAGLCCQLLNLNSY
CECPSRFNTSKMFLKIALNMLVTAIEELFKDDFKFHDNIDCDSNFTKDNEESVRKTF
EEVLNKAVGNDNLIRLDNSSNLRLKTTGSIEIGTDVGTEVFAKYENEAITALRNKIV
KPIKAFYRVGITHALPWSYQIKDPVTKKLVWTGYCVDFTAKLAEKMNFDYELVEPKK
GTFGKKHNGVWDGVVGDLASGQTDLAITALIMTADKEEVIDFVAPYFEQTGITIVMR
KPVRKTSLFKFMTVLKLEVWLSIVAALIVTGFMVWFLDKYSPYSARNNKKAYPYPCR
KFTLKESFWFALTSFTPQGGGEAPKALSGRTLVAAYWLFVVLMLATFTANLAAFLTV
ERMQAPVQSLEQLARQSRINYTVVSNSQTHKYFINMKFAEDTLYRMWKELTLNASTD
DSRYRVWDYPIREQYGHILLAINDSNPVANAEEGFKNVNEHLDADYAFIHDSSEIKY
EISRNCNLTEVGEVFAEKPYAVAVQQGSHLQDGISKMILLLQKDRFFEGLQAKYWNN
SVKGDCPNTDDNEGITLESLGGVFIATLFGLALAMITLAGEVLYYRRKRKTKELNIK
QSKVFPEKPLDVFPPKPLLLGNNQITIGNTFKPVNLKEKIRKEREAMKISHISLYPR
ARKPINPFEIK
>CbowIR41a
LFLPGDNLLENLSHIYSMQELKYIADIVIVNREYKDEDSQGLYMSDNVFSLWTHSYR
GMDENAKRMFLDLWFSKNQSFMLDENLYPDKLVNQMGRKLEMATFQYEPYSIIGSSE
TESKGSEMVTCLTFARHYNMTPVLVVNDEGYWGDIFDNWTGYGLLGNLVEDKADIGF
SALYTWESDYYFLDLSKPLVRTGITCLVPAPSLAAGWTTPLYSFSTTMWAAVGSMFF
VCIFVQFFMHYFHAKIYDDTNQSLKLLDRSILCVLKLFVQQVVTTRETPPGRSGKYF
MGLLFTFSLFLSSSYSSGLSSIMTIPRYGRPINTVEEFAESKISWGATQDAWTMSLK
GVEDPT
>CbowIR75q
MLNSLTLFMILFLNFSSAMKNYTEIINIVDELLTKQNIPSEVTAYLCWSKVLKANLF
KRLSASNILTKIIATDDIVDLFPSEYQIYLVDLDCEGSNEILKKAQRKKLFSRPFRW
VFCGNIEQPLFNDLYFGVDSRIFFIDNAGSEYHIKMPYKREKNSKKFTVNDLAEWNS
LQGFTRFDEFAAARNRTDLFGMNINISCVYTDSDTLNHLEDYRNIHIDPLTKLSWIL
VHHLMSILNASATVIFRNTWGYRDSNTSLFSGMIGDLQTGEAELGGTASFFTIDRID
VVEFYASSAPTYMKFIFRAPPLSYVTNVFTLPFHTYVWYCSFLLLVLIFFAIYVIVK
WEWKDVVFREKLERMHDGSISPLRPTFFSVLLMEIGAITQQGTDSEPKSNAGRIATI
FTFIALMFMYTSYSANIVALLQSTTESIRTLEDLLNYRISLGVQDIVYAHHYFEVRI
QLSIDSYPNQTPGLKWLNTQDLGKLWR
>TcasIR40a
MRRDHGGDLVSASFDIVAGFLFEEICICFDKNTNINFLQHLLVRFVSNNIAIKLFNI
TTVEVQ
DKYFAFLNYQVTNHLGANTIFFSSHKFYEHVLLEINERDFIRRNLIYIFNWGRRPFS
RYFVRN
IINVMKVFVITNPRNDTFRIFYNQAVPYKKHHLEMVNWWQHGVGLFNHPTLPAKYNN
V

FKDFKENVFKIPVIHKPPWHFVQYGNDSIKVTGGRDDRILSLLSKKLNFRYDYFDPP
ERIQ
GSSASENGTFKGVLGLIWKRQAEFFIGDVALSHERANYVEFSFITLADSGAFITHAP
SKLNE
ALALLRPFQWQVWPAIGVTFVVVGPVLYAIIALPNAWRPRFRVRSHARLFFDCTWFT
TTV
LLKQTGKEPSSSHKARFFIIILSISSTYVINDMYSANLTSLLAKPGREKAINNLNQL
EKAMAT
RGYDLYVERHSSSYSLFENGTGIYSRLWQMMNRRQTHFLLESVEEGVQLVRDSTNKA
VIA
GRETLFFDIQRFGASNFHLSEKLNTAYSAIALQLGCPYIEEINKILMAIFEAGIITK
MTENEY
EQLGKKKQTTSETEKELIPGVKKENRRVAKVSEDNEKLQPISIKMLQGTFYLLCIGN
IFSGF
ILLAEILVYKHRKTYKHKKRRHRFVYLRKIRHSVASKFGAVVDAVRRVYRRAMHDAF
VAT
LEYLE
>TcasIR21a
MQRGLIVLKLCLTALALKSLDKRALQKSHEKSQLEKWEDKFLNRDPSFDQTASLVNL
ISK
VALDELSGCSATILYDKFTETSSDLLLEKLFRTFPIPYLHGQITDKYHMKVPKLQTS
QDTCT
GYILFLKDVMRSKDVVGPQTNNKVVLVSRSSQWRVYEFLASEQSQSFMNLLVIAKSE
KIV
SSSIARLICLALHLKFGTALAIYAPNGGKSAVYPSVIANVPKLGFRSAESVTSVITQ
NGANL
GIGGLYITDTRLKATDMSHIHSQDCAAFISLASTALPRYRAIMGPFHWTVWLSLTLV
YLFAI
FPLAFSDKHTLRHLLDKPEEVENMFWYVFGTFTNAFSFFGKDSWSKTDKFATRLLIG
FYW
IFTIIVTACYTGSIIAFVTLPVFPATVDTPEQLVRGKYTVGTLDKGGWQYWFENSTD
PITQK
LLTRIDFVPDIESGLKNTTKAFFWPYAFLGSRAQLDYIVRTNFTTINKRSLLHISSE
CFVPFG
VSIIYNKNALYSKIIDQGVLQAVQSGIVDKIKNDVEWETMRSASGKLLAANSYGKSL
KALT
VDDRALTLDDTQGMFLLLGIGFLLGGASLLSEWMGGCLHLCKGNRNQSATSIQSNYR
SHE
VPTPREKLDSMQFNSFENHKIEEEIVEERNCIIHRQDDDDIEEHINRLFDFEGVFGE
ANPDS
RTGPEEELSFKNTTKAFFSLYAFLDSRAQLDYIVRTYFTSMNKRSLLHISSECFVPF
GVSIIY
NKNALYSKIIDQGVLQAVQSGIVDKIKNDVEWETMRSASGKLLAANSYGKSLKALTV
DD

RALTLDDTQGMFLLLGIGFLLGGASLLSEWMGGCLHLCKGKRNQSATSIQSNYRSHE
VPT
PREKLDSMQFNSFENHKIEEEIVEERNCIIHRQDDDDIEEHINRLFDFEGVFGEANP
DSRTG
PEEELSEENGKK
>TcasIR76b
MGLFEIALAALCLNATCPGEEEPPEFPEVQYLAPDSNDRKTLFAQLTEQLKNENLII
TTLKN
DRLSGTEKRNNTILGKGIAFDLLNILQDKFQFNYTLIEPKANVWGAEKFGVLDLLKD
KKA
NLSAAFLPVLTQYSNHISYSPSLDTGEWVVLMKRPKESATGSGLLAPFNLPVWLLIL
LSLV
VVGPVIYFIIYLQAKLCKDDNNKVFPLPACIWFVYGALLKQGTTLNPMTDSSRLLFA
TWWI
FITILTAFYTANLTAFLTLSKFTLPITEPKDIGEKRYKWVTTKGNALEDTVTVNESL
TELGKI
LGQPQRYLYVSDSDILRNYVHKRNWMFIREKPIVEYVMYDDYKEKTRNQIEEAKRCT
YVI
TKFSVVSFSRAFAYSKDFKYKPLFDSTLVQIVKCHKCFSLLSRIQYLVESGIIKFKL
REELPD
TEICPHNLGNKERQLRNSDLLMTYEIVGGGFIISAIVFIIEVIIRRQKKPKTKSLPL
QNPNKH
TFEINLNNNYEKFGHFPYSSKFVTPPPPYHTLFNPPHKSDNMKKRNFNGREYWVYDS
ISG
ETKMIPMRTPSALLFQYTN
>TcasIR93a
MLLELVLSSAFVCVIRGDSFPSLLTTNATLAVIIDREFLSNEYEVIKHAIESYLVFA
KREILKH
GGVNVQYYSWTTINIKKDVTAIFSIASCPDTWRLFRQARDANLLHMAISESDCPRLP
PDEA
ITVPLITRGEELPQLLLDLRTRQTYNWNSAFILYDDTLSRDQVTRVVKSITAQYSNL
RVNAA
AISFVKLETRLPMDEIRRQVKEILSSVSIKTVGGNFLAIIGYELVELLMEYAKMFGL
VNTRT
QWLYIISNTHFRHKDINRFRQLLSEGDNIAFLYNNTVNNDTCTGGIQCHCEEILSGF
TRALD
EAILFEWETSSQVSDEEWEAIRPSKLDRRNSLLQGIKTFLLQRGQCDNCTSWLMKTG
DTW
GREYQQNGTDSGGLISVGNWRPSDGPSMSDELFPHIVHGFRKRNLPIVTFHNPPWQI
IRSN
ESGAVSEYAGVIFELIKELSKNLNFTYTVELAKIGQEFSANLTKNEAQVVTNFIPDS
ILDMIR
NKSVAFGACAFTVTEESKRLINFTSPISTQTYTFLVSRPRELSRALLFMSPFTGDTW
LCLSA

SIVSMGPILYYIHKYSPVYEYKGLSKRGLSSVQNCIWYMYGALLQQGGMHLPQADSA
RII
VGAWWLVVLVLATTYCGNLVAFLTFPKIDIPITTIDELLAHSGTVTWSMPKGSYLER
TLKY
TTEPRFRYLFDKKVEVGNFKNMIEDIENGKHVHIDWKIKLQYIMKQQYLDSDRCDLA
LGL
DEFLNEQLAMVVSQDTPYLEIINDEIKKLHQVGLIQKWLTDYLPKKDRCWKNNRHIV
EVN
NHTVNMDDMQGSFFVLFLGFLLSFFITIGEKLWHKYVTKKKMKIIQPFTT
>TcasIR64a.1
NKISLILVILSKTETYIIKSCLSNAIVDFAILANVAFSLRISCYKLFMHIKLIANVF
YNQLDQV
LNRNHYHLAVIIDSGCIDYADFAIQDKKYFYETYHWLVPTTPQNLNNSLNFLQKSPL
NINS
DVNVAILNGEGTKWSILDVYNPASSHHGQFTVTKLGLCDETNGYQAKIAGNKYWSRK
N
MTGVQFKSAVVVPDPSIKLNDYLTSDKNRQLHSMHRFQSVTVNYCREMYNFSLEIQR
TNS
WGYLTPNGHFDGLVGLLERRLVDFGSSPLIYKLDRMPVIDYSYGNWVLRSTFIYRRP
KIIE
ASYKIFLRPLSRTVWICIVLMMVLLMLFLKVVFSREKRLLQKRNLVDSSWSFLFLFT
LGAF
CQQGATCHPQLLSSRTLSIFVFLFCILTYQFYSASIVSYLLIDPPRKINNLKDLSDS
NLRAGIE
DILIDRNYFVQTTDPVAIELFNKKIKFSNNNSGFYEPWDGLDLVKQGGFAFHVETST
AYPIIE
ETFTNEEICELEEVQMYRTQPMHTNLQKNSPFREMMNYCMLHLVENGLMYRLRKYWD
A
RKPMCIESAKKFTFNVGLKEFSSGLIVLSYGILISLGLLLREVIVHKK
>TcasIR64a.2
MSPPLPFMILLSVLTQTHALLDINLIENYFTEKSIKSATVFGCFRKTEQLNLVKIFS
RGSSPIS
VLNLNQAGVYQSIKSNHQQIGVVLDGDCPESESFLITVSPGFTHIAPNVVFISVRST
ETXFD
VKHHWLILSKSIQFLEKIKNAVVNINADIHVAVQSGTNWTIFDVYNPASEHGGSLKY
TRVG
FYSRGRGYNAQTNEAKYWRRKDMTGVTFKTMVVLLVPFEGPLEDYLHNDDNRNINTF
N
RFQNKLLRFCRDYYNYSMIVELGSSWGYPFPNGSFDGMVGAMEKKLIDFGSSPIFVR
EDR
ARVIDYGRNTWSWKAGFLFRSPKSRTSIEIFLKPLSTSIWLITGVLATASIVILKMV
TTFERN
RYHSTSETSWSLSFLFTLGALCQQGSPWVPKMACGRITAISIFLLSLIIYQFYSASI
VSHLLM

KPTNKIRNLKDLTDSSLKVGCEDIIYNKDLFAHTTDKVLKDLYAKKIYGKGNTSHFF
PPEK
GLDLVRQGGYAFHIEVARAYPIIETTFPDNAICELREVKLFKNTDLYNTMQKGTPFR
DMLE
SCFQRLAEQGILDREKKHWHPRKPECIQSSQAFVTFHVGLDEFYPALLVLLIGIVIS
LTVLV
VEKQIHIAREKMEREKGVVF
>TcasIR64a.3
FQLRVLMERLFFLSVLAVIIYTTNCTDNHDIITSYIKEKSVKYATVFGCFTKKEKIN
LVKIISH
ICPISVFDINRLNIENRMESRHFHTGIILDGDCPSAEKFLINCGRSYLFDVKHHWLI
VASSEKI
REKFNNVILNINADINVIIPEKPSNWSIIDVYNPASQHGGVLNFTRVGFYNKHDGYK
IKYTG
VKYWNRKNLTGVTFKSMVVVTYSKTXKNSAYTIFQLPVPFEGTLQHYLDSDDNRDVN
TF
NRFHSRLISFCRDYYNFSLDIEVSKSWGYTNEDGTFDGMVGALERKIIDFGSSPLFL
REDR
ARVIDYGRNTWILRSLIKQQFRIISNWGFSAAFIFRNPKVRTSLEIFLRPLPSSVWL
ITGLLAI
VSIIILKLATSFERRRYVYDVETSWSISVIFTLGAFCQQGSPSTPKMACGRIATFFI
FLLSVLIY
QFYSASLVSHLLNKPLTKIKNVRDLLLSPLKAGCEDILYDRDYFLHTTDKVAKELYA
KKIL
GKSNSSNFHTPEAGLKLVAEGGYAFHVETATAYPIIESTFQDQAVCELREVPLFRTQ
PMHAN
FQKKSPFRDMFDTCFQRLAEHGLLVRERKHWHPRKPECIQSSKSIRFNVGLDDFYPA
LVILL
VGIVASLLILVIEKEFRILTENPA
>TcasIR75q.1
SFLGTILTVYKQLAEKKIVLNVLTNHWKINQTKLSQHTFLVGDTLCPQFNSLLSHVS
KFFC
YQNSQQTLGQIITSSXKWLVFDQNSTVNTNDLLLDSNFAVASQISNGRFHLKLCYKR
APNE
TIKFNEIGVFSNGFEYYNHFIPTRNRSDLSGVNITVSYVVTKPDYPFDVEDYRFRHL
EAFSK
LSYAMVYPMLEMLNCTKKFIQRSSWGYKGANETQFVGGMFGDIQNGTAEIGGTVSFY
TV
DRMSVVDYLSVTTPSDLKFILRAPPLSYVNNLFTLPFDTKVWYCLYFIVGVTVLILY
VIVR
CESTYENALERRNNIDNIKPKFFDVVMLQIEAITQQGSENEPKTMSGRIAVFIVFLV
LMFLY
TSYSANIVVLLQSTSANINTLQDLLNSKITLGVEDVVYSHHYFETQTEFTRKSIYEK
KVAPK

NQKSNFMTTEMGIEKMKDEFFAFHVETTAGYKQIMDTFQEHEKCGLIEIDYLNVLYP
SITI
RKNSPYKEIVKVNFRKIYESGIRHRQLNRIYYKKPHCVGKGGSFKSVGIVDIYFSVE
IFAIG
CFMALWLLLLEVLFKKKIKFLVQ
>TcasIR75q.2
MKILIVFICLLINETTQNNFTDNLIVNTFNFIKILNVPVKISAHICWTRGKFDSLLM
KLYXTV
LANTIHFIKSISDKYNTNLIKNVSPKYANPEHQLFIIDLKCNDSLSVLQQAEKFKLF
KSPFK
WLLLGNSESLPNLYFGTDSQIFVTEPRSQLDDIKTIYKYSPMVPRFVQHSFDRFYTN
TKRT
NLMGTTIKISYVITNLDSLNHLWDYRLQELKKKLYHFLICRNSHIDAINKLNYILVH
NLMD
FLNASRQFTMQPTWGYKNSTTGLYSGMAGDLQKGLADLGGTPLFFTPDRIDIIDYIA
ATTP
TYMKFIFRAPPLSYVTNVFTLPFDSAVWHYCFVMVAVVVVCIYVIVVWEWKETKFEE
KD
THSHIDTLRPNIFDVVMFEIGAITQQGTNAEPKSNSGRIITIFSFLTLMFLYTSYSA
NIVALLQ
STSDSIKNLEDLLNSRIKLGVEDIVYAHYYFENAQEPVRKAIYQQKVAPKGQKPNFM
TAEE
GIRKVQQGFFAFHVELSTGYKIIGEVFQEGEKCGLKEIEYVNLIEPWLATQKKSPYK
EVMK
IGMRKMHETGVQNREIRKIYTRKPQCHSGGSNFGSVGLIDCYSAFLTFGVGIAFAFL
LFVM
ELIVRRYFIRREKERLK
>TcasIR75s
IVLPMINDLIEHFNKTQIILAYLCDKNGTNLLLIRNNNNTNFRRLSGSEPLFXKKLY
QVNVL
SPNSRDMPYPTPPAFLTYVLDAGCSNTKQLLLLVPVITHXLIFGNNILKASEQKQFA
TPFKW
IVYYNNPVELSFFIDEYFTKTNILVDSDVTLATINPTSGTFDLNKIYKRKINGSIII
ENIGIWGR
GLGVTDTGYEKITYKRRRNLTKTVLKSCIVITNNDSLNHLTDKRDIHIDSIAKVNYV
LVQH
LSDTINASLEYSVRGTWGYKDNKSQWSGMIGELTRNEADIGGTALFLTSDRIRVIDY
IAMT
TPTRSKFIFRQPKLSYVANVFTLPFDASVWASVCGLLVIIAGLLYVVVRWEWKKKDY
VQV
VVFFAFWVDFPSSVFCRTNRTSRKFTILGSXVFITFGALCQQGSSSVPFSIPGRITL
IFLLVSL
MFLYTSYSANIVALLQSSSSSIQTLQDILNSRLDVGVDNTVFNHFYFPNATEPIRRA
IYQQK

VAPPGQKPKFYPIEEGIRKMRQGLFAFHVETGPGYKFVSEIFREDEKCGLQEIQYLQ
VPDP
WLAIQKNSSYKKMLKVGLRLLQENGIQEREVGLIYTKKPQCLARGSSFISVGLVDCY
PAA
VVLAGGIGAALAVLILEIYVHQRFVGFLL
>TcasIR8a
MVISENLDKTTANRLKAIRPIPNNFAIVATSSNMEELLQTALDENLVTLPERWNLVF
LDFQY
QQFDKKRLKNMPINLLHMDEEICCRFLQSEKCECPHDFNLQENFLSLATNTLAKILK
TLTM
ENLLRADLNCDDSRYSEATRTRFYELLQQEVDSNDLVFKENFGLHVNINGVIETGDE
KVAE
YNYKTGVTVLDGKKVEPITPFFRIGITHALPWSYKETDSSGNTYWTGYCVDFTEELS
KLM
GFGYEFVEPKSGTFGKKRDGVWDGVVGDLATGETDLAITALIMTADREEVIDYVAPY
FEQ
TGITIVMRKPVRKTSLFKFMTVLKLEVWLSIVGALIVTGFMVWFLDKYSPYSARNNK
KAY
PYPTREFTLKESFWFALTSFTPQGGGEAPKALSGRTLVAAYWLFVVLMLATFTANLA
AF
LTVERMQTPVQSLEQLAKQSRINYTVVKDSDTHKYFINMKHAEDTLYRMWKELTLNA
STDD
TQYRVWDYPIREQYGHILLAINDSNPVANASEGFRIVNEHTDADFAFIHDSSEIKYE
ISKNC
NLTEVGEVFAERPYAVAVQQGSHLQDEISKTILNLQKDRFFEQLQAKYWNHSGKGSC
PTT
DDNEGITLESLGGVFIATLFGLALAMITLVGEVLYYRRKSKIQNSETKKPKTVQTSE
NWKT
DTLMPVSLINKDKQSVTIGTEFKPVNRNRDLSEFGHITLYPRARNRITQTSNE
>TcasIR25a
MASSSAIIYRIAIYSRIATAHLNYSDFLNNVLTETHKMLKLVAFILYCTNLANGQTT
QNINVL
FVNEEGNLVAEKAVDVATNYIKKNNKLGVNADPVKVVGNRTDASGLLDSLCSSYNEM
IA
NSMNPHLVLDTTMTGLASETVKSFTAALGLPTISASFGQEGDLRQWRNIDENEKEYL
VQIS
PPADVIPEIIRSLVLSKNVTNAAILFDDSFVMDHKYKSLLQNVATRHVIAPIKEADK
IGDQL
RQLRKLDIVNFFILGSFENIKRVLDAADSVGFFNRKFSWHAITQDKGELKCNCRNAT
ITLA
KPLIDAQYQDRLGLIKTSYQLNAEPEIAAAFYFDLALYSFLAVKEMIADGVWKRNNA
TNYI
TCDDFDGKNTPRRAGLNLKKYFSKEVSETPTYGPISIVSNGYSFMEFTMQISAVGVR
ESSS

DKSVPLGSWKAGYDNNLTLVDPQIMKNYTADVVYRVVTVEQKPFIIKDETAPKGYKG
YCI
DLIQRISEILNFDYEITPVGDQKFGNMDENGKWNGVVRELMEKRADIGLGSMSVMAE
RE
NVIDFTVPYYDLVGITILMKLPKTPTSLFKFLTVLENEVWLCILAAYFFTSFLMWVF
DRWS
PYSYQNNREKYKDDEEKREFNLKECLWFCMTSLTPQGGGEAPKNLSGRLVAATWWLF
GF
IIIASYTANLAAFLTVSRLDTPIESLDDLSKQYKIQYAPLNGSSTMTYFERMANIEA
KFYEIW
KDMSLNDSLSEVERAKLAVWDYPVSDKYTKMWQAMKEAGLPNTLDEAVKRVKDSRSS
S
EGFAYLGDATDIRYLEITSCDLQMVGEEFSRKPYAIAVQQGSPLKDQFNTAILQLLN
RRELE
RLKEKWWSKNPEAKKCDKQEDQSDGISIQNIGGVFIVIFVGIGLACITLAFEYWWYK
YRK
GGKVVDVQAKHSDVATKINDGFHAKINKLYPRSRF
>TcasIR144
MQVSKILLLSSLLLNRDETSKCLDAIFKQPVVVLRGVPKNLQNFDAWKPETYLILAP
NATV
LEQMLEKWSTIESFNPRAKFWLLTHWHEIKPKTLTILAKFYIVNVAIVTRTGQVFTY
YPYK
YENIAQPDTKPVLLGQCDNVPSFPDKLPKFWRNTTVQVLTKCLLPYVDCSDLDQGLE
TQI
FDLVQEFLKFKVRRIFDKSFKFGLAKINGSYSASFRFLQEREVDMAMGSFRSVGSTQ
FRDF
EFSTNHMEDKLVWVVPKARPMVHWVRLVKIFEPSFWGLLVVLTVAMARVFEKMARFT
D
EPMGIYRKSGFRVAVLILIGSYLKKTPKRFEMRIIFIFWIYFCMVLNIVFNSNLTNV
FFGTFN
TFQVNSFDDIIKSNLEMGLTDDVMHILSQEQNWPEITSTKVISSCAFGPACLNRTIF
QRNLV
CCWGERSIKFRMAKFYTTQVHYVDDHLLFFYLLFYFVKGYPIVPQISKMIVQLKSAG
FVQ
FIKSKVDKLEPRQGNELTTKILTLKRLEGPFYFLLVGWVGGIMIFGYEVVTYERKRR
KKVR
QEVTKILKKKMRQNEKVKILEI
>TcasIR41a.1
TKMLFNNFCINILVNFIINNYHKNSRCLLIFTDGDFDYKGEIPTVRIKATNGSFNSY
LIFNYH
GCQSVIIYTSNVTALLIKFETEIRLKMERFNERKFLIVPQNPSEDFDKFFNLKQLYF
ISDLLLV
LPTHNDTIFDLKTHKYVGVIDNNEPVLLDRWFSQNQSFLFGKNLYPNKLQNQLGRPL
KM

ATFTYEPYSIIGNVFEQFFENDFILQGKSVGEHHGSELMSAVQFALKYNMTPVPVIN
EKDY
WGDIFPNWSGNGLLGNLVDDKADVGFSALYTWEFCYHFLELSKPLVRTGITCLVPAP
KLS
ERWLTPLFSYSSYLWFCIILTLVIAIFVLSLVLFCYNHNKTLNLNYPLKRKTTYIHF
LESAVTI
VLKPVFQQSLTLRELPIEIASKLLMGLVLLLALFLTSSYGSGLATVMTIPTYENAIN
TVEDFA
NSGLDWGATQDAWIMSIQNAEEQRYVKIVSKFHPISEEELFQFSKSGKFGFSIERLP
FEDYA
IGDYIKEDVIDNFHLMKEDLYWEQCVIMLRKNSVLLPALDLFILKIFEAGLISHWQN
EAVD
LYMNPKVQRAVKFYRQGQEHTVVKLQWSHVKGPFALLLIGLCISFIIFILELTLKKK
RNQF
>TcasIR41a.2
TLGCLTMTNLNVLLQILLKTYFLNTRCIFLFTDSTIDLQVETPIVYFKVSNTLNPSL
IFQHHG
CQNILIHHENASDIFVQFENLIRLNNERFNERKYIVTGHNSLKILLTKQLEYVSDLL
LVVPK
QTGHYELITHVYRHQNRSKINEPVLLDVWYSQNHSFRQENDLFPNKLTNQNQRVLKI
GTL
SYEPYSVIGKLTVNXSPYYLNLGKDDYSFDGTETSLVYEFVHKYNLTPSFTIMGDDL
WGD
VYANWTGIGLFGSVLNDEIDIGYAAVYTWEEYYKFMDYTKTLIRSGVTCLVPAPQLA
AGW
VTPLRSFSLGMWIALVIVLLSNTIVLNLLFYRNQKYHXNQLFQILLFNAFSKRFFID
SLTTAI
KLYVQQPLTLTLKRGLLKYFIVTNMIMVLFISSSYSSGLSSVMTVPRYGKSIQTVKD
LASSH
LNWTGTTDAWIFSLRQVEEANYENIKNRFVVKTQNDLVTASKQYNFGFSVERLPYGH
YA
VGPYIQRDVICNYRIMQEDLYWGQCTFLLRKNSVLLPLLDKLILRVFEAGLEAYWEN
QVK
CFGRKNMNLRDFLGCLPIHGHVCPKRHYVLYTTYXEHDTIKLTWEHVEGAFAVLVLG
YA
ASIFTFVIELILDKVRS
>TcasIR68a
MIKNLLPYKCVVLISDDIYGGTFTKSWYRRFGPFITFVVIRVDEYEDLLSPFEETQA
CLDTA
KNEGCQMYLILLSNALQVSRLLRFGDKYRVINTRAKFVLLYDNRLFDKPLFYLWKRI
INVI
FIRRYSGQKSDTKKNMPWYEITTVPFPTQITSILIPRRLDIWTKSKFRKGIDLFRDK
TSDLRN
QTLKVAAFSHIPGTTKSLQEKTARTVIGNFSGTEVEILQTVSAAMNFHCELYEPVNV

DVDL
WGGKQSSGKYTGLVGEMVSTNADIALGDLYYTPYILDLMDLSIPYNTECLTFLTPES
LTDN
SWKTLILPFKYFRPAMWAAVLVCLLICGAVFHALARFHETISQNKSQVLEIHTKRKK
IIILSI
CPEIEKLDSNLKYTKMREQYKPPRFEGQSIGLYQFSEPFNSVLYTYSMLLLVSLPKL
PTGWS
LRMLTGWYWLYCLLLVVAYRASMTAILARPTPRVTIDTLQELVNSRLKCGGWGEINR
QFF
KSSLDPITKLIGENFELVNDSNEAVDRVAQGVFAFYENSYYLKEALVKRQLRFQIAR
TTQN
QSEREMRDIAREDRNLHIMTDCVIKMPISIGLQKNSPIKPRVDKYIRRVLEAGLIKK
WLQD
VMASILNAEVQSTQEEMKAIMNMKKFFGAIVALFIGYFISVVVLIVENVYFHFFVKR
NPHY
NKYTRSIHHVKKAE
>TcasIR100l
MPRKLFLWIFFLLVSCYGNLSETHLQFLKRYFVSANSVAISMLQTHHQEVKIRDLAE
VISR
KLNSIGTPVVVHENHKSGSLNIIMIVWSLKILRQFLDSLVVPEEKGTYYIIILEQDC
ATVHSD
FAQILEQFWCEHNVLNVVVQNPCSGGTFYLFLPFEHRDNFWGSCKSWDFNEQMPNKL
RN
LNQFPLKISLFLYNPTLIAKLPKGLKTNPRYHNLSASKGYGGLDGFLLRELVDYFNF
DPVIV
ENLEEYGRVLPNGTAFGSLGDVVNQRVHFSINSRFLMDYGTKEIEYTFPYISDEICM
LVPKS
LKVPTWKTLLKCFNTLSWVLIFVSCLCSTFAWYFVGPSKNLHKLIWQIYCFIVGIPQ
KIEPS
FSQFVFLLSCFFFNVTIFGIIQGSYFTEFATTSFYPDIDTLEELYESNLPVATHFWF
LLDGDTS
DLMTKLKTHKIEATGDCLEQTARQRNIATLGRKSESDLIIRTKYTSRDGTPLVHIVE
ECHTS
LYLCGIVPKGSHFLAPFNQIITRLFEGGFTTKWYRDVFDGIISEEKPQLDETVSFNS
LNMND
LQTAFHILTIGHLFSIMVLIGEVVIKGKHNKKLLT
>TcasIR100
KVTIIILIMMCLSLPKIQTCPIKINHLKEHFKQVKSARIMILQNEIIVTDWLIMELI
KDNKITV
TVQKAIRNFEPFNTSNLTRFEALEFNDTIPTLQTDSTCGHLIIVKNEERLYQYLKSD
PGFLIL
NPRHFYAIVAMELFKTNVLREFWSLQVSNILLLDCDTSYTVLPFNGTTIRINAYTQR
KLLR
NFHNYFLQVSMQPKPPTAIVKFPKPLRENPIYKDLVPFKDYAGLDGCLLKVLTQRLN

MKY
VIVGNGQKYGTVLKNGTTTGTLAWIASNKVQISTNGRFLMTYGTNKLEFTVPYSSDQ
VC
AVVPKALKIPKIIMLAKSLTPSSWFMIFLIYVICVLIYTLMGSTGSTWTLYAIFHGF
PVKIVPT
SRQSFFLTSCMLFSIIIMTIIEGSFFKTFTTTTYYKDINTLEELDESELPIAETFFS
FTNDKS
RIMTSLKRKKLVINRDDILEQVARKRNIAKLERKRDIKVRLKTEFLDEEGESRLHVV
EECFTTF
YIGFIVPKNSIFLPTFNNVIRRIFESGLTQKWYGDVEFSIFLEKIFKLENNIKHHSF
SFDNIVSA
LCVLFIGLSLALLVFFWEVTKXKQITLIYVSLIYCIISRH
>TcasIR100j
LTLVQVVICLLEVSHYDNEKFVNVYQHFTLVRYLTLTFLNDGVHRIDLNNLVVDLMS
RLN
FSMMIKEKRLGKNSTTFQESDPFQGHIMVVYDVKVLLAFLEESTEVVPKARGSFAIL
FTSL
KCPTHYETNHALKQLWTNHGTANLIAFCDNIYVYHPFSKNDSTWGATLDYSPATETP
NLF
RNFNGYLLRVSLFKRPPTALKQVPSYISNNPIYRDLKPGDFAGLDGTLLRFLSNYLN
FTVVI
DESHPTHGRVLKNGTITGSLSDVVSHRVDFSANDWFLIDYQTPEIEPTVPFSYDQVC
PVVS
KALKVPQWKAFFFIFDLTSWVLIFFMWLCCVFVWHVLNPFRDLSTIIWEICSVLFGN
PVNV
VPLSNQHMFLGSCMVLNIIIMGIIQGSVFTDFTTTTFHKDINTLEELDEAGLKIASS
AWYLD
FDTTDLIKRLKTKQIRNYIGSYKDTAFKRGMAVLGRKQDVEHMVKVEFVAEDGSPLL
HVT
SECLQTFLLVSLFPKGSPFLPTFNNVITRLFEAGLTVKWYQDVTSTGTMLQQMKNFA
NRRP
TGLFSLNDAKLAFYALFVGYIASFVTFLTEILTKNHHNNVHNHVDVLKAQHHGQVQV
DQ
>TcasIR100n
DTFWIVYQTHFLLTDYLTLHILETEDHKFELRQFTQNILKRVNKYGYFLSVRITKSS
LNKRN
KSYHFPSTAYAPSQNLAKLSDDQEFYKAKRLSTDSKHGFALIVWDLTTLHLFLDQDY
RTIV
PEGRGTYAIQVVSKQCDVKNEIAFTLQRLWTEYQVINVVAQTPCSCDKTHIFIYHPF
VKRE
GFWGLATSHTLDQIKGDSRLISNTLSDFNGFPLRISIFPRTPTAMQTLPKLLHYNPI
YRNLTW
SKGFAGLDGLVLATLAEYFNFEVVLVGSLLEDDFGKVLPNGTTVGSLADITERRAVY
NAN

ERLVAYFNLDQIDFTVPYTREDICLVVPKAAKIPKWKILFQSLDPQSWCFTLFAYVS
CFMF
WYNIGPSRSLPKVSWQMFSFFLGIPTKSFARKLDQVLFLIPCMIFSVVMLGVVQGSF
FTKL
TLFSFYQDVNTLEEMADLELPIGAFIWNLIRDDSDVIRRLKSKSVKPPDNIFDMIAA
HRNIA
TIETRARAQLLIGSKYVDDDGFPLLHIVNECLTTFLNANIVPKGSALLTVFNAVLGK
LFESG
LTRKWNNDVVDSLIAEKMISVNRKRVRTKSFSLYDAQGAFFVILVGYACSVFVFLCE
IVLK
XDKICYLALIINKT
>TcasIR100e
DDFWRVTKNHFLLVNSLTIQVLQTEEHQYDLNQYTVTLLKRLNSLNLLVALRMQEKF
LSG
RNFPKHSVTNHTFSTTKPKFDPIGGEELTQLKRLSSDSSKGYFIVIWDVESLHNFLD
EDFQV
VVPEARAXYMIHFAFTYSTEACKIVKLQVSSVLTRLWIDNNVFNIIAQTSCLCDLEV
YVHR
PFVKRGGFWGLTNSYQMSEIVENPRIIANPLINFNQFPLKIGIFPRPPTVIETLPKL
LTDSPIY
KNLSFSKGFAGVDGLVLGTLAECLNFDTTVITSKPNSYGYIYKNGTATGAIADVIDR
RMVF
SANSRFLLIYNTDQLEFTVPYTAEKMCLAVPKALKVYKWSSMFRCFNKLTWVSIICS
FGIC
TIFWYLLKWQKLVTALATIAQFLLGVPANVRPNVPQMLFLNSCMGFNIVIMGIIQGF
LFQS
FTTTSFYPDINTIEEMVDSELPLRSSIFYFLRIDNSSLIHKLKSRTMAAPPNVYDLV
AFHRNIA
TTDIKSHVDFMVRSRYLDEDGWPLIHTVDECFETFLIANIVPKGSAFLTVFNNVITK
LLEGG
LTQKWYEDVINSLILENWINLNRNKSKTHAFSLYDLQVAFYVIIMGCAVAILVFVAE
IVHKR
RNXNNCCNNHHKNIIFAA
>TcasIR100f
DDFWVIFSTHFLLATSLTFITVQTNSKQYDLRLLAQAIIQSMDKDQVMTTRHVILHN
YAEN
INFNVVFKTGTKKNARDFVTDLLAKTKKLASDSREGFVIITWNVNVLQKFLAQHISE
INPR
TRATYLFILISSSDSLRKIKHCLHFLWHKYDILNIVVHVLGCGTTTTLIYRPFCKTK
NSWGEI
TAHQIEEIVQQPLLLTNSLQDLNQYPLQVSLFARDPTALTQLPKLLQNNPIYKNLAS
FYGLD
GSMLSTMAKILNFEVVIVENHDRLPFGRVWPNGTASGTLGDVVNRRVALSSNSRILA
DYNTQ

EIEFTVAYNGDSICVAVPKSLKVPKWRVLFECFDAASWLLTLSVFIVCLCFWYCVAL
KN
FARILWDVYSFLMGIPTRIVPSRQYFFLSSCMVFNVIILQLLQGWLFTAFTKTVFYP
DLDTL
EVLEKTNLPVATNMWFLFKDNSEVIQKLSSRGIGKTPNSLDLVAYSRNICVLDKRQD
LELY
SQAKFVGPDGLSLLHIVNQCLTSVLLVNIVPKGSPFLPVFNDIMSRLFESGFTKKWY
SDVV
TSRVTEKMVSLGRKERNFSFKIKDLQAAFYVMMAGCVFSLFVFVGELVTHXVFVMNK
SS
QSKSHRFLLNCNYGV
>TcasIR100g
TLFKIAEVTFFMVTMHEEFLSLLFGNYYHTNLYQTVKIQEKFARTNNKTGAWYENVA
LDQ
KLDPPIDQNWQRVKLRTSDSFEGFIIIVWDPQTLDQFLNQNFSLVVPRARATYFLLF
VFSIY
ENCKLVNHILKRFWSEFSVLNIIAQTPYCCNKVYIHRPFVKTTNSWGVTQSYTLTEV
TQNL
ALITNPLLDLNQFPLRIALFEKNPTAIRKLPKALQNNPIYRNLSRSKGFAGSDGFLL
SAMVE
YLNFDPLIDETLEPMNFGHVLPNGTVCGVLAEVVHKRTDYAGNCRLMTYFGTDGYEF
TA
PYSSEKIAMVVPKAGKVPRWRSLFNCFNALSWSLIFSIAIVSTVFWCFLRRSQHLKR
ASWE
MFAHFVGIPCRVVPSRGQFMFLTACMMFNIIILGIIQGSFFTDFTTTSYYPDLNTLE
QVLDSN
LPIMAFAWRLLRTNSSPILQKLEQRSIPYEDNVYELVALYRNVAALDRRLDLELEIK
TKYSG
RDGVSPLHIVDESLVTFLTTSLVPKGSPFLVVFNHVIRSMFEAGLTAKWYDDVVTSL
IIEHK
HKTPSFGVKYRPFTLQDVQAAFYVIAFGYSCSVFVFWCEIIVKFSGKIKHFHYYFVL
I
>OcomIR1
MSHRWGLSGAAAVLVVGIVASKFAGVPASSTLPPQVKIGAIFTEDEKNGPNELAFKY
AVNRINKDKTLLPYTSLVYDIQYVPRDDSFHASKKACQLVQYGVHAIFGPSDPSLGA
HIHSICDALDIPHLEARLDVESDVREFSINLHPTQHLLTAAFQDVMTFLNWTRIAII
YEKEFGLLKLRDLVRSPQNSGLEIHLRQANPESYQDVLKEVKDREIHNIVIDTKPIN
LQLLLKGILQLQMNDYKYHYLFTTFDIETFDLEDFKYNFVNMTAFRIVDTEDVSVKE
ILRSMSKSQFNKDLRLLNSTFIQAEPALIYDSVFVFAVGLQTLEQSHTLKLSNLSCD
KEQPWDGGLSLINYINSVEIKGLSGPIEFKEGRRIKCKLDLLKLKQPSLVKVGEWHP
VTGVNITDRAAFFDPGTMNFTLVVTTILETPYVMMHTTNNYTGNNRFYGFCIDILDR
ISQEVGFDYLLDLVPDRKYGARDPNTGMWNGMVLQLMQHKADLAVGSMTINYARESV
IDFTKPFMNLGISILFKVPKSQQAKLFSFMNPLATHIWLYVLSAYILVSITMFVVAR
FSPCEWQNPHPCEIDNELVKNQFSLANSFWFTIGTLMQQGSDLNPKATSTRIVGGIW

WFFTLIIISSYTANLAAFLTVERMITPIENAEDLAGQTEIPYGTLESGSTMTFFRDS
MIETYKKMWRFMENRKPSVFVPTYEEGIQKVLDGNYAFLMESTMLDYIVQRDCNLTQ
IGAPLDSKGYGIATPKGSPWRDKISLAILELQEKGEIQMLYDKWWKKSGDICHRNDK
GKESKANSLGVDNIGGVFVVLLCGLAFAVIIAIFEFCYNSKKNALNDKRSASMPHQS
LCSEMKSEFCFALRCGGSRQRPALRRKCSKCMSGISYVPAMLDIPPHPPLPPARVLT
RNITSPQMDIPDRSARDPF
>OcomIR2
MHKSVIVVLLLLFASVIGGFRKKTINVGVFLDDDEDYENTVIAIASFVRRVNLFSKV
EYLLHPHIFKTQKNEILKTGQTACRLLDKGIAAIFGPESLEINDMIQSVSSTLKIPQ
FQTFWNPKLATLPVSDKSIQIFNLHPSPRALSKALATLVRENDWKKYTILYENDNGL
VRLQESLKQLSPDDPAVEFKALGPRENLRSVLKEVQSSGVAHFILDCEADHIMDILR
VAQELKLLSEFHSYILTNLDAHTLNWLELKNIRSNITALRLLDPDSTNMKNAALVWN
QNMKYGILDTVKEPFILKEIDNLRKKKPFRDIKTNNIKTKTALMFDAFNLFISTFSE
LDEKYPVKLEPLNCEANQTTEFGERFTDVLAQMKKENLLLETLTGSITEFDPTGYRQ
HYKLQIIEWVGNRFRVSGTWDPTIPQKINLTITLAEREEEIMKIIQKRNFRVVSRLG
DPYLMEAVDPTGRGLYGNDRFEGYTMDLMKEICKPESLNCTFTFELVPDGMYGNFDH
KTKQWNGIIKELLEYKADLGICDLTITYERRKAVDFTSPFMTLGIGILFAKAVKEPP
DFLTFSHPLSFEVWLYIATSYLIISLIMFLSARLNPNDWENPHPCNPYSPELENIWN
LKNCCWLTLGSFMTQGCDLLPKGICTRMVVAMWWFFVLIITACYTANMTAFLTNSRM
GVTITSAEDLAAQNKIKYGCVKDGATSSFFKDSNFSTYHRMWVQMDSADPSVFENSN
KEGVKKVLNSKRKYAFIMENSNIEYEMERNCELIQVGSNLDSKGYGIAMPFNAPYRK
SINAAILKMQEMGVLHTLKTRWWKEKNGGGQCTKDKLSADEAATEMGLDNVGGVFLV
LAVGVGLSLLIAIFEFLWNVRKVAVVQKITPKEALVKEFKFAIDIWARQKAVNRSRN
ISLEKIENETEERM
>OcomIR3
MLNKVNFLIVLIIIDLFIQSDCQTSQNLNVLFVNEEGNDIADKALDVALTYLKKNGK
LGINVDIRRVVGNRTDSTTFLDTLCSTYNLMLESKTYPHLVLDTTMTGLGSETVKSF
TAALALPTISASFGQEGDLRQWRNINENEKKYLIQISPPADIIPEIIRTIVLHQNIT
NAAILFDTSFVMDHKYKSLLQNVATRHIITPIKDGNEVVDQLAQLRKLDLLNYFILG
SLTNIKRVLDAADSLNYFNRKFAWHAITQDDGEIKCVCKNATILFVKPLPNTNYQDR
LGIMRRTYQLNIEPIITSAFYFDLFLHSFIAVKEIIASGEWKNAMTDYITCDDYNGN
NSPKRPGLDLKKSFNKENSETFTYGPLFIASNGLSFMDFQMVLSSIGVREGASDKSF
SLGTWSAGFDTNLTLANPKAMVNLTADVVYRIVVVEQKPFIFKDEKAPKKFNGYCID
LINKIAEILKFDYDLVAVDKFGAMDENGKWDGMIKELIEKRADVALGSLSVMAEREN
VIDFTVPYYDLVGITILMKLPETSTSLFKFLTVLENDVWLCILAAYFFTSLLMWVFD
RWSPYSYQNNREKYKDDEEKREFNLKECLWFCMTSLTPQGGGEAPKNLSGRLVAATW
WLFGFIIIASYTANLAAFLTVSRLDTPIESLDDLSKQYKIQYAPLNGSSAQTYFERM
ANIEARFYEIWKDMSLNDSLSEVERSKLAVWDYPVSDKYTKMWQAMKEAGLVNSLDE
AVERVRSSKSTSEGFAYLGDATDIKYLEITNCDLTTVGEEFSRKPYAIGVQQGSPLK
DQFNTAILQLLNRRELERLKEKWWNKNPEKKDCEKVEDQSDGISIHNIGGVFIVIFV
GIGLACITLAFEYWWYKHRKGGRILDVQVAPRPNKESHLPGEILFPKPKFLDNTDPR
AKNTLFQRSKF
>OcomIR4
MYFKKVFVWFTYLSVFKIGSTEMFPSLLTTNASIAIVVDRDFLRESYEDVKVEIDDY

IIYAKREILKHGGINVYPYSWTAINVRKDLTAILS
VASCRDTWRLFKRTQSENLVHMAITESDCPRLPLDTAITIPLISKGEELPQIIMDLR
GSNIYEWITLVVIYDDTLNRDMITRVIKSLTQHRTDKAKATGISLLKLDSNMSRTDL
KAKLSTINPKITGNNYLVIVRYKLVEIIIEYAKSLGLTAINDQWMYVISDTNRNFEE
IVIFENLLKEGDNVAFLYNASFTTKACVGGRKCHIEVLLKSFTTSLDQAILEEIDTS
SQVSEEEWEAIRPNKIERRDFILKNMKNYLSKNDACDNCTLWKISTGDTWGVEYQTN
KRRSIPEIQSVGFWRPSYGTQMTDELFLHVTHGFRGKTLPLITFHNPPWQVLKFNET
GDVVGYSGLIFDIVTELSKNLNFTFKLEVVNKSDINDWLTTTSDTNIDLTNNVPNIM
LDLLKNKTFALGACAVTVTEDLKATVNFTKPITILSYTFLAARPKELSRALLFISPF
TPDTWLGLAASIASMGPLLYFIHRYSPVCEYKGIPMKGGLASIQNCIWYMYGALLQQ
GGMHLPHADSARILVGAWWLVVLVMATTYCGNLVAFLTFPKIDIPITNLEQLIAHRE
TVSWSFKEGGFLEKELKVSDEYRFKEILKGKSNHKFSNIDALMNSIQQGKHAYIDWK
IKLQFLMKKRFLKTGRCDFVLGLEEFFNEKLALIVAPESPYLSKINEEIKKLQQVGL
IQKWLKDYLPKKDRCWKNKHLIEVKNHTVNLDDMQGSFFVLLLGFISALLLLFLEKL
WYKKFKKQRKKMIQPFVT
>OcomIR5
MFVRVVLIALQGVIFVNGILTGDLKIAGIFENIQLHQTAFTYSNNVYNFEKEAKDVA
VSHVVDSSILKDDPFTALKGTCLFLKQGIVGLFGPQSSLNLNIVQSITERKDIPHIL
TRWINPSHMGLQTINFYPSPSKLAEAFLDITRRLEWQTFTILYTDTDNFLQIIEFVE
RAKNDGVIVYTENVDPYKSGNYRSTLKNAVKSGQKNFVLDCPIRDLRNLLTQLQQVG
LLSTGYNFFITNLDAHTEDLIPYMYSEASITGVHLTKPNDEMTARASMELCHLYNIT
FKLECGTPEIDTETALILDSVSVFLQTLKTLEVTQGQILTCDSDDSWSYGLDIINTL
KSGSYEGLTGLIKFGNDGYRNAFQLTIYQLSGGLIERGNWNTTVGLSEDVVLTFEEE
LLQDDEESDLRDRHLKVLISLTAPYAQSRQSGRRLTGNERYEGFAVDLIEEIAEMEG
FEYTFAVREDHQHGHFDPLSGKWTGMIGDVIEGRADLAISDLTINKDRVDPVEFTQP
FMAVGISILFKKPSELPPSFFHFTEPFSLNFWQHLGIAYVVIVCALFVIGRLSPNEW
QRVETCKQSKKYLENDLTFLNSCWFVSSAVFRQTTNVRINSISGRIISATWWLFCIV
LVAMYISFSLSRDAIVIREEMFEDVPSFLEYAEANSIKYGAKKGGTTEAFFKNSKNP
YYEKIARYMEDHPDDMPSMTAEGVQRTLDGDYAFFMESATIEYTTRRHCNLTSYVGL
LDEKGFGIAVKKGSSLLAPFNRAIIKLQASGELLRIKRKWWEEKYAGDACDDDADSE
VAPKTVEHVNGLIALTFSGIAVALVTSFFEFIVHVYRLSKRIKQPYGKVFSDELKKF
FARKRTVQNMEGVALTKSENGNNSSTNKETA
>OcomIR6
MKEIPHIEVRWDDYPANGTLINLHPYPDTLTRTYYDLIVAWGWQNFVILYENTESLQ
RVGEIFKLFNPQKNRIVLRQINPLKCGGNFRPILKEVWKSGATHFLLDCSVDILEDV
LKQAQQVGLMTDKHHFIITNLDLHTIELMPYQYSETNITGMRFVDPDNKELAEKAEK
IYKQGLPIFTAYRLKLQEALIFDAVKMFSEALKASEPLVEPTIISCYNEKEKLRSGT
TIVNEMKNLDYIGLTGPIKFDIKGYRTHFSLDVFELMEGGQTVVGHWNASKSPALNV
SRMIALNEDDKDTDIRNKTFRVMITLTEPYGMRVESTNPVYGNDQYEGFAVDIIKEL
SAIRGFNYTFLIREDKKNGEYVNKTGKWNGIIGDLIDENADLAICDLTITKDRAEAV
DFTGPFMMLGISILYRKPTKAPPSFFSFADPFAFEVWKLLVVSWVGVSLILFTLGRI
SPAEWENPYPCIEEPEYLVNQLDLRNCVWFITGSIMQQGSEIELKSISTRMVAGMWW
FFTLLMVSSYTANLAAFLTTEKPNPHFTNLHELVENAHD
LDIMVGAKDGGATETFFKSKWLADENSDFGKIYTMMMKDKKKLKILDNTVGVEAARK

GYYAFFMEDQSIEYETQRKCDLNQVGDKLDEKGYGIAMRKNSTYRNSLSTAILLMQN
SGKIDQIKRKWWEERKGGGQCSSEAESGDATPLNLKGVEGVFWVTIGGTILSFFLAL
FETTLYCIKKSKRVGYSFFDALKEEIKFYFKFGKMEKEVGNPEEVAEEISDEGPMGY
SLVNKETSAETLQSNNSNKSKRSRKSKSRNNKKNMNLKPPSLSGESQIGWRHSHHEV
>OcomIR7
MKWWSLLVITVLFGVVQAGEGNIQVLFIREKGQEQFVEWYHQFIASYSSTVKIESAV
VNAEELYEDSSKICESLSTGGKLVLDISWGKNEDVKSLLLDNGVPYIKIDVSISIFL
NLLDAYLDLRNASDVTFIFEDPSYIDQTLYYWLNSAKMRMLMTETLNAATVKRLKDL
RPLPNNYALLATTNKMNKLFKTALLQNLVMIPERWNLVFLDFNYKHFEKRYLQNSIN
ILHLNSEICCYLLNQNSYCDCPSDFNLQHHYLQKVLRIIFSSVIKMFGQGLSVPNFV
CNISEPNDEFQNKLEEMLKSNIENENMLTFNYSKLSLKTSGSIKIASNESIDTIGRY
ENGIVFVPENVTVKPIKNFYGIGVTQAIPWSYKVQDPITGQWKWTGYCIDFAIKLAE
VLNFDYEFVEPKEGRFGKKINGVWDGIVGDLASGRTDIAVTTLIMTADKEEVIDFVA
PYFEQTGITIVMRKPVRKTSLFKFMTVLKLEVWFSIVAALVVTGFMVWFLDKYSPYS
ARNNKKAYPYPCRMFTLKESFWFALTSFTPQGGGEAPKALSGRTLVAAYWLFVVLML
ATFTANLAAFLTVERMQAPVQSLEQLARQSRINYTVVEGSQTHKYFINMKFAEDTLY
RMWKELTLNASTDDDRYQIWDYPIKEQYGHILLAINDSNPVKTPAEGFMNVNEHLDA
DYAFIHDSSEIKYEISRNCNLTEVGEVFAERPYAIAVQQGSHLQDDISKTILQLQKD
RFFEGLHDKYWNQSAKGDCPSTDDNEGITLESLGGVFIATLFGLALAMITLVGEVFY
YRRKQKTQENKSTLLNPQSNKTL
>OcomIR8
MLLKKITLQYLSDCTPIILANGFAEKNQLLQNLLGNFPTAFIYGQIMENYSITLDLS
IEKMQPTCFSYILFIEDVMKTKDVIGEQINSKIVVITRSSQWRVFEFLSREESRCFV
NLLIIVKSQAIVAPHEEVPYILYTHKLYVDALGSSKPEVLSSYQHNHLTKNVDLFPR
KLNNGFSGHRFIVAMSHQPPYVISNGLKTIDGENIYEGIEIRLVNILSKIYNFTTDY
REVTENVWIGSSEAVTKTVEDNRANLGIGGIYVTPDKIGRMGLSRWHSRDCAAFISL
ASTALPRYRAILGPFKWTVWLALIFIYLGGIFPLAFSDKLTLKHLLKNPEEMENMFW
YVFGTFTNCFTFTGKGSWCQADKLTTKLLIGFYWLFTIIITACYTGSIIAFVTLPVY
PSVIDTARQLVSGWYQIGVLDKGEWPFLFLNSTDEVASKLIKNMDLVPTVEEGLKNT
TKNSFWKYAFLGSRAQLDYLVRTSKTKKSKRSAMHIGKECFVPFGVSIAYPLNSIYG
EIISNGIELIKESGIMTKIKSDVEWEMMRSATGKLLSASTATAIKTLSYEDRALSLE
DTQGMFLLLAFGFVIGGVTLIFEWFGGCYKICQRKPRRGSDESIESNPRTHERQTTR
QTNFLHHYAMLQKSMESNVNSEKVKENETQENAKELEQLDQQHAISMQNNEANKYEE
NIEKIIDEIFDDALNYNNVSPKIRSNSN
>OcomIR9
MLKLHNRYFLTSMDAHTLDFSALNTTANITTIRLHDPKSDDFLNTIQRWELTEFANH
NKRVPLDPLSIKTETVLFHDAILLLTDTINAMSIK
PGISHSSLMCNGSDVSDDGFTLRNYLLINTPSLTLTGPIKFNEQGERVDFNINVVDI
IDDTLIATWFASNNSLILGRNYNQTVDAAISNLQKVMVIVSSRIGEPYLMHREPTEG
EILTGNNRYEGYSMDLIAGIAKIIGFNFQFEITEKYGNYDKVEKRWNGLIREVLEKR
AHLAICDLTITPERREVVDFSMPFMTLGIGILHKMPDKGDVNMFGFLDPFSPIVWIY
TATLYLIISIVLFFISRMTPGDWENPHPCEENPEELENIWDVKNCLWLTLGSIMTQG
CDILPKGISSRLAVGMWWFFSLIMTSSYTANLAAFLTKANLESPIDGAEALSKQTKI
KYGCLANGATESFFKNSNYSTYQRMWMNMQTTKPSVFEDSNAEGVQRVRTTKNGLYA

FLIESTQIEYETETKCDLKQIGKLLDSKSFGIAMPMNSPYRSAINRAVLKLQESGEL
GELKKKWWKLKRKEKSCEAPSEEEDSLKLGLANVGGVFLVLGIGIGMSCLFAILEFL
WNCRNISVQEHISYMHALKIELKFACNIFITKKRVKPLLSENGSSQRSDDEEKNENK
SIIQGMLQSIGSNLNITQ
>OcomIR10
MRLKEISIFLTLALSAQTRYYGSIKIISDFIVRLHEPVKVNAYLCWPQEHKIQIWKE
LSLNNVSCDIGHDSKNITYHFPPEHQLFLIDTNCNGSHNILKQGNELSLFEHPFKWI
ILGSGNNIIEEFYFRADSQIFLIEHEENGKEYNNYRIQALYKLFRDFSGFIQNDVAE
WSLKDGFKQYNEFSFSKNRTDLRGTNLNITYVILNSDTFNHLEDYRDTHIEPLAKLN
WLIMKNLLFILNATSTPVYQETWGYSRDGNTGRFTGMMGELQSGRSEIAGMALFMNM
DRIGVIEYVSPSVSTCMKFIFRAPPLSYVSNVFTLPFDTDVWYSCFGLIPLIFITIY
LIVKWEWKDPIFKENMRENNSVVPLRPGFVDVLLVELGAITQQGTESEPKSNAGRIA
TIFVFIAFMFLYTSYSANIVALLQSTTESIKTLEDLLNSRISLGAHDTVYMHHFFET
AREPTRKAIYQQKIAPKGQTPNFLTIEEGIGRVRKDFFAFHVEVSPGYKMIGETFQE
SEKCSLKEIFFVELGDPWVCIKKRSAYKEIIKVALRKMLERGIQRRDINRLYTKKPL
CQSRGSNFGSAGILDCYAAFLIFGTGAIFSCLILFLEIMINKKIKAIEMKTVIISSR
INNETE
>OcomIR11
MQFSRLLKFLFVFNIISIILVNGLIEVQFVKDYFKEKMIRYGTIIGCFSHKDYFAFT
KSLAFGNEMVAIININKIKATKDKLWFLEETKSHIGVILDGDCDEKNLKSFLVQCGW
NNYFNGKIHWLIVTTNSNFSTNFKDVKLFINADINIMYPKGDTNFYVIEDVYNPAST
KGGDLKSIYMGQYSRTLGFRIKQEKYKYWMRKNMSGVTLRTTIVLPVDFEGTLMDYL
RNEEHPEINTFNRFHYKLISSCVNYYNFTSVVSREKSWGYLKSDGTFDGMAGQLERK
LIDYGSSPLFYRVDRSKILDFARQTWTLRAAFIFRDQRTINWVEVFLKPFTTSVWLC
LLFIFVLTIVFLKFASIKEKRSIRVDAEINWSYLLLNTLGVFCQQGFDVVPLFVSGR
VTLFFVFICSLLVYQFYSASIVSSLLTKPNTKIKSIEDMLHSTLKVGCEDTLYTKDF
LRYTTNKPIQDLYLTKILGKGNTSNFLPANDGLAMVKDGGYAFHVEVATAYPIITKT
FSEQTINELREVQMYMSQPMYFNLQKNSPFKDMFNTCLQRLAEYGILNREIRFWHPK
KPEGVHSLFVLTSLSLEYFYPLLSMLLIGILISTVILVLEIAWDFRQRARETRVVVP
ILQFIH
>OcomIR12
MKLTATLVSLFSTVVLSTYVDFIINFINIYETRSVTQFSCNEKESFTLFKQLTQHST
NARVLNVDPKYVSHIGPGNRQLFILDLECDRSLEL
LQKSNELNYFMAPYKWILFHTTSLEDILDTYFHNLDILVDSDVSICNKLDNGTITVK
KIFKYRSDHPIQIENAGHWTKTEGYNDFGMEKILARRRKNLKGTTLNTCIVVLNNDT
LNHLTDKRDKHIDSITKVDYVLIQHLGDFVNATLNFSIESSWGYKMNNSKWNGMIGY
LTRKEADIGGTALFFTMDRVDVIEYVAMVTPTKSKFVFRAPKLSYVTNVFTLPFNRQ
VWISTLCLVILIGFLIYVILKWEFYKKKGSVEDEPSNANIKVDEIEVALISFGAVCQ
QGSHVIPSSLSGRITTILLFISLMFLYTSYSANIVALLQSTSSSIQTLEDLLKSRLK
IGVFDTVYNRHYFPNNKDPIHRAIYEQKVAPRGQKDNFMPLEEGVRKIRDGLFAFHM
ETGVGYKVVGEIFQEHEKCGLQEIPFFDFIDPHIAIQKNSSYAKLIKIGFRKIWESG
IQPREVNLIYTRKPICTSRSSSFITVGMVDCYAAAVVLVVGIVLSLLIWVLEMLTYN
RSKFNQTLQNKVKNKFPISFAVPFQQK
>OcomIR13

MKKIMFFLFISSIFPIYAENRNDEVIAVITDLIVKINLPVKVTAFICWSQADELLLL
KVLSNNSIPCRIEKYDIFTSYFSPPEHQLFLLDGSCNNSKYFLEKGSQLNLFRHPFK
WIIWGQRNENDLKQMYFRIDSKIFVIYKEKRCNNEIYHIQLLYKLKGALEFITNDLA
VWNSSQGFLTFNEFSFYRNRSNLMGLKLNIGFVVTDPRTYQHLEDYRDVYIDTLTKY
NWFGMKLLTPIFNVTPNNIFHTTWGYPQKDKTFNGMIGDLQFNRAELGGSSSFITKE
RIEAVDFVAPSAPTYIKFIFRAPPLTSVSNVFTLPFSSSVWYACFAIIPIILIVVFV
IVKWEWEDDKFKEKVLIHEANSISPLQPRFFDVLVMELGAITQQGAESEPKSNAGRI
ATICVFIAFMFLFTSYAANIVAILQSTTDNIKTVEDLLNSRMSCGVEDLPYQRNFFE
TAKEPIRKAIYEQKIAPKGQKSNFISMHEGISRVQKGFFAFHVEIFTGYTVISKTFQ
EDEKCSLKELVYTKHTEPWISIKKKSAYFYLFKVGLIRAIEMGIQRRHYHRLYTKKP
VCNSNANNFVSVGILDIYAAFLIFGIGTTISVTIFIFEIFMDKKIKQTQTFYGN
>OcomIR14
MQNYGFRIGSYEGKIPIYFPPEHNLFLVDADCNCSNIVLQQANQLKLFNHPYRWVIW
GETEHHTFRHLFFPVNSEVFVITKMKSNNQEIYQIKSYYKLTENSLVFLENDVAQWQ
TKNGFLMYNELGSRNRSNLMGHRIKVAYAAVFNDSYNHLGDFRDTHLDPYLKASWFL
INYVMEMANMTSNKIIQTSYGKRILNSSRFDGLLGEIQTGTAEIAGCAYLILSEGIS
IVDYVAPCCPTYLRFIFRAPPLSYVSNIFILPFKINVWYSCIGLVFAIFVVVYLIST
WEDSVPIFKLKDYRSLTSKLEPRISNVLIMELGAITQQGAESESKSFSGRIATFVTF
ISLMFLFTAYSANIVALLQSTADNIKEAEDLLNYPITLGMRNVSIVHHLFKSSTEPT
KLAIYNKILSKDIKPNVMTIDEGISRIRKEFFAFQTDASSAYKLITETYQEYEKCNL
REVAVFNIDEPYIFIKKKSFYKEFIKVGLRKIMETGLQKRAINRIYSKKPKCTGHSN
SFGSAAISDCYAAYLIFGIGICISLIIFCLEMFFHKKKFKLLVSSDICLQ
>OcomIR15
MGLAEFVITSLCLNATCNFEDYRPNISTYKQERILQLQNDLSKETLVITTLQNGELS
GYEKRNGSIIGTGIAFDIINIIKDKYRFNYTIILPEDQVFLGDGRSQNVKNLLESQK
ADLAVAFLPIIHSLRSDITFSTSFDMAQWSVLMNRPQDSAAGSGLLAPFTKEVWILI
IFSILIVGPIIYLIVLIQNRLYKDNENEAYSISTCLWFVYGALLKQGSTVNPKRDSS
RLLFSTWWLFILILTAFYTANLTAFLTLSKFTLPITSAEDIGKKHYRWVTNKANAIR
DQMIAERDDENIYKETLLDKIGTKNVFAMGEDKEILNTFVKSRGMMFIRETTVLNHV
MYNDYRKKVNNGIAESERCTYVLAKFPIAKFSRAFAYSKGFKYKELFDLSIQNLVEG
GIIQFKLRENLPDAEICPLDLGSTERKLRNADLLLTYIIVGGGLAVAACVFLMEILW
RQCQTVCCKKRQLLNNFQNQLNYNNNIYDSKSSKNFFKSNITPPPSYQTLFKPPFSY
KEEGGYQKQINGRDYWIIDTKDGFKQMIPQRTPSALLFQFSN
>OcomIR16
MKSKTFDGLSGRISFDAEGFRTDFVVDILELTFGGLMKIANWNSTTRIFDIKRSINN
QPDKADNMFNKTLIVIISTTPPYQMLKDTTTPLYGNDRYEGYGIDVIEQLSKLLGFN
YTFVLQEDGQYGILNKSTGKWNGMMGEIIDGRADLAITDLTITSERESAVDFSEPFM
NLGITILYKKPEPVPPSLFMFVSPFSTNVWIMLGLSYILVSTAIFIMGRLSPSEWQN
PYPCIEEPQYLINQFSIRNSLWFTIGALMQQGSELAPISISTRAASGFWWFFILTMV
SSYTANLAAFLTVTTLVTPFNDIDELAELAQKKQISFGAKSSGATANYFRDSNLTKY
KEIWNYMLKHPDMMMGDNDAGVEKAEKENYAFLMESTSIDYVIERHCGLAKVGPLLD
DKGYGIAMKKFSPYRNDISTAILQLQEKGILTNLKIKWWKEKRGGGKCMAKEENSEA
TPLDLQNVGGVFLVMFLGSILAAVGSLSELAWYIYKKAEKENISFKEEFKKELKFVV
KFKQNVREIDTISNKTNSDKNLIGHIKY

>OcomIR17
MRTTEETNLASISSLKKVNVVVSTRIGEPYLMNAESPDGKPLVGNDRYEGFTKDLMD
EIATILEFKYEILLTKNNIYGKFIESEKRWTGLIGDLLEGRAHLALCDLTINQQRRS
YVDFSMPFMTLGIAIMHKIPVKEEAKPFAFLDPFATSVWIYSATLYLVVSIVLYFIA
RMTPGDWENPHPCDDNPTVLENIWNLKNCHWATLGTIMNQGCDILPKGISSRLALSM
WWFFALIITNSYIANLTAFLTKANLEPPIKNVEDLGKQSKIKYGLLEGGATEQFFKE
SNISLYQKMYNDMKAQRPSVFEKDNLDGVTRVQNTKNELYAFLMESTGIEYHIQTKC
DLRQVGPWLDSKGFGIAMPMNANYRGAIDKAILELQEAGKLSILKKKWWKLKRKEPL
CEDIRSLNKDENEGDLDLARTSGIFMVLVVGISIAIVLGLTEFLWNVRNISVEEHIP
YWEALKIEVKFAANIWITKKKIKPVVSESSSTGGSKSEKDSRSMIHSFLHSASSFIN
LNQNQE
>OcomIR18
MGDVELTHERFKYVEFSFITLADSGAFVTHAPRQLNEALALLRPFQWQVWPAIGVTV
IVVGPILYALIALPNQWHPKFRVRSYSRLFFDCTWFTITILLKQTGREPRSSLKTRF
FIILLSFSATYVISDMYSANLTSLLARPGREKAIHNLYQLEQAMITRGYKLYIEKHS
FSNELLENGTGIYSRLYELMTLKQGYNNILIDSVEAGVKLVKESKNVAVMGGRETLF
FDIQRFGSSNFHLSGKLNTAYSAIALQLGCPYIEEINKILMAIFEAGIITKMTENEY
EKLGKEKEISSTEIAENVAKDTNKDLKRQIKAEEENERLKPISLKMLQG

>PstrGR2
MLKFKKMKNTVHAPVINREKEINLHTTHGCLRNLLILGKISLVFPVEGIEGKDPGKI
QFKFVSLKTFLSVIFLISTSTCFLLSIVKSLTSRNTMQRLISPLYYCHIASVAFLNL
HLSRKWKRYVMSIHAAELNFLKNYKPDKKLKRKIHATIAIVILVGLCQYSITEANFA
ISHDCLNEGAGFEQLIRKQYHYIYDYINYNVFNGVFLLLILSFASTLAWNFGDLVIIT
ISIIYTSRFNQIVDKIKWYTSYKNRNASIFSKEIEKLHDTFWRDSRRDYARMQLLCS
TTSDLFGILTLITYSFNTMFILIQLFVSLRPREYLIEILYFISSFSFVVGRAVMSSI
YGGWLYEAGRAALPIINAVPTEMYTEEASTFISQIQMSSPYLTGRKFFTITKGIILS
IAASIITFELGLIQFNQSEIERYLSTSNVTICY
>PstrGR9
MLLDHSKWIGILGVFPVYKRNLYFCMYILLRIAMCFCCLVSTHVYFKRYLDNYEMEF
GNFFFLKLASIGYELINYVNLSQVVHFREPGRALYENIQQLRFAIKKSNYTREIAGF
YLWIVVFIICVLHGSIGFVYEEWSISFWAFNAALTYLQLVGTALLLYDLAVIFTENN
DYLSIRIRRSFTSYVRNRFETQIELDSIVKCVTRMGESTGLISDFFGISIFNFVFVA
FLENLFNLNWVLSAMISNTRDIKDVADVILFVLQILIIIVLNVSSLIMVLSCDKIEA
KSNELLKHCYCLQPDVKDPILLKQLIDLTDYIKELKPKMWISGFFRVNKRLIPLMIS
TLASYLIIIIQFHKL
>PstrGR11
MIVEDLYVSTPTFEAIQPLLITSRLFGLFPISYKKNGTNYRLKWSLVYAVYSYSLCV
GLALWTMIGMSLDLKNDPEHSLRMTDEKTRFVTGGDVSIVVIIVIFATATLHLKIKK
FWKLMITLNQADSIIPLRNSKRYRDASILFIAVVVVTLSLILTFDISSWFVKLHARG
LNPLDYLQYYFAFYLNYSIMIMMEVFYWHVIYLIKIRISLLNGDLRKVKEKINFKEG
NYFFEKIVGKVYLDRFSRSSSRQTACLSSTGTIIKNEENSELGKRLIMLSILHDKIF
EAVTIVNNSIEFCIHVVMLSCLLHLIVTPYFLLKGIIKENSGTYYVLLQSAWLIGHI
GRVLIIVEPCQYCINEYKKTSNLICELLTYEVDNEVKKAMTILSLQLSYCKLRFSSC

GFFKINRSLLTSIAGAVTTYLVILFQFSDT
>PstrGR13
MCEKSAKNTENPDGITDPHSPRKKSTVKRFISGILMGSFFFAIVYFGVPTMYMTCFG
LQIKCVDEVLKIGYNLKKIQEIPFFHALNWYLLMVANYFLFGETFIQHGRVYLRKYY
LLDMLSSYHRFISFCFYFIGLVWFVVILRKKILRQQFSLLFWTHFLLIVITAQACMI
MQAMYEGIIWLIMSIWLVSINDSCAFIFGKHFGKTPLIRVSPKKTLEGFILGGICTI
ILGAFLSHFLCHFKYLVCPIKFASVDNEITYRSNCTPSYNFQPIAYAIGDFSVNYYP
FMKHSFYLSIFASVIAPFGGFCASGFKRAVGVKDFADTVPGHGGILDRGDCQYLMMT
FVNVYIMTFVKDPNVEEIFRKIAKLSDEQQLEFYSLLRSSLSDLLAGDVGNTSKLTL
YGSYF
>PstrGR15
MDEPDRNSEPYNSLHHRNPHQRTNFQRQNLPVIEVETPTEDEMQITEAEILHFYDDF
YHTTKSLLVLFQVMGVMPIERELGHTTYRWTSATNVWAYFIFAIETIFVSIVFQERL
KLVLLPGKRFDEYVYAFIFLSILVPHFLLPLGAWTNGSEVAKFKNMWTKFQYKYYCV
TKSKLVFKNFTVITYSLCVASWVIGIMVMLAQYYVQPDMLLWHTYAYYHILAMLNCL
CSLWFINCMAKGQAARWLSQNLHNALQSSDPATRLSEYRDLWVDLSHMMQQLGKAYS
GMYAMYCVLIIILTTVVASYGCLTEILDHGLSFKEGGLFLISVYCMTLLFIICNAGHQ
TSNKMGPEFRERLLNVNLGAVDERTRQEVNMFLTAIDKNPPIMNLNGYANVNRKLIS
SVITSMATYLVMLMQFRLSLMRNAAIAAKRAAIANLTAHHT
>PstrGR19
MSQDDDVVKATLPVWVFCQMFGLCFSSTKAIASTKIPSRKRNVGKFVYWFATVSIAL
HLSFYNFSYELDPSKNVVIQAGDILNGLSSIAATVSSIFTSFLFENRILTVMKDLCE
VNDKLKKYPKSISYKGIKKFAYAEIIYLLNSWIYFLISYVITCPSNLAECLGSWYLM
YTITKIFDVNLARFISHMSIVYKQLCTLNSNILHLSEERYVISLNLPKERYKIDALE
NFKEIYSEILRIGEEINYVHSFSLLLIIANQFISIFSSLYFCFFGYYINGNYIKPNS
LSTLLVPLMSLIHPGGQLLAIAIVCQLTISESKQTGKIIYRIPINRNARTLMQRVSM
>PstrGR23
MDEARMSTVKADVNILTFFFKMGKFFGVVPLHNKKSSRLKTIISLSYFAILSLLLST
IFIVSVWDRHKMYDSMKVTNIIVDVLVMITNFCFILTIRLGNLINNRYFDKIMESFR
KIDACLDQSNFTICRMRVFKIYFFIVGFHVLYLGMHAYELYYRIVNHQTSFLIWVLY
IPGFLKIHHQLFVVTLVAKMNTMLHTRYQFVIHCLGLNIDKENLNLELLELNKKSLT
LDQIYYVFKKLYDLVSDMNVLFNWQIFFVLTCTVLEILNVINFMKQDQAWSVLAVDI
AYGALYTISTLAIIKSCNDVQMLGEKVYVCCYTSQETLGGSKFENELLKFAKLIKPL
LPQYTAGGFVTVNQRILSSLFSATLTYLIVIIQFDLST
>PstrGR26
MTLYNAVVSFDEVMYRKLLKTARYMGVVPFSLKKRRISAIFAWLLIFMYVSTEIWQL
KLRVTYHRPFVNFMVVLSDIFLVINENIAMVTAFFTCLAHKKQWSTFFYRMSIITPF
IRERCEIGFRAKLAGYTQISLFGCCVVNEFFQIYYTKELAESITPYLMFFISNIYVI
FACLLLTKIVNVILKFQRNINQDISLLEEKKYLICDSLIKMFESKLILISKTIVDIN
TLFGWEMFCVFVNFVIIVMNFITTSFFLQEDWNRKSITYFVGNFFRVVVYLVNVMMI
VYKFSEIGKQNEDFIVTCFYLQEDMPNLHIRNELLHLATFAKKIIPKLTAVDYFQVN
KLMMGGLFSTITTYLVLCAQFNVGST
>PstrGR27
MDMKRKARKEDIIWDRSIHNVFSWLFFAMQLFGFMPLRGIFKRDLSAIKFTWMSWIS

LYALLTSAGMFFMLATQVARFFIYNMHTQEMQRFFYFMKSWFVSWFFFVLAREWKDM
LVRWNEVDEALARFGRPKCAKRKLNFGMVFFVAFFIGDYVLIQTQRTEQDILKHGVI
LSWKAWEHFQKHRIFKYIYELYPYNFFSSLCLLIIQLQVLFSGTFLDLFIMGTSFCL
ASRMKLITKKIKEMSFNRIESDEVWINLRESYNKLELLCCYINSKIGVAVLICFMGN
LGILLIHLYNSIKVHGVIERSYLYYSFLFLLSKTIAVCIFGAKINDESQKPLSYLFT
IDNYAYNVEIKRLTHQIGKNTMALSGMNFFYLRRNILLKIAGSIVTYELVLVQFAED
ILKETHSANRTTTL
>PstrGR29
MDGSSILSTMDFHHRRIKDTYFVDFWYKAGIALGLFPWTLQKGFMKHQIIALAFLFL
AFSILSVYIIIDKENCGYGMGLLIFTSLDNAAHGLFNLIIILYDLVIERKRWRTIKEL
FHYLDKNLNYTNYVSDTYITFYIIGSILHLLLNIYANWHFFFKNSSSVGIVSGVIWN
LYQMQKFITIGYLINVSLLLKRRIEKCCRILENLVKNPYQQKTEVAYKLMSLKNYKY
YVLIVWNLNSLFGRKLLLFLVMAFITILDEVQFILDIDMSDIKIAIVLENLTELILI
MVLTIIIAIAGDIIEKSGDKMIKLLNILQLSIEDDFLKQELSKFAALVTELRPSLSV
AGFFDVNRKLLPMLLSSFSAYIIILIQLKQ
>PstrGR34
MSMTSNVLFTIVRLQLALTNVTSIVTLVLVKRRRLVGKTRRLGNVERTLQANFQRTI
DYKPTVLTAQLSLFIGLILCIGIFDVYVTASSLQYDAFDAAIHSLYRLQVFIVALTT
AQVRCCCARIQAAVEFANERMRDTLRTVAGEATFFLRRYDELCDAVDAVSDVYGAAM
LMIVGCVVANLLAGSNALLKLLTVVRRDDALLLTSLFGIVEYAVISVMLAVPCGNAS
NEARKTCLVSYKILLGFQGSSRFGRCKNVKEELLLLAEHVRARNVRFSTGFFPVDYN
VLFLILGSVATYLIIILQFQ
>PstrGR36
MAAVERQDAAGALNRLHEGTKPVVRAAQLFGVMPLRCIAGDWDGVRFEWRSLAVAFS
ALNAAGAFLSMALWLVKFCVDGIVVDKTVYMAFYFCTFLCTVHFMKVARNWPSILKE
WSFVETTMKNYGTEENLKRKFLWMGGVSFLVGTVEHILFIINGLYQSEVCDSFSRSR
FRATMEVLFSNYFTFISFNTIAGVLIKMINVLATFTWIYTDLFISIVSHALTVKFRQ
FVTRIKTNPDRVGHQKFWREIREDYQKLYGLCKKVDKCISFLVLVSFMHNIFFLCIQ
LYNSLKQRKGLIETAYFVYSFAFLVFRIIAVSMYGALLHDEAQKPLEYLHNVPTEYY
CSEVRRLINQIYTCPVGIGSGFFVVSRNFMLQIAGTIVTFELMLFQFAPIDSKNRIY
NKTE
>PaenGR2
MLKSEKSLKLVEKITAAPPPINADFKLLTNYLGAANFFGVIYFFNKGYCFKTFGLLL
TILVAGVAFFSFK
VQKIIYENLHPTLKGMSIVLTVTDTVFLTFILVSLMLRKREHWKKLFETMHIFENIL
NTCDYLDNENGMW
RISLKIILISTTILHSIYSLYYYIFENIFEAFQIILVLRGLVNLYLILITTFYLTLT
NWLRNRYTFMHKY
LTTTITKPGYFLIVRKVIITFKLAETVVQKTNHLFGSIIFTSLAVCVINILYYFVLA
LDLTSISTEAKCI
NYIAPLIYAVFLVVLTMSCNSVETSGHSLIKTCYLLHESAELDVDKDHLMLLVKYVE
EWRPIFSAAGFYD
VNQASLSSIFSSIITYLVIAIQFNMVLA
>PaenGR3

MEKKVADLSANERKLTESYQLVVETMGVNKFFGLGPLAGKSKFSAIFFVCATLTASV
FALKNRFHSLGER
SPIVGRMALGQPLFEALFSLYCFVYFSFFAKDCKKDLIDAMNKLELSVNVRFKENFH
WLKPRIIIIILIEF
LALYIYTFYISITNNRFNILIIILSAQFIAVNCTLQICVYYLVITNWLRNKYDNFNSM
IVSICEKPYSKFD
EKIDNIIDYLEKSSTVVKLNNKLFGTSIFTSNCLFIYIILYTTVSLFDGREREDSTL
NTLKCIFPIPFVL
YLIVLAMACDAVEKSGKRVIKTCYLFVEELKDAAKKEQLRLLARYAEQWCPVFSAAG
FYDINQSCLTSIF
SAILTYFVIIIQFNMVLSDQ
>PaenGR4
MIYRNSQLLLSRTFFFYYTFLTATVIYNMALSLRNCLVGLKIYIVKSLGVESSVFLS
ETKSLRTIMTPIS
VIGEETFWKLLEASKIFNIIFECVEIFNDIFGVLILFFNVVTVLIIILVSLNTLLITG
EMEQLTVEVAIDS
ISVCLMFIIWNSFVTSACGLLKRESVTIITLCCKLQHALPHGSKERQEILNLARQIN
RKSPRVTAAGFCD
VDFSLIFSIFSCVGTYIVVLIQFSHIDF
>PaenGR5
MHEATVFKDTLIVRSFYKFGSFMGVFPSNFATGELSLRITIVVTTITLISLIRFYMY
VSQETIILQISTI
IDFAEIFSYYIFSILTLRSLIRNKRMWKEFFGTIANIDKEVGMWKEFFGTIANIDKE
VGIGYTNKYLLLL
RVLFWIALDLAASIVIFNSGTSILIAVYVIMVILQMTAISVFVLESCLLIQKRQDML
SSKLETFRQEMFG
KEFDREATSVERAIKNIYFVADTISSAFGWLFFSIFILNFIIMFVALDKMVETSFDD
TITYSEGFINNIY
NYAYLGIFFAMTISLAIAGDKIEKTGLKIKKLCHVLQVDVDNPIIKAHLREFAVYFE
ELRPVLTVSGFFV
INRNMIPLLISSLTSYAVILIQLKSGP
>PaenGR6
MENIKDVTKNWTKRKLPTKRNHLNFHPDFSQTSHDFLKYILLTCQFIGLFPMHNICG
KDPAKIKFSWLSW
KIVYTIIISASSIFCSYSGYYRAFTFHPVITHLLFPVVFTQVAVATLISINVSKNWQ
KCITSITYLELEI
NKKYGEPKNVRKYLMFLIFIIYAIGSIEHLFELISNALQFGSSGFINCIRERCNYFY
HECNTCWSAVFLV
FCTISWAVCIAIDVFIINLGVLFASRFKQIADSFESYNDALQIEETSHIYRLDNILK
YYKQIREDYIKLT
SAVYQVDCLCGNLIICSYISNITSILIQLFNSLRHFNSLLHTIHFLYSFGFIIMRLV
LVTIYASSVNEES
KKIASIVLKVPDDIWSQDLQRIISHIYYDSTALTGGQFFKITRSLTLNVDSCFHY
>PaenGR7

FRVVYSALNASGAFISFGFWLIKFCVDGLVVDKTAHMAFYISTFLCSIHLIKLAKHW
NGILQEWSYIEMS
MRGYGNDVNLKKRYLWMTVTSFTLGTIEHALFIINGIYAADACASYVISPLRATMEV
LFNNFFSFITFNV
WLGILVKLINLLATFTWIFTDIFISLVSVALTAKFRQLVVRLKKNKVMHHQFWREIR
QDYHKLYILCKKM
DKHISFLVLISYMHNIFFLCIQLYNSLRERKGVVESTYFVFSFSFLVSRIIAVSMYG
AWLHDEARKPMEY
LYNVPTEYYCNEISR
>PaenGR8
GVEHALVNAYKLKNKFDREDNFSEAVKYFFIDSYKHVFNMVDYSVWLGLLIQFLNLQ
RTFYWNYTDVFIM
VIGSVLTYKLKQISKKIQDTATSRVNDVVSWKIIRKDYVRIAELCHVVNETISGVII
TCFLMDLYFVLLQ
LYKSLRPIESVIEKVYFYISFGLLLLRIFCLCIFGGAVFEEWKDIRFYLNTIATSAY
NLEIERLTNHVAT
WELSLSGKNFFSISRGLILQMAGAIVTYE
>PaenGR11
RMNKPMSRTEMLYIGLGHLVFILTRFYYHWFKMHHVDWFVILILVYEVMQHYYFFLS
TLTICHYVRIIQY
GFKHAVDLLKQYNKSEMTDVELVEKIRYIGKDCRYLFRIGENNNDIFGWTILLIFLN
GIVQVLRTTNLML
YLLQGSHLTLGRAFIFYSKTLLCFAETIYIIISCESSSRAGGRIRLVCHKLQEGVHI
KSSARDEFFKLAT
VVTTFSPKLTAANFFVINRGTLLGIVNLSTTYLLVILQFYMRESKEAEQQMNSTRNF
NITCENESTIILQ
>PaenGR12
KKLSRVLTNLMAFERIYYKMTKKKFSQILGNKPLVITVALPVLACATMVVTHVTMAH
FKFLQVVPYCYIN
MITYLICGVWYMLCDLLGNIALTVVDDFDQILKNISPANKIAEFRSLWMLLSRIIRD
IGNAFGFTLTFLC
LYLFLIITLTIYGLMSQIQAGLGIKDIGLAITGFFAGLMLLLISDEAHYASNCVKVQ
FQKKLLLVELNWM
SDDAQQEINMFLRATEMNPTDMTLGGFFEVNRNLFKSLIATMVTYLVVLLQFQISIP
EDGDEGDSTTKH
>PaenGR14
MMFSKKVSKSKENENELICMQFMTKICALAGLFPGNSNTNINKIYVIVLTTASIVGW
IYSVYIKNNFMLT
GCNVFIKITDQISCGLLCLATVTFSLTSTFVYPGKFFNAIQSLKRFDYLINISSPRT
VSMGLAIFIFGNA
VLLFPMVLDTLAWINRYDLNTYKCYFIRNFQYYQLGVITFLWLWFALEIRKRFVLFN
KFLIKMIPTPSIL
LRNDAHHHGLFKMANIPQIRDEIKHKTALYNGLCDIVDLINDVFGAGLLIYVIFTIS
YVLLYSIIILIEYT

VFDSKTRIDNYIIITSIVWIIGDFVKMMVVALAGETLSSESFKTVRICYGIINSLEN
GPNQYYDGIKQEL
NFLIQQATHRKPCLTASGFFVANSTMMGFIIGSITSYVIVAVQFLNEMSN
>PaenGR15
FSQSKKLAKWLNEICIFDKEIQKSNFKIDFKSEFFITFLMALSGSIISLAGIVFIVT
VDVLCFYPTSFKA
ANSNISYCAPIMALSLLFLSVCSILLVLKRRFKWINCGIQTLKEQLDEKYSTDTFTI
PFRKSIKSVDLNE
DVIVYALEDLRTKHWKMHSLVEEINSIYSFPLLFSFLQIFLAFQNYTLYFIKSPTTV
YTQKTTKLYQFFG
KGIIAIYAVQMVLMSSICSSLSYEVHKTG
>PaenGR16
MNLLKILNGNGLLLKDRIASNYFQEVMKFPLKLAQTFSYFPIFIGDREEPLQFKWLY
WRIGYSLITFILF
IIELCFVVLETFKNPMNPLEIKVVVFHLGAVIQFILFFKLTYHWPNFVKEWTKIEYH
MKTYEIVENIRLK
LILIAGIIMFIAGGE
>PmacGR2
MLKSEKSLKIVGKITAAPPPINADFKLLTNYLGAANFFGVIYFFNKGYCFKTFGVLL
TILVAVVAFFSFK
VQKIIYENLHPTLKGMSFVLTVTDTVFLAFILVSLMLRKREHWKKLFETMHIFENIL
NTCDYLDNENGMW
RISLKIILISTTILHSIYSLYYYTFENVFEAFQIILVLRGLVNLYLILITTFYLTLT
NWLCNRYTFMHKY
LTTNITKPGYFLTVRKVIMTFKLAETIVQKTNHLFGSIIFTALALCVINILYYFVLA
LDLTSITIEAKCI
NYIAPSIYAVFLLVLTMSCNSVETSGHSLVKTCYLLHESAELD
>PmacGR3
NKYDNFNSMIVSICEKPYGKFDEKIDNVIDYLEKSSIVVKLNNKLFGTSIFTSNCLF
IYIILYTSVSLFD
GREREDSTLNTMKCIFPIPFVLYLIVLAMACDAVEKSGQRVIKTCYLLVEELKDAEK
KEQFRLLARYAEQ
WCPVFSAAGFYDINQSCLTSIFSAILTYFVIIIQFNMVLSDQ
>PmacGR4
MQTYYFVCVDYVSRMSTLIKMLSTILENFASLYSLTLSFFGLFLPSKDFSNWNRYEI
LRKYSGLLYATLT
TIGYCYLQWYASQNTYKTSRTSNVILSTSTDTLLTIICLISTLGNAFCKKGKWIRLL
KLQNFCRKRTRID
GFWRNKFYLVLEVLFGHAIFCFVVGAAQYKNVIEDDLSKLAATFIKNVFFYYTFLIA
TVIYNMALSLRNC
LVGLKIHIVKSLDVESSVFLSETKSLRTIMTPISVIGEETFWKLLEASKIFNIIFEC
VEIFNDIFGVLIL
FFNVVTVLIILVSLNTLLITGEMEQLTVEIAIDSISVCLMFIIWNSFVTSACGLLKR
ESVTIITLCCKLQ

HALPHGSKERQEILNLARQINRKSPRVTAAGFCDVDFSLIFSVFSYVGTYIVVLIQF
SHIDF
>PmacGR5
MHEAIVFKDTLIVRSFYKFGSFMGVFPPNFATGELSLPITIVITTIILLCLIRFFLD
ISQETIILQISTF
INLAEIFSYYIFSILALRGLIRNRRIWKEFFGTIANIDKDVGVGYTNKNLLLLRVSF
WIALDLAATIVIF
NSGTSIFITLYWIMVILQMTAISVFVMESCLLIQKRQNMLSSKVETFRQEMFEKEFD
LEATSIERAIKNI
YFVADTISSALGWLFFSIFILNFIIMFVALDKMLETSFDDKITYSEGFINNIYNYAY
LGIFFAMTISLAI
AGDKIEKTGLKIKKLCHVLQVDVDNPIIKNHLREFAVYFEELRPVLTVSGFFVINRN
MIPLLISSLTSYA
VILIQLKSGP
>PmacGR7
MHHQFWREIRQDYHKLYILCKKMDKHISFLVLISYMHNIFFLCIQLYNSLRERKGVA
ESTYFVFSFSFLV
SRIIAVSMYGAWLHDEARKPMEYLYNVPTEYYCTEISRLIDQMYTSPVGITGSGFFI
VTRNFLLQMAGTI
VTFELMLFQFAPLDSKNRAYNRSISCI
>PmacGR9
YSDMIVKKIGKHMLEDTLFLQCIFRIGSIFALFPWNVKTCEVFIQHTLMIILNIATI
TSVYVSIEKDGDS
IYSGQILVFVHSAFHLTLSISYFVTLVLNKNRWKRFFITFSRIDKNLGKCYISRKTI
ALKSMSWIFLLAL
GLSVFISLLQFFSKLVFVTYMMLFIELFIITMFIFEASEMVNNRLIKCQKYLDLTCK
NRINVKEDFIKNI
KRIESAHRDIFLIIEILNSAFGVQILLLFVYDFLETLADINYALDNNASFTIKWDAL
YLFVICAMSISLA
IAGDKIEKAGYNLINKCHILEVEVENTTTKEILQDLSSFLEDLRPTLTVAGFIDLNR
TVIPLLLSTVTSY
AIILIQLKL
>TcasGR1
MRNDHGSNTHLHPDDAIRRAKIVKVAASPTSANPDEEPDPELLDRYDNFYQTTKSLL
VLFQIMGVMPIER
SGKGRTTFRWLSSTSIYAYFIFGAETIFVTMVFKERLYLILRPGKRFDEYIYGIIFL
SILIPHFLLPVAA
WTNGTEVAKFKNMWTRFQLKYYQVTGTPIIFHNLTLITYSLCVISWAVGIGIMLAQY
YLQADMLLWHTFG
YYHILAMLNCLCSLWFINCTAKGRVAVWMCNNLHKALESRNPAKILGAYRDLWVDLS
HMMQQLGKAYSGM
YSMYCLLILLTTIVASYGSVTEIMDQGISFKEAGLFMIAFYCMTLLYIICNEGHHAT
RKMGPEFRERLLN
VNLSAVDQKTRQEVHMFLMAIEKNPPIMNLNGYANVNRKLISSNLNERNCFSNIVKL

RTLSPTQT
>TcasGR2
MEISDLAQLYGNELHIKQISKWLRGSARAQEIQKRSELDSKDGHVIDEHDQFFRDHK
LLLVLFRVLGVMP
IQRGEIGRITFGWTSIPMLYAYVFYVVTTVLVVLVGYERFDILLNKSKKFDEYIYSI
IFIIYLIPHFFIP
FVGWGVAYEVCDYKNSWGGFQLHYYKITGKNLQFPLLSTLIIIISLGCLILAVVFLL
TLSALLEGFTLYH
TTAYLHIITMINMNCALWYINCRAVGNASTALAESFQNDVDRNCSAYIIAHYRVLWL
SLSDLLQKMGNAY
ARTYSTYSLFMMANITVAVYGFTSEIVDHGIRFSFKEIGLLVDSTYCLFLLFVFCDC
SHQASLNIARRVQ
VTLLQVNLSQVDPATRKEIDIFLVAIQMNPPKVSLKGYTVVNRELVTASVATIAIYL
IVLLQFKISLLNM
RG
>TcasGR3
MPKTHRSIPKSALNSNAAGWGCLPSSSAFSAGMKSGCGSAQSMGKRLDTHLGLVRAL
SRRWPTKNTDTTH
AIQFSSALSWPQADASHPRPGCVTSRLSRTLLLLLLGSAQRRRQRRVWTTPRRPKAA
GEELGVVSTINSS
TMYHQDQAVSILGEAIPKRRSVFLESGVNSADSFKASKVGPAPPIKFINKSSTDKFG
NGAIYEVLKPIYA
LMRIVGIFPIKNTEPGMFRVAPELLGYSVVVFVVVMGYIGFIEWDKVEIVRSQEGRF
EEAVIDYLFTVYL
LPIIINPLVLYEARKLANVVTDWVNFERIYYKLTKKKLSVFFGNKPVILTVVLPLLA
CGVMVVTHITMAH
FKIIQVVPYCYINCLIYLIGGFWFMQCDVVGKVASQLAEDFQMALKHVGPSSQVADY
RSLWMLLSKLIRD
VGNASGYTVTFLCLYLFLIITLTIYGLLSQLQAGFSTKDIGLTINAGLAIFILYFIC
DEAHYASNCLRVQ
FQKKLLLVELSWMNDEAQQEINMFLKATEMSPTDISLVGFFDVNRNLFKSLLATMVT
YLVVLLQFQISIP
EEASPTNSTTITTQTPN
>TcasGR21
MTITISKELFHVLSPVLYLSRFFCLQPLKWTKTSAGNYIITKSRFYTIYTLAASCLL
VITSITGLSQVYQ
LDVIYLVRLGDTTRRFVTYSDIVVVLLPCVIGPVFALFKTNQTINYLSHLKQFDSLQ
NQPTKSTKIFQIT
ALTTFCTAFTLSMDLFLWLKLSHNYIFLLCLPYYISYWSTVVIELLFWHFVHLIQIR
ISVINKKLAKMVV
TGLNSVTTLKKPHAEVEDLVKGYEKLIEATNSINYCYGFPILVIILGCLIHLLVTPY
GLYSIIMSTGDST
SILSQTVWMTAHILRLFLIIEPCHECFIKTKETSQLICKLLCLSVNQEVKKSLEFFL
TYLGECKIEFSVY

GFTKINRELLTTIAGAITTYLVILFQFK
>TcasGR23
MTITISKTLFHVLSPVLHLSRLFCLLSVKWTKTSCGNYKITKSRFYTIYTLATSCLL
VTTSIYGLSQVYQ
LEVVYLIRLGDTTRRFVTFSDIVVVLSPCVTGPAFALFKTNQTITYLSHLKQFDSLF
NQPKKSPKIFFTT
ALAIFCTAFILSTDIVLWLKLSRNYIFLLCLPYYISYWSTMAIELLFWHFVHLIRIR
IGVMNEKLAEMVV
TGLNSETTLKKLQDLVKGYEKLIGATNSVNDCYGFPILVIILGCLVHLLVTPYDVYS
IIMSTGDSTSILS
QTVWMTAHILRLFLIIEPCHGCFVKAKETSQLVCKLLCLSVNQEVRKSLEFFLMYLG
ECKIEFSAYGFTK
INRGLLTTIAGAITTYLVVLFQFNKNG
>TcasGR26
MANCATALTLVCWGFWGFVHDMEIASFVSLGFTGSVDVVISSFDISDVLLSCLYFIV
SMPFKCAKLSIVF
HNLNKVDAIITPVFCDRFYSNLVWFSRCWFVFLPVLYTLDVFMWGNTSWLGVNNYFA
YYVSYSIVVLHEL
QYYQVVKMAQLRVSGINKTVKENIKKDTSRIKLEFIFDLIHCYNNTTDAIETINSSF
NKTVTLMLFSCYV
HLVTCPYQLFVMITSNETSILNYVYCLWVLLQIFRLVLVVEVCHNCEEEIQNTRILV
SQLLNCRLDKNVK
KEANTFLFLMVKKKIKFSAYGLPKVGRHLLLSVASSIGGYWMILLQFSSRTSKI
>TcasGR27
MQVASFTALGFRGTADFVIACFDVNDVIVSAIFFVTSTPFKFKHFVQIVENFDRIDA
RISPILVEQIRKR
SNIFVKVLVTFLPTLYVLDLFMWGKNNWEGLNNYFAFYIMYSIVVVHELQYWHIMTM
MYARILGLNKTLR
DYFKNKTGFCEHEILVVTQSFNSINDSVEEINKCFSYSTTTIIFSCYIHLVISPYQL
FVVVSSTETSLFN
YVYLLWISLHIMRVLTIVEVCQKCENENRKTRSLVYQLLLCKLNEKVKNMVRVLFFL
VTTRKILFSAYAL
PKINRRLIISILSSISTYWMILMQSTSRTIQVV
>TcasGR34
MSAKLLTFLLNGGTYLAIVPPLNKQNNYKYKFYVVYAMSAFISGSVIATLFFKDFYAD
FVLIKKAVCIVED
IFLLSLNLHILNIFGLRRREQWSALIECFKATENLVLVELNKRKVPYYLGFVIANIV
HFSIAVYEYFFWT
QSIGLSFIKKYFNRYLQMYLNFYCIFLLCVVTNMILLRYKGFKATLRTQTHTHLNKN
SLLNLISKVEKTM
YSLKKIVDLYNTMFGWTLFLIISLTTIQILNYIDFIIFYTQVEAGSVQIVLLSLAII
TWVFSGALVLILL
CSFVEQEVEEIVLLTYDIRLRVAGKINLEGYKMQEFTNFVNKNLPSFTAANFFHIGR
STILNMLSTVSTF

IIILIQFRKQ
>TcasGR36
MSFKLLKLLLRTGKFLRVTPPLSNKDTGFLQRSYILGVFFTISVGVIMSTLYKKCYL
QYIHIKIIISFLE
DIALYTLNFYASVVLNFWKAESWFHLMENLKATESLTSAKREKLPYYFGFLLLNIVY
WVLALHEIIIWYR
LTNLDYFLKQYFIVIIQLYLKFFCTFLLNVVTNMLLSRYRGVKLLLLNFLRRKKKFG
ALNTLDMMHQTET
IVHFLKKTVDMYNEIFGFPIFLTISFTTLHILNYVHYTIFYSNRFEVLDMIILNIGF
LTWNLVGAVTLIT
LCDLVQQEGEKILTLSYKLHVQCHTVEDVCRFESFLQLISNNLPKFTAAKFFYIRRS
TILDMLNTVYTFL
IIMIQFEAQ
>TcasGR57
MSLKLLKLVFKIGGLFAMSPARIEKNGLVFPSKAYSLLWIILLSAGISVTAVYRTTS
YKTLSTIGLTLQA
STDVILFILNISTILVTMTKKNKWNKFIDILNFFKNGSEDQNVFWFTPFLATNVVFV
IVMIFETYVWTQI
MTELDFFKLYAIEYFQLYAQFIVYSMICSCLNLILESSQNIYKTINFLKPKRLNNFP
LKKIKGDFRALAQ
CVEIFNNIFGWLILLSIGFTFFELLTCIQYMIVGKGNTVPVIIYRVMFLTWLMVGTF
NSVFICDSVEEKV
MNIRMVAYKTAAKCAEETEDMKKLLSAINNFPHFTAAGFFDLNRKTILGFFNAFLTF
LIVAIQFENFEM
>TcasGR59
MPLKLLKLVFKIGGLFAMSPARIEKNGLVFPSKAYSLLWIILLSAGISVTAVYRTTS
YKTLSTIGLMLQA
STDVILFILNISTILVTVTKKYEWNKFIDVLKILRNGSEDMNVFWFTPFLATNVVFV
IIMIYETYLVIQI
IGPDFFNLYAIEYFQLYAQFIVYSMIYSCLNMILDSIQNISKAINSLKPNRLNSFLL
KQIKSDFRALSQC
VEIFNNIFGWLILLLIGFTFFELLRSVQVMIMGTRNTTVATVIIRSMFIIWLMVGTF
NSVFICDSVEQKV
MNMQMVAYNTVANCAEGRETEEMKNLIYAIDHNFPHFTAAGFFKLNRKTILGFFHAL
LTFLIVAIQFENF
KM
>TcasGR60
MSLKSVNLVLKIGSLLALTPAKIKKNRVLFPSKAYAFFWIILFLTGVSVSAFYRKAL
YEEYSTIRIILQV
ATDIILLILNISTIVITRIKKHQWNKFIRILQTLKSNKESFSFLPFFITNVVLMIML
TYETYLWSSIMGV
EYYEMYAVEYFQTYAQFIVYYLIYAFLNLILDRAENLSKKVSFLKLNRLNFNLKKII
KSDFCALAECLDT
FNDIFGWLILLLIGFCLLQQLTYLHNLIIQPEIQEVKIIIYEIIYITWHMVGTFSSV

FICDLIQQKVRNI
ETAASSSDENEFKESKKLRNVIVNDHFPHFTAAQFFDLNRKTILGVFNALVTFVIVA
IQFDSVGV
>TcasGR61
MSLKLIKLVFKVGGLLAITPAKMGKRGLFFPSKAYALLWIILLTAAVVVSAVYKKAS
YENLTSVLLTLSV
ATDIILFVFNLCTIMTTVTKRHQWIKFIKILKTLQSNNNEHLFYLLPFLITNVIFVI
NFGYETYLWSQII
GAEFYKTYAVEYFQCYSQFIVYFLVYAFLKLILDNVRNISRNINRLKMQPNRLNNCA
LKALKTDFLALAE
CTDIFSDIFGSLILLLMGFCILQQLTYLHSLVVKSSRDTISILVYKIMFITWHMVGT
FISFFLCDMIEQG
VRHVEMVAHQIETNRVEEETEALEMLVDTIKHNFRYFTAARFFNLNRKTFLGTVNAL
FTFLIVGIQFENF
KI
>TcasGR66
MTIQLLSLICKFGGWMGLTPVSVEPTGFSPKGYSLLWILLFTLGVTISGVYRTDFYK
KLSPMRLIVQTCL
DFLLLALNISTILTTVRKQQQWAQLIQNLKVVATTKTKAWFLPFVVTNIIFVLFHTY
EAFVWTRIMGVAY
YEQYAVEFFQFYAQFIVYFLIYAVLEMLLQKYKTVTYVMTGQLKAQNSNLLKSVCAD
LCLLSECVDLFNS
LFGFLVLLLVALTTLQLLIYIQVIVIGTKNTIETVAYSVIFILWHIVGTFSGIFLCD
LIRREIANVQVLA
YKIEAKRRDNEIKMFVKIVDASCRNFTAARFFELNRRTILGVSNAVITFLIVMVQFE
NITI
>TcasGR72
MATKLASFIFRIGSILAVTPSYNTGEMTFVRKLYGLLFATIMTISIVVSTINRDFYR
SIPTVPAIERTIL
DINMALLNYYLIFSVMFWKRIQWKDLIKKIHVLVDKNCFPFAVIFLLIQIFDLVFLI
TAFCFKLDLMGLE
YVKKYNFVYVQDYMVFFFNSIMSFILSIVLLKYKQLYNSLLHTYRIQTIEKVQKNLR
FLKVVNESFNDIF
EWPIFLIISYTTLHLLCHFDNIFMTSMSHKYGSVPTKKIVADLSILFFCFFGTAGMI
LLCDAVLIEAEKV
LTVSYKLTEFYEEKLYLTRVVLENFPSFSAAKFFNVNRSTLLKVLTSVIGFLIVFVQ
IRQV
>TcasGR76
MDVKTIDTIFQLGKFALLTPKSRSEKTPSLGRKIYAFILMVFLTVGALVNIVCKIPH
YQTLGLMQMVLRV
LSDFSLLLSTVFALVMVKTTTRGWYHLIRNLSQVCGSGTSRKTAFVAWFVSETLILI
CMQVAFMRKAAFR
FIQTHFFQILQIFFIFFRAVTGITVLHMVKQCYHSQKEIIRKGFEALPSVEHNLLLL
KEAVSCFNDIFGW

NFFYFIIFGICRTLVLIDDNVKSPKGMITRVVYYNFSLEVLGGLVILVKFWTYALYF
IMLADKVVKEFDD
IFADFSEIFARTTNNKKYSLLNEIEQCRPKFSAARFYDINKSTVFSIISTVTTFVII
ILQFELRVEF
>TcasGR77
MSLKALDTVFKIGKYILLTPETPTTKKPSLKKRFYALLLIIIYTITSSFCMFLQSAV
FRHRSLMQNSLRV
VVSMNSYFSAIFSLIRINSTTRSWYYMVKNLSQVSSSGTNCKCTFMAWFTVVLITRL
YDTFVFMKMFGFR
FLIYSVSDLVLFFRLIFSIITAITVLQMIQQCYHFQKKTLLTRIANSVDSDQVFETL
AKLQPDFFCLQKA
VSHFNDIFGWDILFYEFFVVCRTLVYIDDTVKMPEHLRKRQHIDVVLEISLKIVLMA
FFWLLILHFTLVC
DQVLKKYEEILNILLKIKFFLRKKVNKRRSRCLRRIEQSCPEFSAARFYCINRATLF
SLLNAATTFLIVL
LQFKVY
>TcasGR79
MNSIPIETIFKFGKYTLLTPKSRIEKKPSRQKQFYALFLIFFYTFGEVFTLVLRIRN
MEYRSQTLMQTTL
RLIRDLSLYFSVIYSLLRVKRMMRSWYYLLTNLTYNNTNRKYYWLNVCCLNLSIVTI
FFNFATKKLEQFI
VRFMKYHFFGTLKLWSQLFSTFAGIEVLHVVKQCYHLQKINVQQSGIFINLPFLEHN
LIRLKNCVIYFNR
IFGWNILFGHIFTVCRTLIYIDDNVKGLGKQYNIASSKTLKLSINIISLVQLWIFQL
YLLILCDQILREF
DQIVVILSKVKSILNKKCCEKCGLKKIEFCRPTFSAARFYGIDFSNTFLGLVGAVVT
FLIVLLQFQIN
>TcasGR87
MKEVPGWRNMKPLYYLCNFLAVTPPHSLGNTVTPVSLRFKLYTIVHIFIIIALYAHS
SYGRENFIYGSMN
MTVAITDKIANFMLTFFNVSLRIILVFSKGKVIKSFFNQGYELSKQEIFNCCSKKSF
RMNFAIFNLYMVL
LLLFDAYLWISSVGVRMFQYYIGRSFTYYVCNTTIFLIFHSVLPIKRFFTSLGIAFD
NIMKNLICEIDGR
HEFFLAEFKSAKTPPNKLNCYDLRRVRKNYNSICELVDAFNNIYGLAMLEIIIVVIT
YVLNLTDLFLVYG
MSKSRNIEGVSFGTNLVILCALWITTLLLFTILLAYGCAGATSEAEKIAKICFYWLN
EIPTMPVSIKDQT
IKEELALLAQQSTSRTSKFSAAGFFPVDFTLLGFIFGSVTSYIIISIQFIE
>TcasGR97
MKNHFSKLMKNRRGRKTSKMMNGMTKQIQDVMPGGNRLKISLTLFAELTMSSQSLHS
TLETLCFLNQLIG
APFSQTRHKLYPTYCKILLFTHLTTLLFTFCNIWWRCSNSQRISTKLIKTLNYFFKV
TIIMSTILNSMIL

KKKNVAQLIPTLKEAKVMLPDVKQKWSCFTHFDSIFVLFSIMTSFIFHLTYKSGCYL
IQDLLDDVEMVQS
LIVLLTSLKFLELLSNYFDQTNNYFDIILKSEIFISDMKMRRVNQLYRTLYRILELW
NNIYGVFLLQLFL
HIMVVLIDNLLSLVEEFKLGYSFSWKVVIRSQTIAIYLVLALLLSKIGYQISEKKYK
TSKTVLNGLVEIT
LRGGKQSQLNLLLLKLKTWPNFVSASGYFRLDYGFFLSLLAAVVSYIVILLQLS
>TcasGR109
MFVVNNMVFHLSIKDINFIRPLWSFLNLLQITPYYDFDKNVTIRPLLSKIHGMILIV
IKTVWIVTMIKDE
DVFQMWKTFSFTQKFTFTLVIINTVALNILTIVKSSLLKAHSWKRMIKNFQQIDVKL
RNRGRVETSVWGN
FYFKYTLKQILFLIFCAYELYSWSNFIAKIALSSIWFSPMGDLFYEFQTFNLITCLL
QSIKVRYEVLNEK
LVMITKRKAKSLYELQEMVQIYRILGETIEIYNSLFGYQILLITFHCGLQLVGCLNF
PLVALNTSNFDIN
IILCNIIFLMIMLGCFIWMVLLMESTVQEAEKFVNFCYKLQEQVFQDSREIDVLRTV
TAYAQHFTRKFSA
GGFFYIGKKIIFSLIGHAATYLIIAVQFNERQFQK
>TcasGR110
MSLNLSVRDINFIKYLWRYYNFFLITPWHDFDNIVPCNSSICKIFATLLIFIKVYWV
SDATFLDKHLLFT
QKVNYCITTVTITTLNLLTVVKSSFLADGNKWRTLFNNLRYLDLNLHNKGKTEGSIW
KNFYFTFVLRQLF
FIMSVVYQIHVWTSKEKISFLRGFWLSPAVDMFYEFQIIELMTSVVKSLESRYADLN
ENFLQCQDEKFES
FVYYHRILGETVDLFNSLFGYQTLLITFHIGSQLVVCLNFSFGFLTPSSFTLQLDLL
ISNFWVVLVIIVS
FVRMVILVDSTVQQAERFLDLLYKMQMVFPQLRKNCFLTKFSKHNIRKFTAAGYFQM
RKGIIFSVMANVA
TYFIIAVQLNESQKTT
>TcasGR114
MNLNLSVKDINFIKYLWKYFNIYLITPWYNFDKNAFYSPNMCKIYGTLLILLKLFWL
FDVTFLHDKLNKS
YRQLLFTQKINYAFSCLNIFVLTILSIANASFVGTDNKWKILLANFRYLDLNLHNNE
KTERKIWNNFYST
FLLEQTLFFGCLCYQAHVWSRIMKITFLESLWFSPTVDRCYEFQVVTLIVAFLKCFN
NRYKELNHRLLKC
LNNNQKVLQEYQNLVCYHRLLGETIDAFNKLFGYQILLLVFHAGLQLVGCLNFPYVF
FTSASNTPEKCSV
AFSMLFSNFSVLLIFLFALVKMVVLMDSIVQEAERFVDLSYKMHVKWPQCKEIDVII
KLTKFAKQSVRKF
SAAGFFQTRKSIIFSVISNVATYFIISIQFNQSQNIV
>TcasGR117

MCLAIIILMNNKLNMVLVKSMLQRIKMEEKLVVEKFLNSLQIYLQHNQIFGFVTFTCT
RSNFRSSKLLILY
NIILQVLFVSFVSYWLYLVLEADDMLPIYKNTYLIILFADFAYLETTWICTLLKKDK
LLELFKRLIHFDT
KCQENSTVIDYKRHKKRLLCYLLARYVALALVILFSEILVIVSEQEWSFSTGLLVMI
FNSALSYKASEIV
VMLRSRFAILNKQIRFLNQYLRLKPEGRISNRRVFISFSKICYLHQHLSKSVKLFNE
VFGVSLLVLFGNS
FLSIVLALFRTAAELQASQIKWTRIAYMALASVPFIFDSIHLCDVCYSTIGTVSKAG
ELIHQIQTEDHDI
IDEIEMFSLQIANEQVEFNAAGFFPINYTLVFSIIGGATTYIIILIQLSATLNE
>TcasGR118
MRNVPQSYCFVTILFDSVNKLGTQIVNPYSVKQYVMQQQVKNFRHSVRSVFFLSEIF
GLVNLKYRETYFR
LSKTKTFCTLVTALVYCSLAIFVLCELLIEGTTSILINVPSLIIHVSTSAYVATVWI
NSVINRWKFIEFI
RKVLEFDVKCVSNYTKQQSKIHLIVRSVFVTTYLMFDYCTVLRVQRFNNYQSLAHYL
RVFFTVFNVVHCY
LASELVLMLKNRFVTLNVQLTKLTKNCATKAQSVVLGRICTLHHHLSKLVTRFNEIF
GLGLLLMFGVSFL
LITQTIFIICVIVQSEQIAWLHLLYIFLVGIMYAADVFYICHVCCSTIHEVSKAGEL
IHKIETNDHEIID
KIEMFSLQILNERAGFSAAGFFPIDYSLVFSFLGGVTTYIIILLQLSSSVPV
>TcasGR119
MIFWCNFLNTMQQQVVNKFLDSVKIVFVQSDIFCLINFNYRENHFRLSKSKFCYTFL
ATLVYSSLMSFVL
FKILLQETKLLLFKITSVMIAVSGLVYIATVWICTLMNRCKFSEFMTKLLEFDVKLQ
SDRLMIDYKTQKT
GSKIHLFVKCVFLMGYQIFDWVQKEQSLAHSVGVFLTIFNAAHCYLATELVLMLKNR
YIILNGRLIKMET
KAQSVALGKICTLHHHLSKLIRLFNEIFGHGLLLMFGISFLLVTQTIFALCVVLQLQ
ENDWLQLGYLIFV
STLYTANVVCICHVCYSTIQEVSKSGELIHKIATNDHEIIDKIEMFSLQILNERAGF
TAAGFFPIDYTSA
FSFVGGVTTYIIILLQLSASLAV
>TcasGR122
MMDRINHKKFINTTKLLLWCDQTIGLITFDINTPSFKLSKIRSFLNITASAVLLPFA
IYHIFMHIVPTKL
ISFYKSTAILEIAFEVIFIVTVWMTGAVKHSKIAHFLNKMIRLDERFQSVGLQIDTV
REKRRIKIHFFLR
SLFAIMLPLGILLISQDVIANVLLFIFIVVKSGVAFQTIEFVSIIRNRFVILNRYIE
ESISKYKHAELVM
PLCKSCDLHHRLSKLIKQLNATYGLILLLMFTSHFIFIVVSIFYMSAYLISNPIMWD
RVMLLTFWSAFFI

VNVLYICNQCYNTVEETKQTKKIIHKIQSENMTVMDVVEMFSQQIESHNICFTAVGF
FRIDHTLLSTIVG
GATTYFIILIQLSSSLLPNDPKNTTS
>TcasGR133
MSVLKQIPIWLKCLFWVFGILQLSENNEETLILKVVPIPFWCYYTAIVIRLWTSMSS
SQFYYYGISDILQ
YTDILCDLMVITSLGIHFVFFYKRNTKLQNLVVEIDRIEIKHTKQKSINWLRIINLS
GIFLNLFLLFSGI
GVIVDIEYQLTYYFGSFLSGLDVILVGVFLDNLKNKFKWINQRFFETKQETDLVQIF
PSGHLLRARTKRK
TIFQIQNLAQIHYSLVTLVHKTIRIFEITILFGTASWFTIIINEIYYLIYMVSQYSS
KLDMSHWAQLPLV
VHYCIWIYFQIRIWNDLQEEANKTVFYVHDIWNLLAIKDENNTDMRYLHLISTKLMS
MKLELTAKGFFPL
NWSLLYTIIAAVTTHVIILIQFKI
>TcasGR137
MSVFTETPLYLKLIYKLTGVYHNSGKNNFFSVVTSIVWCLPFYSYFIYLCIDFNLLS
HTSSDVFVYIEFC
SNLSTIMFTTISFFVFNVRRNRIRALLKKVKNLRLGVISRSTKPNWPRMALIGSIVV
NTALIPFKKCAKI
NYTIYTWFPALISTSEVMYLNVVLNYFNEKFDSINHNLQNLLITSFLLTQSDKLKSY
EEDDTNYEIQHIQ
DLSLVHYKLVQQVSEFSDIFGITIILSLILWFENIIETFYFLIYMMVNGSDSLIYES
AVYGCYVVFAISW
MFILIFSFSTVQNKANATVIYVHDIGNKYLLSGKMNVKTQHLSFISARLLSMKLQFT
ALHFFNLDWTFCH
MLVAAVATYVVILVQFRI
>TcasGR153
MTIITRSNNVVGGKFYKKRNKVSNFGLTFEFIFGIIILYTTYSMSFVKRNHVRDVVL
QLNRIDELLAKMK
QKFRYTRAVWYQLIIFFSGFLMILIIASMQIQNIRIENFPPLSLFMCIIFLFPLVIL
YDLNSQYGFTAIL
IYERCFIKCKTTQKHTFRYFKQNNDFDGNPRNLMQGGKHHKLKLQHSDATSHNARIF
HQSLLNFLLLLDG
NVTVVDTILNLQIFQHSSKPFSASCWLAWGFLKSYEILHVTISCHLASQQANIVGRK
VHKVLIRTQNDEI
EEKLLMFSKQVQHSSFKFTLCGLFNIDAGLLFNMIGSSTTFIVIMIQFQETITPTIC
VSNKKAMF
>TcasGR154
MSTQDIYDAVYPLLLTTASFGLSAIFVETKNNTRKLAVFNLLKILNIIYLSLFSALL
YVAFTSLKNTHLH
VNYNSGVTKIGIIFQILANIMATYVIYFINISKSHNILTCIENIKKADLMFRDLGEK
IIYRKHFLYEVCM
ILFGVLAILGRSIVTHIYMGRNIFLTENETHFALFFPLFVSYLVQINFVLLITLVHE

RFALINRLLDNFN
EEKVKHNYLSLKPPYEYQTKYNQAKIELLMELHDFLTDVGNKLNDSFSIQILSCLTS
QFLTEVFTIFYLY
YESLILKNKIAALVWLMWSIWTTLEIFYVTVNCHLTTKEAKNTGIAIHKVLMNESDP
DAKRKLMVFSQQV
NHRSLQFTACGLFYIDATLIFTIVGAAATYLLIMLQFQEGIEAQCSNNTLLN
>TcasGR155
MLRLVMVVTIRFLYYFCFSVINFVIYFRICFLKCSKFLVCHHTLTIRRTSFLEIKGN
IITGTADTVYSYG
ISLVIALIYCCNYCKRNCIIKALNKLHSVDLSLEKFGYTLPLVSVILMFVEYCNCNL
YISPVLKSYTVFC
NSVCGVEIILIMVEEYQFFMYLILLKQRFRAINTLLATNSEPINSHKIHTVLTTNCD
KGMIVKLRSLHLQ
LCSAGKLLNEYFSIQILFLVALSFVGFTTNAYYSLDVIADNFANNERGVDIIPATLI
WTVCRFIELVLIS
VICTATKNEVRATGELLYQVKDRFQSDIAVQLQLFGKQILHCDFKFTAFDFFDVDMT
MFYGVIGSAATYL
TILIQFEIAVKDYNKHSNTTSVP
>TcasGR156
MHTLQHFTIDTRSTHSMKNFKYLKVLVTFAHFICLFPITINIRKNGLNYNFTKKCYF
LRMVLIDLLIVGC
ILKHIFNILMKNVTLNDVVFIFCSAPIIITILDIEILGYVNKAKFGKLLINLYTINY
NFKESERNDYVSL
LQLVFVFCYFIYLFTVYYIYYSVHEASIINFGYTLAKFMIFTSTCIYTNLLRIIEAD
FSKLNHLLSGTEN
LDLVLPIYSQLVFMCKKINKLYGHQLLLTILTYLIWTIYEMYHLAILWSCTSTNCPR
FLIMLALSYTVIQ
EVMLFTILWNCQNTRVASEDFKAIWYSILIKKADSFCKKKLENYSLQLINHRVVFTA
MGLYVLNMEHFFS
LLGSLVTLIVILTQFSTSV
>TcasGR160
MSARNIITSLYPCYISQLLIGCAPIKITEIRSNLQIKHRRVYDYSTILILLLIISFI
YYRYSLMDFYDFN
SSALVKFMTTVYFVIVSGLISTNVIYSRVQRQVMTQFLYKIYTVDKKMRNIGIKLKY
ENIQYFSTKVLLT
GFATFTIYLICLLTTQGFQNRIQGLITYGLLYLAVLHYISSTCSFMTTIQIITNMAE
NLMKKTEDLLIYK
SFYRQIPLREMMKLHQEIYDLLQLSTKIFAAQVLFSFSVAFVIITFQSFSIICAVYN
NSPYLRYSIASAI
WIVVFVTEKMVLTFSCHKCMFRSIELRQKLGKYAVAHSKNKLLVKEINAFGLQLLHE
PLHIYAGSMFLID
LPIFTSVCGTATTYIILLLQTDHKVDLIYSMKKVFGVF
>TcasGR166
MLPKQFLKLFKDPCDVYTAIHPLFYVCTFFGLAPYSLVRVENGKKVFKFAWWPLTRN

ALLVLILLGALTY
HAIFDLISFKDSDLQQKLRYFEEVFSSLLSCCSVIFGCIFALKVIEVFKNIEEVDVA
FRSLAVWVPYKHL
YVNILIHLSGLVTIVATLTVTIIFFASYQYGTKTYSLFIVFMTVILPYFINLLMELQ
YCHYLNILRVRYQ
LLNEYLETLVQETNRTSVEGWTDVSNVKRKSKEISKLPKSMLAISDPVFIVDQVAAL
HIKLTDTAHMINY
AFCVQQLLRITVAFISIVTALFLVAINFNKSSSEENEGKTTQLDYFFTFWAFSNACE
VMAIVWITSETCE
EANTCPRILHKIRNNTTNTNLQDTIEIYSLQMYHNRLYFTVCGLFPLDYTLLYTIVA
GVTTYLVILIQFN
NSDFVQRNSTEFDNATESY
>TcasGR168
MPLLDEIPFYWKYIYKIIGIIQFPTEENASNIVPHLWPLPQNCLFIYICVKYYVSIN
HLKFLGIFYFVDA
LTSIGTALSLVISITIFIQRSRDLKKLLLQLKEIKIDSVHRKTRSKANHYLRIILII
TIFYYFLFIPFES
EPLFYTLFFIYPGIILLFDHIFLSDILEIICNEFEQINREIKYQTTSHRIIFKTKKI
NDEEINDKMVRAE
QLSLCHCDLTALTLNICHHFETTIIVAMITSFNYVTSTVYFLIYFLARSYEANLFVL
FGNNFAFLSFYVF
WLLLILEMFTRTEKEANITGRYIHDIWNKFEANGNMTKKLRHLQLVSIRFLNNKLEF
RARNFFRLDWSFC
HTMIAAITTYVIILVQFHI
>TcasGR170
MTKSTVYDTVSWLILLSLLLGLYPSYLQTVGKCQRLKTDKNCTIVIIFHLILFAVLL
YFASLEKNPLFDG
QLYSFNTFAKLLIFVVSLSGNSAVFIMLIISFMHKKSFKNFVNNVAALDETLAKLGQ
YINYETDYYVCLA
MTITGPLVIIGNIAMELWNMPRENIEPLPNVILACHMLSFLMVHQGETQFVAANVIL
KTRFKTINNILEN
LWAKKLIVVKDKKKSRSDEQTIDICMRCHDQLCDMCGAINMLFGFPIIIGCLIQFNT
IVFSFCYCYYNSK
MTKDGVYNTLFFIVYVSLLLGPHPTYFGKTGRKTVLKTNKYCNFIILFYLSVFVILV
YYASLDETPNPVT
NGKLYNFNTFAKLLILVVSLSGTFGFFGTHILSYINKASFKKIVNTVATFDETWAKL
GLEINHKEDFRVC
LVFTLTGPFFTFCNVLMEMWNIPRENIDPIPLVVVLTHLIPFMLIHQGETQFVVANI
ILRRRFALINSIL
RKLYKNKYRKLIVDGKKSEEQIVDICIRSHDKLCDVCDSVNRIFGFIIIIGCLIQFN
TIVFAFCYCYYSI
SIRPISTLGWFFWSLLRIYELARKAAFAHLNSNEARATLNWVSKLIIRSNPSLEEKL
QIFALQLTHRAPT
FTALGLFPINGSFAFTVVGAATTYITIIYQFQVNKPTCGP

>TcasGR179
MSNQFGYCPFLKKIIPVLKLTQTKLFVFLKCFLTTILAIFSIYNTFNKFANRLFVIR
ITAIVYIFLDLLY
IGSIVVSELTFEHTLLNIVNHLMTMEEQLPKRKSASVVMLLGASFLSHIVTIGCVIS
FVLSEGYSNEWDN
NVHVAHAINANLLKTWAHVKFTTIFFIISKDFEELNKVFYNCSNQKRRNVMHLHMTL
SNISRQMNTIFSF
VLVFSVAFYFVNLIARIIQIYCMYAFGTSRIHNHEAIGLAVETFRLLVVVWVSKRCM
CQANEIKNVVCKI
LINTRNKVQRKEFNAYALQMFHETLEITAGGCFVIDFTLIFTIITAGSNYVVIILQF
LYYDNQYTYRLRL
WN
>TcasGR189
MTRLVKSPIVLIYIYRVFGIIQFCDSRSEIAKFVSRHVCILFYLFYIYISVQCYQSF
KTIIFTGIFFYFD
FAIMCTSYVLMFTLISNFYFRNDQLEKVLFKLKSICPKSESFGSNFIRLMVTVSAFF
FIFLLFLVEDMPL
VCSMELFLPLVVASFNHVFLYDVMAAIFSKFQTINNHINSISVAEIENLIILHSELT
DMATHFFYFFEIP
TIVGLVNWFGYALNMIYYILYVLINSISLNMWFFAIFNISILSFLFFWLFSIIWALE
QTRKKAKETLSLI
HDAWNEKILKRNIDSEARHLILLSVRLFVSQLKFSARNYFNVDWSFFHLALSAIATY
VIILVQFNFKP
>TcasGR190
MWSPNDVSEILVVFCKIWTCFGLPVYKFNVQGNFCLEKSLTFFVLNLSILTVGLLLH
VVFIPVANNLWSH
ISFCQYCIHYVQTFGIVIFSHVKRKEFEEIFNDLLAIESKIRLFGGGSFGYHKLGKE
LVVFVIQQSLLVT
ILAIANFFTTAETTEIVLYLLGYYFGYLSNFHFGFFLLIMPKIGFKLYCDFINSING
QPVKNVMKMYGKL
YSLPVSLVKTLQGFILLRVFSTFTIATLDIFFAISLVIPQELPWFKTFCLIFVNILW
LSGLLLNEFLILL
YFEKMLEQKLVICRLIRNNRPVRYKYLDLIYLRFNHEPRHFTVCGLFPLKCTLIFSV
VAGVATQVTYLFQ
FVNKLEQ
>TcasGR191
MWCPNDVSDIVLAFCKVWTYFGLPIYKLKTVNETKKIIQHQKYYICVLNLVIFGSVL
LLYFVFLPVENNL
ASYVSFYEYFVNYFQTIVIIIYAHVKKKEFEDIFNELISIEGNIHYFSGRKLIYDKL
GKELVRYVVQQTI
VSILLFVFDIYSNIRSETELVFIIGYYFGYWANFHFGFFLLFLPKIVLKLYCDFINA
IKHQNAKNTIKVY
TKLHSLSVSVVKTLQVFILLRTFSTFSITALDIFFGISLLTTHHFSQFLTICLIFMN
VSWLINVLFNEFL

MLYYFEKILEQKQLIGELISENNRFCNRKELILIYLKFGHEPPFMVCGLFPLNCTLI
FSMIAGVATQIIY
LFQFIQKKDLENYLY
>TcasGR197
MWAPNELSEIIVVFCKIWTFFDLPVYKLETTNAKKRFKNKPAFYYFTVLSMLLLGVV
FYLIFFPQENSLN
YYSLLLQYFIIYGQSIGTVIYSQMKRKDFEDIFNDLLLMEEKIHEFGQTNTNYHTLG
KQIVACVVLQSVL
VLLATAFDVYLNMDSGPYRMYISGYYLGVLTSYHFGFFLLVMPVIIVKLFNDFISSI
KEQTTEYIVEIYL
DLFKLAKRIFQTLQGFILLKITTTFTTTTMDLFHCSYSLEIIEIPFVVGLIMVIGNV
LLTMTTLLLDFAM
FFYYHLILEKKKILSDIVKKNETNFYKNKKVDLVYLQLHQKPTTFMVCGLFPMNCSL
GFSMIAGITTYVI
YLVQFTPKSDIMF
>TcasGR204
MYHYYYEYLKSSRYLRIFCTLPVTYDKNRKPRPRFRPFPFLWFYAFALVFQFSIMSF
AATDIILELRTEK
VTKVAVKLVKIAVTLDILVVVYKSRNKFVDCMNSVLYYEEQAEKNNIVMRYRYSLRI
LLLMKKVMVLSTS
FIGNLSCFMTGNTYISCVVSFITYNVTYLISSIVLTQFCGLILVARQHLIFVNRALF
NLSKDPQQAIEKM
NELMTYHFEVCDMTIKINQAFSAHLLLIIVRTFVEFFNSLHSIVKEKSHLLVMYDFF
WSTCAIFEFIFML
FACKRATETVIKLGSFVLRFLIKRWLQCSRTGRILNLLQTHNNNKLNLLRNVFLLQI
RHKHVTFSANSLF
SIDNKMFLHLISTSVAYLLIIIQFEKE
>TcasGR205
MSFAATHIILELRTEKVTKVAVKLVQIAVTLDILVVVYKSRNKFVDCMNSVLYYEEQ
AEKNNIVMRYRYS
LRILLLMKKVMVLSTSFIGNLSCFMTGNTYISCVVSFITYNVTYLISSIVLTQFCGL
ILVARQHLIFVNR
ALFNLSKDPQQAIEKMNELMTYHFEVCNMTIKINQAFSAHLLLIIVRTFVEFFNSLH
SIVKEKSHLLVMY
DFFWSTCAIFEFIFMLFACKRATE
>OcomGR1
MPPDGQTHNDFIIKKNHFHDSIKFIVCWAQFFGVMPLHNINKTWDMVYFKWYSFRVV
YSVLNTCGALISFGFWMVKFCAEGLVVDKTAHMAFYISTFLCSMHLIKLSRHWNDLL
KEWSYIEMCMRSYGNDVNLKRRYLWMTVTSFTLGSIEHVLFVINGVYAADACPSYEM
SPLRATMEVLFSNFFSFITFNVWIGVLVKIINLLSTFTWIFTDVFISLISVALTAKF
RQLVLRLKKNRVMHQKFWKEIRQDYHKLYVLCKKIDKHISFLVLISYMHNIFFLCIQ
LYNSLRERKGIAESTYFVFSFSFLVSRIIAVSMYGAWLHDEARKPLEYLYNVPTEYY
CTEISRLIEQMYTNPVGITGSGFFIVTRNFLLQMAGTIVTFELMLFQFAPLDTKNRA
YNRTEA

>OcomGR2
MFTKKSPKKKESKLPCIHFILKLCTVVGVFSVNKKNKFNKIYVLLLMIASIVGSVYS
ICAKDLKEFNIFITLFDNVSCGFLCLATVTFTITSTFVYPGKFLDVIECLKRFDMLS
NGASPPTTVYKFVRFIVFAHVITLVPIILDTFAWIVLYSLQSYKNYFVRNFQYYQLG
VIIFLWFWFAVEFKKRFTLLNHRLIKLIPVPTNILLKNDKESNEFFNLTIHYQVRDE
IKHITALYSGLCDALDLMNNIFGAGLLLYVIFTISYILLYSIIFISYKVFWIREIGM
GEYIIYTSIVWIIENFIKIMVLALIGESLSNEAMKIVNICYGMINSLDNSPNDNCNE
IKKNLKFLIQQAAHRKPCLSASGFFVANSTMTGFVIGNITTYLIVAIQFLSQMVENK
NTKDE
>OcomGR3
MDYKSSKMEKSKSEDMDNLPKQTLPPPPAKQNALKRTVTGLSLLAGFGLIVYGGVPV
IILTAILIQVKCYQEIIKIAYQIKKIPDIPLFRTINWYFVITVNYLYFGEILAKPTQ
VFINKYHLLQLLVRYHRFVSFIWYFLGIIWFLAMLKKRLIRQQFSLLFWTHFILIAV
SLQCYLTLQNMFQGLIWIIIPIWLIILNDVFAYTFGRLYGKTPLINLSPNKTVEGFI
LGGLSTFILGAALSFIFCHFQYLVCPIKYVEVGDSIVASTNCTRSYLFEPIPYYIGN
TGLYLNYYPFIKHSLYMSIFASIIAPFGGFCASGFKRANGVKDFGDLFPGHGGLTDR
FDCQFLMMTFVNVYISTFVKCLDVDSVYEQIMDLSEEAQLEFYYKLKETLGNSVISN
>OcomGR4
MSLYLKDFNIEYLYSLLKFSEKFAVTPPLNRKKQEFVHFKYFHLLVIAIIIVGSVFS
CYGIIVHFVPKFKGSVSVLRLLCVIFFAVLNLMSVLVAAWNVDTWNTFLKLFKRLDQ
KLNNKCGKEAFNLTNKIALVLFHVGLICIFGYDAYVWYTNYRWALFKFYVYRYISLL
QCNVIMLVVVHFASALSSRYKDINTLLIKNSDLHNVFNCLIPPTRSVLRLNNLKNIK
EVAESYLVLSELVEMFNTLFGWQIFMVSEYIILFLLEFSNNLMLSLDNTQKSSEVSH
NFRTVLTLILFSLLTIFYQSIVIIYCENVNKESRNTLTICYNLQDIVDPMSEERKEL
NILSDVVNLLKTRIHAARFYYIDRRVLGRVFTYLFSYSIVLIQF
>OcomGR5
MEHEIGLTLSWNSLKCVRALQIISQFFGVVVPNLYEYKGVSVFYIIYSVFLCFLTLT
VLSILSYIKLKDIYINVALTTKVVITYDAISLIFTNIILIIFPIFIKRKKMVYLVNH
LHNLEKHINLRFQDKVIYDSKKLCLQLIIYIGFLFSIHIFGICLSLFKAAPANEHLY
IFYFYYPFQNVMILATLQIHFYSSRIRSLYKFLHTNMYKSLHYHDLVLDVASTRIDV
LCFFRKYEDLCNAILMVNDIYGFQIFTIICYVVFNIINITHMLIKFCIKVKIFHIGE
QFSLLFLVVQIMYYALFVIVLSISCGNTSIEAKKVSNISYKLLFTFISNPISLKHYK
NIKEELLILVEHIHGMKINFSTGFFRINYNTLLLVV
>OcomGR6
MVKFEKSLEVKEIHVRTPRSINGDFKLLTSYLKVGNFFGITYFYANTSYYFKIFGSL
LTILLAVVAFYSLKVQRIIYKDLHPTIQGMSLVLTVSDSLFIFYFLVSLMVRKRGHW
NKLFETIHTFESILNTYEYLENGKMWKIILKLLLIFTTILHTLYSLYYYTFKVIFKE
FQMILILRGLTNLFLIFITTYYLTLTHWLDNRYTFMCKYLKMMRNKPGYVVIIRKVI
TTFKLAETIVDKTNHLYGSILFASLTLCIINILYYFVLALDLTSISLEVKGINYLAP
LIYVIFLLVLTMSCNTVEINGHSFINTCYFLHESTESDIERDHLMLLVKYIEEWRPI
FSAAGFYDVNQSSLSSIFSVIVTNLVIAIQFNMVLS
>OcomGR7
MLKLYRSEHLIENQADYKVLKFFFNFGSYFGGTSLKYKKSNGFYYLNFAVVLLFYVL
YLYRFCIISLTPSEPLFKFVTIVWRVCLVLIATYFVFWPHCFYHNEFEQIYRKLKDL

EDILRALKFDQKNGVKKLKIRIFLVVLVWVFAHLIGSSVVTFLTQPIEVLFFTVPNI
IFIILLFMNIFLISIVLERRMNYATKIVKKCSQYKDLERNDLPLIKNVLKLNQEIIE
LYNKLFGYPISFFLTASLLQLLRYVFMTIYFNKDESDNALSAFEMVISWGMTITIII
HCDYLEKRGKLFYAKCFKLHENTKTYDINNEYLLLAETAKKLVPNIIVPLYGLINRQ
TFNVLFSAFVDVLVILIQFQMSM
>OcomGR8
MFPLNNILHTKLASVEFKWKSPWTIYAIFIIMCTFFFVITSLVRICVNEFKLHLVDS
FLFSVFSLLILLAFFKIAMSWPKLAYDFKKIEGIFRNCGYQKKCKPIFTLMLSLSTI
SMMMEHSLAVTNTYFNIDPAKYQELNRTRTEQYFVEEFPFVFDYLPYYFLLGMLAFF
VNLCNVFSWSFIDSFILIMSYALAYRFRQIHYNVKYGINKKITSTIFWGKLRQHYTQ
MAVLCQRVNDTISNIILISFGANMYFIVTQLYKGLVTRRTTVDQCYFLYSFGLLLLR
CNLVLYFGSMVQVESKKPLDYLICIRDEAFNVETQRFLLQIHTQDTSLSGGQFFTVT
RGLLVEVSKSSSNH
>OcomGR9
MYIITIFYVFYYQRFILEHSNYGSGVLFIGVVIISATNIITSWVIYFMNLLKLRHII
GILDQIAAIDELGIMPHDYSSLIKFQLGNILWTLVYFLIGLLQFYHINRKHKVINAD
TFLICDIPSVFRYINDCFYLNIVKILFAKTKLLNGKLASIFNDDLKINQKGKLLFVK
EYQKPNDSQLDKLETLMNMYQKLIRIQMNINKTFSVQIFVTIANLFFNVLFLGFNCY
YEFKIQQRFISSMNWLGCLLIVSSEIIWIVTLCHRTTKQLQHTGTIIHKILDTKEVV
>OcomGR10
MVINESVCFHLLNMFTVTRYTNSWKKLIRLINFINKNLHHNINTIIHSIKIIMFLLI
YLSTNYFYILEPFNNVIDVLLGFFDTMVYGEVTLSTVLYWEVLKFLEIRYVLLKKLA
NGLFLHHSRNGNYHKTLLNIKTNVFFLHQVTIYLNKVLGKQILVLVLLSFSQILGFY
NSSLTNKVTTFILIETPEIFLPIIFSVVIILASDKVEKRANDLIRTCRYIQATKGDE
IAGALTNLAFELRPKFTAAGFFNINQGLLTTFFSTLSTYLIIIILQFKLS
>OcomGR11
MVAITLNRYVLISCQQHYNRLYSKISIWIQLLFIWGIAFLLMIPALTGIWGQLGLNP
STFSCTILSKDGKSPKKTIFLIGFILPCVVIILAYSCIYYSVRKSRKKLKLHRPITE
KRSSRRERDDSRLTKLMAIIFLCFLICFLPLMLVNVLDDVDIKYPTIHVLASIATWA
SSVINPFIYAASNRQYRSAYSKLFVKVKSSVIFFDSKHNSNIKADKNSVTYKPSGSV
THS
>OcomGR12
MRYFLMFLPHVIGMYHQLFVINLVTKLNHAVEIRYIFLRKCIASNAGKIDFMETKDN
IISLDQIFQISKLMFKLCRMVNDIFGPQIFFILSRTVLETLTIINLLKSDMEWSSIA
SDLAYGTTFVISTIFIVKSCDNVVNSAMNILQTCYTNQETLKGTLPENKLLRFAKLN
KHLIPRYSAGGFADIDQKLLSALFSATLTYLIVIVQFDLSSKS
>OcomGR13
MALSLTNSLVGLKMYIMRSLDVESSVILPESKSFRSVSIIDDHAFNKLLEASKIFNI
IFESVEIFNNIFGVLLLFLNVVTVLVILVSLNTLLITGETDQLTVDVAINSICDFLL
FLTCNSFVTSTCCGLKRESVLVIKLCCKLQHVLPYCSKERQELLNLARQVNRKSPRI
TAAGFCDVDSSLIINTFSYVGTYIVVLIQFSHIDF
>OcomGR14
MNSYDILDNLKLKLILISGIIMFIAGVEHGLVNAYKLKRELDQEDDLDEALKNFFTT
SYCHVFNKVEYSFWLALLIQFLNLQRTFYWNYNDVFIMLIGTVLTYRLKQITKKIQD

TATIRVNDIVTWKTIRKDYVRVSELCHIVNKRISSIIIICFLMDLYFVLLQFYLSLR
PIESVVEKVYFYLSFGLLIMRIFCMCFLGG
>OcomGR15
MLFMCGVCCLAVATYAYYNNIIIFDNANMVSDDGIKETYPHSWVPTVFLILFGFFAY
AGIKVLPFILIGEVFAVEIRAFSSGLCAGVGYVFGFITNKTFLTLVNNFGIPVVFWF
YAAAALLGTVTLYFVLPETEGKSLYEICEHFAGRSRLQNSVRRNKKPMGTSDGVNND
AFEANERSESVL
>OcomGR16
MFNRMLALLLISLIFTNFAESLPAIVGTSRQLLLAAPDRIPVYIRSGDTPLDDISHD
LAEAFNFYAQKNNRITFGRFLKDKPDNDAPVKSELRENTMLSETAKFPLDLDIDDDK
DTNNPVNDPQISKDKDSKPHTNIQRIPRP
>OcomGR17
MLSCLLHLIVTPYFLLVEILGNGDIFFILLQVIWLLTHIGRVLIIVEPCHTCINEHE
KSSNLICEILMTGVDEQVKNALTVLSLQLNYCKLNFSSCGFFKINRSLLTSIAGAVT
TYLIILFQFKDT
