# Supplementary material for: Identification of candidate chemosensory genes of Ophraella communa LeSage (Coleoptera: Chrysomelidae) based on antennal transcriptome analysis
Source: Sci Rep. 2019 Oct 29;9:15551. doi: 10.1038/s41598-019-52149-x (PMC6820725; doi:10.1038/s41598-019-52149-x)
Supplement: Supplementary file 2 — Supplementary Material S2 [file 41598_2019_52149_MOESM2_ESM.pdf]

Identification of candidate chemosensory genes of *Ophraella communa* LeSage (Coleoptera: Chrysomelidae) based on antennal transcriptome analysis

Chao Ma[1], Chenchen Zhao[1], Shaowei Cui[1, 2], Yan Zhang[1], Guangmei Chen[1], Hongsong Chen[1, 3], Fanghao Wan[1] and Zhongshi Zhou[1*]

Supplementary Material S2: Primers used for qPCR expression analyses.

OcomOR1-F	ATGTCAGCAATGTCAAAAAG
OcomOR1-R	GTAACTACAGCGCCAAGCAC
OcomOR2-F	TTATTCGATTGCGATTGG
OcomOR2-R	AAACAGCGACGATAGTGC
OcomOR3-F	TCCCTTATGTTCACAACTATCT
OcomOR3-R	AATTCATCTGCAAAAGCGACTA
OcomOR4-F	TATCTAGTTTCTACAGCACCAA
OcomOR4-R	TTCATTACCGAACCAACA
OcomOR8-F	ACCGAGAAGAGTGGAAATG
OcomOR8-R	AATACAGCGTTGGACATAA
OcomOR12-F	GTTTTGGATTTATTCAGACGAC
OcomOR12-R	AGTGAAAATAGTTAGAGCAACC
OcomOR13-F	TTATAGCCCGACGAGCCCT
OcomOR13-R	GCACGTAGAATACCAAAAAGCG
OcomOR15-F	TGAACGCTAAAGTTAGACAG
OcomOR15-R	CATAATCAGATAATCGTGGT
OcomOR16-F	TTTGAATAAGCCAGTGGGAGC
OcomOR16-R	TTCCGTATCAGTCATTATCGTTTG
OcomOR17-F	GAGAAGAGGAAGGCAGAA
OcomOR17-R	CGTAGCGAGCAAGATAGA
OcomOR18-F	TAAGTATGTTGCTGGTGAAG
OcomOR18-R	AAAATAAGACAAGTGCTGAA
OcomOR19-F	TTCCAGCATATTGAAACCC
OcomOR19-R	CGTATCTTGGCACAAACTC
OcomOR20-F	ATATCAAAGAGTGCGGAAGT
OcomOR20-R	TAAGGGTTTATGGGAACG
OcomOR21-F	GATTCCGACTTGGTTGCC
OcomOR21-R	TCGCTGCTGTTCCATAAA
OcomOR25-F	ACCGAAGAATATCGAGCAA
OcomOR25-R	ATAGGGCATCCAAACGAG
OcomOBP2-F	ATAAACCTGATAAAGCTCCAC
OcomOBP2-R	TACGCACTGGGAAATACAC

OcomOBP10-F	AAACTATTCACCCAGATGT
OcomOBP10-R	AACCAAATACCAATTCACT
OcomOBP19-F	AACGCAAGAGCAGGTAATA
OcomOBP19-R	TTTCATCATCGCCAATCAC
OcomOBP20-F	TTTCTGATGATCCTGCTT
OcomOBP20-R	ATAACCTCGCCACTTTCA
