# Supplementary material for: Identification of candidate chemosensory genes of Ophraella communa LeSage (Coleoptera: Chrysomelidae) based on antennal transcriptome analysis
Source: Sci Rep. 2019 Oct 29;9:15551. doi: 10.1038/s41598-019-52149-x (PMC6820725; doi:10.1038/s41598-019-52149-x)
Supplement: Supplementary file 3 — Supplementary Material S3 [file 41598_2019_52149_MOESM3_ESM.pdf]

Identification of candidate chemosensory genes of *Ophraella communa* LeSage (Coleoptera: Chrysomelidae) based on antennal transcriptome analysis

Chao Ma[1], Chenchen Zhao[1], Shaowei Cui[1, 2], Yan Zhang[1], Guangmei Chen[1], Hongsong Chen[1, 3], Fanghao Wan[1] and Zhongshi Zhou[1*]

Supplementary Material S3: The Blastx match of *O. communa* candidate chemosensory proteins, sensory neuron membrane proteins, gustatory receptors and ionotropic receptors.

The Blastx match of *O. communa* candidate chemosensory proteins and sensory neuron membrane proteins.

Unigene ID	NAME	Length	ORF (aa)	Status	Signal peptide	Blastx best-hit	Species	Score	E-value	Ident	Accession
**CSP**											
**Cluster-9750.63730**	**OcomCSP1**	**1321**	**261**	**Complete ORF**	**Y**	**chemosensory protein 3**	***Pyrrhalta maculicollis***	**334**	**4.00E-110**	**68%**	**APC94216.1**
**Cluster-9750.29843**	**OcomCSP2**	**1534**	**137**	**Complete ORF**	**Y**	**chemosensory protein 9**	***Pyrrhalta aenescens***	**178**	**2.00E-50**	**84%**	**APC94297.1**
**Cluster-12049.0**	**OcomCSP3**	**483**	**135**	**Complete ORF**	**Y**	**chemosensory protein 8**	***Pyrrhalta aenescens***	**167**	**2.00E-50**	**66%**	**APC94296.1**
**Cluster-9750.42480**	**OcomCSP4**	**632**	**134**	**5' missing**	**Y**	**chemosensory protein 5**	***Pyrrhalta aenescens***	**260**	**3.00E-86**	**89%**	**APC94300.1**
**Cluster-9750.33095**	**OcomCSP5**	**1249**	**131**	**5' missing**	**Y**	**chemosensory protein 2**	***Pyrrhalta aenescens***	**194**	**2.00E-57**	**70%**	**APC94295.1**
**Cluster-9750.36295**	**OcomCSP6**	**668**	**129**	**Complete ORF**	**Y**	**chemosensory protein 10**	***Pyrrhalta maculicollis***	**154**	**8.00E-45**	**71%**	**APC94219.1**
**Cluster-9750.32800**	**OcomCSP7**	**1392**	**128**	**Complete ORF**	**Y**	**chemosensory protein 4**	***Pyrrhalta maculicollis***	**205**	**2.00E-61**	**77%**	**APC94217.1**
**Cluster-318.0**	**OcomCSP8**	**500**	**127**	**Complete ORF**	**Y**	**chemosensory protein 9**	***Pyrrhalta maculicollis***	**231**	**1.00E-75**	**82%**	**APC94215.1**

**Cluster-9750.58022**	**OcomCSP9**	**740**	**124**	**Complete ORF**	**Y**	**chemosensory protein**	***Galeruca daurica***	**209**	**7.00E-66**	**78%**	**ARM20140.1**
**Cluster-9750.53229**	**OcomCSP10**	**485**	**121**	**Complete ORF**	**Y**	**chemosensory protein**	***Galeruca daurica***	**194**	**3.00E-61**	**81%**	**ARM20143.1**
**Cluster-9750.46863**	**OcomCSP11**	**748**	**118**	**Complete ORF**	**Y**	**chemosensory protein 11**	***Colaphellus bowringi***	**163**	**7.00E-48**	**78%**	**ALR72525.1**
**SNMP**											
**Cluster-9750.32917**	**OcomSNMP1a**	**2616**	**535**	**Complete ORF**	**N**	**sensory neuron membrane protein 1a**	***Pyrrhalta aenescens***	**887**	**0.0E+00**	**84%**	**APC94303.1**
**Cluster-9750.37085**	**OcomSNMP1b**	**1880**	**530**	**Complete ORF**	**N**	**sensory neuron membrane protein 1**	***Phyllotreta striolata***	**707**	**0.0E+00**	**62%**	**ANQ46504.1**
**Cluster-945.1**	**OcomSNMP2**	**2014**	**518**	**Complete ORF**	**N**	**sensory neuron membrane protein SNMP2**	***Colaphellus bowringi***	**521**	**5.00E-176**	**50%**	**ALR72544.1**
**Cluster-9750.65642**	**OcomSNMP3**	**2290**	**505**	**5'missing**	**N**	**sensory neuron membrane protein SNMP3**	***Colaphellus bowringi***	**673**	**0.0E+00**	**61%**	**ALR72545.1**

The Blastx match of *O. communa* candidate gustatory receptors.

**Unigene ID**	**NAME**	**Length**	**ORF (aa)**	**Status**	**TMD**	**Best blastx-hit**	**Species**	**Score**	**E-value**	**Ident**	**Accession**
**Cluster-3550.0**	**OcomGR1**	**1265**	**405**	**3' missing**	**8**	**gustatory receptor 7**	***Pyrrhalta aenescens***	**547**	**0.00E+00**	**86%**	**APC94345.1**
**Cluster-7383.0**	**OcomGR2**	**1304**	**404**	**Complete ORF**	**7**	**gustatory receptor 14**	***Pyrrhalta aenescens***	**380**	**5.00E-126**	**59%**	**APC94341.1**
**Cluster-9750.31200**	**OcomGR3**	**2177**	**399**	**Complete ORF**	**7**	**gustatory receptor 1**	***Monochamus alternatus***	**384**	**5.00E-125**	**60%**	**AIX97155.1**
**Cluster-15681.0**	**OcomGR4**	**1171**	**386**	**3' missing**	**7**	**PREDICTED: putative gustatory receptor 39b**	***Nicrophorus vespilloides***	**86.3**	**7.00E-15**	**25%**	**XP_017776029.1**
**Cluster-5196.0**	**OcomGR5**	**1200**	**378**	**3' missing**	**6**	**PREDICTED: putative gustatory receptor 2a**	***Tribolium castaneum***	**119**	**2.00E-26**	**29%**	**XP_015840061.1**
**Cluster-17208.0**	**OcomGR6**	**1391**	**378**	**Complete ORF**	**7**	**gustatory receptor 2**	***Pyrrhalta aenescens***	**466**	**7.00E-160**	**69%**	**APC94332.1**

**Cluster-13505.0**	**OcomGR7**	**1298**	**365**	**Complete ORF**	**7**	**gustatory and pheromone receptor 39a-like**	***Anoplophora glabripennis***	**65.1**	**1.00E-07**	**27%**	**XP_018566104.1**
**Cluster-9750.33312**	**OcomGR8**	**1256**	**356**	**5'missing**	**5**	**gustatory receptor for sugar taste 64e-like**	***Leptinotarsa decemlineata***	**235**	**2.00E-70**	**37%**	**XP_023022942.1**
**Cluster-7591.0**	**OcomGR9**	**990**	**285**	**5' 3' missing**	**4**	**PREDICTED: putative gustatory receptor 28b**	***Tribolium castaneum***	**84**	**2.00E-14**	**26%**	**XP_015837372.1**
**Cluster-11998.0**	**OcomGR10**	**1122**	**277**	**5' missing**	**5**	**gustatory receptor 1**	***Pyrrhalta aenescens***	**96.3**	**1.00E-20**	**70%**	**APC94331.1**
**Cluster-12804.0**	**OcomGR11**	**2418**	**231**	**5 'missing**	**4**	**gustatory receptor 5**	***Anomala corpulenta***	**301**	**1.00E-91**	**64%**	**AKC58582.1**
**Cluster-16652.0**	**OcomGR12**	**1055**	**214**	**5' missing**	**0**	**gustatory receptor 68a-like**	***Anoplophora glabripennis***	**301**	**4.00E-27**	**36%**	**XP_018567270.1**
**Cluster-12678.0**	**OcomGR13**	**838**	**206**	**5' missing**	**3**	**gustatory receptor 4**	***Pyrrhalta maculicollis***	**292**	**6.00E-94**	**69%**	**APC94249.1**
**Cluster-12025.0**	**OcomGR14**	**665**	**201**	**5' 3' missing**	**3**	**gustatory receptor 8**	***Pyrrhalta aenescens***	**249**	**3.00E-80**	**76%**	**APC94346.1**
**Cluster-17813.1**	**OcomGR15**	**935**	**183**	**5' missing**	**4**	**gustatory receptor 4**	***Colaphellus bowringi***	**186**	**1.00E-54**	**51%**	**ALR72530.1**
**Cluster-14617.0**	**OcomGR16**	**576**	**143**	**5' missing**	**0**	**gustatory receptor 1**	***Colaphellus bowringi***	**93.6**	**4.00E-21**	**54%**	**ALR72527.1**
**Cluster-8535.0**	**OcomGR17**	**635**	**126**	**5' missing**	**2**	**gustatory receptor for sugar taste 43a-like**	***Anoplophora glabripennis***	**186**	**1.00E-53**	**55%**	**XP_018574194.1**

The Blastx match of *O. communa* candidate ionotropic receptors.

**Unigene ID**	**NAME**	**Length**	**ORF (aa)**	**Status**	**TMD**	**Blastx best-hit**	**Species**	**Score**	**E-value**	**Ident**	**Accession**
**Cluster-9750.60840**	**OcomIR1**	**3080**	**931**	**Complete ORF**	**4**	**ionotropic receptor 3**	***Pyrrhalta maculicollis***	**1754**	**0.0E+00**	**94%**	**APC94260.1**
**Cluster-9750.40878**	**OcomIR2**	**3131**	**926**	**Complete ORF**	**3**	**ionotropic receptor 7**	***Pyrrhalta aenescens***	**1533**	**0.0E+00**	**84%**	**APC94352.1**
**Cluster-9750.36273**	**OcomIR3**	**3318**	**923**	**Complete ORF**	**3**	**ionotropic receptor IR6**	***Colaphellus bowringi***	**1422**	**0.0E+00**	**81%**	**ALR72535.1**
**Cluster-13573.1**	**OcomIR4**	**2911**	**892**	**5' missing**	**3**	**ionotropic receptor 93a isoform X2**	***Anoplophora glabripennis***	**1069**	**0.0E+00**	**61%**	**XP_018576793.1**

**Cluster-9750.5340**	**OcomIR5**	**2720**	**886**	**Complete ORF**	**3**	**ionotropic receptor 6**	***Phyllotreta striolata***	**1116**	**0.0E+00**	**59%**	**ANQ46498.1**
**Cluster-9750.16790**	**OcomIR6**	**3032**	**837**	**5' missing**	**3**	**ionotropic receptor 8**	***Pyrrhalta aenescens***	**1493**	**0.0E+00**	**88%**	**APC94353.1**
**Cluster-9750.22975**	**OcomIR7**	**2480**	**821**	**5' 3' missing**	**3**	**ionotropic receptor 8a**	***Colaphellus bowringi***	**1084**	**0.0E+00**	**66%**	**ALR72538.1**
**Cluster-11817.0**	**OcomIR8**	**2646**	**712**	**5' missing**	**4**	**ionotropic receptor 4**	***Pyrrhalta maculicollis***	**1141**	**0.0E+00**	**75%**	**APC94262.1**
**Cluster-18656.0**	**OcomIR9**	**2170**	**680**	**5' missing**	**3**	**ionotropic receptor 1**	***Phyllotreta striolata***	**1040**	**0.0E+00**	**78%**	**ANQ46493.1**
**Cluster-9750.53719**	**OcomIR10**	**2165**	**633**	**Complete ORF**	**3**	**ionotropic receptor 1**	***Pyrrhalta aenescens***	**821**	**0.0E+00**	**71%**	**APC94347.1**
**Cluster-9750.60331**	**OcomIR11**	**2033**	**633**	**Complete ORF**	**4**	**ionotropic receptor 6**	***Pyrrhalta aenescens***	**748**	**0.0E+00**	**75%**	**APC94350.1**
**Cluster-9750.65280**	**OcomIR12**	**2052**	**632**	**5' missing**	**4**	**probable glutamate receptor**	***Anoplophora glabripennis***	**689**	**0.0E+00**	**59%**	**XP_018562688.2**
**Cluster-9750.64092**	**OcomIR13**	**2021**	**624**	**Complete ORF**	**3**	**ionotropic receptor 5**	***Pyrrhalta aenescens***	**781**	**0.0E+00**	**66%**	**APC94348.1**
**Cluster-9750.13671**	**OcomIR14**	**2102**	**563**	**5' missing**	**3**	**ionotropic receptor 2**	***Pyrrhalta maculicollis***	**775**	**0.0E+00**	**62%**	**APC94258.1**
**Cluster-17558.0**	**OcomIR15**	**1864**	**555**	**Complete ORF**	**3**	**glutamate receptor ionotropic, delta-1**	***Leptinotarsa decemlineata***	**659**	**0.0E+00**	**58%**	**XP_023022733.1**
**Cluster-6047.0**	**OcomIR16**	**1811**	**541**	**5' missing**	**3**	**glutamate receptor ionotropic, kainate 2-like**	***Aethina tumida***	**706**	**0.0E+00**	**64%**	**XP_019867781.1**
**Cluster-15123.0**	**OcomIR17**	**1703**	**519**	**5' missing**	**4**	**glutamate receptor ionotropic, kainate 2-like**	***Leptinotarsa decemlineata***	**761**	**0.0E+00**	**68%**	**XP_023020738.1**
**Cluster-12980.0**	**OcomIR18**	**1018**	**334**	**5' 3' missing**	**3**	**ionotropic receptor 40a**	***Anoplophora glabripennis***	**520**	**2.00E-179**	**74%**	**XP_023310509.1**
